# Discovery of Small-Molecule Allosteric Inhibitors of *Pf*ATC as Antimalarials

**DOI:** 10.1021/jacs.2c08128

**Published:** 2022-10-04

**Authors:** Chao Wang, Bidong Zhang, Arne Krüger, Xiaochen Du, Lidia Visser, Alexander S S Dömling, Carsten Wrenger, Matthew R Groves

**Affiliations:** † XB20 Department of Drug Design, Groningen Research Institute of Pharmacy, University of Groningen, Antonius Deusinglaan 1, 9700 AD Groningen, The Netherlands; ‡ Unit for Drug Discovery, Department of Parasitology, Institute of Biomedical Sciences, University of São Paulo, Avenida Professor Lineu Prestes 1374, 05508-000 São Paulo, Brazil; § Department of Pathology and Medical Biology, University of Groningen, University Medical Center Groningen, 9700 RB Groningen, The Netherlands

## Abstract

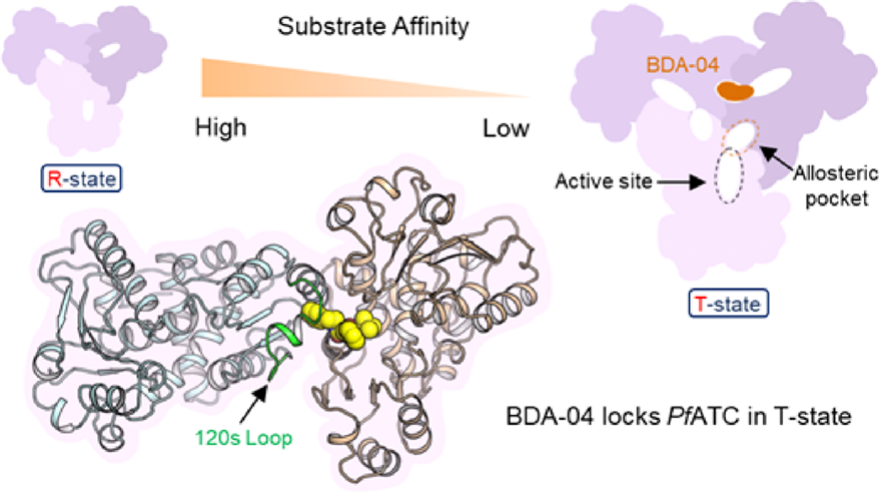

The discovery and development of new drugs against malaria remain urgent. Aspartate transcarbamoylase (ATC) has been suggested to be a promising target for antimalarial drug development. Here, we describe a series of small-molecule inhibitors of *P. falciparum* ATC with low nanomolar binding affinities that selectively bind to a previously unreported allosteric pocket, thereby inhibiting ATC activation. We demonstrate that the buried allosteric pocket is located close to the traditional ATC active site and that reported compounds maintain the active site of *Pf*ATC in its low substrate affinity/low activity conformation. These compounds inhibit parasite growth in blood stage cultures at single digit micromolar concentrations, whereas limited effects were seen against human normal lymphocytes. To our knowledge, this series represent the first *Pf*ATC-specific allosteric inhibitors.

## Introduction

Pyrimidine nucleotides play a critical role in all living organisms and are essential for the synthesis of DNA, RNA, and other crucial cofactors.^1^ There are two pyrimidine synthesis pathways: a salvage pathway and a de novo pathway (Supporting Information Figure 1). While the degree of pyrimidine synthesis is highly dependent on both the type and stage of cells, in general nondividing and slowly dividing cells rely on salvage pathways that use nucleosides derived from the hydrolysis of nucleic acids to support survival. However, the salvage pathway cannot satisfy the continuous demand for nucleic acids in proliferating cells, which then become dependent on de novo synthesis.^2^ For instance, activity of the de novo pathway is upregulated in cancer cells and blood stage *Plasmodium* parasites.^3,4^ As many parasites also lack a functional salvage pathway,^3,5,6^ specific inhibition of de novo pyrimidine synthesis can be lethal to both proliferating cancer cells and potentially other parasites without impacting the human host.^7,8^ These features make species selective inhibition of pyrimidine biosynthesis an attractive avenue to explore.

Aspartate transcarbamoylase (ATC) catalyzes the second step of de novo pyrimidine synthesis, combining l-aspartate (l-ASP) and carbamoyl phosphate (CP) to form carbamoyl-aspartate (CP-ASP) and phosphate (Supporting Information Figure 2). The ATC from *Escherichia coli* is a textbook enzyme that has been well characterized.^9^ PALA (*N*-(phosphonoacetyl)-l-aspartate), the most potent current ATC inhibitor, was first synthesized by Collins and Stark as a transition state analogue.^10^ While PALA showed promising in vitro and in vivo properties, it failed as an anticancer drug in a clinic trial^11^ and as an antimalarial drug in ex vivo assays.^12^ In depth structural analysis of the ATC catalytic cycle has been performed previously,^9^ which indicated that ATC exists in a low substrate affinity “T” state and a high substrate affinity “R” state. Significant structural rearrangements are required to transition between these states. In this manuscript, we describe the fragment-based development of a *Pf*ATC inhibitor that binds to a previously unknown allosteric pocket of *Pf*ATC. This development was driven by fragment screening using X-ray crystallography, as well as in vitro biochemical and biophysical assays. Subsequent elaboration of this compound series (hereafter known as BDA) resulted in potent inhibitors of *Pf*ATC in vitro. We also determined the structure of *Pf*ATC in complex with a range of compounds by X-ray crystallography, which supports an allosteric inhibition mechanism. The most potent *Pf*ATC inhibitors are shown to be selective between the human and parasitic ATCs and demonstrated a strong suppression on blood stage 3D7 growth in culture, while showing a limited effect on normal lymphocytes. Finally, the cytotoxicity of the most potent inhibitors in culture was assessed against a panel of human cell lines. The results reported here support the BDA series as an opportunity to develop a novel antimalarial and strongly suggest the potential of the BDA series as a tool system to assess ATC inhibition in other proliferative diseases.

## Results

### Identification of a *Pf*ATC Allosteric Pocket by X-ray Crystallography

**Figure 1:**
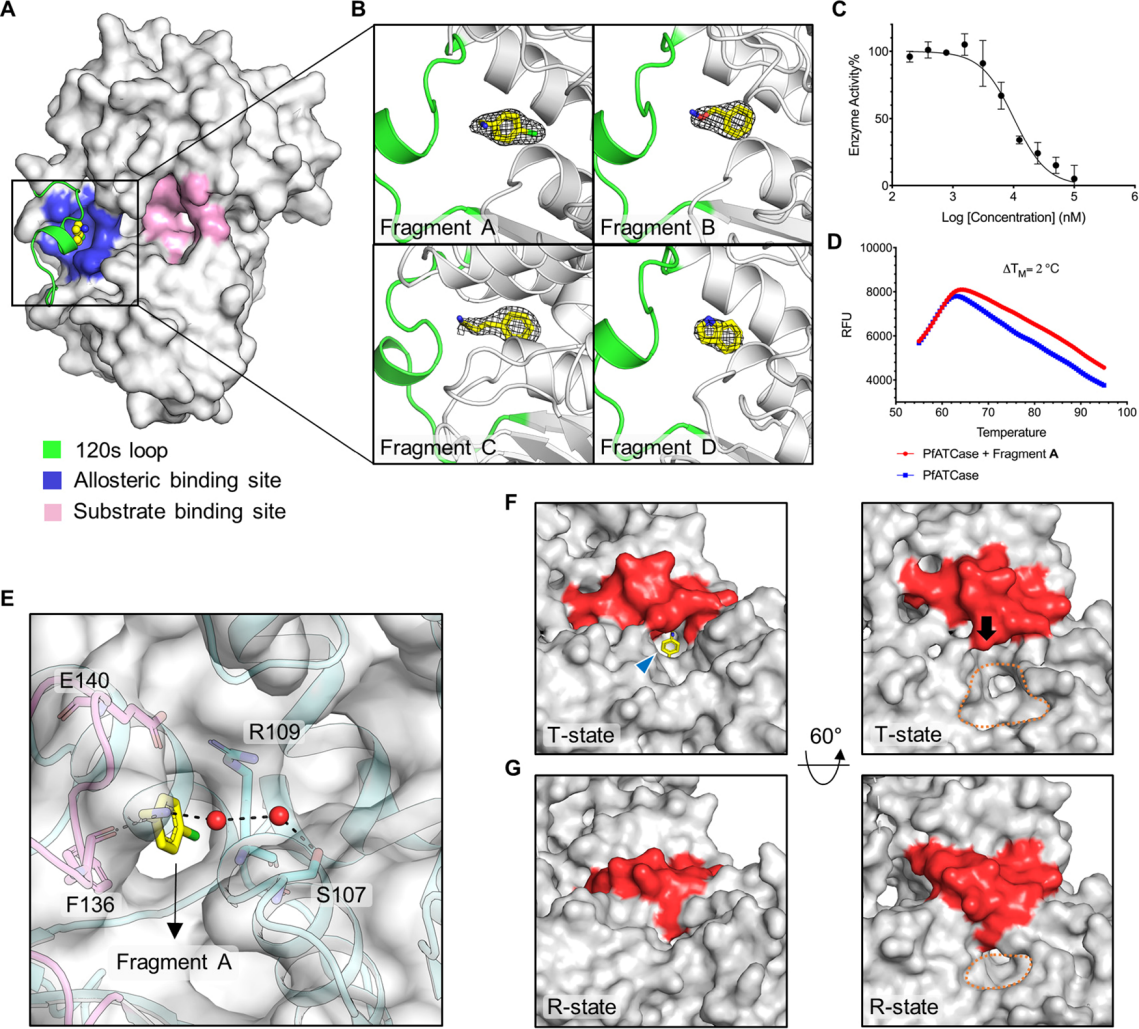
Fragments bind directly to an allosteric pocket and inhibit *Pf*ATC activity. (A) Surface representation of the inactive *Pf*ATC monomer (white; PDB ID: 7ZCZ) with the 120 s loop (residues 128–142) from the adjacent monomer highlighted in green showing the newly identified allosteric pocket (blue) where Fragment A (yellow spheres) is bound. The substrate binding site is shown in light pink. (B) Ribbon diagram showing the 2Fo–Fc electron density map of Fragments A–D contoured at 1σ. (C) In vitro enzyme assay of Fragment A against *Pf*ATC (50 nM, *n* = 3). (D) Differential scanning fluorimetry (DSF) results showing *Pf*ATC (blue) and *Pf*ATC in the presence of Fragment A (red). (E) Key interactions between the allosteric binding site of *Pf*ATC and Fragment A (as shown in (B)), and the surface of *Pf*ATC is shown in gray. (F) Surface representation of the Fragment A:*Pf*ATC complex, showing the surface proximal to the active site. The 120 s loop is highlighted in red and the active site indicated by orange dashed lines. The position of the allosteric pocket is indicated by a blue arrow. Fragment A is shown in yellow sticks. (G) Surface view of the citrate:*Pf*ATC complex near the active site, showing that upon binding of citrate, the 120 s loop shifts toward the active site, covering the allosteric pocket and forcing the substrate domain toward each other. Figures were produced with PyMOL (www.pymol.org).

Initially, we performed a 140-fragment structure-based screening experiment using a subset of an in-house multicomponent reaction (MCR)-compatible library^13^ and the availability of high-resolution crystals.^14^ As the smaller fragments used in our search will typically bind with lower affinities, resulting in weak electron density, pan dataset density analysis (*PanDDa*)^15,16^ was used to analyze the results of this fragment screening experiment. The resulting crystal structures were deposited in the Protein Data Bank (PDB)^17^ under accession codes 7ZCZ (Fragment A liganded *Pf*ATC), 7ZEA (Fragment B liganded *Pf*ATC), 7ZGS (Fragment C liganded *Pf*ATC), and 7ZHI (Fragment D liganded *Pf*ATC). Superposition with our previously deposited citrate-liganded *Pf*ATC structure (a model of the substrate bound form of *Pf*ATC; PDB ID: 5ILN) mapped the fragment binding sites on *Pf*ATC to a new pocket. Fragments A, B, C, and D (Supporting Information Figure 3A) bind in a buried hydrophobic cavity formed by the α3 helix, α4 helix, and β1–3 sheets of the adjacent subunit (Figure 1A,B). Enzymatic assays indicated an IC50 for these compounds of 10, 125, 150, and 145 μM, respectively (Figure 1C, Supporting Information Figure 3B). DSF was performed against these fragments. Fragment A increased the thermal stability of *Pf*ATC by 2.0 °C (Figure 1D), with similar results for Fragment B (Supporting Information Figure 3C). Comparison of Fragment A-D:*Pf*ATC complexes with the citrate:*Pf*ATC complex and apo-structure of *Pf*ATC (PDB ID: 5ILQ) suggested an allosteric mode of inhibition, as Fragments A–D bind in a cavity which is near the traditional substrate binding site (Figure 1A). These results allowed us to identify an allosteric pocket that we had hypothesized existed, based on the discovery of a distinct allosteric pocket on the human homolog.^18^

### Fragments Inhibit *Pf*ATC by Stabilizing the Inactive State

To fully understand the conformational changes driving *Pf*ATC function, our previously released unliganded-*Pf*ATC and *Pf*ATC:citrate (Supporting Information Figure 4A) complex structures were used to model the conformation of the T- (low substrate affinity and low activity) and R-state (high substrate affinity and high activity) enzyme active site, respectively. We also defined a functional 120 s loop (residues 128–142). As shown in Supporting information Figure 4A, the binding site of *Pf*ATC is composed of the Asp domain and CP domain. When both substrates are present in the binding site, a conformational change in *Pf*ATC is induced—converting the active site from the T to the R state. Conformational changes, including the motion of the 120 s loop, induce structural alterations in the position of the substrate binding domains relative to each other. During this conformational change, the Asp and CP domains close by 15.8°, while the distance closes by 2.3 Å (Supporting Information Figure 4B,C).

In the citrate-*Pf*ATC complex, both Ser135 and Lys138 from 120 s loop form polar contacts with phosphate (which represents the product of *Pf*ATC) to support the 120 s loop in the closed position (Supporting Information Figure 5A), whereas in the Fragment A:*Pf*ATC complex, Ser135 and Lys138 adopt an open conformation, forming a hydrophobic region buried under the 120 s loop that accommodates Fragment A (Supporting Information Figure 5B). The 120 s loop of the citrate-bound *Pf*ATC showed a significant shift compared to the Fragment A-bound structure between the α-carbons of Ser135 in the two structures (7.8 Å; Supporting Information Figure 5C). Further analysis revealed that the amino group of Fragment A forms polar contact with Phe136 and a water-mediated bridge with the main chain of Ser107 (Figure 1E, Supporting Information Figure 5D). The main aromatic ring faces the Arg109-Glu140 pair, with which the fragment forms a cation−π interaction.^19^ Fragment A blocks the motion of the 120 s loop and holds *Pf*ATC in its T-state—preventing the movement of the Asp domain and CP domain toward each other to form the carbamoyl aspartate and phosphate (Figure 1F,G). Structural alignment of Fragment A:*Pf*ATC and apo-*Pf*ATC structures did not show any significant impact on the structure of *Pf*ATC. However, stabilizing effects of Fragment A were confirmed by DSF experiments.

### Activity Assay-Based Fragment Screening Identifying Compounds 1–5 as Targeting the Allosteric Pocket

**Figure 2:**
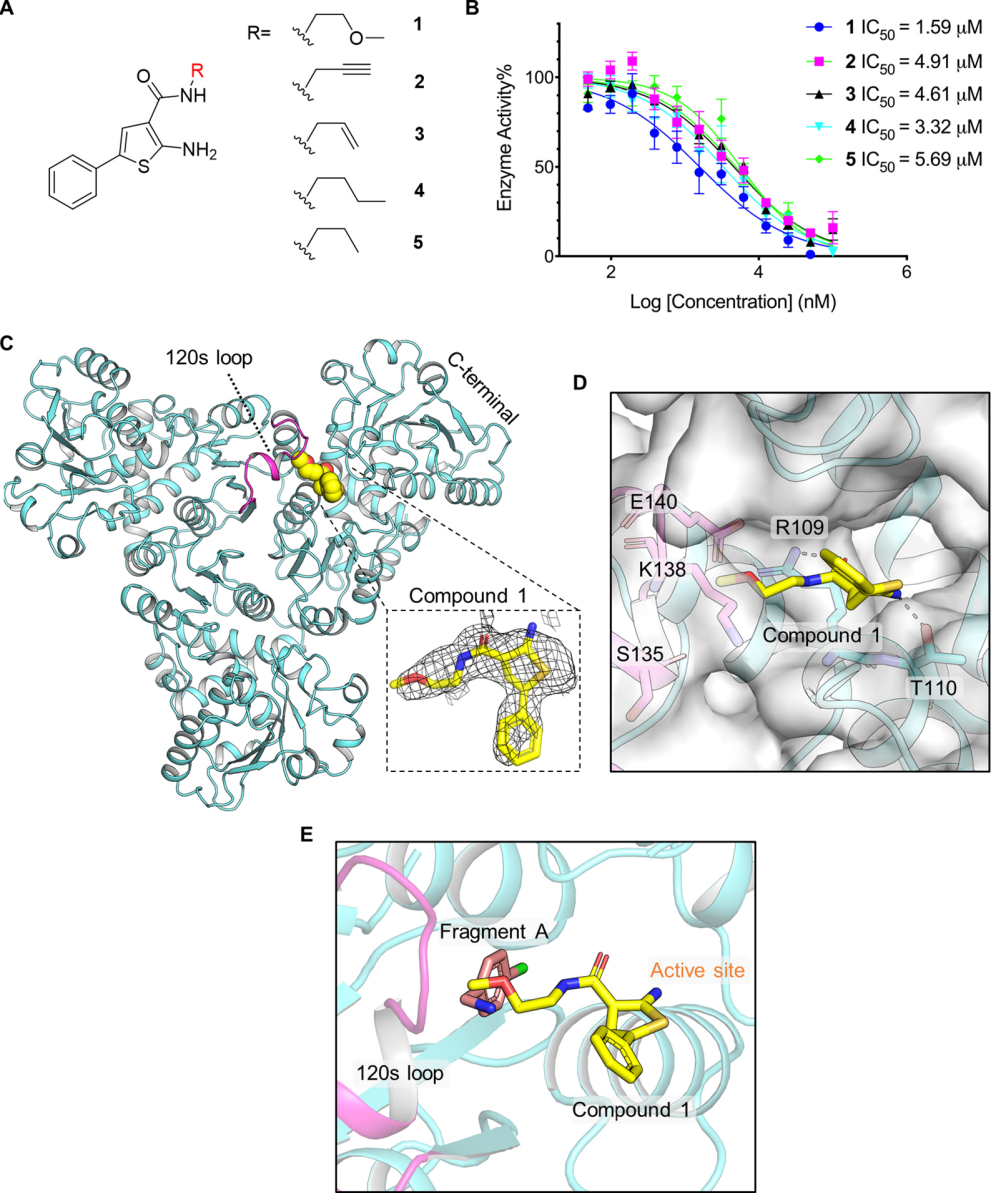
Activity assay-based screening identified compounds 1–5 bind to the allosteric pocket and the channel which links to the substrate binding site. (A) Chemical structure of compounds 1–5. (B) In vitro enzyme assay of compounds 1–5 against *Pf*ATC (50 nM, *n* = 3). (C) Overall structure of the *Pf*ATC in complex with compound 1 (PDB ID: 7ZST), the 120 s loop is highlighted in light magenta, and compound 1 is shown in spheres. The 2Fc–Fo density map of compound 1 is contoured at 1.2 σ. (D) Key interactions between the binding site of *Pf*ATC and compound 1; the surface of *Pf*ATC is shown in gray. (E) Ribbon representation of the Fragment A:*Pf*ATC complex superimposed on the compound 1:PfATC complex. Fragment A and compound 1 bind in a similar allosteric pocket, part of compound 1 extends to the channel which links to the allosteric pocket.

To further confirm the druggability and function of this allosteric pocket, we performed a fragment-based screening of a 1020-member in-house fragment library using an enzymatic assay following the production of carbamoyl-aspartate at 466 nm.^20^ In this assay, we used a cocktail method in which fragments were divided into 85 groups, such that each group contained 12 compounds. Each fragment pool was tested in six concentrations. Once inhibiting compound pools were identified, the individual components of the associated pools were screened individually. Following this approach, five compounds were identified which differ only with respect to the presence of methoxy or acetenyl groups on the acetyl moiety extending from the central phenylthiophene ring. We termed these *Pf*ATC compounds 1–5 (Figure 2A). Activity assays demonstrated that compounds 1–5 showed inhibition of *Pf*ATC, with IC50s of 1.59, 4.91, 4.61, 3.32, and 5.69 μM, respectively (Figure 2B). Crystal soaking experiments were performed to establish the binding mode of compound 1 as an exemplar. The cocrystal structure of compound 1 bound to *Pf*ATC showed that the terminal methoxy side chain of compound 1 extends deep into the allosteric binding site and is flanked by the 120 s loop, forming a polar contact with Arg109 (Figure 2C,D). The amino group of compound 1 forms a polar contact with Thr110. The core 5-phenylthiophene ring extends to a channel linked to the new pocket.

### Generation of BDAs Based on the Cocrystal Structure Identified That BDA-04 Selectively Inhibits *Pf*ATase via Noncompetitive Substrate Inhibition

**Figure 3:**
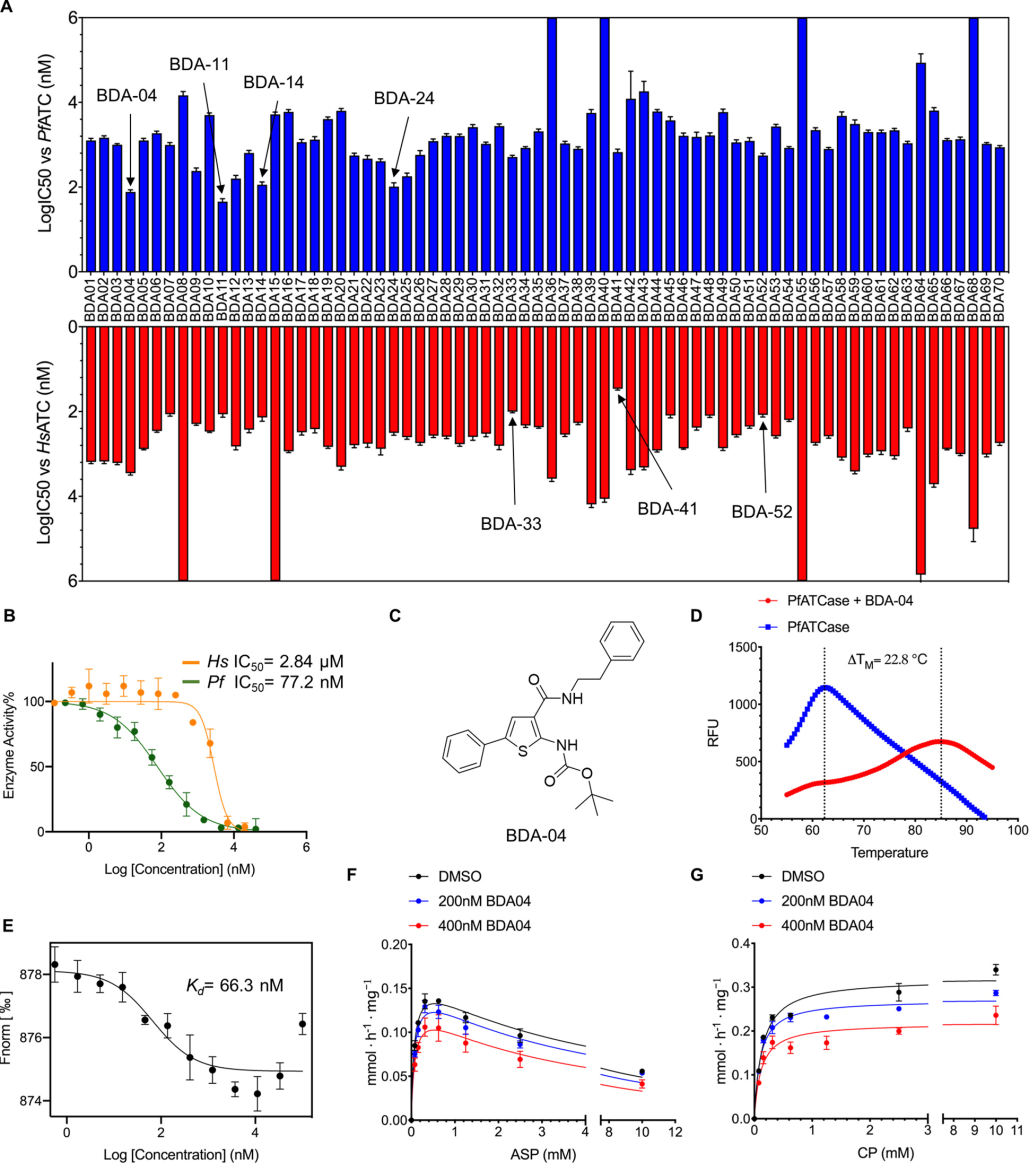
BDA-04 binds *Pf*ATC and inhibits via an allosteric mechanism. (A) A total of 70 compounds were synthesized and tested in activity assays against *P. falciparum* ATC and *Homo sapiens* ATC. The experimentally obtained IC50s against both malarial (blue) and human (red) ATC are shown. (B) Inhibition of ATC of *Pf* (*Plasmodium falciparum*) and *Hs* (*Homo sapiens*) by BDA-04 (*n* = 3). Assays were performed at concentration ranging from 20,000 to 0.1 nM. (C) Chemical structure of BDA-04. (D) DSF results showing the thermal stabilization of *Pf*ATC in presence of BDA-04. The *T*_M_ value of *Pf*ATC increases by over 20° after incubation with BDA-04. (E) MST result showing the binding affinity of *Pf*ATC (50 nM, *n* = 3) with BDA-04. (F,G) Activity assay at fixed concentration of one substrate (2 mM CP (F)) or 1 mM Asp (G) varying the other substrate at different concentrations of BDA-04 (0, 200 and 400 nM). The fit to the Michaels–Menten equation is shown (*n* = 3).

While compound 1 showed relatively modest inhibition against *Pf*ATC, alignment of the cocrystal structures of Fragments A-D with the cocrystal structure of compound 1 (Figure 2E) prompted us to merge both molecules, using compound 1 as the core structure, and replacing the methoxy group with different aromatic rings. We also modified the amino group and generated a new scaffold, suspecting that varying the R1, R2, and R3 groups would generate a series of compounds with optimized binding and improved inhibition (BDAs, Supporting Information Table 1). We evaluated the BDA series in an in vitro activity assay against malarial and human ATC (Figure 3A, Supporting Information Table 1). BDA-04 shows strong selectivity, with measured IC50s of 77.2 nM and 2.8 μM against *Pf*ATC and *Hs*ATC, respectively (Figure 3B,C). We then performed DSF experiments against BDA-04, which showed that BDA-04 raised the *T*_M_ value of *Pf*ATC by approximately 23° (Figure 3D). Similar results were seen for BDA-11, BDA-14, and BDA-24 (Supporting Information Figure 6). To further characterize BDA-04, we then performed microscale thermophoresis (MST) that determined the *K*_d_ of BDA-04 as 66.3 nM (Figure 3E). Significant deviations from the sigmoidal distribution of the MST curve are present at concentrations higher than 10 μM, which we have interpreted as indicative of BDA-04 insolubility at concentration in excess of 10 μM. To characterize the mechanism of inhibition by BDAs, enzyme activity assays were performed using BDA-04 as an exemplar (Figure 3F,G). This analysis indicates that BDA-04 acts as a noncompetitive inhibitor with CP and Asp. To our knowledge, it represents the first demonstration of a noncompetitive ATC inhibitor.

### BDA-04 Binds to a Novel Allosteric Pocket and Stabilizes the Inactive State of *Pf*ATC

**Figure 4:**
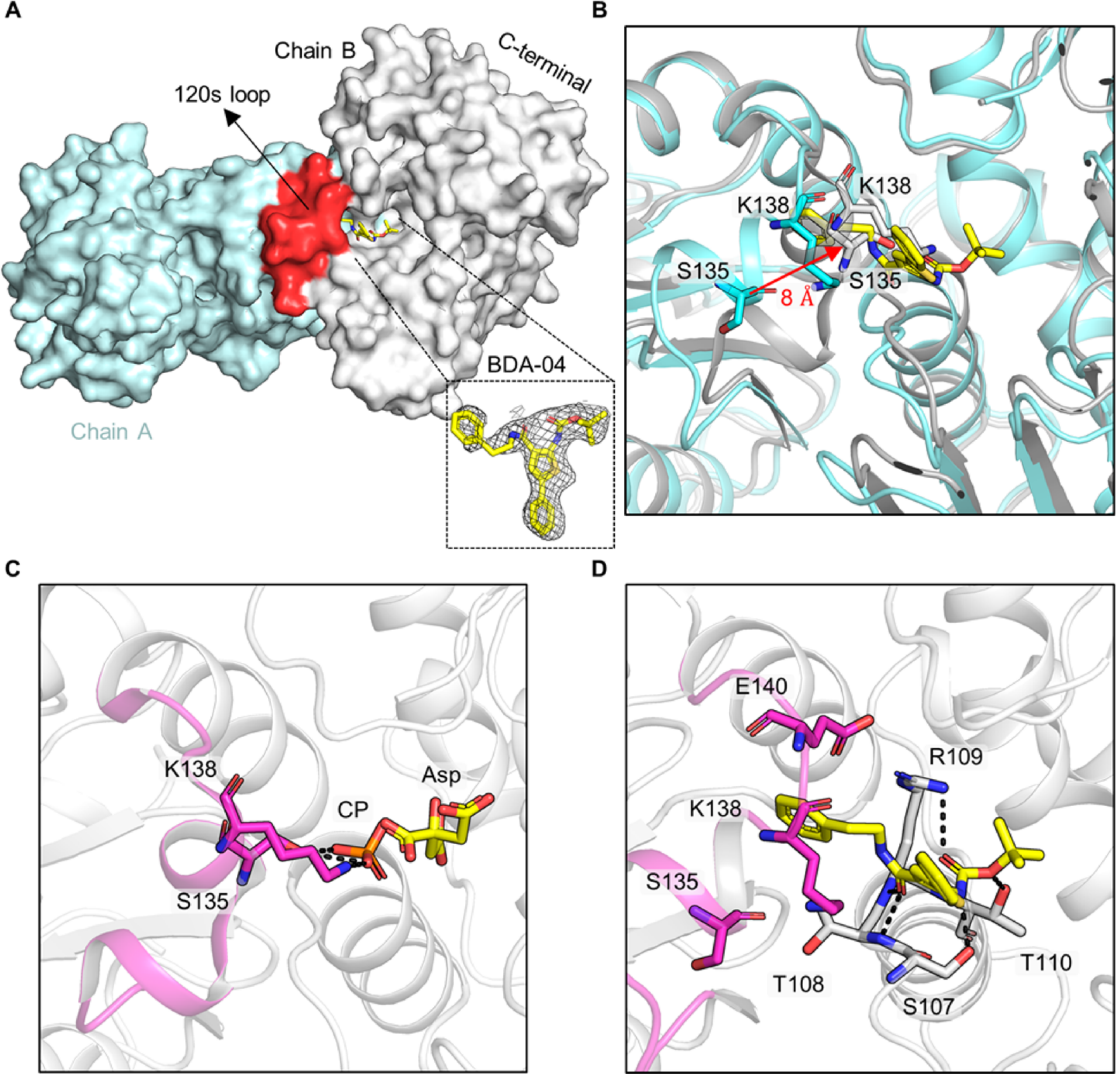
BDAs bind at the allosteric pocket of *Pf*ATC. (A) Structure of *Pf*ATC in complex with BDA-04 (PDB ID: 7ZP2). Two monomers of the trimer are shown. The 2Fc–Fo density map of BDA-04 is contoured at 1.0 σ. (B) Superimposition of the BDA-04:*Pf*ATC complex (cyan) with citrate-bound *Pf*ATC (gray). (C) Crystal structure of the citrate:*Pf*ATC complex, representing the R-state of *Pf*ATC, showing the important interactions of the 120 s loop (highlighted in light magenta) with phosphate. Citrate and phosphate are shown in sticks. (D) Stick representation showing the key interactions between *Pf*ATC and BDA-04.

Compound BDA-04 was cocrystallized with *Pf*ATC, and the complex structure was solved at a resolution of 2.1 Å (PDB ID: 7ZP2). The inhibitor is well defined in the electron density. The structure demonstrated BDA-04 bound to the allosteric pocket shielded from the solvent by the 120 s loop and extending into the channel to the active site (Figure 4A). The terminal benzene side chain of BDA-04 is deeply buried in this hydrophobic area formed by the α3 helix, α4 helix, and β1–3 sheets from an adjacent subunit. The 5-phenylthiophene ring extends from the hydrophobic binding site to the “gate” of the pocket, blocking access to the allosteric pocket. The Boc moiety of BDA-04 partially occludes the CP binding domain. Superimposition of the BDA-04:*Pf*ATC complex with citrate-bound *Pf*ATC reveals that the 120 s loop moves by approximately 8 Å (measured between the α-carbons of Tyr134 in the two structures (Figure 4B)). The side chains of Ser135 and Lys138 from the 120 s loop, which recognize the CP, are located directly in the CP binding site and form polar contacts with phosphate (Figure 4C). However, in the BDA-04:*Pf*ATC complex, Ser135 and Lys138 shift out of the CP binding site as the 120 s loop is blocked by BDA-04 (Figure 4B,D). The structure of the BDA-04:*Pf*ATC complex superimposes well with the apo *Pf*ATC structure and thermal shift assay showed BDA-04 raised the *T*_M_ value of *Pf*ATC and reduces the B-factor of the 120 s loop, strongly suggesting that BDA-04 maintains *Pf*ATC in its inactive state. BDA-04 therefore inhibits *Pf*ATCase allosterically, by stabilizing *Pf*ATC in inactive state that indirectly blocks the binding and recognition of CP.

Binding of BDA-04 completely blocked the movement of the 120 s loop, with the amino groups of the gatekeepers Ser135 and Lys138 moving by 8.5 and 7.1 Å, compared to their active position, respectively. The benzene side chain moiety of BDA-04 forms a cation−π interaction with the Arg109-Glu140 pair and is surrounded by hydrophobic elements of Arg109, Glu140, and Tyr137 (Figure 4D). The carbonyl group from the benzene group side chain moiety of BDA-04 is in close contact with the backbone of Arg109 and forms a polar interaction with the main chain of Arg109. The 5-phenylthiophene moiety of BDA-04 forms a salt bridge with Arg295. The Boc moiety of BDA-04 forms polar contacts with the side chains of Arg109 and main chain of Ser107. The amino group of the Boc side chain moiety of BDA-04 forms a hydrogen bond with the side chain of Ser107.

We also cocrystallized a close analogue of compound 1 and BDA-04: BDA-14 (Supporting Information Figure 7), which possesses a hydroxyl group instead of the benzene ring. The hydroxy group of BDA-14 did not alter the overall binding mode of the molecule. While a hydroxy group chain forms an additional hydrogen binding interaction with Thr110, this additional interaction is not reflected in improved in vitro results.

### Cellular Activity and Selectivity of BDAs

To evaluate the cellular effect of BDAs on *P. falciparum* 3D7, we performed experiments using the most potent compounds identified from the in vitro activity assay. These experiments demonstrated that the EC50 values of BDA-04, BDA-11, BDA-14, and BDA-24 against blood stage 3D7 cultures were 2.4, 3.4, 42.5, and 2.0 μM, respectively. We also performed an initial cytotoxicity study of the same compounds against cultured human lymphocytes and found that BDA-04 and BDA-14 showed no cytotoxicity at 100 μM (Supporting Information Table 2 and Figure 8). Additionally, no cytotoxic effect on cultured human HepG2 cells was observed for BDA-04 at a concentration of 100 μM.

## Conclusions and Discussion

This manuscript details the discovery of a class of allosteric inhibitors of the malarial aspartate transcarbamoylase, termed the BDA series. Experiments performed indicate that these compounds are high-potency inhibitors of enzymatic function in vitro and are selective between the human and parasite homologs. The best performing compounds of this series (BDA-04, BDA-11, BDA-14, and BDA-24) display IC50s of 77.2, 45.7, 114.3, and 102.7 nM and 2839, 115.9, 137.2, and 316.3 nM against *Pf*ATC and *Hs*ATC in an in vitro assay, respectively. The binding site and mode for these compounds have been determined by X-ray crystallography, indicating that their mode of action is to stabilize the enzyme in its low substrate affinity “T” state, thereby providing allosteric inhibition that allows for the observed selectivity. This combination of structural biology and in vitro assays arrived at compounds that show both high inhibition in the in vitro activity assay and tight target binding, with *K*_d_ values for BDA-04 determined as 66.3 nM. These compounds were then tested against blood stage malarial cultures and human lymphocytes. These experiments showed that BDA-04, BDA-11, BDA-14, and BDA-24 inhibited plasmodial proliferation in red blood cells with EC50s of 2.43, 3.37, 42.5, and 2.03 μM, respectively, clearly showing the potential for this class of compounds in the development of antimalarials. This is in contrast to the performance of PALA as the best ATC inhibitor, which showed no inhibitory effects in ex vivo experiments.^12^ The initial cytotoxicity experiments indicate that significant selectivity exists between the impact of these compounds on malarial and human cell cultures. For example, BDA-04, which had a measured EC50 in parasite proliferation assays of 2.43 μM, demonstrated EC50s of ∼1000 μM against normal lymphocytes. These results indicate that the BDA series represents a strong lead series for the development of novel antimalarials. While these compounds inhibit blood stage proliferation of the malarial parasite, we have no data on the effect on these compounds on other stages of the malarial life cycle. An interesting synthetic-chemistry feature in the fast and efficient lead optimization of the thiophene molecules BDA series is the multicomponent reaction nature involving a Gewald MCR.^21,22^ Using this reaction, complex molecules could be built up in a few steps with many possible variations.

These data provide further support for the development of inhibitors of this enzyme—and the pyrimidine biosynthesis pathway—as attractive targets for drug discovery. We have analyzed the potential for other binding sites on the human and malarial ATC enzymes using FTMap.^23,24^ This computational analysis suggests no binding to the human homolog allosteric sites and allosteric pockets of *Pf*ATC as a potential binding site (Supporting Information Figure 10). However, we cannot currently exclude the potential for off-target effects of the BDA series, and further experiments to confirm the target(s) of these molecules within the parasite are required. These experiments and further development of the BDA-series to improve potency are in progress. However, this series of molecules carries additional value as tool compounds to assess the druggability of pyrimidine biosynthesis in other disease-causing parasites.

## Supporting Information Available

Experimental details and analytical and characterization data, including supporting information Figures 1–10 and Tables 1–5 (PDF)

## Notes

The authors have submitted a patent application on the molecules described in this manuscript (No. 21211903.6).
