# Supplementary material for: Discovery of Small-Molecule Allosteric Inhibitors of PfATC as Antimalarials
Source: J Am Chem Soc. 2022 Oct 4;144(41):19070–7. doi: 10.1021/jacs.2c08128 (PMC9585585; doi:10.1021/jacs.2c08128)
Supplement: Supplementary file 1 — ja2c08128_si_001.pdf [file ja2c08128_si_001.pdf]

Supporting information for

Discovery of Small Molecule Allosteric Inhibitors of *Pf*ATC as Antimalarials

Chao Wang,[&,†] Bidong Zhang,[&,†], Arne Krüger,[‡] Xiaochen Du,[†] Lidia Visser,[§] Alexander S S Dömling,[†] Carsten Wrenger,[‡] and Matthew R Groves*[,†]

[†]XB20 Department of Drug Design, Groningen Research Institute of Pharmacy, University of Groningen, Antonius Deusinglaan 1, 9700 AD, Groningen, The Netherlands

[‡]Unit for Drug Discovery, Department of Parasitology, Institute of Biomedical Sciences, University of São Paulo, Avenida Professor Lineu Prestes 1374, 05508-000 São Paulo-SP, Brazil

[§]Department of Pathology and Medical Biology, University of Groningen, University Medical Center, 9700 RB, Groningen. The Netherlands

This PDF file includes:

Materials and Methods
Synthetic Procedures
^{1}H NMR and ^{13}C NMR Spectra
Supporting information Figures 1 to 10
Supporting information Tables 1 to 5
References

METHODS

***Pf*ATC cloning, expression and purification of ATCs.** Wild type *Pf*ATC-Met3 was cloned, expressed and purified to homogeneity according to Lunev et al [14].

***Hs*ATC cloning, expression and purification of ATCs.** The full-length human CAD gene (carbamoyl-phosphate synthetase 2, aspartate transcarbamylase, and dihydroorotase [Homo sapiens (human)] Gene ID: 790) was purchased from Eurofins and was amplified by PCR using Phusion High-Fidelity PCR Master Mix (New England Biolabs), using the forward primer (*hs*ATC_fwd; 5'-agggcgccATGCTGCACTCATTAGTGG-3') and reverse primer (*hs*ATC_rev; 5'-cgaattcgCTAGAAACGGCCCAGCAC-3'). The pETM-41 vector was obtained from European Molecular Biology Laboratory (EMBL) and the PCR reaction was performed using Phusion High-Fidelity PCR Master Mix(New England Biolabs), the forward primer sequence (pETM-41_fwd 5'-ccgtttctagCGAATTCGAGCTCCGTCG-3') and the reverse primer sequence (pETM-41_rev 5'-gcagcatGGCGCCCTGAAAATAAAG-3'). The final expression plasmid pETM-41-hsATC-full was obtained by Gibson Assembly reaction using E2611 Gibson Assembly Master Mix purchasing from New England Biolabs, and it encoded the *hu*ATC with N-terminal His_6-tagged maltose-binding protein.

His-tagged maltose-binding protein *Hs*ATC was recombinantly expressed using *E. coli* BL21 star competent cells transformed with a plasmid containing an MBP and His_6 tagged human ATC construct (pETM-41-hsATC, Supplementary Table 2). The culture was propagated in 1L of selective TB media supplemented with 50 µg ml^{-1} kanamycin, 35 µg ml^{-1} chloramphenicol at 310 K, followed by induction with 0.1 M of IPTG for 18h at 291 K. Bacterial cells were harvested by centrifugation and resuspended in 35 ml lysis buffer [20 mM Tris-HCl pH 8.0, 0.5 M NaCl, 5% (v/v) glycerol, 2 mM β-mercaptoethanol (BME)]. Cell lysis was performed by sonication on ice.

The lysate was clarified by centrifugation at 45,000g (SS-34 rotor, Thermo Scientific) for 50 min. The supernatant was filtered using 0.45 µm filter membrane (Whatman) and applied onto a 5 ml Ni^{2+} HisTrap HP column (GE Healthcare, USA). Following washing with Lysis buffer supplemented with 40 mM imidazole, the *Hs*ATC protein was eluted by increasing the concentration of imidazole to 300 mM. Excess imidazole was removed and the His-tag was cleaved off by overnight dialysis against dialysis buffer [20 mM Tris-HCl pH 7.0, 75 mM NaCl, 5% (v/v) glycerol, 2 mM β-mercaptoethanol (BME)], with inclusion of TEV protease within dialysis bag. Then, the sample was loaded onto a 5 ml HiTrap SP HP column (GE Healthcare, USA) and equilibrated in dialysis buffer. The elate was pooled and concentrated at 277 K to 2 mg ml^{-1} using Vivaspin Turbo 4 concentration column with a 10 kDa cutoff (Sartorius Stedim Biotech, Germany).

The concentrated sample was further purified by size-exclusion chromatography (SEC), the protein was concentrated to a volume of 1 ml and purified via SEC suing a HiLoad 16/60 Superdex 75 column (GE Healthcare) pre-equilibrated with SEC buffer [20 mM Tris-HCl pH 7.0, 100 mM NaCl, 2% (v/v) glycerol, 0.2 mM tris(2-carboxyethyl)phosphine(TCEP)], using NGC liquid chromatography system (BioRad). The purified protein as a single peak and was pooled and concentrated to 2 mg ml^{-1} at 277 K. the final concentration was determined based on the protein theoretical absorbance at 280 mm [ABS 0.1% (1mg ml^{-1}) = 0.354].

***Pf*ATC activity assay.** Enzymatic reactions were performed in a total volume of 150 µL in 50 mM Tris-Acetate buffer at pH 8.0 and the final concentration of *Pf*ATC is 50 nM. L-Aspartate (Asp) and carbamoyl-phosphate (CP) saturation curves of the enzymes were assayed using a fixed concentration of CP (2 mM) and L-aspartate (1mM). Small-molecule dose-response curves were measured using assay buffer supplemented with 2% (v/v) DMSO, 2 mM CP and 1mM aspartate. *Pf*ATC was pre-incubated with Asp and compounds for 10 min by putting the plate in a shaker at room temperature. The reactions were initiated by adding CP and quenched after 10 min with

100µL of stop mix (two volumes of Antipyrine (26.5 mM 2,3-Dimethyl-1-phenyl-3-pyrazolin-5-one in 50% (v/v) sulfuric acid) and one volume of 2,3-Butanedione monoxime (80 mM 2,3-Butanedione monoxime in 5% (v/v) acetic acid). After plates were sealed with transparent sealing tape to prevent evaporation and incubated overnight in the dark place at room temperature. After incubation, the plates were heated at 95ºC for 15 min in dark place, and kept for 30 min before measuring at 466 nm suing a Synergy H1 Hybrid Reader (BioTek). Analyses were performed using Microsoft Excel and Graph Pad Prism.

***Hs*ATC activity assay.** The enzymatic assay was performed as described herein above for enzymatic assay of *Pf*ATC with minor modifications. Briefly, the reaction was carried out at room temperature in a total volume of 150 µl of 10 mM L-aspartate and 5 mM carbamoyl phosphate saturated substrate solution in 50 mM Tris-Acetate buffer pH 8.3. *hs*ATC was pre-incubated with inhibitor and L-Asp for 10 min at room temperature on a shaker. The reaction was initiated by adding CP and stopped after 5 min with 10 ml color mix. Then the plate was covered by sealing tape and kept in dark place at room temperature and then heated to 95ºC for 15min in dark and kept the plate in the dark for another 30 min before measuring the absorbance at 466 nm suing a Synergy H1 Hybrid Reader (BioTek). Analyses were performed using Microsoft Excel and Graph Pad Prism.

Human cell line experiments. The cell lines A375 (melanoma), H1299 (lungca), MCF7 (breastca), REC-1 (mantel cell lymphoma, SUDHL2 (diffuse large B-cell lymphoma) and normal lymphocytes (obtained from tonsils) were cultured in RPMI 1640 (Lonza BioWhittaker, Walkersville, MD, USA) with 10% fetal bovine serum (FBS; HyClone Thermo Scientific, Waltham, MA, USA). Cells were plated in 96-wells plates and incubated with increasing concentrations of compounds for 72 hours as indicated. All experiments were performed in

triplicate. AlamarBlue (Thermo Fisher Scientific, Waltham, MA USA) was added eight hours prior to read-out (extinction 560nm, emission 590nm).

Crystallization of *Pf*ATC in complex with Fragment A/B/C/D. A freshly prepared protein solution at a concentration of 10 mg ml^{-1} and immediately used in crystallization trials. The screening for crystallization conditions for *Pf*ATC was performed using a high-through crystallization robot (Gryphon, Art Robbins) against commercially available sparse-matrix screening kits (JCSG *plus* and PACT *premier*; Molecular Dimensions Ltd.). All experiments were performed at 293 K using the sitting drop vapor diffusion technique in 96 well MRC2 plates (Molecular Dimensions Ltd.). Equal volumes (0.2 µL) of protein solution and crystallization reagent were equilibrated against 50 µL of reservoir solution. Medium size single crystals appeared overnight in various conditions containing PEG3350/4000. The apo-crystals were soaked for 10 min using crystallization-liquor supplemented with 10 mM fragment A/B/C/D and transferred to the solution plus 20% glycerol, flash-cooled and stored in liquid nitrogen. The flashed cooled crystals was shipped to Petra III, Deutsches Elektronen-Synchrotron DESY Beamline P11. Crystallization conditions parameters are summarized in **Table S4.**

Crystallization of *Pf*ATC in complex with Compound 1/BDA-04/BDA-14. The purified *Pf*ATC protein at a concentration of 10 mg ml^{-1} was incubate with compound 1/BDA-04/BDA-14 (10 mM stocks in 100% DMSO) at a molar ratio of 1:5. The mixture was incubated at 20 ℃ for 40 minutes, then centrifugation was performed to remove the precipitate. The supernatant was immediately used in crystallization trials. The crystallization reagent were the same as apo crystallization condition plus 5% DMSO (v/v) compound 1/BDA-04/BDA-14 (1 mM). The crystals were flash-cooled and stored in liquid nitrogen, and was shipped to Petra III, Deutsches Elektronen-Synchrotron DESY Beamline P11. Crystallization conditions parameters are summarized in **Table S4.**

Data collection, processing and refinement. All the diffraction data sets were collected using synchrotron radiation wavelength 1.033200 Å at DESY beamline P11. The data collected for *Pf*ATC crystals grown in presence of fragments and compounds were processed using XDSAPP [25] and Aimless [26], and the structures were solved and initially refined using the DIMPLE pipeline within CCP4 suite [26] with coordinates of the unliganded *Pf*ATC (5ILQ) as a starting model. The final refinement included manual rebuilding in coot [27] and Refmac5 [28]. The resulting crystal structures were deposited in PDB [29]. Data collection and processing statistics are show in **Table S5**.

Differential Scanning Fluorimetry (DSF). The purified *Pf*ATC was incubate with 5000 × Sypro Orange (Invitrogen) and dilution it to 1:400 stock of dye in assay buffer (50 mM Tris-HCl pH =8.0 and 300 mM NaCl). Each reaction consisted of 2 µL compound (10 mM stock in 100% DMSO), 5 µL *Pf*ATC-Sypro orange mixture and 43 µL assay buffer. Final protein assay concentration was 5µM, final DMSO concentration war 2% (v/v). After incubation for 20 min at 20 ℃, the samples were hated from 25℃ to 95℃ at a rate of 0.5 ℃ min^{-1}. The inflection points of the melting curves were determined using BioRad CSV 96 control software and processed & plotted by GraphPad.

Microscale Thermophoresis (MST). MST measurements were performed on a Nanotemper Monolith NT.115 instrument (Nanotempter Technologies GmbH). Purified *Pf*ATC was labeled using the RED-NHS Monolith Protein Labelling Kit according to manufacturer's protocol. The MST measurements were performed in MST buffer (50 mM Tris-base pH= 8.0, 300 mM NaCl) supplemented with 0.05% (v/v) Tween 20 in standard capillaries (Nanotemper Technologies GmbH). Labeled *Pf*ATC was used at a final concentration of 20nM. The compounds were titrated in a 1:1 dilution following the manufacturer's protocol. All binding reaction were incubated for 10 min followed by centrifugation at 20000g prior to loading. All measurements were performed in triplicate at 20% LED and 40% MST power.

Cell culture and growth-inhibition assay of *P. falciparum*. *P. falciparum* 3D7 parasites (Wellcome Trust Dundee) were maintained in continuous culture at 37 °C and an atmosphere consisting of 90% N_2, 5% O_2, and 5% CO_2 as described previously [30] with modifications.[31] Parasites were maintained in 25 mM 4-(2-hydroxyethyl)-1-piperazineethanesulfonic acid (HEPES) and 11.9 mM sodium bicarbonate buffered RPMI 1640 medium supplemented with D-glucose (11 mM), hypoxanthine, AlbuMAX-I (0.5% w/v), and 10 µg/mL gentamicin. 0+ blood was provided by "Hospital Novo Atibaia" (Brazil) in agreement with the ethics committee at ICB-USP. Compounds were tested for their inhibitory effect against *P. falciparum* 3D7 conducting SYBR Green I (Invitrogen) drug assays as reported.[32, 33] Briefly, two-fold serial dilutions of compounds were prepared in 96-well plates starting from 100 and 20 µM in technical triplicate and incubated for 96 h under normal growth conditions using an initial parasitemia of 0.5% and a hematocrit of 2% in a volume of 100 µL per well. Parasite proliferation was measured by quantifying DNA via SYBR Green I emitted fluorescence in the wells after addition of 100 µL lysis buffer supplemented with SYBR Green I (0.02% v/v) and incubation for 1 h at room temperature in the dark. Fluorescence was quantified using a CLARIOstar plate reader (BMG Labtech, Germany) at excitation and emission wavelength bands of 485 (± 9) and 530 (± 12) nm, respectively. Focal and gain adjustment was performed using the negative controls (highest expected fluorescence signal). Data was acquired via the CLARIOstar (V5.20) and MARS software, manually normalized, and plotted using the nonlinear regression curve fit implemented in GraphPad Prism as described below in more detail. Non-treated parasites, highest solvent concentration on parasites, and highest drug concentration in medium were used as controls for maximal growth, solvent control, and native drug fluorescence, respectively.

Nonlinear regression fit and analysis of dose-response drug assays. Nonlinear regression as implemented in GraphPad Prism 8.4.3 (log(inhibitor) vs. response – Variable slope (four parameters)) was used to fit the measured data to interpolate the IC_{50} value from the curve. No specific model was applied. Data was pre-processed by min-max-scaling according to the following formula

$$y_{normalized} = \frac{y - y_{minimum}}{y_{maximum} - y_{minimum}}$$

where y is the fluorescence signal in each well, $y_{minimum}$ the background fluorescence, and $y_{maximum}$ the highest measured fluorescence signal in the untreated wells. Drug concentrations (in µM) were transformed to the log(10)of the values. Means of each independent experiment were plotted as individual values, and the SD of the mean from the means shown as error bars. The test for *homoscedasticity* was performed to confirm if no weighting of values was appropriate.

BDA series syntheses

General methods

All chemicals were purchased from commercial suppliers and used without any purification unless otherwise noted. ^{1}H NMR (500 MHz) and ^{13}C NMR (126 MHz) were recorded on a Bruker Avance 500 spectrometer. ^{1}H and ^{13}C NMR spectra were reported in parts per million (ppm) referenced to deuterated solvents, for example, $CDCl_3$: δ = 7.26 ppm (^{1}H) and 77.05 ppm (^{13}C) or DMSO-d_6: δ = 2.50 ppm (^{1}H) and 39.52 ppm (^{13}C). Chemical shifts for ^{1}H NMR were reported as δ values and coupling constants were in hertz (Hz). The following abbreviations were used for spin multiplicity: s = singlet, bs = broad singlet, d = doublet, t = triplet, q = quartet, quin = quintet, dd = double of doublets, ddd = double of doublet of doublets, m = multiplet. Chemical shifts for ^{13}C NMR reported in ppm relative to the solvent peak. Thin layer chromatography was performed on silica gel plates (0.20 mm thick, particle size 25 μm). Flash chromatography was performed using RediSep Rf Normal-phase Silica Flash Columns (Silica Gel 60 Å, 230-400 mesh). High resolution mass spectra were recorded using a LTQOrbitrap- XL (Thermo) at a resolution of 60000@m/z400.

Scheme 1. Syntheses of compounds BDA-01-31, BDA-34, BDA-37, BDA-38, BDA-39, BDA-46, BDA-49, BDA-50, BDA-55, BDA-57, BDA-58, BDA-59, BDA-60, BDA-61, BDA-62, BDA-63, BDA-70.[a]

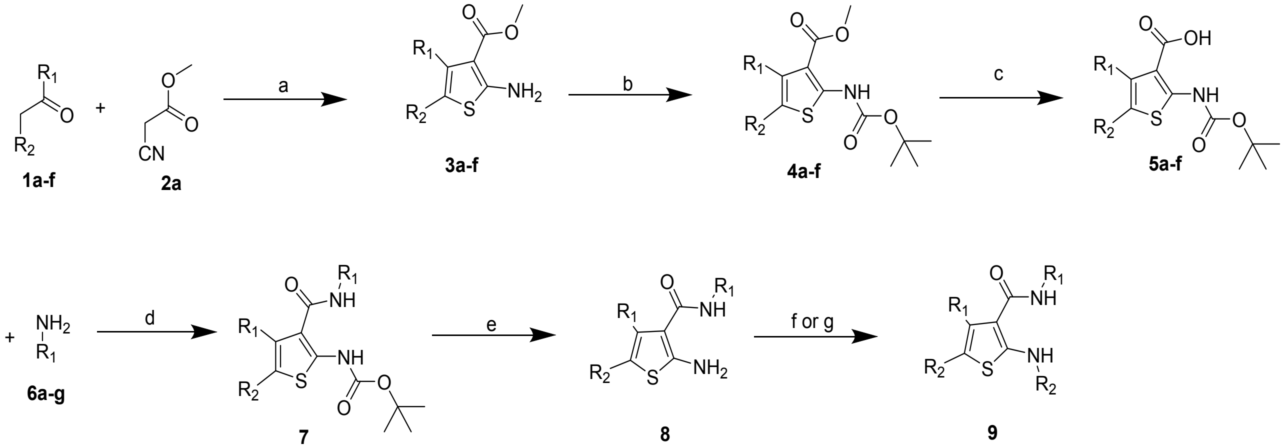

[a]Reagents and conditions: (a) S, Et_3N, EtOH, 50 °C, 16 h; (b) Boc_2O, DMAP, Dioxane, 60 °C, 4 h; (c) NaOH, MeOH : H_2O : THF = 2 : 2 : 1, 80 °C, 6 h; (d) HATU, DCM, rt, 12 h; (e) TFA, DCM, rt, 1 h; (f) $NaBH(OAc)_3$, DCM, AcOH, rt, 18 h; (g) HATU, DCM, rt, 12 h.

General Procedure for Preparing the products Using Method A

As Described in Scheme 1:

Step1:

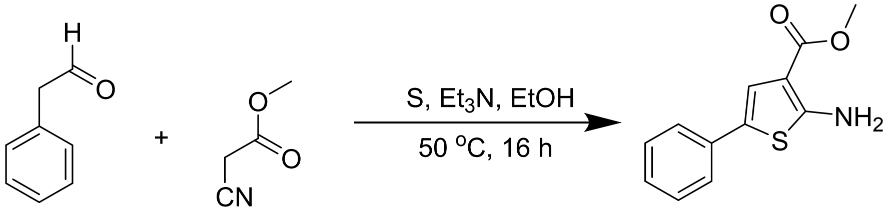

a1 b1 BDA-01

A 100 mL round bottom flask was charged with 2-phenylacetaldehyde (**a1**, 6 mL (6 g), 50 mmol, 1.0 eq), methyl 2-cyanoacetate (**b1**, 5 mL (5.1 g), 55 mmol, 1.1 eq), sulfur (1.6 g, 60 mmol, 1.2 eq), and triethylamine (7 mL (5.05 g), 50 mmol, 1.0 eq) in ethanol (70 mL). The reaction is heated 50 °C in an oil bath for 16 h. Then, the reaction was cooled down to room temperature. A batch of 120 mL ice water was poured into the mixture to yield a precipitate which was filtered and washed with cold ethanol to obtain 8.1 g (68%) of the title compound **BDA-01** as light yellow powder.

Step2:

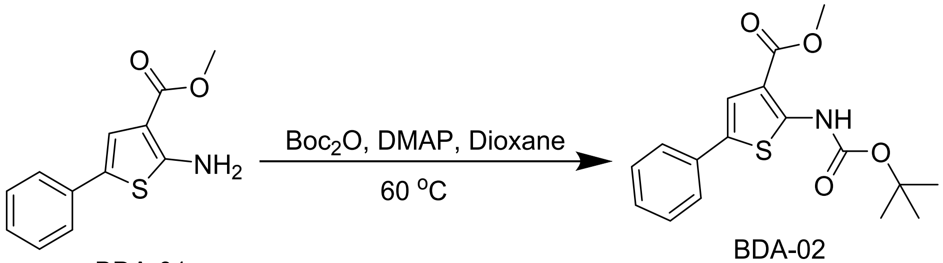

BDA-02

BDA-01

To a 50 mL round bottom flask were added dioxane (30 mL), compound **BDA-01** (1.16 g, 5 mmol, 1.0 eq), Boc_2O (2.18 g, 10 mmol, 2.0 eq) and DMAP (61 mg, 0.5mmol, 0.1 eq) and the reaction mixture was stirred at 60 °C for 4 h. Then $N_2H_4 \cdot H_2O$ (0.75g, 15mmol, 3.0 eq) was added and the mixture was stirred at 40 °C for an additional 1.5 h. After cooling to room temperature the solvent was removed under reduced pressure, and the residue was purified by column chromatography on silica gel (EtOAc–heptane 5 → 50%) to yield the methyl ester of **BDA-02** (1.35 g, 81%).

Step 3:

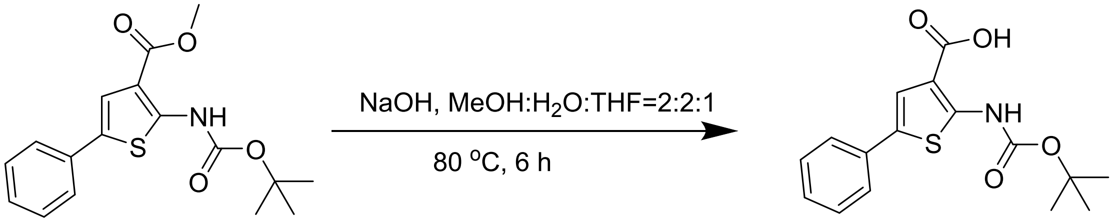

BDA-02 BDA-03

The ester **BDA-02** (1.67 g, 5 mmol, 1.0 eq) was subsequently subjected to a base hydrolysis at 80 °C for 6 h with a solution of NaOH (2.7 g, 13.5 eq) in 100 mL of a solvent mixture (MeOH–H_2O–THF = 2:2:1). The reaction was cooled to room temperature and the organic solvents were removed in vacuo. The aqueous layer was acidified with 5% HCl to give a precipitate, which was triturated with methanol to afford compound **BDA-03** (1.2 g, 70%) as yellow solid.

Step 4:

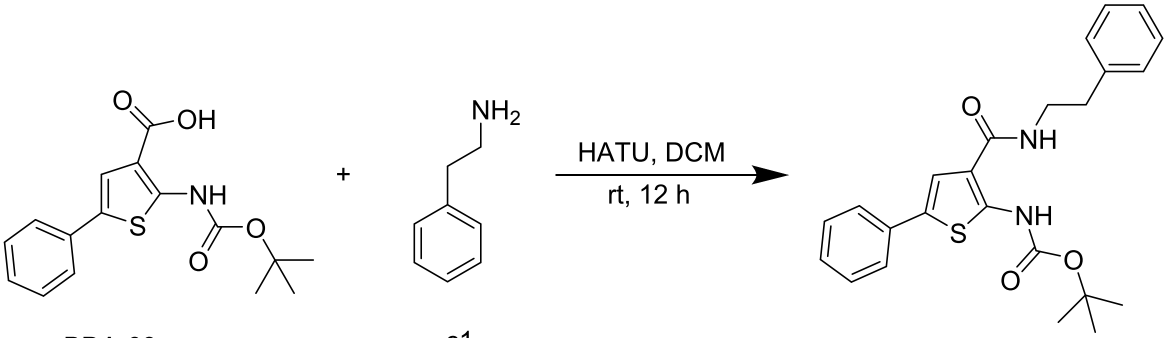

BDA-03 c1 BDA-04

To a solution of compound **BDA-03** (110 mg, 0.34 mmol, 1.0 eq) and DIPEA (180 µL, 1.02 mmol, 3.0 eq) in DCM (10 mL) was added HATU (155 mg, 0.408 mmol, 1.2 eq) and amine **c1** (50 mg, 0.408 mmol, 1.2 eq). The reaction mixture was stirred at room temperature for 12 h. The mixture was washed with water and extracted with EtOAc. The organic layer was separated, dried over $MgSO_4$, filtered and concentrated under reduced pressure. The residue was purified by column chromatography on silica gel using EtOAc–PE (5% → 50%) as an eluent to give **BDA-04** (106 mg, 74%) purple solid.

Step 5:

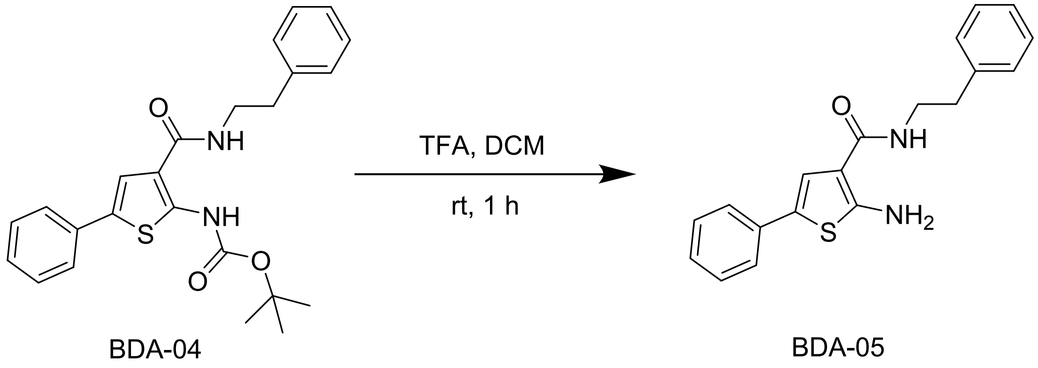

BDA-04 BDA-05

To a solution of compound **BDA-04** (60 mg, 0.142 mmol, 1.0 eq) in DCM (5 mL) was added TFA (2 mL) in DCM (2 mL) at room temperature for 1 h. The solvent was evaporated by high vacuum to give the final product **BDA-05** (45 mg, 98%).

Step 6:

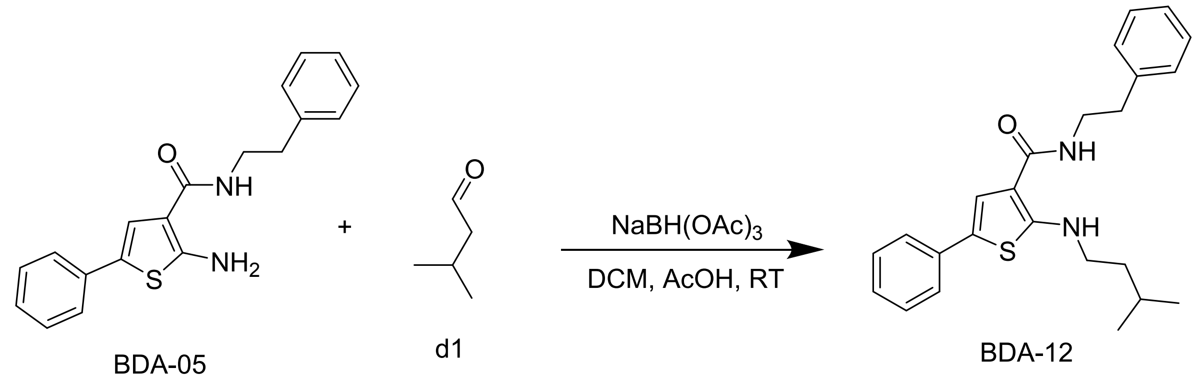

To a solution of **BDA-05** (333 mg, 1.0 mmol, 1.0 eq) in dichloromethane (10 mL) and acetic acid (0.05 mL) were added 3-methylbutanal **d1** (87 mg, 1.0 mmol, 1.0 eq) and sodium triacetoxyborohydride (255 mg, 1.2 mmol, 1.0 eq). The reaction mixture was stirred for 18 h at room temperature and concentrated. The residue was dissolved in ethyl acetate, washed with aqueous sodium bicarbonate solution and with water, dried over anhydrous $MgSO_4$ and concentrated. The residual oil was purified on a silica gel flash chromatography column eluted with EtOAc–PE (5% → 50%) to afford the desire compound **BDA-12** as a slightly yellow oil (294 mg, 75% yield).

Step 7:

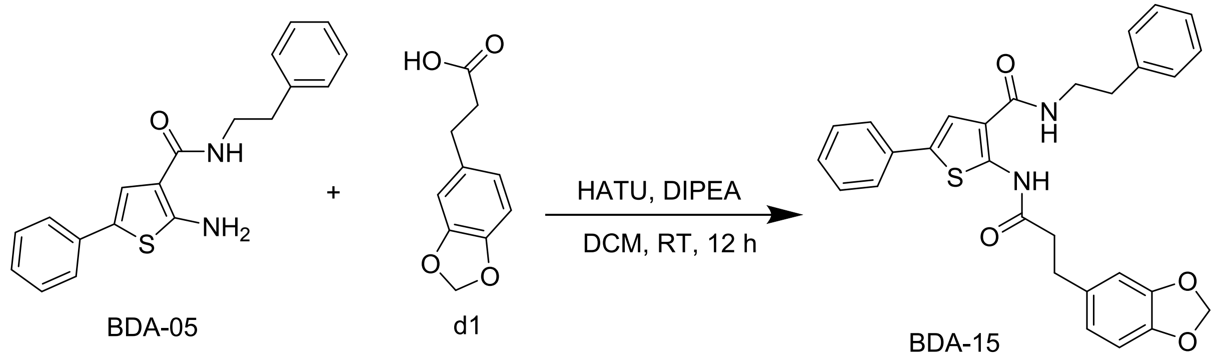

To a solution of compound **BDA-05** (333 mg, 1.0 mmol, 1.0 eq) and DIPEA (540 µL, 3.0 mmol, 3.0 eq) in DCM (10 mL) was added HATU (420 mg, 1.1 mmol, 1.1eq) and acid **d1** (195 mg, 1.0 mmol, 1.0 eq). The reaction mixture was stirred at room temperature for 12 h. The mixture was washed with water and extracted with EtOAc. The organic layer was separated, dried over $MgSO_4$, filtered and concentrated under reduced pressure. The residue was purified by column chromatography on silica gel using EtOAc–PE (5% → 50%) as an eluent to give **BDA-15** (318 mg, 64%) yellow solid.

BDA-01: methyl 2-amino-5-phenylthiophene-3-carboxylate

The product was synthesized according to procedure **step 1** and purified by column chromatography and afforded as yellow solid (8.1 g, 68% yield), M.P.= 178 - 182 °C; ^{1}H NMR (500 MHz, $CDCl_3$) δ 7.46 (dd, *J* = 8.4, 1.2 Hz, 2H), 7.35 (t, *J* = 7.8 Hz, 2H), 7.26 (s, 1H), 7.23 (s, 1H), 6.05 (s, 2H), 3.86 (s, 3H). ^{13}C NMR (126 MHz, $CDCl_3$) δ 165.80, 162.22, 133.94, 128.85, 126.65, 126.61, 124.70, 121.10, 107.61, 51.15. HRMS (ESI) m/z calculated for $C_{12}H_{11}NO_2S$ $[M+H]^+$: 234.0524; found $[M+H]^+$: 234.0521.

BDA-02: methyl 2-((tert-butoxycarbonyl)amino)-5-phenylthiophene-3-carboxylate

The product was synthesized according to procedure **step 2** and purified by column chromatography and afforded as yellow solid (1.3 g, 81% yield), M.P.= 176 - 181 °C; ^{1}H NMR (500 MHz, $CDCl_3$) δ 7.63 – 7.61 (m, 1H), 7.60 (s, 2H), 7.42 (t, *J* = 7.6 Hz, 2H), 7.35 (t, *J* = 7.3 Hz, 1H), 3.87 (s, 3H), 1.46 (s, 9H). ^{13}C NMR (126 MHz, $CDCl_3$) δ 165.74, 152.12, 150.50, 133.76, 128.93, 127.26, 127.22, 125.17, 119.41, 111.77, 82.50, 51.68, 28.22. HRMS (ESI) m/z calculated for $C_{17}H_{19}NO_4S$ $[M+H]^+$: 334.1015; found $[M+H]^+$: 334.1013.

BDA-03: 2-((tert-butoxycarbonyl)amino)-5-phenylthiophene-3-carboxylic acid

The product was synthesized according to procedure **step 3** and purified by column chromatography and afforded as white solid (1.2 g, 70% yield), M.P.= 196 - 201 °C;
^{1}H NMR (500 MHz, DMSO- d_6) δ 12.58 (s, 1H), 7.54 (d, *J* = 8.2 Hz, 2H), 7.42 (s, 1H), 7.36 (t, *J* = 7.8 Hz, 2H), 7.21 (t, *J* = 7.3 Hz, 1H), 1.50 (s, 9H).^{13}C NMR (126 MHz, DMSO- d_6) δ 168.99, 152.14, 144.23, 134.99, 129.49, 129.01, 126.79, 124.65, 123.51, 122.97, 80.82, 28.42. HRMS (ESI) m/z calculated for $C_{16}H_{17}NO_4S$ $[M+H]^+$: 320.0937; found $[M+H]^+$: 320.0931.

BDA-04: tert-butyl (3-(phenethylcarbamoyl)-5-phenylthiophen-2-yl)carbamate

The product was synthesized according to procedure **step 4** and purified by column chromatography and afforded as yellow solid (106 mg, 74% yield), M.P.= 168 - 173 °C; ^{1}H NMR (500 MHz, $CDCl_3$) δ 10.97 (s, 1H), 7.53 (d, *J* = 8.2 Hz, 2H), 7.37 (t, *J* = 7.5 Hz, 4H), 7.29 (s, 1H), 7.27 (d, *J* = 7.1 Hz, 3H), 6.96 (s, 1H), 5.98 (s, 1H), 3.70 (q, *J* = 6.9 Hz, 2H), 2.96 (t, *J* = 7.0 Hz, 2H), 1.57 (s, 9H). ^{13}C NMR (126 MHz, $CDCl_3$) δ 165.23, 152.46, 148.56, 138.77, 133.76, 132.83,

128.97, 128.85, 128.80, 127.31, 126.73, 125.17, 116.09, 113.67, 81.99, 40.71, 35.90, 28.26. HRMS (ESI) m/z calculated for $C_{24}H_{26}N_2O_3S$ $[M+H]^+$: 423.1738; found $[M+H]^+$: 423.1733.

BDA-05: 2-amino-N-phenethyl-5-phenylthiophene-3-carboxamide

The product was synthesized according to procedure **step 5** and afforded as yellow solid (45 mg, 98% yield), M.P.= 198 - 202 °C; ^{1}H NMR (500 MHz, $CDCl_3$) δ 7.41 (d, *J* = 7.7 Hz, 2H), 7.35 (q, *J* = 8.0 Hz, 4H), 7.31 – 7.19 (m, 5H), 6.88 (s, 1H), 5.92 (s, 2H), 3.67 (d, *J* = 6.3 Hz, 2H), 2.94 (d, *J* = 6.8 Hz, 2H). ^{13}C NMR (126 MHz, $CDCl_3$) δ 165.98, 160.54, 139.18, 134.05, 128.92, 128.71, 126.62, 126.55, 125.17, 124.64, 118.31, 109.71, 40.64, 36.13. HRMS (ESI) m/z calculated for $C_{19}H_{18}N_2OS$ $[M+H]^+$: 323.1147; found $[M+H]^+$: 323.1141.

BDA-06: tert-butyl (5-phenyl-3-((4-sulfamoylphenethyl)carbamoyl) thiophen-2-yl)carbamate

The product was synthesized according to procedure **step 4** and purified by column chromatography and afforded as yellow solid (333 mg, 85% yield), M.P.= 198 - 203 °C;
^{1}H NMR (500 MHz, DMSO- *d*$_6$) δ 11.29 (s, 1H), 7.82 (s, 1H), 7.77 (d, *J* = 8.4 Hz, 1H), 7.55 (d, *J* = 7.1 Hz, 2H), 7.48 – 7.40 (m, 4H), 7.31 (s, 2H), 5.77 (s, 2H), 3.52 (q, *J* = 7.9, 7.3 Hz, 2H), 2.94 (t, *J* = 7.3 Hz, 2H), 1.51 (s, 9H). ^{13}C NMR (126 MHz, DMSO- *d*$_6$) δ 165.22, 151.84, 147.14, 144.02, 142.59, 133.92, 131.49, 129.70, 129.61, 127.76, 126.35, 126.14, 125.06, 124.91, 119.38, 114.99, 82.15, 55.40, 35.22, 28.18. HRMS (ESI) m/z calculated for $C_{24}H_{27}N_3O_5S_2$ $[M+H]^+$: 502.1427; found $[M+H]^+$: 502.1424.

BDA-07: 2-amino-5-phenyl-N-(4-sulfamoylphenethyl)thiophene-3-carboxamide

The product was synthesized according to procedure **step 5** and afforded as red solid (89 mg, 98% yield), M.P.= 228 - 233 °C; ^{1}H NMR (500 MHz, DMSO- *d*$_6$) δ 7.93 (t, *J* = 5.7 Hz, 1H), 7.77 (d, *J* = 8.2 Hz, 2H), 7.59 (s, 1H), 7.49 (s, 2H), 7.45 (d, *J* = 8.4 Hz, 2H), 7.40 (d, *J* = 6.8 Hz, 2H), 7.36 (t, *J* = 7.7 Hz, 2H), 7.32 (s, 2H), 7.18 (t, *J* = 7.1 Hz, 1H), 3.49 – 3.43 (m, 2H), 2.91 (t, *J* = 7.3 Hz, 2H). ^{13}C NMR (126 MHz, DMSO- *d*$_6$) δ 165.80, 165.78, 161.34, 161.24, 144.43, 142.17, 142.14, 134.59, 129.30, 128.86, 126.18, 126.12, 126.05, 123.96, 123.01, 122.98, 119.72, 108.93, 40.15, 35.68. HRMS (ESI) m/z calculated for $C_{19}H_{19}N_3O_3S_2$ $[M+H]^+$: 402.0917; found $[M+H]^+$: 402.0914.

BDA-08: 2-(2-(1H-indol-3-yl)acetamido)-N-phenethyl-5-phenylthiophene-3-carboxamide

The product was synthesized according to procedure **step 7** and purified by column chromatography and afforded as brown solid (157 mg, 78% yield), M.P.= 201 - 203 °C; ^{1}H NMR (500 MHz, DMSO- d_6) δ 12.06 (s, 1H), 11.12 (s, 1H), 8.44 (t, *J* = 5.7 Hz, 1H), 7.81 (s, 1H), 7.54 (t, *J* = 8.8 Hz, 3H), 7.46 – 7.38 (m, 4H), 7.30 (q, *J* = 9.0, 8.4 Hz, 3H), 7.22 (dd, *J* = 11.4, 7.2 Hz, 3H), 7.10 (t, *J* = 7.6 Hz, 1H), 7.00 (t, *J* = 6.9 Hz, 1H), 3.97 (s, 2H), 3.42 (q, *J* = 8.4, 7.3 Hz, 2H), 2.80 (t, *J* = 7.6 Hz, 2H). ^{13}C NMR (126 MHz, DMSO- d_6) δ 169.31, 164.79, 145.09, 139.79, 136.78, 134.03, 132.16, 129.72, 129.12, 128.87, 127.77, 127.47, 126.65, 125.35, 125.23, 125.06, 121.75, 119.20, 118.87, 116.48, 112.05, 107.21, 40.87, 35.43, 33.56. HRMS (ESI) m/z calculated for $C_{29}H_{25}N_3O_2S$ $[M+H]^+$: 480.1743; found $[M+H]^+$: 480.1740.

BDA-09: tert-butyl (3-(4-(((tert-butoxycarbonyl)amino)methyl)piperidine-1-carbonyl)-5-phenylthiophen-2-yl)carbamate

The product was synthesized according to procedure **step 4** and purified by column chromatography and afforded as yellow solid (264 mg, 80% yield), M.P.= 170 - 173 °C; ^{1}H NMR (500 MHz, $CDCl_3$) δ 10.95 (s, 1H), 7.58 (d, *J* = 7.1 Hz, 2H), 7.38 (t, *J* = 7.7 Hz, 2H), 7.29 (d, *J* = 2.2 Hz, 1H), 7.09 (s, 1H), 5.85 (d, *J* = 7.9 Hz, 1H), 4.10 (dtd, *J* = 11.3, 7.4, 3.9 Hz, 3H), 2.92 (s, 2H), 2.03 (d, *J* = 15.0 Hz, 2H), 1.63 (s, 2H), 1.56 (s, 9H), 1.49 (s, 9H), 1.48 – 1.39 (m, 2H). ^{13}C NMR (126 MHz, $CDCl_3$) δ 164.67, 154.74, 152.39, 148.81, 133.73, 132.93, 128.97, 127.36, 125.21, 116.12, 113.47, 82.00, 79.82, 47.02, 32.24, 28.45, 28.24. HRMS (ESI) m/z calculated for $C_{27}H_{37}N_3O_5S$ $[M+H]^+$: 516.2517; found $[M+H]^+$: 516.2514.

BDA-10: tert-butyl (3-(4-(4-fluorophenyl)piperazine-1-carbonyl)-5-phenylthiophen-2-yl)carbamate

The product was synthesized according to procedure **step 4** and purified by column chromatography and afforded as yellow solid (159 mg, 83% yield), M.P.= 178 - 182 °C; ^{1}H NMR (500 MHz, $CDCl_3$) δ 9.60 (s, 1H), 7.57 (d, *J* = 7.1 Hz, 2H), 7.39 (t, *J* = 7.7 Hz, 2H), 7.31 – 7.28 (m, 2H), 7.07 (s, 1H), 7.04 – 6.99 (m, 2H), 6.96 – 6.91 (m, 2H), 3.94 – 3.88 (m, 4H), 3.22 – 3.15 (m, 4H), 1.56 (s, 9H). ^{13}C NMR (126 MHz, $CDCl_3$) δ 166.57, 158.66, 156.75, 152.26, 147.75, 147.54, 133.83, 132.71, 128.99, 127.33, 125.24, 118.96, 118.64, 118.57, 115.86, 115.68, 114.68, 82.11, 50.77, 28.25. HRMS (ESI) m/z calculated for $C_{26}H_{28}FN_3O_3S$ $[M+H]^+$: 482.1836; found $[M+H]^+$: 482.1832.

BDA-11: tert-butyl (3-((4-hydroxyphenethyl)carbamoyl)-5-phenylthiophen-2-yl)carbamate

The product was synthesized according to procedure **step 4** and purified by column chromatography and afforded as yellow solid (195 mg, 91% yield), M.P.= 174 - 176 °C; ^{1}H NMR (500 MHz, $CDCl_3$) δ 10.94 (s, 1H), 7.51 (dd, *J* = 8.3, 1.3 Hz, 2H), 7.36 (t, *J* = 7.7 Hz, 2H), 7.27 (t, *J* = 7.4 Hz, 1H), 7.11 (d, *J* = 8.5 Hz, 2H), 6.95 (s, 1H), 6.84 (d, *J* = 8.5 Hz, 2H), 6.02 (t, *J* = 6.0 Hz, 1H), 5.47 (s, 1H), 3.64 (q, *J* = 7.0 Hz, 2H), 2.87 (t, *J* = 6.9 Hz, 2H), 1.56 (s, 9H). ^{13}C NMR (126 MHz, $CDCl_3$) δ 165.30, 154.57, 152.45, 148.51, 133.71, 132.88, 130.63, 129.94, 128.96, 127.32, 125.17, 116.11, 115.68, 113.70, 82.06, 40.92, 34.93, 28.25. HRMS (ESI) m/z calculated for $C_{24}H_{26}N_2O_4S$ $[M+H]^+$: 439.1655; found $[M+H]^+$: 439.1651.

BDA-12: 2-(isopentylamino)-N-phenethyl-5-phenylthiophene-3-carboxamide

The product was synthesized according to procedure **step 6** and purified by column chromatography and afforded as yellow solid (231 mg, 75% yield), M.P.= 172 - 175 °C; ^{1}H NMR (500 MHz, $CDCl_3$) δ 8.18 (t, *J* = 5.6 Hz, 1H), 7.43 (d, *J* = 8.2 Hz, 2H), 7.35 (dt, *J* = 11.7, 8.0 Hz, 4H), 7.28 (d, *J* = 2.8 Hz, 3H), 7.20 (t, *J* = 7.4 Hz, 1H), 6.91 (s, 1H), 5.69 (t, *J* = 6.0 Hz, 1H), 3.67 (d, *J* = 6.0 Hz, 2H), 3.39 – 3.17 (m, 2H), 2.93 (t, *J* = 6.9 Hz, 2H), 1.79 (dt, *J* = 13.4, 6.7 Hz, 1H), 1.64 (q, *J* = 7.0 Hz, 2H), 0.99 (d, *J* = 6.6 Hz, 6H). ^{13}C NMR (126 MHz, $CDCl_3$) δ 167.77, 166.19, 139.20, 128.89, 128.68, 126.53, 124.13, 118.56, 45.94, 40.46, 38.08, 36.22, 25.74, 22.46, 0.01. HRMS (ESI) m/z calculated for $C_{24}H_{28}N_2OS$ $[M+H]^+$: 393.1967; found $[M+H]^+$: 393.1963.

BDA-13: 2-(((1H-imidazol-2-yl)methyl)amino)-N-phenethyl-5-phenylthiophene-3-carboxamide

The product was synthesized according to procedure **step 6** and purified by column chromatography and afforded as yellow solid (131 mg, 75% yield), M.P.= 185 - 189 °C; ^{1}H NMR (500 MHz, $CDCl_3$) δ 9.05 (s, 1H), 8.33 (d, *J* = 8.8 Hz, 2H), 7.83 (s, 1H), 7.65 – 7.55 (m, 2H), 7.42 – 7.31 (m, 8H), 7.29 (s, 2H), 7.02 (s, 1H), 3.83 (q, *J* = 6.1 Hz, 2H), 2.97 (t, *J* = 6.1 Hz, 2H). ^{13}C NMR (126 MHz, $CDCl_3$) δ 162.54, 151.01, 146.86, 143.71, 140.09, 138.45, 133.10, 131.97, 129.21, 129.05, 128.88, 128.72, 128.47, 126.60, 125.72, 125.09, 124.65, 39.95, 35.65. HRMS (ESI) m/z calculated for $C_{23}H_{22}N_4OS$ $[M+H]^+$: 403.1529; found $[M+H]^+$: 403.1525.

BDA-14: tert-butyl (3-((2-hydroxyethyl)carbamoyl)-5-phenylthiophen-2-yl)carbamate

The product was synthesized according to procedure **step 4** and purified by column chromatography and afforded as brown solid (165 mg, 61% yield), M.P.= 166 - 171 °C; ^{1}H NMR (500 MHz, $CDCl_3$) δ 10.88 (s, 1H), 9.95 (s, 1H), 7.58 (dd, *J* = 14.0, 7.3 Hz, 2H), 7.45 (s, 1H), 7.38 (q, *J* = 7.8 Hz, 2H), 7.29 (s, 1H), 7.14 (s, 1H), 6.49 (t, *J* = 5.6 Hz, 1H), 3.89 (t, *J* = 5.0 Hz, 2H), 3.64 (q, *J* = 5.6 Hz, 2H), 1.57 (s, 9H). ^{13}C NMR (126 MHz, $CDCl_3$) δ 166.18, 148.84, 133.72, 133.61, 132.96, 128.97, 127.39, 127.35, 125.25, 125.17, 119.77, 116.31, 113.35, 82.09, 62.43, 42.24, 28.25, 28.22. HRMS (ESI) m/z calculated for $C_{18}H_{22}N_2O_4S$ $[M+H]^+$: 363.1379; found $[M+H]^+$: 363.1373.

BDA-15: 2-(3-(benzo[d][1,3]dioxol-5-yl)propanamido)-N-phenethyl-5-phenylthiophene-3-carboxamide

The product was synthesized according to procedure **step 7** and purified by column chromatography and afforded as yellow solid (318 mg, 64% yield), M.P.= 188 - 190 °C; ^{1}H NMR (500 MHz, $CDCl_3$) δ 11.97 (s, 1H), 7.57 – 7.53 (m, 2H), 7.38 (q, *J* = 7.5 Hz, 4H), 7.31 (d, *J* = 7.6 Hz, 2H), 7.28 – 7.26 (m, 2H), 6.97 (s, 1H), 6.76 (d, *J* = 7.6 Hz, 2H), 6.72 (dd, *J* = 7.9, 1.7 Hz, 1H), 6.05 (t, *J* = 6.0 Hz, 1H), 5.94 (s, 2H), 3.71 (q, *J* = 6.9 Hz, 2H), 3.04 (t, *J* = 7.7 Hz, 2H), 2.96 (t, *J* = 6.9 Hz, 2H), 2.80 (t, *J* = 7.8 Hz, 2H). ^{13}C NMR (126 MHz, $CDCl_3$) δ 169.22, 165.29, 147.71, 146.19, 146.03, 138.63, 134.29, 134.07, 133.68, 129.01, 128.83, 127.54, 126.79, 125.47, 121.22, 115.60, 115.08, 108.88, 108.35, 100.86, 40.71, 38.64, 35.81, 30.92. HRMS (ESI) m/z calculated for $C_{29}H_{26}N_2O_4S$ $[M+H]^+$: 499.1667; found $[M+H]^+$: 499.1662.

BDA-16: tert-butyl (3-((2-hydroxy-2-phenylethyl)carbamoyl)-5-phenylthiophen-2-yl)carbamate

The product was synthesized according to procedure **step 4** and purified by column chromatography and afforded as brown solid (235 mg, 71% yield), M.P.= 191 - 194 °C; ^{1}H NMR (500 MHz, DMSO- d_6) δ 11.32 (s, 1H), 8.57 (s, 1H), 7.95 (s, 1H), 7.57 (d, *J* = 7.1 Hz, 2H), 7.44 (t, *J* = 7.8 Hz, 2H), 7.40 (d, *J* = 6.8 Hz, 2H), 7.36 (t, *J* = 7.6 Hz, 2H), 7.29 (dt, *J* = 12.3, 7.3 Hz, 2H), 5.60 (d, *J* = 4.4 Hz, 1H), 4.83 – 4.73 (m, 1H), 3.56 – 3.48 (m, 1H), 3.26 (ddd, *J* = 13.6, 8.5, 5.4 Hz, 1H), 1.52 (s, 9H). ^{13}C NMR (126 MHz, DMSO- d_6) δ 165.34, 151.86, 147.08, 144.16, 134.02, 131.35, 129.71, 128.60, 127.69, 127.60, 126.43, 125.02, 124.83, 119.91, 119.75,

115.16, 82.11, 71.59, 47.72, 28.19. HRMS (ESI) m/z calculated for $C_{24}H_{26}N_2O_4S$ $[M+H]^+$: 439.1637; found $[M+H]^+$: 439.1634.

BDA-17: tert-butyl (2-((3-((4-hydroxyphenethyl)carbamoyl)-5-phenylthiophen-2-yl)amino)-2-oxoethyl)carbamate

The product was synthesized according to procedure **step 4** and purified by column chromatography and afforded as brown solid (215 mg, 86% yield), M.P.= 181 - 184 °C;
^{1}H NMR (500 MHz, $CDCl_3$) δ 7.41 (d, *J* = 6.9 Hz, 2H), 7.33 (t, *J* = 7.8 Hz, 2H), 7.25 – 7.18 (m, 3H), 7.06 (d, *J* = 8.5 Hz, 2H), 6.91 (s, 1H), 6.25 (s, 2H), 5.98 (t, *J* = 6.1 Hz, 1H), 5.16 (t, *J* = 5.9 Hz, 1H), 4.19 (d, *J* = 5.8 Hz, 2H), 3.63 (q, *J* = 6.9 Hz, 2H), 2.90 (t, *J* = 6.9 Hz, 2H), 1.50 (s, 9H). ^{13}C NMR (126 MHz, $CDCl_3$) δ 169.34, 165.88, 160.41, 155.78, 148.88, 137.17, 133.92, 129.94, 128.88, 126.64, 125.29, 124.65, 121.49, 118.09, 115.60, 109.64, 80.33, 42.63, 40.53, 35.48, 28.34. HRMS (ESI) m/z calculated for $C_{26}H_{29}N_3O_5S$ $[M+H]^+$: 496.1816; found $[M+H]^+$: 496.1813.

BDA-18: tert-butyl (3-((2-methoxyphenethyl)carbamoyl)-5-phenylthiophen-2-yl)carbamate

The product was synthesized according to procedure **step 4** and purified by column chromatography and afforded as yellow solid (157 mg, 87% yield), M.P.= 179 - 182 °C;
^{1}H NMR (500 MHz, $CDCl_3$) δ 11.01 (s, 1H), 7.55 (dd, *J* = 8.4, 1.3 Hz, 2H), 7.38 (t, *J* = 7.7 Hz, 2H), 7.30 – 7.29 (m, 1H), 7.28 – 7.26 (m, 1H), 7.21 (dd, *J* = 7.3, 1.8 Hz, 1H), 7.01 – 6.90 (m, 3H), 6.39 (s, 1H), 3.92 (s, 3H), 3.70 – 3.59 (m, 2H), 2.98 (t, *J* = 6.5 Hz, 2H), 1.56 (s, 9H). ^{13}C NMR (126 MHz, $CDCl_3$) δ 165.29, 157.37, 152.49, 148.21, 133.93, 132.62, 130.83, 129.00, 128.16, 127.49, 127.24, 125.09, 121.12, 116.31, 113.98, 110.66, 81.88, 55.59, 40.44, 30.00, 28.26. HRMS (ESI) m/z calculated for $C_{25}H_{28}N_2O_4S$ $[M+H]^+$: 453.1829; found $[M+H]^+$: 453.1826.

BDA-19: N-(4-hydroxyphenethyl)-2-(isopentylamino)-5-phenylthiophene-3-carboxamide

The product was synthesized according to procedure **step 6** and purified by column chromatography and afforded as yellow solid (146 mg, 75% yield), M.P.= 188 - 190 °C;
^{1}H NMR (500 MHz, $CDCl_3$) δ 9.07 (s, 1H), 7.70 (s, 1H), 7.61 – 7.52 (m, 2H), 7.37 (hept, *J* = 7.8 Hz, 3H), 7.29 (s, 1H), 7.15 – 7.06 (m, 2H), 6.86 (dd, *J* = 14.8, 8.5 Hz, 2H), 5.53 (s, 1H), 3.76 – 3.66 (m, 2H), 3.04 – 2.61 (m, 4H), 1.53 – 1.44 (m, 1H), 1.38 – 1.15 (m, 2H), 0.87 (d, *J* = 6.6 Hz, 6H). ^{13}C NMR (126 MHz, $CDCl_3$) δ 163.26, 157.62, 154.71, 133.95, 131.10, 129.94, 129.70,

129.17, 128.88, 127.66, 125.43, 122.25, 115.56, 57.71, 40.19, 35.79, 34.56, 26.42, 22.60. HRMS (ESI) m/z calculated for $C_{24}H_{28}N_2O_2S$ [M+H]$^+$: 409.1954; found [M+H]$^+$: 409.1951.

BDA-20: tert-butyl (3-((2-(cyclohex-1-en-1-yl)ethyl)carbamoyl)-5-phenylthiophen-2-yl)carbamate

The product was synthesized according to procedure **step 4** and purified by column chromatography and afforded as yellow solid (152 mg, 62% yield), M.P.= 174 - 176 °C; ^{1}H NMR (500 MHz, $CDCl_3$) δ 10.99 (s, 1H), 7.55 (d, *J* = 7.1 Hz, 2H), 7.38 (t, *J* = 7.8 Hz, 2H), 7.28 (t, *J* = 7.4 Hz, 1H), 7.04 (s, 1H), 6.01 (t, *J* = 5.5 Hz, 1H), 5.58 (s, 1H), 3.55 – 3.45 (m, 2H), 2.27 (t, *J* = 7.5 Hz, 2H), 2.02 (d, *J* = 29.3 Hz, 4H), 1.70 – 1.65 (m, 2H), 1.63 – 1.58 (m, 2H), 1.55 (s, 9H). ^{13}C NMR (126 MHz, $CDCl_3$) δ 165.13, 152.47, 148.30, 134.72, 133.82, 132.77, 128.99, 127.30, 125.15, 123.96, 116.13, 113.87, 81.92, 37.63, 37.09, 28.25, 27.93, 25.33, 22.83, 22.37. HRMS (ESI) m/z calculated for $C_{24}H_{30}N_2O_4S$ [M+H]$^+$: 427.2079; found [M+H]$^+$: 427.2074.

BDA-21: tert-butyl (5-phenyl-3-((4-(trifluoromethyl)phenethyl)carbamoyl) thiophen-2-yl)carbamate

The product was synthesized according to procedure **step 4** and purified by column chromatography and afforded as yellow solid (169 mg, 89% yield), M.P.= 180 - 183 °C; ^{1}H NMR (500 MHz, $CDCl_3$) δ 10.94 (s, 1H), 7.61 (d, *J* = 8.2 Hz, 2H), 7.53 (d, *J* = 7.4 Hz, 2H), 7.37 (t, *J* = 6.7 Hz, 4H), 7.31 – 7.27 (m, 1H), 6.99 (s, 1H), 6.06 (t, *J* = 6.1 Hz, 1H), 3.70 (q, *J* = 6.9 Hz, 2H), 3.02 (t, *J* = 7.1 Hz, 2H), 1.57 (s, 9H). ^{13}C NMR (126 MHz, $CDCl_3$) δ 165.34, 152.44, 148.74, 142.93, 133.65, 132.96, 129.18, 129.00, 127.40, 125.72, 125.69, 125.66, 125.63, 125.16, 116.01, 113.45, 82.12, 40.56, 35.83, 28.25. HRMS (ESI) m/z calculated for $C_{25}H_{25}F_3N_2O_3S$ [M+H]$^+$: 491.1538; found [M+H]$^+$: 491.1532.

BDA-22: tert-butyl (3-((4-bromophenethyl)carbamoyl)-5-phenylthiophen-2-yl)carbamate

The product was synthesized according to procedure **step 4** and purified by column chromatography and afforded as yellow solid (181 mg, 92% yield), M.P.= 190 - 192 °C; ^{1}H NMR (500 MHz, $CDCl_3$) δ 10.95 (s, 1H), 7.53 (d, *J* = 8.0 Hz, 2H), 7.47 (d, *J* = 8.4 Hz, 2H), 7.37 (t, *J* = 7.7 Hz, 2H), 7.28 (t, *J* = 7.4 Hz, 1H), 7.13 (d, *J* = 8.4 Hz, 2H), 6.98 (s, 1H), 6.03 (t, *J* = 6.0 Hz, 1H), 3.66 (q, *J* = 7.1 Hz, 2H), 2.91 (t, *J* = 7.0 Hz, 2H), 1.57 (s, 9H). ^{13}C NMR (126 MHz,

$CDCl_3$) δ 165.29, 152.44, 148.67, 137.75, 133.68, 132.91, 131.84, 130.57, 129.00, 127.37, 125.18, 120.56, 116.05, 113.52, 82.07, 40.61, 35.38, 28.26. HRMS (ESI) m/z calculated for $C_{24}H_{25}BrN_2O_3S$ $[M+H]^+$: 501.0878; found $[M+H]^+$: 501.0875.

BDA-23: tert-butyl (3-((4-methylphenethyl)carbamoyl)-5-phenylthiophen-2-yl)carbamate

The product was synthesized according to procedure **step 4** and purified by column chromatography and afforded as yellow solid (248 mg, 88% yield), M.P.= 170 - 173 °C;

^1H NMR (500 MHz, $CDCl_3$) δ 10.98 (s, 1H), 7.54 (d, *J* = 7.1 Hz, 2H), 7.38 (t, *J* = 7.7 Hz, 2H), 7.30 – 7.26 (m, 1H), 7.20 – 7.14 (m, 4H), 6.96 (s, 1H), 5.97 (s, 1H), 3.68 (d, *J* = 5.8 Hz, 2H), 2.92 (t, *J* = 6.9 Hz, 2H), 2.37 (s, 3H), 1.57 (s, 9H). ^{13}C NMR (126 MHz, $CDCl_3$) δ 165.21, 152.47, 148.52, 136.30, 135.63, 133.77, 132.77, 129.49, 128.97, 128.73, 127.30, 125.16, 116.13, 113.70, 81.98, 40.79, 35.42, 28.27, 21.09. HRMS (ESI) m/z calculated for $C_{25}H_{28}N_2O_3S$ $[M+H]^+$: 437.1869; found $[M+H]^+$: 437.1863.

BDA-24: tert-butyl (3-((3-methoxyphenethyl)carbamoyl)-5-phenylthiophen-2-yl)carbamate

The product was synthesized according to procedure **step 4** and purified by column chromatography and afforded as yellow solid (261 mg, 90% yield), M.P.= 175 - 177 °C;

^1H NMR (500 MHz, $CDCl_3$) δ 10.99 (s, 1H), 7.52 (d, *J* = 7.7 Hz, 2H), 7.36 (t, *J* = 7.7 Hz, 2H), 7.29 (d, *J* = 7.9 Hz, 1H), 7.27 (d, *J* = 2.4 Hz, 1H), 7.00 (s, 1H), 6.87 – 6.79 (m, 3H), 6.11 (t, *J* = 6.0 Hz, 1H), 3.81 (s, 3H), 3.69 (q, *J* = 6.9 Hz, 2H), 2.92 (t, *J* = 7.0 Hz, 2H), 1.56 (s, 9H). ^{13}C NMR (126 MHz, $CDCl_3$) δ 165.25, 159.91, 152.46, 148.48, 140.36, 133.74, 132.80, 129.83, 128.97, 127.31, 125.12, 121.14, 116.22, 114.51, 113.75, 112.05, 82.01, 55.20, 40.59, 35.92, 28.26. HRMS (ESI) m/z calculated for $C_{25}H_{28}N_2O_4S$ $[M+H]^+$: 453.1836; found $[M+H]^+$: 453.1831.

BDA-25: tert-butyl (3-((3,4-dihydroxyphenethyl)carbamoyl)-5-phenylthiophen-2-yl)carbamate

The product was synthesized according to procedure **step 4** and purified by column chromatography and afforded as yellow solid (161 mg, 63% yield), M.P.= 197 - 201 °C;

^1H NMR (500 MHz, $CDCl_3$) δ 10.86 (s, 1H), 7.50 (d, *J* = 7.6 Hz, 2H), 7.35 (t, *J* = 7.7 Hz, 2H), 7.26 (t, *J* = 7.3 Hz, 1H), 6.95 (s, 1H), 6.85 (d, *J* = 7.9 Hz, 1H), 6.77 (d, *J* = 2.2 Hz, 1H), 6.65 (d, *J* = 8.0 Hz, 1H), 6.07 (t, *J* = 5.9 Hz, 1H), 3.60 (q, *J* = 6.9 Hz, 2H), 2.79 (t, *J* = 6.9 Hz, 2H), 1.55 (s,

9H). ^{13}C NMR (126 MHz, $CDCl_3$) δ 165.37, 152.44, 148.44, 144.00, 142.57, 133.59, 132.95, 131.36, 128.97, 127.37, 125.13, 121.10, 116.13, 115.67, 115.57, 113.65, 82.21, 40.85, 35.08, 28.25. HRMS (ESI) m/z calculated for $C_{24}H_{26}N_2O_5S$ $[M+H]^+$: 455.1646; found $[M+H]^+$: 455.1641.

BDA-26: tert-butyl (5-phenyl-3-((3-phenylpropyl)carbamoyl)thiophen-2-yl)carbamate

The product was synthesized according to procedure **step 4** and purified by column chromatography and afforded as yellow solid (238 mg, 83% yield), M.P.= 176 - 179 °C;

^1H NMR (500 MHz, $CDCl_3$) δ 10.99 (s, 1H), 7.55 (d, J = 7.1 Hz, 2H), 7.39 (t, J = 7.7 Hz, 2H), 7.34 (t, J = 7.6 Hz, 2H), 7.29 (d, J = 6.8 Hz, 1H), 7.24 (dd, J = 11.7, 7.1 Hz, 3H), 6.85 (s, 1H), 5.86 (t, J = 5.8 Hz, 1H), 3.56 – 3.45 (m, 2H), 2.77 (t, J = 7.4 Hz, 2H), 2.01 (p, J = 7.0 Hz, 2H), 1.55 (s, 9H). ^{13}C NMR (126 MHz, $CDCl_3$) δ 165.18, 152.46, 148.37, 141.42, 133.78, 132.69, 128.95, 128.68, 128.47, 127.29, 126.25, 125.17, 116.17, 113.65, 81.94, 39.42, 33.77, 31.06, 28.26. HRMS (ESI) m/z calculated for $C_{25}H_{28}N_2O_3S$ $[M+H]^+$: 437.1867; found $[M+H]^+$: 437.1862.

BDA-27: tert-butyl (3-((2-oxo-2-phenylethyl)carbamoyl)-5-phenylthiophen-2-yl)carbamate

The product was synthesized according to procedure **step 4** and purified by column chromatography and afforded as yellow solid (188 mg, 74% yield), M.P.= 184 - 187 °C;

^1H NMR (500 MHz, $CDCl_3$) δ 10.89 (s, 1H), 8.06 (d, J = 8.0 Hz, 2H), 7.73 – 7.66 (m, 1H), 7.63 (d, J = 7.3 Hz, 2H), 7.57 (t, J = 7.8 Hz, 2H), 7.41 (t, J = 7.8 Hz, 2H), 7.31 (d, J = 10.1 Hz, 2H), 7.08 (t, J = 4.3 Hz, 1H), 4.95 (d, J = 4.3 Hz, 2H), 1.57 (s, 9H). ^{13}C NMR (126 MHz, $CDCl_3$) δ 193.99, 165.19, 152.39, 148.96, 134.43, 134.25, 133.72, 133.04, 129.09, 129.01, 128.01, 127.38, 125.27, 116.49, 113.33, 82.05, 46.24, 28.26. HRMS (ESI) m/z calculated for $C_{24}H_{24}N_2O_4S$ $[M+H]^+$: 437.1545; found $[M+H]^+$: 437.1540.

BDA-28: tert-butyl (3-((4-fluorophenethyl)carbamoyl)-5-phenylthiophen-2-yl)carbamate

The product was synthesized according to procedure **step 4** and purified by column chromatography and afforded as yellow solid (164 mg, 90% yield), M.P.= 181 - 184 °C;

^1H NMR (500 MHz, $CDCl_3$) δ 10.96 (s, 1H), 7.53 (d, J = 7.1 Hz, 2H), 7.37 (t, J = 7.7 Hz, 2H), 7.28 (t, J = 7.4 Hz, 1H), 7.21 (dd, J = 8.7, 5.4 Hz, 2H), 7.04 (t, J = 8.7 Hz, 2H), 6.99 (s, 1H), 6.05 (t, J = 6.1 Hz, 1H), 3.70 – 3.62 (m, 2H), 2.92 (t, J = 7.1 Hz, 2H), 1.56 (s, 9H). ^{13}C NMR (126 MHz, $CDCl_3$) δ 165.28, 162.71, 160.77, 152.45, 148.61, 134.42, 134.40, 133.70, 132.88, 130.27, 130.21,

129.00, 127.36, 125.16, 116.07, 115.67, 115.50, 113.59, 82.05, 40.85, 35.14, 28.26. HRMS (ESI) m/z calculated for $C_{24}H_{25}N_2O_3S$ $[M+H]^+$: 441.1667; found $[M+H]^+$: 441.1662.

BDA-29: tert-butyl (3-((4-methoxyphenethyl)carbamoyl)-5-phenylthiophen-2-yl)carbamate

The product was synthesized according to procedure **step 4** and purified by column chromatography and afforded as yellow solid (178 mg, 87% yield), M.P.= 178 - 181 °C; ^{1}H NMR (500 MHz, $CDCl_3$) δ 10.97 (s, 1H), 7.54 (d, *J* = 8.0 Hz, 2H), 7.37 (t, *J* = 7.7 Hz, 2H), 7.28 (d, *J* = 4.1 Hz, 1H), 7.19 (d, *J* = 8.7 Hz, 2H), 6.95 (s, 1H), 6.91 (d, *J* = 8.7 Hz, 2H), 5.94 (s, 1H), 3.83 (s, 3H), 3.70 – 3.61 (m, 2H), 2.90 (t, *J* = 6.9 Hz, 2H), 1.57 (s, 9H). ^{13}C NMR (126 MHz, $CDCl_3$) δ 165.20, 158.44, 148.55, 133.78, 132.82, 130.70, 129.79, 128.96, 127.31, 125.18, 116.08, 114.21, 113.70, 81.97, 55.31, 40.86, 34.96, 28.26. HRMS (ESI) m/z calculated for $C_{25}H_{28}N_2O_4S$ $[M+H]^+$: 453.1864; found $[M+H]^+$: 453.1863.

BDA-30: tert-butyl (3-((4-hydroxybenzyl)carbamoyl)-5-phenylthiophen-2-yl)carbamate

The product was synthesized according to procedure **step 4** and purified by column chromatography and afforded as yellow solid (185 mg, 86% yield), M.P.= 175 - 177 °C; ^{1}H NMR (500 MHz, $CDCl_3$) δ 10.97 (s, 1H), 7.55 (d, *J* = 7.1 Hz, 2H), 7.36 (t, *J* = 7.7 Hz, 2H), 7.29 (s, 1H), 7.26 (d, *J* = 8.5 Hz, 2H), 7.07 (s, 1H), 6.86 (d, *J* = 8.7 Hz, 2H), 6.15 (d, *J* = 5.8 Hz, 1H), 4.94 (s, 1H), 4.56 (d, *J* = 5.7 Hz, 2H), 1.57 (s, 9H). ^{13}C NMR (126 MHz, $CDCl_3$) δ 165.05, 155.22, 148.78, 133.71, 130.15, 129.41, 128.96, 127.33, 125.18, 116.14, 115.69, 113.44, 82.04, 42.99, 28.26. HRMS (ESI) m/z calculated for $C_{23}H_{24}N_2O_4S$ $[M+H]^+$: 425.18548; found $[M+H]^+$: 425.18545.

BDA-31: tert-butyl (5-phenyl-3-(phenylcarbamoyl)thiophen-2-yl)carbamate

The product was synthesized according to procedure **step 4** and purified by column chromatography and afforded as yellow solid (126 mg, 64%), M.P.= 169 - 175 °C; ^{1}H NMR (500 MHz, $CDCl_3$) δ 10.88 (s, 1H), 7.65 (s, 1H), 7.62 – 7.58 (m, 4H), 7.40 (td, J = 7.8, 6.0 Hz, 4H), 7.31 (dt, J = 8.0, 1.7 Hz, 1H), 7.24 (s, 1H), 7.20 (tt, J = 7.4, 1.3 Hz, 1H), 1.57 (s, 9H). ^{13}C NMR (126 MHz, $CDCl_3$) δ 163.58, 152.41, 149.65, 137.24, 133.63, 133.15, 129.13, 129.02, 127.48, 125.28, 124.87, 120.93, 116.05, 113.79, 82.24, 28.24. HRMS (ESI) m/z calculated for $C_{22}H_{22}N_2O_3S$ $[M+H]^+$: 395.1418; found $[M+H]^+$: 395.1425.

BDA-34: tert-butyl (3-(benzylcarbamoyl)-5-phenylthiophen-2-yl)carbamate

The product was synthesized according to procedure **step 4** and purified by column chromatography and afforded as brown solid (135 mg, 66%), M.P.= 171 - 177 °C; ^{1}H NMR (500 MHz, $CDCl_3$) δ 10.98 (s, 1H), 7.55 (d, J = 7.1 Hz, 2H), 7.41 – 7.33 (m, 6H), 7.27 (t, J = 7.4 Hz, 1H), 7.11 (s, 1H), 6.29 (t, J = 5.8 Hz, 1H), 4.64 (d, J = 5.8 Hz, 2H), 1.57 (s, 9H). ^{13}C NMR (126 MHz, $CDCl_3$) δ 165.14, 152.45, 148.83, 137.99, 133.73, 132.93, 128.96, 128.88, 127.79, 127.74, 127.32, 125.18, 116.21, 113.45, 82.04, 43.50, 28.26. HRMS (ESI) m/z calculated for $C_{23}H_{24}N_2O_3S$ $[M+H]^+$: 409.1523; found $[M+H]^+$: 409.1528.

BDA-37: Tert-butyl (3-((3-fluoro-4-morpholinophenyl)carbamoyl)-5-phenylthiophen-2-yl)carbamate

The product was synthesized according to procedure **step 4** and purified by column chromatography and afforded as brown solid (185 mg, 74%), M.P.= 173 - 178 °C; ^{1}H NMR (500 MHz, $CDCl_3$) δ 10.85 (s, 1H), 7.93 (s, 1H), 7.52 (dd, J = 8.4, 1.3 Hz, 2H), 7.47 (dd, J = 13.9, 2.5 Hz, 1H), 7.34 (t, J = 7.7 Hz, 2H), 7.26 (d, J = 6.0 Hz, 2H), 7.17 (dd, J = 8.6, 2.3 Hz, 1H), 6.87 (t, J = 9.1 Hz, 1H), 3.90 – 3.84 (m, 4H), 3.09 – 3.00 (m, 4H), 1.55 (s, 9H). ^{13}C NMR (126 MHz, $CDCl_3$) δ 163.62, 156.28, 154.33, 152.39, 149.44, 136.92, 136.85, 133.55, 132.94, 132.34, 132.26, 128.99, 127.43, 125.12, 118.68, 118.64, 117.01, 116.99, 116.39, 113.78, 110.22, 110.02, 82.34, 66.98, 51.00, 50.98, 28.22. HRMS (ESI) m/z calculated for $C_{26}H_{28}FN_3O_4S$ $[M+H]^+$: 498.1835; found $[M+H]^+$: 498.1838.

BDA-38: Tert-butyl (3-((1-benzylpiperidin-4-yl)carbamoyl)-5-phenylthiophen-2-yl) carbamate

The product was synthesized according to procedure **step 4** and purified by column chromatography and afforded as brown solid (196 mg, 80%), M.P.= 172 - 176 °C; ^{1}H NMR (500 MHz, $CDCl_3$) δ 11.01 (s, 1H), 7.57 (d, J = 7.7 Hz, 2H), 7.41 – 7.32 (m, 6H), 7.32 – 7.24 (m, 2H), 7.17 (s, 1H), 6.11 (d, J = 7.9 Hz, 1H), 4.07 – 3.93 (m, 1H), 3.59 (s, 2H), 2.95 (d, J = 12.1 Hz, 2H), 2.21 (t, J = 11.7 Hz, 2H), 2.01 (d, J = 9.1 Hz, 2H), 1.74 – 1.62 (m, 2H), 1.55 (s, 9H). ^{13}C NMR (126 MHz, $CDCl_3$) δ 164.76, 152.42, 148.53, 137.36, 133.79, 132.74, 129.34, 128.95, 128.35,

127.37, 127.28, 125.16, 116.51, 113.78, 81.93, 62.71, 52.19, 46.70, 31.96, 28.25. HRMS (ESI) m/z calculated for $C_{28}H_{33}N_3O_3S$ $[M+H]^+$: 492.2214; found $[M+H]^+$: 492.2219.

BDA-39: Tert-butyl (3-((2,2-diphenylethyl)carbamoyl)-5-phenylthiophen-2-yl) carbamate

The product was synthesized according to procedure **step 4** and purified by column chromatography and afforded as yellow solid (202 mg, 81%), M.P.= 170 - 174 °C; ^{1}H NMR (500 MHz, $CDCl_3$) δ 10.95 (s, 1H), 7.48 (dd, J = 8.4, 1.3 Hz, 2H), 7.40 – 7.33 (m, 6H), 7.33 – 7.29 (m, 5H), 7.29 – 7.25 (m, 2H), 6.79 (s, 1H), 5.94 (t, J = 5.9 Hz, 1H), 4.08 (dd, J = 7.9, 5.8 Hz, 2H), 1.57 (s, 9H). ^{13}C NMR (126 MHz, $CDCl_3$) δ 165.19, 152.44, 148.59, 141.78, 133.71, 132.76, 128.95, 128.87, 128.10, 127.31, 127.03, 125.16, 116.09, 113.66, 82.02, 50.59, 43.79, 28.27. HRMS (ESI) m/z calculated for $C_{30}H_{30}N_2O_3S$ $[M+H]^+$: 499.2042; found $[M+H]^+$: 499.2046.

BDA-49: Tert-butyl (3-((4-hydroxyphenethyl)carbamoyl)-4-methyl-5phenylthiophen-2-yl)carbamate

The product was synthesized according to procedure **step 4** and purified by column chromatography and afforded as yellow solid (192 mg, 85%), M.P.= 172 - 177 °C; ^{1}H NMR (500 MHz, $CDCl_3$) δ 7.42 – 7.35 (m, 2H), 7.33 – 7.30 (m, 2H), 7.29 (t, J = 2.9 Hz, 1H), 7.27 (d, J = 2.2 Hz, 2H), 7.18 – 7.09 (m, 2H), 5.96 (s, 1H), 5.83 (t, J = 5.4 Hz, 1H), 3.71 (q, J = 6.9 Hz, 2H), 2.95 (t, J = 6.9 Hz, 2H), 2.13 (s, 3H), 1.58 (s, 9H). ^{13}C NMR (126 MHz, $CDCl_3$) δ 166.66, 159.42, 151.96, 149.78, 136.60, 134.13, 129.76, 129.68, 128.48, 127.61, 126.97, 121.57, 120.96, 111.49, 83.60, 40.42, 35.00, 27.72, 15.96. HRMS (ESI) m/z calculated for $C_{25}H_{28}N_2O_4S$ $[M+H]^+$: 453.1835; found $[M+H]^+$: 453.1839.

BDA-57: Tert-butyl (3-((2-morpholinoethyl)carbamoyl)-5-phenylthiophen-2-yl) carbamate

The product was synthesized according to procedure **step 4** and purified by column chromatography and afforded as yellow solid (166 mg, 86%), M.P.= 175 - 179 °C; ^{1}H NMR (500 MHz, $CDCl_3$) δ 10.97 (s, 1H), 7.57 (d, J = 7.1 Hz, 2H), 7.39 (t, J = 7.8 Hz, 2H), 7.30 (d, J = 8.7 Hz, 1H), 7.11 (s, 1H), 6.68 (t, J = 5.0 Hz, 1H), 3.79 (t, J = 4.7 Hz, 4H), 3.55 (q, J = 5.4 Hz, 2H), 2.66 (t, J = 6.0 Hz, 2H), 2.57 (s, 4H), 1.56 (s, 9H). ^{13}C NMR (126 MHz, $CDCl_3$) δ 165.29, 152.46, 148.46, 133.81, 132.79, 129.02, 127.35, 125.20, 116.35, 113.78, 81.96, 66.95, 56.87, 53.30, 35.37, 28.26. HRMS (ESI) m/z calculated for $C_{22}H_{29}N_3O_4S$ $[M+H]^+$: 432.1952; found $[M+H]^+$: 432.1956.

BDA-59: Tert-butyl (5-benzyl-3-((4-hydroxyphenethyl)carbamoyl)thiophen-2-yl) carbamate

The product was synthesized according to procedure **step 4** and purified by column chromatography and afforded as yellow solid (188 mg, 83%), M.P.= 178 - 182 °C; ^{1}H NMR (500 MHz, $CDCl_3$) δ 7.34 (t, J = 7.3 Hz, 2H), 7.28 – 7.26 (m, 1H), 7.23 (dd, J = 8.9, 2.6 Hz, 4H), 7.15 – 7.11 (m, 2H), 6.27 (s, 1H), 5.98 (s, 1H), 5.64 (t, J = 5.8 Hz, 1H), 3.92 (s, 2H), 3.59 (q, J = 7.0 Hz, 2H), 2.88 (t, J = 6.9 Hz, 2H), 1.59 (s, 9H). ^{13}C NMR (126 MHz, $CDCl_3$) δ 165.79, 160.02, 152.09, 149.65, 139.74, 136.74, 129.76, 128.61, 128.57, 126.65, 125.92, 121.44, 119.83, 108.12, 83.60, 40.40, 35.99, 35.49, 27.73. HRMS (ESI) m/z calculated for $C_{25}H_{28}N_2O_4S$ $[M+H]^+$: 453.1842; found $[M+H]^+$: 453.1847.

BDA-60: Tert-butyl (3-((4-hydroxyphenethyl)carbamoyl)-4,5-diphenylthiophen-2-yl) carbamate

The product was synthesized according to procedure **step 4** and purified by column chromatography and afforded as yellow solid (195 mg, 76%), M.P.= 175 - 181 °C; ^{1}H NMR (500 MHz, $CDCl_3$) δ 7.32 – 7.26 (m, 3H), 7.19 (d, J = 6.3 Hz, 2H), 7.11 (d, J = 7.4 Hz, 3H), 7.06 (d, J = 8.5 Hz, 2H), 7.03 – 6.94 (m, 4H), 6.45 (s, 2H), 5.02 (s, 1H), 3.35 (q, J = 6.9 Hz, 2H), 2.52 (t, J = 7.0 Hz, 2H), 1.59 (s, 9H). ^{13}C NMR (126 MHz, $CDCl_3$) δ 166.23, 160.74, 151.97, 149.56, 136.34, 136.32, 133.86, 133.82, 130.39, 129.41, 129.05, 128.65, 128.29, 128.15, 126.42, 121.24, 109.86, 83.49, 40.01, 34.57, 27.74. HRMS (ESI) m/z calculated for $C_{30}H_{30}N_2O_4S$ $[M+H]^+$: 515.1927; found $[M+H]^+$: 515.1929.

BDA-62: Tert-butyl (3-((4-hydroxyphenethyl)carbamoyl)-5-isopropylthiophen-2-yl) carbamate

The product was synthesized according to procedure **step 4** and purified by column chromatography and afforded as yellow solid (170 mg, 84%), M.P.= 172 - 178 °C; ^{1}H NMR (500 MHz, $CDCl_3$) δ 7.27 – 7.22 (m, 2H), 7.16 – 7.12 (m, 2H), 6.26 (d, J = 1.1 Hz, 1H), 5.94 (s, 2H), 5.70 (t, J = 6.0 Hz, 1H), 3.65 – 3.53 (m, 2H), 2.93 (dd, J = 6.9, 1.2 Hz, 1H), 2.90 (t, J = 7.0 Hz, 2H), 1.58 (s, 9H), 1.25 (d, J = 6.9 Hz, 6H). ^{13}C NMR (126 MHz, $CDCl_3$) 165.88, 158.69, 152.06, 149.67, 136.78, 135.07, 129.79, 121.44, 116.20, 108.00, 83.60, 40.42, 35.57, 29.58, 27.72, 24.22. HRMS (ESI) m/z calculated for $C_{21}H_{28}N_2O_4S$ $[M+H]^+$: 405.1871; found $[M+H]^+$: 405.1876.

BDA-63: Tert-butyl (3-(4-(4-hydroxyphenyl)piperazine-1-carbonyl)-5 phenylthiophen -2-yl)carbamate

The product was synthesized according to procedure **step 4** and purified by column chromatography and afforded as yellow solid (209 mg, 87%), M.P.= 175 - 181 °C; ^{1}H NMR (500 MHz, DMSO- d_6) δ 10.00 (s, 1H), 8.89 (s, 1H), 8.33 (s, 1H), 7.63 (d, J = 8.2 Hz, 2H), 7.40 (t, J = 7.8 Hz, 2H), 7.32 (s, 1H), 7.29 (t, J = 7.4 Hz, 1H), 6.83 (d, J = 9.0 Hz, 2H), 6.68 (d, J = 8.8 Hz, 2H), 3.67 (t, J = 5.4 Hz, 4H), 3.03 (t, J = 5.2 Hz, 4H), 1.50 (s, 9H). ^{13}C NMR (126 MHz, DMSO- d_6) δ 164.86, 152.52, 151.81, 144.39, 142.04, 133.89, 129.55, 127.72, 125.36, 125.20, 121.07, 118.92, 116.02, 81.58, 50.73, 28.44, 28.31. HRMS (ESI) m/z calculated for $C_{26}H_{29}N_3O_4S$ $[M+H]^+$: 480.1915; found $[M+H]^+$: 480.1919.

BDA-46: 2-amino-N-(4-fluorophenethyl)-5-phenylthiophene-3-carboxamide

The product was synthesized according to procedure **step 5** and afforded as red solid (53 mg, 98%), M.P.= 195 - 201 °C; ^{1}H NMR (500 MHz, $CDCl_3$) δ 7.41 (d, J = 8.0 Hz, 2H), 7.35 (t, J = 7.8 Hz, 2H), 7.26 – 7.20 (m, 3H), 7.07 – 7.01 (m, 2H), 6.85 (s, 1H), 6.23 (s, 2H), 5.79 (t, J = 6.0 Hz, 1H), 3.64 (q, J = 7.1 Hz, 2H), 2.91 (t, J = 7.1 Hz, 2H).^{13}C NMR (126 MHz, $CDCl_3$) δ 165.77, 162.67, 160.72, 160.38, 134.73, 134.70, 133.89, 130.29, 130.22, 128.90, 126.74, 125.43, 124.68, 117.82, 115.57, 115.40, 109.64, 40.61, 35.34. HRMS (ESI) m/z calculated for $C_{19}H_{17}FN_2OS$ $[M+H]^+$: 341.1071; found $[M+H]^+$: 341.1074.

BDA-50: 2-amino-N-(4-hydroxyphenethyl)-5-phenylthiophene-3-carboxamide

The product was synthesized according to procedure **step 5** and afforded yellow solid (167 mg, 99%), M.P.= 192 - 197 °C; ^{1}H NMR (500 MHz, DMSO- d_6) δ 9.19 (s, 1H), 7.85 (t, J = 5.7 Hz, 1H), 7.60 (s, 1H), 7.46 (s, 2H), 7.40 (dd, J = 8.3, 1.5 Hz, 2H), 7.38 – 7.33 (m, 2H), 7.21 – 7.14 (m, 1H), 7.06 – 6.98 (m, 2H), 6.75 – 6.65 (m, 2H), 4.12 (d, J = 5.0 Hz, 2H), 2.70 (t, J = 7.6 Hz, 2H). ^{13}C NMR (126 MHz, DMSO- d_6) δ 165.71, 161.33, 156.08, 134.74, 130.15, 129.98, 129.94, 129.44, 126.31, 124.04, 121.85, 121.25, 121.06, 115.67, 115.52, 108.65, 41.06, 35.24. HRMS (ESI) m/z calculated for $C_{19}H_{18}N_2O_2S$ $[M+H]^+$: 339.1125; found $[M+H]^+$: 339.1128.

BDA-55: 2-amino-N-(4-hydroxyphenethyl)-4,5-diphenylthiophene-3-carboxamide

The product was synthesized according to procedure **step 5** and afforded yellow solid (201 mg, 97%), M.P.= 193 - 199 °C; ^{1}H NMR (500 MHz, DMSO- d_6) δ 9.19 (s, 1H), 7.40 – 7.31 (m, 3H), 7.29 (s, 2H), 7.17 (dd, J = 7.6, 1.8 Hz, 2H), 7.15 – 7.11 (m, 2H), 7.11 – 7.06 (m, 1H), 6.99 – 6.91 (m, 2H), 6.78 (d, J = 8.5 Hz, 2H), 6.67 – 6.61 (m, 2H), 5.39 (t, J = 5.4 Hz, 1H), 3.16 – 3.04 (m,

2H), 2.26 (t, J = 7.3 Hz, 2H). ^{13}C NMR (126 MHz, DMSO- d_6) δ 165.58, 160.22, 156.11, 136.59, 134.66, 134.37, 130.49, 129.39, 129.28, 128.77, 128.50, 119.14, 115.69, 115.54, 109.94, 40.69, 34.39. HRMS (ESI) m/z calculated for $C_{25}H_{22}N_2O_2S$ [M+H]$^+$: 415.1451; found [M+H]$^+$: 415.1453.

BDA-58: 2-amino-5-benzyl-N-(4-hydroxyphenethyl)thiophene-3-carboxamide

The product was synthesized according to procedure **step 5** and afforded yellow solid (169 mg, 96%), M.P.= 191 - 197 °C; ^{1}H NMR (500 MHz, $CDCl_3$) δ 7.33 (t, J = 7.4 Hz, 2H), 7.27 (d, J = 13.6 Hz, 2H), 7.22 (d, J = 6.8 Hz, 2H), 7.00 (d, J = 8.5 Hz, 2H), 6.83 – 6.77 (m, 2H), 6.28 (s, 1H), 5.94 (s, 2H), 5.78 (t, J = 6.0 Hz, 1H), 3.89 (s, 2H), 3.53 (q, J = 7.1 Hz, 2H), 2.76 (t, J = 7.2 Hz, 2H). ^{13}C NMR (126 MHz, $CDCl_3$) δ 166.01, 160.06, 154.77, 139.66, 130.44, 129.86, 128.64, 128.58, 126.70, 126.19, 119.81, 115.64, 108.06, 40.79, 35.96, 35.08. HRMS (ESI) m/z calculated for $C_{20}H_{20}N_2O_2S$ [M+H]$^+$: 353.1255; found [M+H]$^+$: 353.1258.

BDA-61: 2-amino-N-(4-hydroxyphenethyl)-5-isopropylthiophene-3-carboxamide

The product was synthesized according to procedure **step 5** and afforded yellow solid (149 mg, 98%), M.P.= 190 - 195 °C; ^{1}H NMR (500 MHz, DMSO- d_6) δ 9.18 (s, 1H), 7.68 (t, J = 5.8 Hz, 1H), 7.09 – 6.98 (m, 4H), 6.77 (d, J = 1.3 Hz, 1H), 6.69 (d, J = 8.5 Hz, 2H), 3.33 – 3.25 (m, 2H), 2.91 – 2.78 (m, 1H), 2.70 – 2.62 (m, 2H), 1.19 (d, J = 6.8 Hz, 6H). ^{13}C NMR (126 MHz, DMSO- d_6) δ 165.90, 159.63, 156.04, 131.64, 130.20, 129.92, 118.62, 118.49, 115.64, 115.50, 106.67, 40.96, 35.32, 29.32, 24.57, 24.49. HRMS (ESI) m/z calculated for $C_{16}H_{20}N_2O_2S$ [M+H]$^+$: 305.1225; found [M+H]$^+$: 305.1228.

BDA-70: 2-(1-cyanocyclopropane-1-carboxamido)-N-(4-hydroxyphenethyl)-5-phenylthiophene-3-carboxamide

The product was synthesized according to procedure **step 6** and purified by column chromatography and afforded as yellow solid (293 mg, 68%), M.P.= 185 - 190 °C; ^{1}H NMR (500 MHz, $CDCl_3$) δ 7.42 (d, J = 6.9 Hz, 2H), 7.34 (t, J = 7.8 Hz, 2H), 7.28 (d, J = 8.2 Hz, 2H), 7.23 (t, J = 7.3 Hz, 1H), 7.14 – 7.10 (m, 2H), 6.87 (s, 1H), 6.23 (s, 2H), 5.85 (t, J = 5.9 Hz, 1H), 3.65 (q, J = 7.0 Hz, 2H), 2.93 (t, J = 7.0 Hz, 2H), 1.89 – 1.85 (m, 2H), 1.84 – 1.80 (m, 2H).^{13}C NMR (126 MHz, $CDCl_3$) δ 166.76, 165.80, 160.39, 148.88, 137.61, 133.89, 129.99, 128.88, 126.70, 125.39,

124.69, 121.30, 118.31, 117.92, 109.63, 40.48, 35.52, 19.97, 13.51. HRMS (ESI) m/z calculated for $C_{24}H_{21}N_3O_3S$ $[M+H]^+$: 432.1372; found $[M+H]^+$: 432.1378.

Scheme 2. Syntheses of compounds BDA-32, BDA-33, BDA-35, BDA-40, BDA-41, BDA-45, BDA-47, BDA-48, BDA-51, BDA-52, BDA-53, BDA-54, BDA-56. [a]

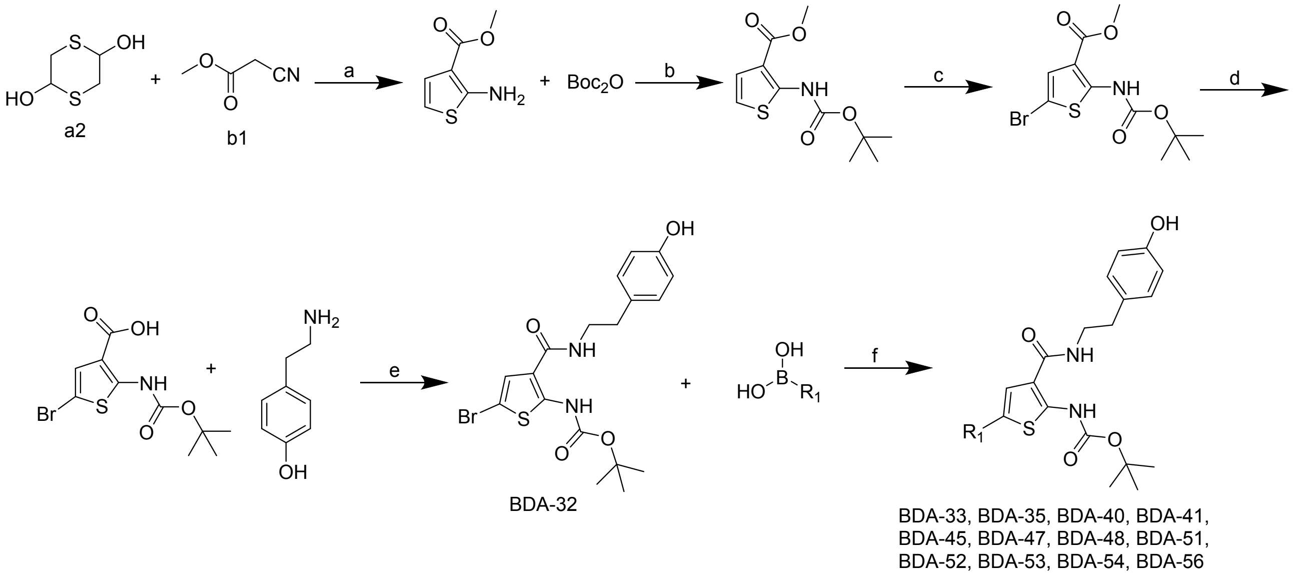

[a]Reagents and conditions: (a) S, Et_3N, MeOH, 40 °C, 12 h; (b) Boc_2O, DMAP, Dioxane, 60 °C, 12 h; (c)NBS, DCM, 0 °C- rt, 1.5 h; (d) NaOH, MeOH : H_2O : THF = 2 : 2 : 1, 80 °C, 6 h; (e) HATU, EtOAc, rt, 12 h; (f) $Pd(PPh_3)_4$, K_3PO_4, DMF: H_2O = 4 : 1, 80 °C, N_2, 16 h.

General Procedure for Preparing the products Using Method B

As Described in Scheme 2:

Step 1':

Methyl 2-cyanoacetate (4.0 g, 40.0 mmol, 1.0 eq), 1,4-dithiane-2,5-diol (3.04 g, 20.0 mmol, 0.5 eq), and triethylamine (1.7 mL, 12.0 mmol, 0.3 eq) in 100 mL methanol are added into a 250 mL round-bottomed flask. The reaction mixture was heated at 40 °C for 12 h. Then the reaction was cooled to room temperature and extracted with DCM (200 mL x 3). The organic layer was dry with $MgSO_4$, filtered and concentrated under reduced pressure. The residue was purified by column chromatography on silica gel using EtOAc–PE (5% → 80%) as an eluent to give final compound (5.1 g, 80%) red solid. M.P.= 177 - 182 °C; ^1H NMR (500 MHz, $CDCl_3$) δ 6.99 (d, J = 5.7 Hz, 1H), 6.21 (d, J = 5.8 Hz, 1H), 5.96 (s, 2H), 3.84 (s, 3H). ^{13}C NMR (126 MHz, $CDCl_3$) δ 165.82, 162.76,

125.81, 107.03, 106.93, 51.00. HRMS (ESI) m/z calculated for $C_6H_7NO_2S$ $[M+H]^+$: 158.0247; found $[M+H]^+$: 158.0249.

Step 2':

To a 50 mL round bottom flask were added dioxane (40 mL), methyl 2-aminothiophene-3-carboxylate (4.7 g, 30.0 mmol, 1.0 eq), Boc_2O (7.2 g, 33.0 mmol, 1.1 eq) and DMAP (388 mg, 3.0 mmol, 0.1 eq) and the reaction mixture was stirred at 60 °C for 12 h. After cooling to room temperature the solvent was removed under reduced pressure, and the residue was purified by column chromatography on silica gel (EtOAc–heptane 5 → 50%) to yield methyl 2-((tert-butoxycarbonyl)amino)thiophene-3-carboxylate (7.3 g, 95%), M.P.= 177 - 180 °C; ^{1}H NMR (500 MHz, $CDCl_3$) δ 7.36 (d, J = 6.6 Hz, 1H), 7.16 (d, J = 5.8 Hz, 1H), 3.84 (s, 3H), 1.42 (s, 9H). ^{13}C NMR (126 MHz, CDC_{l3}) δ 162.25, 150.37, 146.67, 127.45, 127.21, 122.73, 83.31, 51.74, 27.78. HRMS (ESI) m/z calculated for $C_{11}H_{15}NO_4S$ $[M+H]^+$: 258.0739; found $[M+H]^+$: 258.0754.

Step 3':

To a solution of methyl 2-((tert-butoxycarbonyl)amino)thiophene-3-carboxylate (1.6 g, 6.22 mmol, 1.0 eq) in dichloromethane (10 mL) and acetic acid (10 mL) were added NBS (1.33 g, 7.46 mmol, 1.2 eq) at 0 °C for 0.5 h. The reaction mixture was stirred at room temperature for 1 h. Then the reaction mixture was diluted with water and extracted with ethyl acetate (200 mL x 3). The organic layer was washed with saturated $NaHCO_3$ and finally with brine, dried with $MgSO_4$. The solvent was removed on a rotary evaporator, and the residue was purified by column chromatography on silica gel (EtOAc–heptane 10 → 50%) to yield the product (1.56 g, 75%) as red solid. M.P.= 177 - 180 °C; ^{1}H NMR (500 MHz, $CDCl_3$) δ 10.04 (s, 1H), 7.15 (s, 1H), 3.87 (s, 3H), 1.55 (s, 9H). ^{13}C NMR (126 MHz, CDC_{l3}) δ 164.74, 152.15, 151.50, 126.01, 110.71, 102.55, 82.81, 51.75, 28.15. HRMS (ESI) m/z calculated for $C_{11}H_{14}BrNO_4S$ $[M+H]^+$: 335.9871; found $[M+H]^+$: 335.9883.

Step 4':

The methyl 5-bromo-2-((tert-butoxycarbonyl)amino)thiophene-3-carboxylate (740 mg, 2.2 mmol, 1.0 eq) was subsequently subjected to a base hydrolysis at 80 °C for 6 h with a solution of NaOH (594 mg, 14.85 mmol, 6.75 eq) in 30 mL of a solvent mixture (MeOH–H_2O–THF = 2:2:1). The reaction was cooled to room temperature and the organic solvents were removed in vacuo. The

aqueous layer was acidified with 5% HCl to give a precipitate, which was filtered to afford the compound (465 mg, 65%) as red solid, M.P.= 198 - 203 °C; ^{1}H NMR (500 MHz, $CDCl_3$) δ 9.85 (s, 1H), 7.21 (s, 1H), 1.58 (s, 9H). ^{13}C NMR (126 MHz, $CDCl_3$) δ 168.05, 153.34, 148.42, 128.60, 126.25, 109.82, 84.02, 28.17. HRMS (ESI) m/z calculated for $C_{10}H_{12}BrNO_4S$ $[M+H]^+$: 321.9731; found $[M+H]^+$: 321.9745.

Step 5':

To a solution of compound 5-bromo-2-((tert-butoxycarbonyl)amino)thiophene-3-carboxylic acid (607 mg, 2.0 mmol, 1.0 eq) and DIPEA (1.2 mL, 6.0 mmol, 3.0 eq) in ethyl acetate (20 mL) was added HATU (420 mg, 1.1 mmol, 1.1eq) and 4-(2-aminoethyl)phenol (302 mg, 2.2 mmol, 1.2 eq). The reaction mixture was stirred at room temperature for 12 h. The mixture was washed with water and extracted with EtOAc. The organic layer was separated, washed with water and brine, dried over $MgSO_4$, filtered and concentrated under reduced pressure. The residue was purified by column chromatography on silica gel using EtOAc–PE (10% → 75%) as an eluent to give the product **BDA-32** (318 mg, 64%) as yellow solid, M.P.= 192 - 196 °C; ^{1}H NMR (500 MHz, $CDCl_3$) δ 10.93 (s, 1H), 7.15 – 7.04 (m, 2H), 6.88 – 6.76 (m, 2H), 6.73 (s, 1H), 5.80 – 5.73 (m, 1H), 5.00 (s, 1H), 3.65 – 3.58 (m, 2H), 2.84 (t, J = 6.9 Hz, 2H), 1.54 (s, 9H). ^{13}C NMR (126 MHz, $CDCl_3$) δ 164.18, 154.43, 152.50, 149.51, 130.61, 129.91, 122.82, 115.68, 112.65, 103.17, 82.30, 40.83, 34.84, 28.19. HRMS (ESI) m/z calculated for $C_{18}H_{21}BrN_2O_4S$ $[M+H]^+$: 441.0427; found $[M+H]^+$:441.0434.

Step 6':

The compound tert-butyl (5-bromo-3-((4-hydroxyphenethyl)carbamoyl) thiophen-2-yl)carbamate (136 mg, 0.32 mmol, 1.0 eq), benzo[d][1,3]dioxol-5-ylboronic acid (64 mg, 0.38 mmol, 1.1 eq) and K_3PO_4 (424 mg, 2.0 mmol, 6.25 eq) were dissolved in 4:1 DMF /H_2O under the atmosphere of N_2. Then the $Pd(PPh_3)_4$ (12 mg, 0.001 mmol, 0.03 eq) was added and the reaction mixture was heated at 80 °C for 16 h. The solution was cooled and diluted with EtOAc (30 mL), extracted with EtOAc(100 mL x 3), then washed with water and brine. The organic layer was dried over $MgSO_4$, filtered and concentrated under reduced pressure. The residue was purified by column chromatography on silica gel using MeOH /DCM (1% → 10%) as an eluent to give the final product **BDA-41** (105 mg, 68%) as yellow solid, M.P.= 198 - 202 °C; ^{1}H NMR (500 MHz, DMSO- d_6) δ 11.26 (s, 1H), 9.20 (s, 1H), 8.38 (t, J = 5.5 Hz, 1H), 7.68 (s, 1H), 7.10 (d, J = 1.9 Hz,

1H), 7.06 – 7.00 (m, 3H), 6.97 (d, J = 8.2 Hz, 1H), 6.70 (d, J = 8.5 Hz, 2H), 6.07 (s, 2H), 3.44 – 3.37 (m, 2H), 2.73 (t, J = 7.6 Hz, 2H), 1.51 (s, 9H). ^{13}C NMR (126 MHz, DMSO- d_6) δ 165.09, 156.17, 151.83, 148.56, 147.19, 146.30, 131.48, 129.97, 129.78, 128.25, 118.79, 115.71, 115.56, 114.92, 109.40, 105.52, 101.79, 82.04, 41.29, 34.82, 28.35, 28.18. HRMS (ESI) m/z calculated for $C_{25}H_{26}N_2O_6S$ $[M+H]^+$: 483.1507; found $[M+H]^+$: 483.1532.

The compounds **BDA-33, BDA-35, BDA-40, BDA-45, BDA-47, BDA-48, BDA-51, BDA-52, BDA-53, BDA-54, BDA-56** and **BDA-69** are synthesized according to general procedure 6'.

BDA-33: Tert-butyl (5-(2,3-dihydrobenzo[b][1,4]dioxin-6-yl)-3-((4 hydroxyphenethyl) carbamoyl) thiophen-2-yl)carbamate.

The compounds **BDA-33** was synthesized according to general procedure 6', and yielded final compound (184 mg, 74%) brown solid, M.P.= 195 - 200 °C; ^1H NMR (500 MHz, $CDCl_3$) δ 10.91 (s, 1H), 7.16 – 7.09 (m, 2H), 7.04 (d, J = 2.2 Hz, 1H), 7.00 (dd, J = 8.4, 2.2 Hz, 1H), 6.88 – 6.81 (m, 3H), 6.79 (s, 1H), 5.92 (t, J = 5.8 Hz, 1H), 5.18 (s, 1H), 4.29 (s, 4H), 3.64 (q, J = 6.9 Hz, 2H), 2.87 (t, J = 6.9 Hz, 2H), 1.56 (s, 9H). ^{13}C NMR (126 MHz, $CDCl_3$) δ 187.40, 171.36, 165.22, 154.43, 143.79, 143.20, 130.78, 129.98, 123.59, 118.60, 117.74, 115.66, 115.22, 114.13, 113.54, 97.19, 64.44, 53.44, 40.80, 34.94, 28.26. HRMS (ESI) m/z calculated for $C_{26}H_{28}N_2O_6S$ $[M+H]^+$: 497.1712; found $[M+H]^+$: 497.1715.

BDA-35: Tert-butyl (5-(2-aminopyrimidin-5-yl)-3-((4-hydroxyphenethyl)carbamoyl) thiophen-2-yl)carbamate.

The compounds **BDA-35** was synthesized according to general procedure 6', and yielded final compound (148 mg, 65%) yellow solid, M.P.= 198 - 203 °C; ^1H NMR (500 MHz, DMSO- d_6) δ 11.26 (s, 1H), 9.19 (s, 1H), 8.43 (s, 2H), 8.32 (s, 2H), 7.61 (s, 1H), 7.04 (d, J = 8.5 Hz, 2H), 6.88 (s, 1H), 6.70 (d, J = 8.5 Hz, 2H), 3.44 – 3.36 (m, 2H), 2.72 (t, J = 7.6 Hz, 2H), 1.51 (s, 9H). ^{13}C NMR (126 MHz, DMSO- d_6) δ 165.01, 163.09, 156.17, 154.86, 154.79, 151.85, 146.15, 129.96, 129.77, 126.55, 117.33, 115.63, 114.91, 79.68, 79.62, 41.29, 34.82, 28.32, 28.21.HRMS (ESI) m/z calculated for $C_{22}H_{25}N_5O_4S$ $[M+H]^+$: 456.1622; found $[M+H]^+$: 456.1625.

BDA-40: Tert-butyl (5-(5-chloropyridin-3-yl)-3-((4-hydroxyphenethyl)carbamoyl) thiophen-2-yl)carbamate.

The compounds **BDA-40** was synthesized according to general procedure 6', and yielded final compound (201 mg, 85%) yellow solid, M.P.= 194 - 199 °C; ^{1}H NMR (500 MHz, $CDCl_3$) δ 10.99 (s, 1H), 8.48 (s, 1H), 8.31 (s, 1H), 7.71 (t, J = 2.1 Hz, 1H), 7.11 (s, 1H), 7.08 (d, J = 8.5 Hz, 2H), 6.84 (d, J = 8.5 Hz, 2H), 6.52 (t, J = 5.9 Hz, 1H), 3.62 (q, J = 6.6 Hz, 2H), 2.85 (t, J = 6.7 Hz, 2H), 1.55 (s, 9H). ^{13}C NMR (126 MHz, $CDCl_3$) δ 164.84, 155.20, 152.39, 149.85, 145.60, 142.81, 132.76, 132.19, 131.67, 130.21, 130.12, 126.37, 119.29, 115.76, 114.39, 82.63, 40.83, 34.74, 28.19. HRMS (ESI) m/z calculated for $C_{23}H_{24}ClN_3O_4S$ $[M+H]^+$: 474.1223; found $[M+H]^+$: 474.1226.

BDA-45: Tert-butyl (5-(4-formylphenyl)-3-((4-hydroxyphenethyl)carbamoyl) thiophen- 2-yl)carbamate.

The compounds **BDA-45** was synthesized according to general procedure 6', and yielded final compound (177 mg, 76%) yellow solid, M.P.= 192 - 197 °C; ^{1}H NMR (500 MHz, DMSO- d_6) δ 11.38 (s, 1H), 9.98 (s, 1H), 9.20 (s, 1H), 8.52 (t, J = 5.7 Hz, 1H), 8.07 (s, 1H), 7.95 (d, J = 8.4 Hz, 2H), 7.75 (d, J = 8.4 Hz, 2H), 7.05 (d, J = 8.4 Hz, 2H), 6.70 (d, J = 8.5 Hz, 2H), 3.48 – 3.38 (m, 2H), 2.74 (t, J = 7.6 Hz, 2H), 1.52 (s, 9H). ^{13}C NMR (126 MHz, DMSO- d_6) δ 192.55, 192.50, 164.91, 156.19, 151.83, 148.71, 139.72, 134.93, 131.05, 130.95, 130.00, 129.93, 129.82, 129.76, 125.12, 124.97, 122.22, 115.71, 115.60, 115.57, 82.39, 41.33, 34.79, 28.28.HRMS (ESI) m/z calculated for $C_{25}H_{26}N_2O_5S$ $[M+H]^+$: 467.1633; found $[M+H]^+$: 467.1637.

BDA-47: Tert-butyl (3-((4-hydroxyphenethyl)carbamoyl)-5-(3-morpholinophenyl) thiophen-2-yl)carbamate.

The compounds **BDA-47** was synthesized according to general procedure 6', and yielded final compound (160 mg, 61%) yellow solid, M.P.= 194 - 199 °C; ^{1}H NMR (500 MHz, DMSO- d_6) δ 11.31 (s, 1H), 9.21 (s, 1H), 8.46 (t, J = 5.8 Hz, 1H), 7.79 (s, 1H), 7.28 (t, J = 8.0 Hz, 1H), 7.07 – 7.02 (m, 3H), 7.00 (d, J = 8.4 Hz, 1H), 6.90 (dd, J = 7.9, 2.4 Hz, 1H), 6.70 (d, J = 8.5 Hz, 2H), 3.80 – 3.75 (m, 4H), 3.41 (dd, J = 14.7, 5.4 Hz, 2H), 3.20 – 3.15 (m, 4H), 2.73 (t, J = 7.5 Hz, 2H), 1.51 (s, 9H). ^{13}C NMR (126 MHz, DMSO- d_6) δ 165.12, 164.03, 156.18, 152.12, 146.80, 134.64, 132.09, 130.22, 130.02, 129.78, 119.07, 115.71, 115.57, 115.00, 82.07, 66.55, 48.77, 40.56, 28.34, 28.18. HRMS (ESI) m/z calculated for $C_{28}H_{33}N_3O_5S$ $[M+H]^+$: 524.2113; found $[M+H]^+$: 524.2115.

BDA-48: Tert-butyl (5-(3-formylphenyl)-3-((4-hydroxyphenethyl)carbamoyl) thiophen-2-yl)carbamate.

The compounds **BDA-48** was synthesized according to general procedure 6’, and yielded final compound (173 mg, 74%) yellow solid, M.P.= 191 - 197 °C; ^{1}H NMR (500 MHz, $CDCl_3$) δ 10.89 (s, 1H), 10.05 (s, 1H), 8.07 (t, J = 1.8 Hz, 1H), 7.79 – 7.73 (m, 2H), 7.56 (t, J = 7.6 Hz, 1H), 7.15 (d, J = 8.5 Hz, 2H), 7.00 (s, 1H), 6.96 – 6.90 (m, 2H), 5.87 (t, J = 6.1 Hz, 1H), 3.65 (q, J = 6.8 Hz, 2H), 2.90 (t, J = 6.8 Hz, 2H), 1.57 (s, 9H). ^{13}C NMR (126 MHz, $CDCl_3$) δ 192.63, 164.83, 154.97, 152.42, 148.91, 136.74, 134.87, 130.81, 130.74, 130.30, 130.18, 129.83, 129.41, 124.55, 117.66, 115.87, 114.06, 82.29, 40.45, 34.75, 28.23. HRMS (ESI) m/z calculated for $C_{25}H_{26}N_2O_5S$ $[M+H]^+$: 467.1632; found $[M+H]^+$: 467.1635.

BDA-51: Tert-butyl (5-(3-aminophenyl)-3-((4-hydroxyphenethyl)carbamoyl) thiophen-2-yl)carbamate.

The compounds **BDA-51** was synthesized according to general procedure 6’, and yielded final compound (154 mg, 68%) yellow solid, M.P.= 194 - 199 °C; ^{1}H NMR (500 MHz, DMSO- d_6) δ 11.29 (s, 1H), 9.18 (s, 1H), 8.47 (t, J = 5.8 Hz, 1H), 8.32 (s, 1H), 7.68 (s, 1H), 7.08 – 7.05 (m, 1H), 7.04 (d, J = 8.5 Hz, 2H), 6.77 (t, J = 2.0 Hz, 1H), 6.70 (d, J = 8.4 Hz, 2H), 6.50 (dd, J = 8.0, 1.3 Hz, 1H), 5.21 (s, 2H), 3.46 – 3.36 (m, 2H), 2.73 (t, J = 7.6 Hz, 2H), 1.51 (s, 9H). ^{13}C NMR (126 MHz, DMSO- d_6) δ 165.18, 156.16, 151.85, 149.75, 149.54, 146.42, 134.43, 132.55, 129.98, 129.96, 129.83, 118.65, 115.69, 114.95, 112.64, 110.42, 79.69, 41.26, 34.79, 28.37. HRMS (ESI) m/z calculated for $C_{24}H_{27}N_3O_4S$ $[M+H]^+$: 454.1724; found $[M+H]^+$: 454.1728.

BDA-52: Tert-butyl (3-((4-hydroxyphenethyl)carbamoyl)-5-(p-tolyl)thiophen-2-yl)carbamate

The compounds **BDA-52** was synthesized according to general procedure 6’, and yielded final compound (267 mg, 61%) yellow solid, M.P.= 191 - 197 °C; ^{1}H NMR (500 MHz, $CDCl_3$) δ 10.94 (s, 1H), 7.43 (d, *J* = 8.2 Hz, 2H), 7.18 (d, *J* = 8.0 Hz, 2H), 7.15 – 7.09 (m, 2H), 6.89 (s, 1H), 6.86 – 6.80 (m, 2H), 5.94 (t, *J* = 5.8 Hz, 1H), 3.69 – 3.60 (m, 2H), 2.88 (t, *J* = 7.0 Hz, 2H), 2.37 (s, 3H), 1.56 (s, 9H). ^{13}C NMR (126 MHz, $CDCl_3$) δ 165.26, 154.38, 148.14, 137.26, 130.91, 130.84, 129.99, 129.65, 125.11, 115.63, 115.43, 113.58, 81.94, 40.87, 34.97, 28.26, 21.16. HRMS (ESI) m/z calculated for $C_{25}H_{29}N_2O_4S$ $[M+H]^+$: 453.1812; found $[M+H]^+$: 453.1817.

BDA-53: Tert-butyl (5-(3-cyanophenyl)-3-((4-hydroxyphenethyl)carbamoyl) thiophen-2-yl)carbamate.

The compounds **BDA-53** was synthesized according to general procedure 6', and yielded final compound (155 mg, 67%) yellow solid, M.P.= 193 - 198 °C; ^{1}H NMR (500 MHz, DMSO- d_6) δ 11.31 (s, 1H), 9.20 (s, 1H), 8.41 (t, J = 5.7 Hz, 1H), 7.99 (s, 1H), 7.95 (t, J = 1.8 Hz, 1H), 7.83 (dt, J = 8.0, 1.5 Hz, 1H), 7.73 (dt, J = 7.7, 1.4 Hz, 1H), 7.62 (t, J = 7.8 Hz, 1H), 7.08 – 7.01 (m, 2H), 6.74 – 6.66 (m, 2H), 3.46 – 3.38 (m, 2H), 2.74 (t, J = 7.6 Hz, 2H), 1.52 (s, 9H). ^{13}C NMR (126 MHz, DMSO- d_6) δ 164.90, 156.19, 151.83, 148.05, 135.28, 130.88, 130.67, 129.97, 129.74, 129.43, 129.25, 128.88, 128.07, 127.93, 121.58, 121.46, 118.92, 115.71, 115.59, 115.26, 112.81, 82.34, 41.32, 34.80, 28.31. HRMS (ESI) m/z calculated for $C_{25}H_{25}N_3O_4S$ $[M+H]^+$: 464.1641; found $[M+H]^+$: 464.1647.

BDA-54: Tert-butyl (5-(4-fluorophenyl)-3-((4-hydroxyphenethyl)carbamoyl)thiophen –2-yl)carbamate.

The compounds **BDA-54** was synthesized according to general procedure 6', and yielded final compound (171 mg, 75%) yellow solid, M.P.= 191 - 196 °C; ^{1}H NMR (500 MHz, $CDCl_3$) δ 10.90 (s, 1H), 7.40 (dd, J = 8.8, 5.2 Hz, 2H), 7.05 (d, J = 8.5 Hz, 2H), 7.00 (t, J = 8.6 Hz, 2H), 6.92 (s, 1H), 6.83 (d, J = 8.4 Hz, 2H), 6.25 (t, J = 5.9 Hz, 1H), 3.61 (q, J = 6.9 Hz, 2H), 2.83 (t, J = 7.1 Hz, 2H), 1.53 (s, 9H). ^{13}C NMR (126 MHz, $CDCl_3$) δ 165.39, 163.12, 161.15, 154.79, 152.47, 148.29, 131.85, 130.31, 129.90, 129.88, 126.83, 126.77, 116.32, 115.99, 115.82, 115.73, 113.75, 82.29, 41.06, 34.87, 28.21. HRMS (ESI) m/z calculated for $C_{24}H_{25}FN_2O_4S$ $[M+H]^+$: 457.1513; found $[M+H]^+$: 457.1517.

BDA-56: Tert-butyl (5-(3,4-dichlorophenyl)-3-((4-hydroxyphenethyl)carbamoyl) thiophen-2-yl)carbamate.

The compounds **BDA-56** was synthesized according to general procedure 6', and yielded final compound (181 mg, 71%) yellow solid, M.P.= 192 - 197 °C; ^{1}H NMR (500 MHz, $CDCl_3$) δ 10.96 (s, 1H), 7.61 (d, J = 2.2 Hz, 1H), 7.42 (d, J = 8.4 Hz, 1H), 7.32 (dd, J = 8.4, 2.1 Hz, 1H), 7.13 (d, J = 8.5 Hz, 2H), 6.95 (s, 1H), 6.84 (d, J = 8.5 Hz, 2H), 5.95 (t, J = 6.0 Hz, 1H), 3.71 – 3.55 (m, 2H), 2.88 (t, J = 6.9 Hz, 2H), 1.56 (s, 9H). ^{13}C NMR (126 MHz, $CDCl_3$) δ 164.97, 154.46, 152.43, 149.19, 133.82, 133.11, 130.97, 130.83, 130.68, 129.97, 126.70, 124.19, 117.29, 115.66, 113.82,

82.36, 40.94, 34.92, 28.23. HRMS (ESI) m/z calculated for $C_{24}H_{24}Cl_2N_2O_4S$ $[M+H]^+$: 507.0825; found $[M+H]^+$: 507.0828.

BDA-69: Methyl 2-((tert-butoxycarbonyl)amino)-5-(2,3-dihydrobenzo[b][1,4]dioxin-6-yl) thiophene-3-carboxylate

The compounds **BDA-69** was synthesized according to General procedure 6', and yielded final compound (294 mg, 75%) yellow solid, M.P.= 187 - 192 °C; ^{1}H NMR (500 MHz, $CDCl_3$) δ 10.04 (s, 1H), 7.24 (s, 1H), 7.09 (d, *J* = 2.4 Hz, 1H), 7.05 (dd, *J* = 8.4, 2.3 Hz, 1H), 6.86 (d, *J* = 8.4 Hz, 1H), 4.28 (s, 4H), 3.90 (s, 3H), 1.57 (s, 9H). ^{13}C NMR (126 MHz, $CDCl_3$) δ 165.74, 152.09, 149.96, 143.80, 143.16, 132.17, 127.45, 118.57, 118.49, 117.67, 114.18, 111.63, 82.39, 64.45, 64.40, 51.61, 28.22. HRMS (ESI) m/z calculated for $C_{19}H_{22}NO_6S$ $[M+H]^+$: 392.1125; found $[M+H]^+$: 392.1129.

Scheme 3. Syntheses of compounds BDA-44.[a]

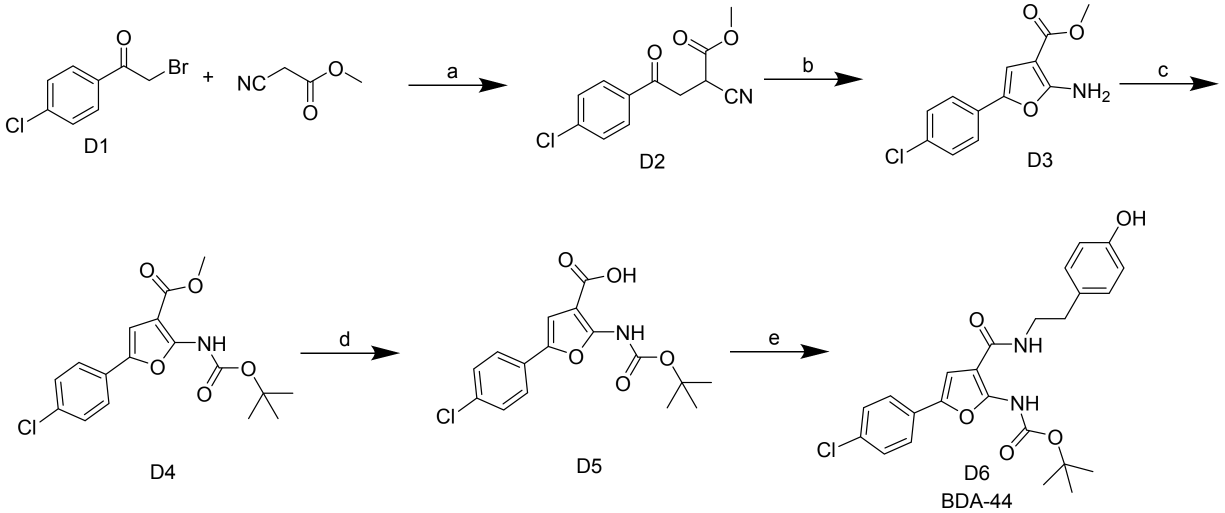

[a]Reagents and conditions: (a) Et_2HN, DMF, N_2, 40 °C, 2 h; (b) TFA, DCM, rt, 16 h; (c) Boc_2O, DMAP, Dioxane, 60 °C, 4 h; (d) NaOH, MeOH : H_2O : THF = 2 : 2 : 1, 80 °C, 6 h; (e) HATU, DCM, rt, 12 h.

General Procedure for Preparing the products Using Method C

As Described in Scheme 3.

Step 1":

To a solution of 2-bromo-1-(4-chlorophenyl) ethan-1-one (2.33 g, 10.0 mmol, 1.0 eq) and methyl 2-cyanoacetate (0.9 mL, 10.0 mmol, 1.0 eq) in dry DMF (20 mL) was added diethylamine (2.0 mL, 20.0 mmol, 2.0 eq). The reaction mixture was stirred at room temperature for 2 h under N_2

atmosphere. The mixture was then diluted with DCM (100 mL), poured into water and washed with 2N HCl. The organic phase was extracted with DCM (100 mL x 3), then washed with water and brine. The organic layer was dried over $MgSO_4$, filtered and concentrated under reduced pressure. The residue was purified by column chromatography on silica gel using EtOAc–PE (10% → 75%) as an eluent to give the final product (1.7 g, 68%) as brown solid.

Step 2”:

Trifluoroacetic acid (15 mL) was added in one portion to intermediate D2 (1.0 g, 4.0 mmol, 1.0 eq) in DCM (15 mL) at room temperature. The reaction was stirred for 16 h and the solvents removed under vacuum. The residue was extracted with EtOAc (100 mL x 3), then washed with water and brine. The organic layer was dried over $MgSO_4$, filtered and concentrated under reduced pressure. The residue was purified by column chromatography on silica gel using EtOAc–PE (30% → 100%) as an eluent to give the final product (0.8 g, 80%) as brown solid.

Step 3”:

To a solution of intermediate D3 (0.75 g, 3.0 mmol, 1.0 eq) in dioxane (40 mL), Boc_2O (0.72 g, 3.3 mmol, 1.1 eq) and DMAP (39 mg, 0.33 mmol, 0.1 eq) were added. The reaction mixture was stirred at 60 °C for 4 h. After cooling to room temperature the solvent was removed under reduced pressure, and the residue was purified by column chromatography on silica gel (EtOAc–heptane 15 → 65%) to yield product D4 (1.03 g, 98%).

Step 4”:

The intermediate D4 (0.7 g, 2.0 mmol, 1.0 eq) was subsequently subjected to a base hydrolysis at 80 °C for 6 h with a solution of NaOH (480 mg, 12.0 mmol, 6.0 eq) in 20 mL of a solvent mixture (MeOH–H_2O–THF = 2:2:1). The reaction was cooled to room temperature and the organic solvents were removed under vacuum. The aqueous layer was acidified with 5% HCl to give a precipitate, which was filtered to afford the compound (540 mg, 80%) as red solid.

Step 5”:

To a solution of intermediate D5 (337 mg, 1.0 mmol, 1.0 eq) and DIPEA (0.6 mL, 3.0 mmol, 3.0 eq) in DCM (10 mL) was added HATU (420 mg, 1.1 mmol, 1.1eq) and 4-(2-aminoethyl)phenol

(165 mg, 1.2 mmol, 1.2 eq). The reaction mixture was stirred at room temperature for 12 h. The mixture was washed with water and extracted with EtOAc. The organic layer was separated, washed with water and brine, dried over $MgSO_4$, filtered and concentrated under reduced pressure. The residue was purified by column chromatography on silica gel using EtOAc–PE (10% → 85%) as an eluent to give the product **BDA-44** (351 mg, 75%) as yellow solid, M.P.= 190 - 195 °C; ^{1}H NMR (500 MHz, DMSO- d_6) δ 9.62 (s, 1H), 9.20 (s, 1H), 8.17 (t, J = 5.9 Hz, 1H), 7.59 (d, J = 8.7 Hz, 2H), 7.52 (d, J = 8.7 Hz, 2H), 7.29 (s, 1H), 7.03 (d, J = 8.5 Hz, 2H), 6.69 (d, J = 8.5 Hz, 2H), 3.36 (s, 2H), 2.70 (t, J = 7.5 Hz, 2H), 1.48 (s, 9H). ^{13}C NMR (126 MHz, DMSO- d_6) δ 162.77, 156.15, 151.32, 148.80, 145.72, 132.29, 129.95, 129.82, 128.76, 125.02, 115.58, 106.23, 105.55, 81.31, 34.89, 28.37, 28.20. HRMS (ESI) m/z calculated for $C_{24}H_{25}ClN_2O_5$ $[M+H]^+$: 457.1525; found $[M+H]^+$: 457.1528.

Scheme 4. Syntheses of compounds BDA-36, BDA-64, BDA-68.[a]

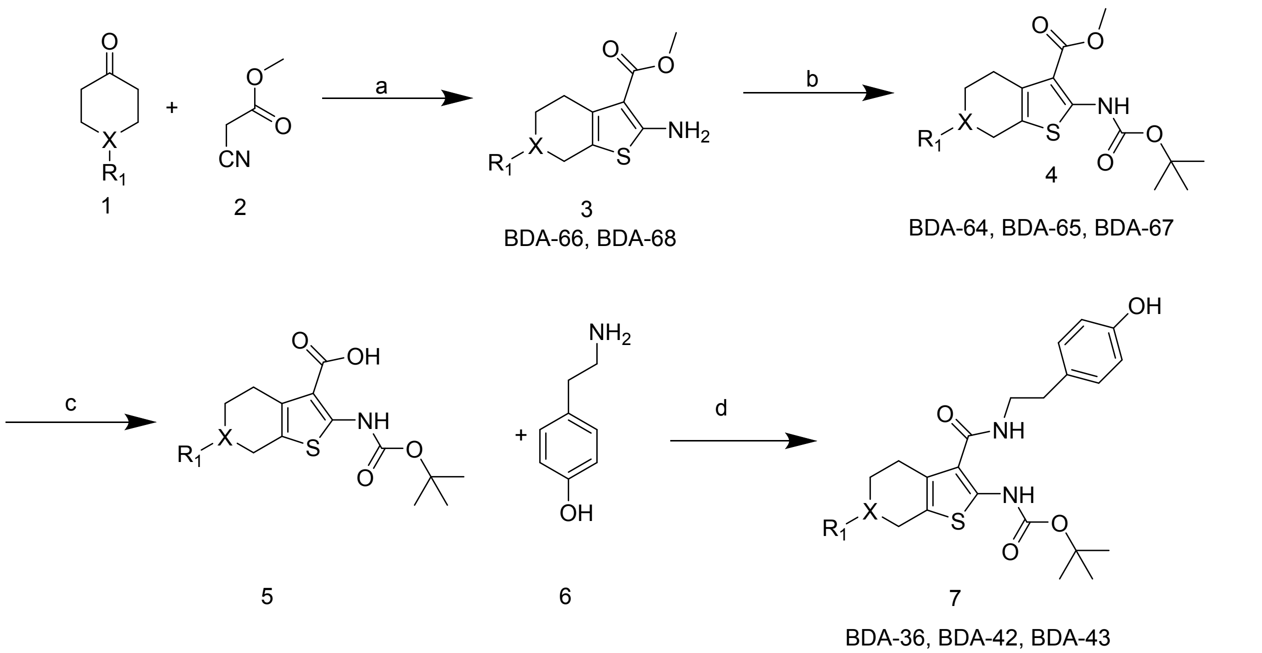

[a]Reagents and conditions: (a) S, Et_2HN, MeOH, 25 °C, 24 h; (b) Boc_2O, DMAP, Dioxane, 60 °C, 4 h; (c) NaOH, MeOH : H_2O : THF = 2 : 2 : 1, 80 °C, 6 h; (d) HATU, DCM, rt, 12 h;

General Procedure for Preparing the Products Using Method D

As Described in Scheme 4.

Step 1''':

A 100 mL round bottom flask was charged with 1-benzylpiperidin-4-one (**1c**, 1.89 g, 10 mmol, 1.0 eq), methyl 2-cyanoacetate (**2a**, 0.97 mL (1.09 g), 11 mmol, 1.1 eq), sulfur (385 mg, 12 mmol, 1.2 eq), and diethylamine (0.52 mL (366 mg), 5.0 mmol, 0.5 eq) in methanol (20 mL). The reaction is heated 25 °C in an oil bath for 24 h. Then, the reaction was cooled down to room temperature. A batch of 120 mL ice water was poured into the mixture to yield a precipitate which was filtered and washed with cold ethanol to obtain 8.1 g (68%) of the title compound **3** as light yellow powder.

BDA-66: Methyl 2-amino-6-phenyl-4,5,6,7-tetrahydrobenzo[b]thiophene-3 carboxylate.
The compounds **BDA-66** was synthesized according to step **1**, and yielded final compound (488 mg, 85%) yellow solid, M.P.= 187 - 192 °C; ^{1}H NMR (500 MHz, $CDCl_3$) δ 7.39 – 7.31 (m, 2H), 7.30 – 7.23 (m, 3H), 5.99 (s, 2H), 3.83 (s, 3H), 3.08 – 2.94 (m, 2H), 2.86 – 2.64 (m, 3H), 2.18 – 2.07 (m, 1H), 2.00 – 1.84 (m, 1H). ^{13}C NMR (126 MHz, $CDCl_3$) δ 166.46, 162.08, 146.08, 132.23, 128.48, 126.90, 126.32, 117.05, 105.47, 50.65, 40.88, 32.39, 30.08, 27.22. HRMS (ESI) m/z calculated for $C_{16}H_{17}NO_2S$ $[M+H]^+$: 288.1024; found $[M+H]^+$: 288.1026.

BDA-68: Methyl 2-amino-6-benzyl-4,5,6,7-tetrahydrothieno[2,3-c]pyridine-3-carboxylate.
The compounds **BDA-68** was synthesized according to step **1**, and yielded final compound (544 mg, 90%) yellow solid, M.P.= 189 - 195 °C; ^{1}H NMR (500 MHz, $CDCl_3$) δ 7.42 – 7.38 (m, 2H), 7.37 – 7.33 (m, 2H), 7.31 – 7.29 (m, 1H), 5.95 (s, 2H), 3.81 (s, 3H), 3.71 (s, 2H), 3.44 (t, J = 2.0 Hz, 2H), 2.84 (td, J = 5.4, 2.6 Hz, 2H), 2.78 (t, J = 5.7 Hz, 2H). ^{13}C NMR (126 MHz, $CDCl_3$) δ 166.32, 162.16, 138.24, 131.07, 129.12, 128.33, 127.19, 114.96, 61.98, 51.28, 50.64, 50.24, 27.18. HRMS (ESI) m/z calculated for $C_{16}H_{18}N_2O_2S$ $[M+H]^+$: 303.1132; found $[M+H]^+$: 303.1136.

Step 2, Step 3, Step 4 according to the method **A**.

BDA-64: Methyl 6-benzyl-2-((tert-butoxycarbonyl)amino)-4,5,6,7 tetrahydrothieno [2,3-c]pyridine-3-carboxylate.
The compounds **BDA-64** was synthesized according to step **2**, and yielded final compound (765 mg, 95%) yellow solid, M.P.= 185 - 190 °C; ^{1}H NMR (500 MHz, $CDCl_3$) δ 10.26 (s, 1H), 7.42 – 7.33 (m, 4H), 7.32 – 7.29 (m, 1H), 3.86 (s, 3H), 3.72 (s, 2H), 3.57 (s, 2H), 2.86 (d, J = 7.4 Hz, 2H), 2.78 (t, J = 6.0 Hz, 2H), 1.54 (s, 9H). ^{13}C NMR (126 MHz, $CDCl_3$) δ 166.55, 150.53, 138.13,

129.62, 129.12, 128.36, 128.10, 127.25, 122.69, 109.48, 82.03, 61.83, 51.32, 51.25, 49.96, 28.23, 26.69. HRMS (ESI) m/z calculated for $C_{21}H_{26}N_2O_4S$ $[M+H]^+$: 403.1621; found $[M+H]^+$: 403.1624.

BDA-65: Methyl 2-((tert-butoxycarbonyl)amino)-6-phenyl-4,5,6,7-tetrahydrobenzo[b] thiophene-3-carboxylate.

The compounds **BDA-65** was synthesized according to step **2**, and yielded final compound (743 mg, 96%) yellow solid, M.P.= 184 - 190 °C; ^{1}H NMR (500 MHz, $CDCl_3$) δ 10.33 (s, 1H), 7.37 – 7.33 (m, 2H), 7.30 (d, J = 1.6 Hz, 2H), 7.28 – 7.23 (m, 1H), 3.88 (s, 3H), 3.10 – 2.97 (m, 2H), 2.93 (dd, J = 16.2, 5.3 Hz, 1H), 2.84 – 2.73 (m, 2H), 2.17 – 2.09 (m, 1H), 2.01 – 1.85 (m, 1H), 1.55 (s, 9H). ^{13}C NMR (126 MHz, $CDCl_3$) δ 162.76, 150.98, 145.60, 143.81, 134.27, 133.70, 128.54, 126.87, 126.45, 126.33, 83.27, 51.39, 40.37, 32.72, 29.78, 27.88, 26.29. HRMS (ESI) m/z calculated for $C_{21}H_{25}NO_4S$ $[M+H]^+$: 388.1521; found $[M+H]^+$: 388.1527.

BDA-67: Methyl 2-((tert-butoxycarbonyl)amino)-4,5,6,7-tetrahydrobenzo[b] thiophene-3-carboxylate.

The compounds **BDA-67** was synthesized according to step **2**, and yielded final compound (610 mg, 98%) yellow solid, M.P.= 183 - 189 °C; ^{1}H NMR (500 MHz, $CDCl_3$) δ 10.29 (s, 1H), 3.85 (s, 3H), 2.78 – 2.71 (m, 2H), 2.63 – 2.58 (m, 2H), 1.77 (t, *J* = 7.9 Hz, 4H), 1.53 (s, 9H). ^{13}C NMR (126 MHz, $CDCl_3$) δ 166.74, 166.34, 152.15, 150.00, 149.86, 131.01, 130.93, 125.08, 110.06, 109.88, 81.76, 60.18, 51.16, 28.21, 26.44, 26.35, 24.30, 24.28, 23.01, 22.86, 22.81, 14.31. HRMS (ESI) m/z calculated for $C_{21}H_{25}NO_4S$ $[M+H]^+$: 312.1215; found $[M+H]^+$: 312.1218.

BDA-36: Tert-butyl (6-benzyl-3-((4-hydroxyphenethyl)carbamoyl)-4,5,6,7 tetrahydrothieno[2,3-c]pyridin-2-yl)carbamate.

The compounds **BDA-36** was synthesized according to step **4**, and yielded final compound (200 mg, 79%) yellow solid, M.P.= 189 - 195 °C; ^{1}H NMR (500 MHz, $CDCl_3$) δ 11.00 (s, 1H), 7.38 (d, J = 4.7 Hz, 4H), 7.35 – 7.31 (m, 1H), 7.10 – 7.05 (m, 2H), 6.79 – 6.75 (m, 2H), 5.71 (t, J = 5.4 Hz, 1H), 3.70 (s, 2H), 3.68 – 3.60 (m, 2H), 3.58 (s, 2H), 2.84 (t, J = 6.7 Hz, 2H), 2.74 – 2.66 (m, 2H), 2.46 – 2.37 (m, 2H), 1.52 (s, 9H). ^{13}C NMR (126 MHz, $CDCl_3$) δ 165.97, 154.59, 147.96, 137.70, 130.49, 129.98, 129.23, 128.45, 127.43, 125.88, 123.35, 115.70, 111.96, 61.69, 51.41, 49.51, 40.57,

34.40, 29.72, 28.27, 26.54. HRMS (ESI) m/z calculated for $C_{28}H_{33}N_3O_4S$ $[M+H]^+$: 508.2236; found $[M+H]^+$: 508.2237.

BDA-42: Tert-butyl (3-((4-hydroxyphenethyl)carbamoyl)-6-phenyl-4,5,6,7 tetrahydrobenzo[b]thiophen-2-yl)carbamate.

The compounds **BDA-42** was synthesized according to step **4**, and yielded final compound (182 mg, 74%) yellow solid, M.P.= 185 - 190 °C; ^1H NMR (500 MHz, $CDCl_3$) δ 11.07 (s, 1H), 10.28 (s, 1H), 7.37 (t, J = 7.6 Hz, 1H), 7.34 – 7.28 (m, 5H), 7.23 (dt, J = 24.4, 7.3 Hz, 2H), 7.16 – 7.13 (m, 1H), 5.85 (t, J = 5.8 Hz, 1H), 3.82 – 3.64 (m, 1H), 3.16 (d, J = 22.5 Hz, 1H), 3.08 – 2.86 (m, 5H), 2.86 – 2.75 (m, 1H), 2.52 (d, J = 15.4 Hz, 1H), 2.11 (d, J = 12.1 Hz, 1H), 1.94 (d, J = 8.0 Hz, 1H), 1.53 (d, J = 13.7 Hz, 9H). ^{13}C NMR (126 MHz, $CDCl_3$) δ 166.23, 164.91, 152.55, 152.40, 149.05, 147.87, 145.72, 145.25, 136.59, 130.56, 129.88, 128.58, 128.56, 126.93, 126.82, 126.57, 126.47, 125.56, 125.13, 122.18, 112.20, 108.81, 82.24, 81.49, 40.63, 40.59, 40.08, 34.95, 32.21, 32.04, 32.02, 29.99, 28.29, 28.17, 26.88, 26.64. HRMS (ESI) m/z calculated for $C_{28}H_{32}N_2O_4S$ $[M+H]^+$: 493.2103; found $[M+H]^+$: 493.2108.

BDA-43: Tert-butyl (3-((4-hydroxyphenethyl) carbamoyl)-4,5,6,7-tetrahydrobenzo [b]thiophen-2-yl) carbamate.

The compounds **BDA-43** was synthesized according to step **4**, and yielded final compound (168 mg, 81%) yellow solid, M.P.= 182 - 188 °C; ^1H NMR (500 MHz, $CDCl_3$) δ 11.05 (s, 1H), 10.27 (s, 1H), 7.33 – 7.29 (m, 2H), 7.16 – 7.11 (m, 2H), 5.85 (t, J = 5.8 Hz, 1H), 3.71 (q, J = 6.9 Hz, 2H), 2.97 (t, J = 6.9 Hz, 2H), 2.92 (d, ^{13}C NMR (126 MHz, $CDCl_3$) δ 166.30, 165.00, 152.53, 152.05, 149.08, 147.46, 136.54, 130.78, 129.83, 127.15, 125.99, 125.61, 122.18, 112.41, 109.03, 82.04, 81.33, 40.60, 34.98, 28.28, 28.17, 26.54, 26.52, 24.36, 24.28, 22.99, 22.90, 22.82, 22.62. HRMS (ESI) m/z calculated for $C_{22}H_{28}N_2O_4S$ $[M+H]^+$: 417.1833; found $[M+H]^+$: 417.1837.

^{1}H NMR spectrum of **BDA-01** (500 MHz, $CDCl_3$)

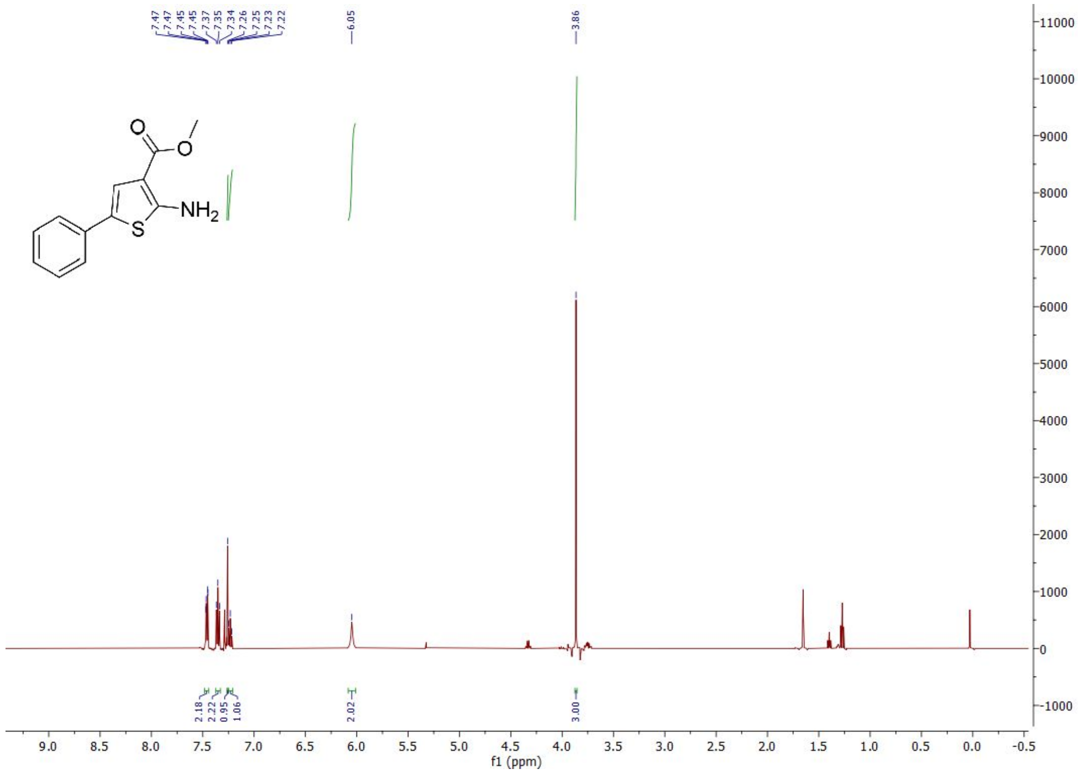

^{13}C{^{1}H} NMR spectrum of **BDA-01** (126 MHz, $CDCl_3$)

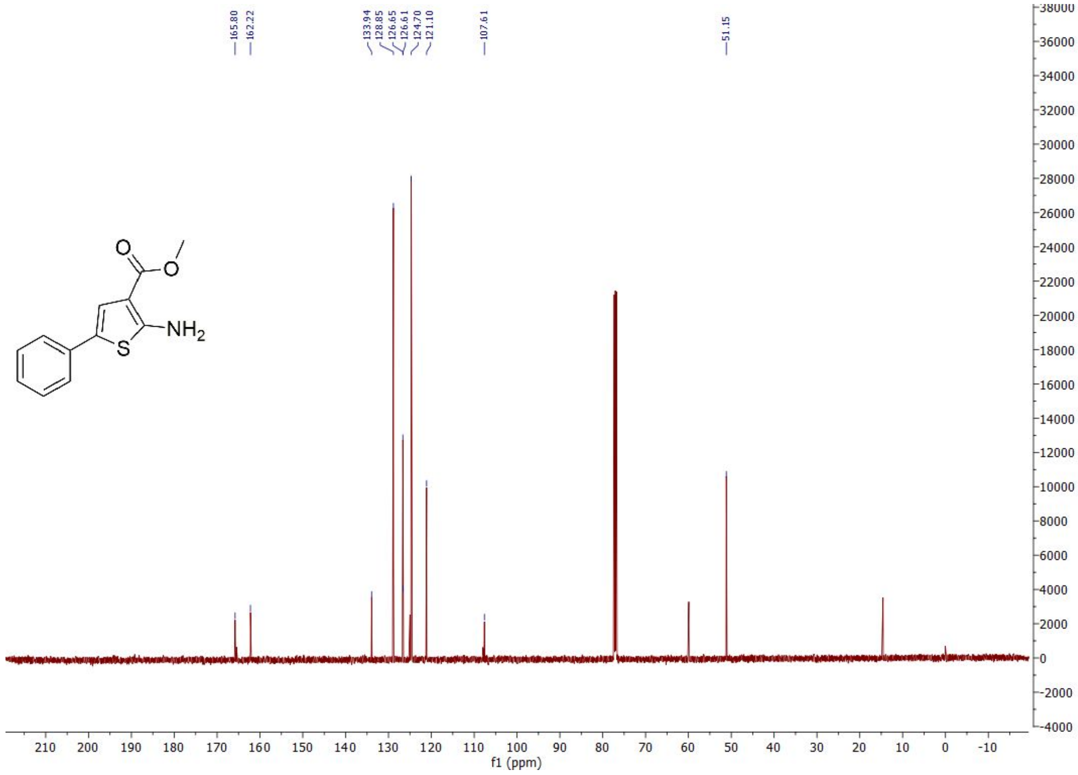

^{1}H NMR spectrum of **BDA-02** (500 MHz, $CDCl_3$)

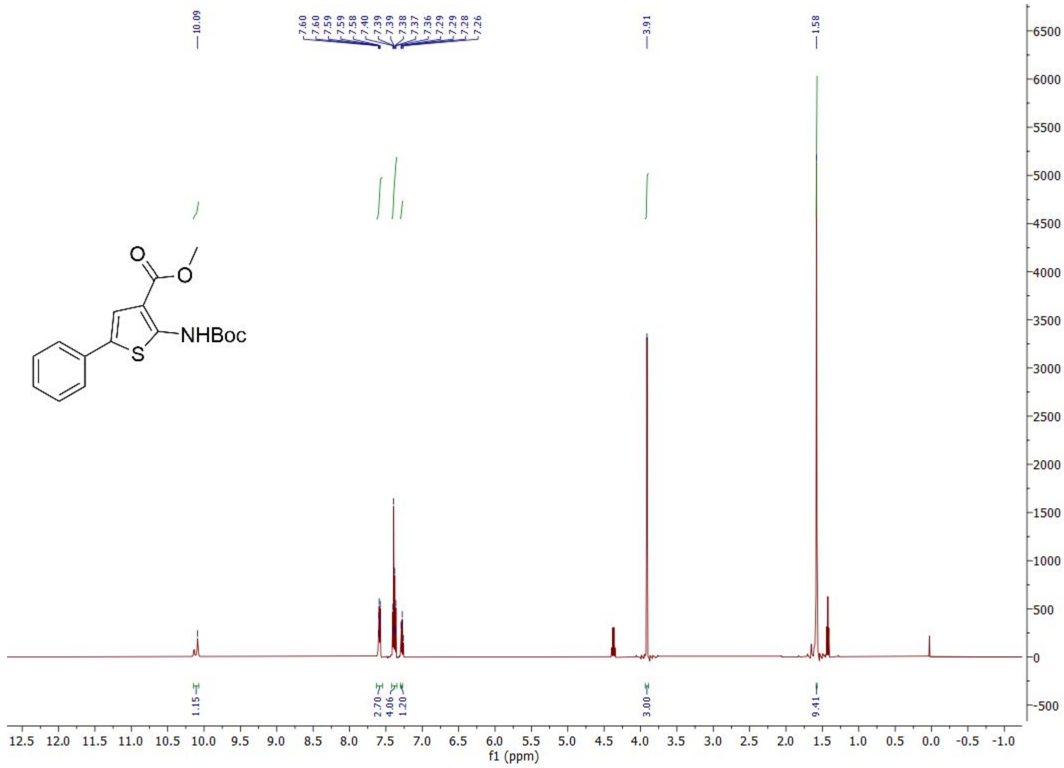

$^{13}C\{^{1}H\}$ NMR spectrum of **BDA-02** (126 MHz, $CDCl_3$)

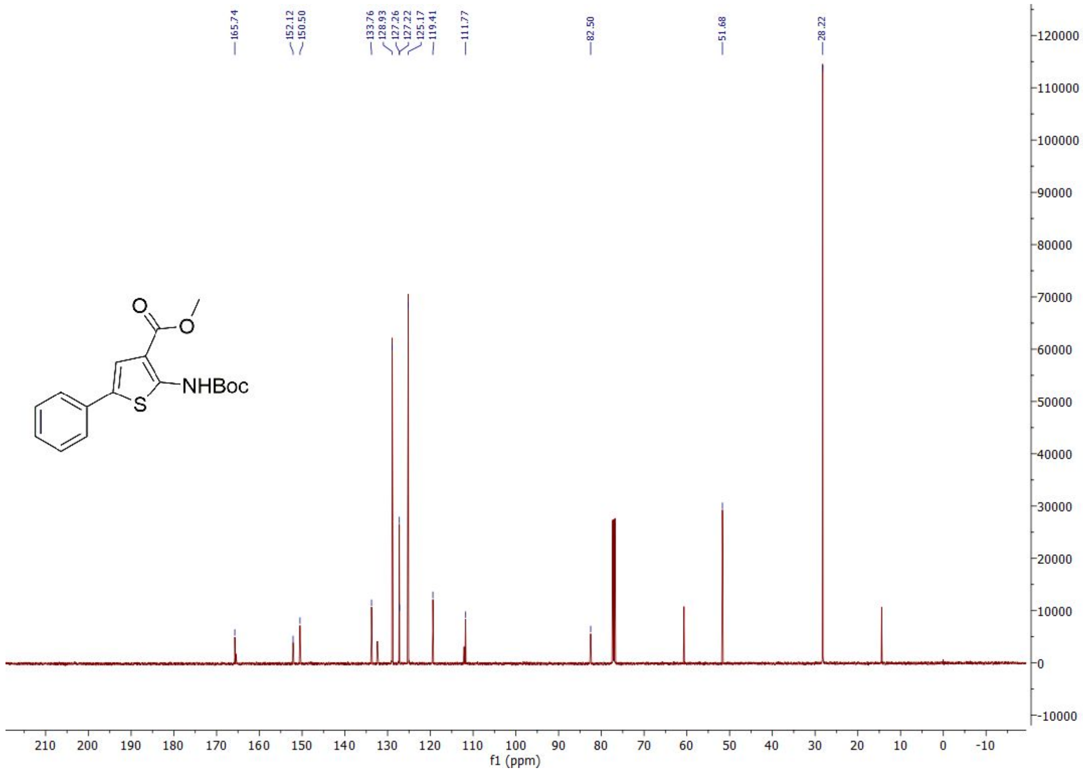

^{1}H NMR spectrum of **BDA-03** (500 MHz, DMSO-d_6)

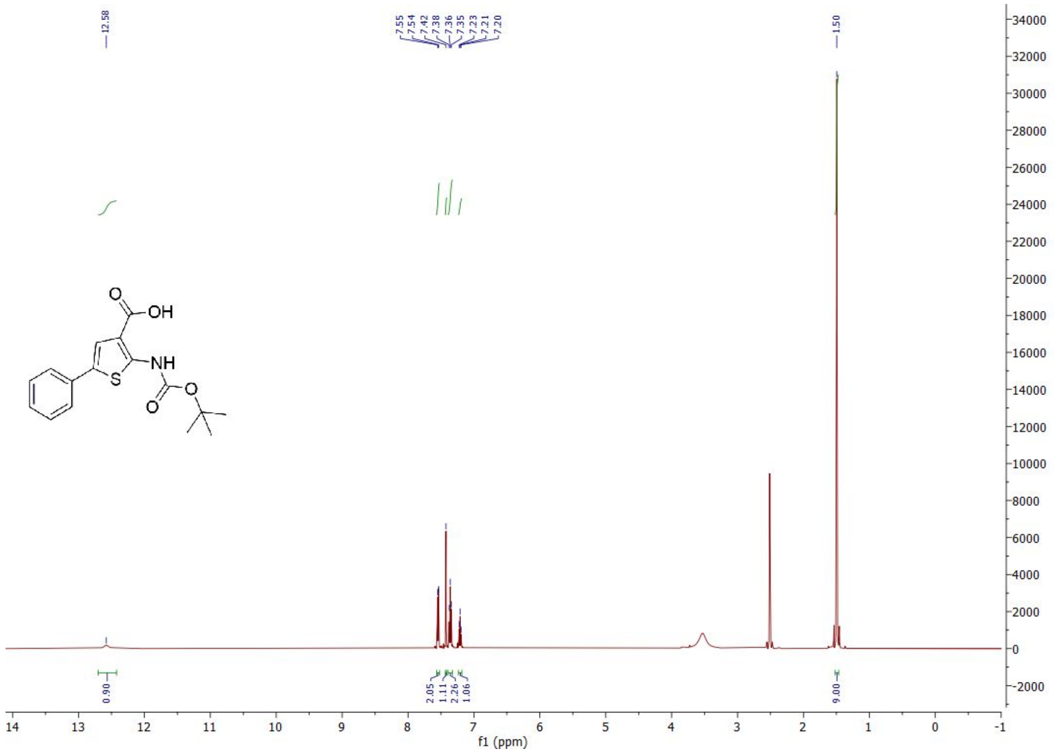

^{13}C{^{1}H} NMR spectrum of **BDA-03** (126 MHz, DMSO-d_6)

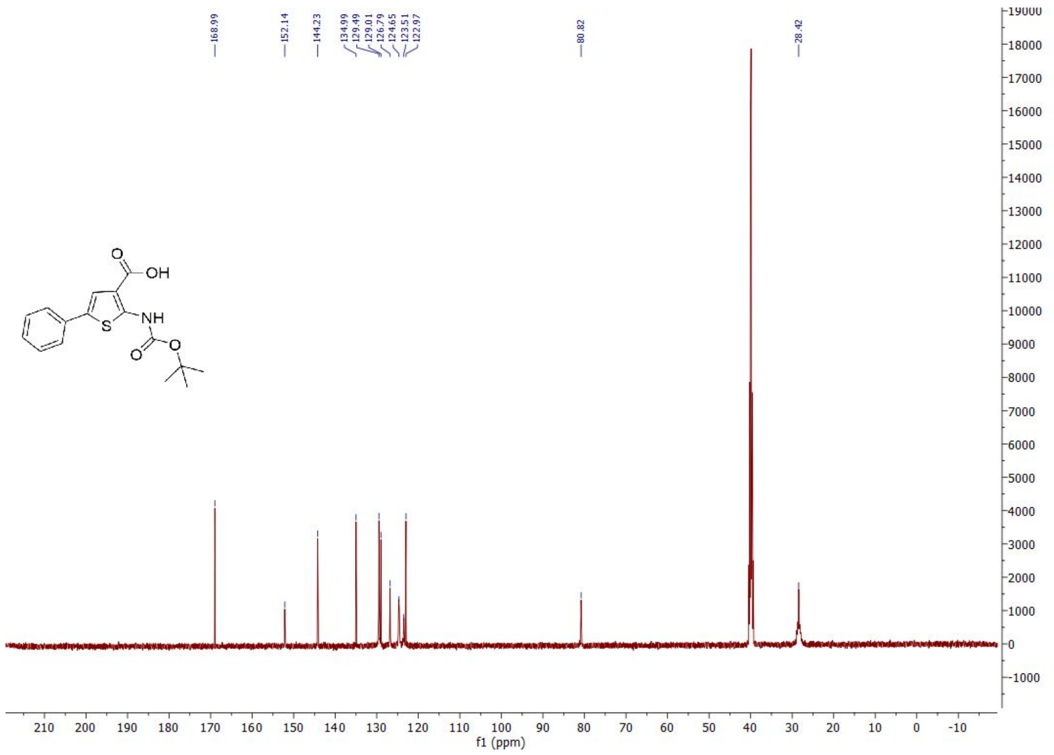

^{1}H NMR spectrum of **BDA-04** (500 MHz, $CDCl_3$)

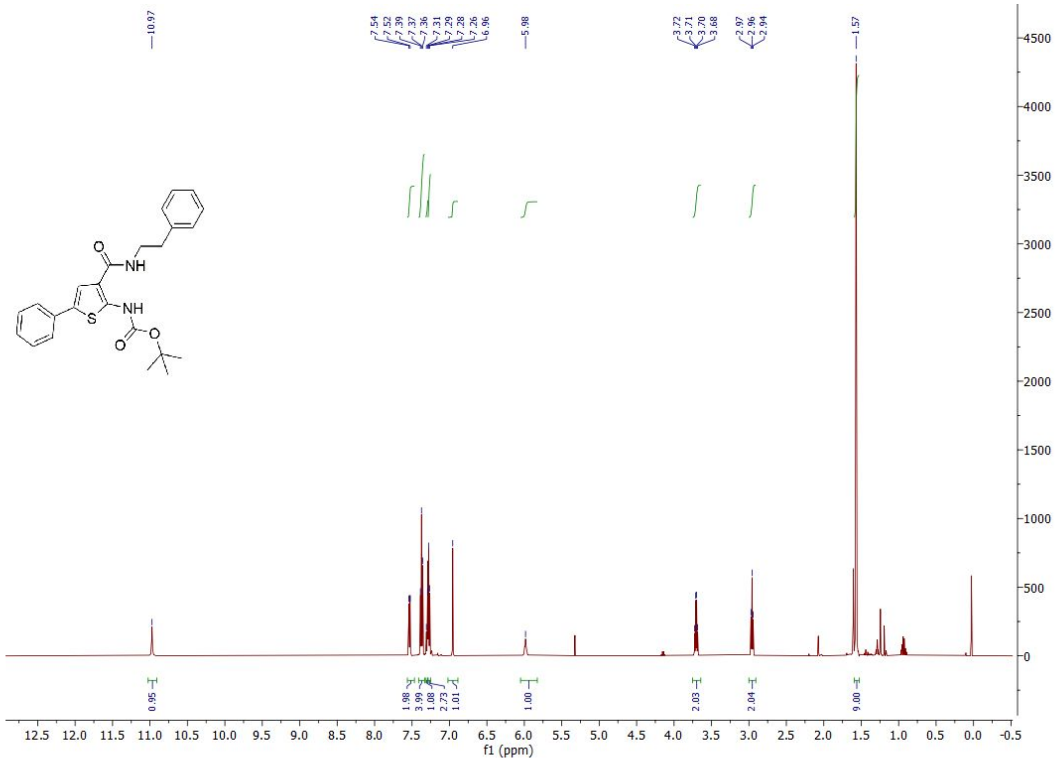

$^{13}C\{^{1}H\}$ NMR spectrum of **BDA-04** (126 MHz, $CDCl_3$)

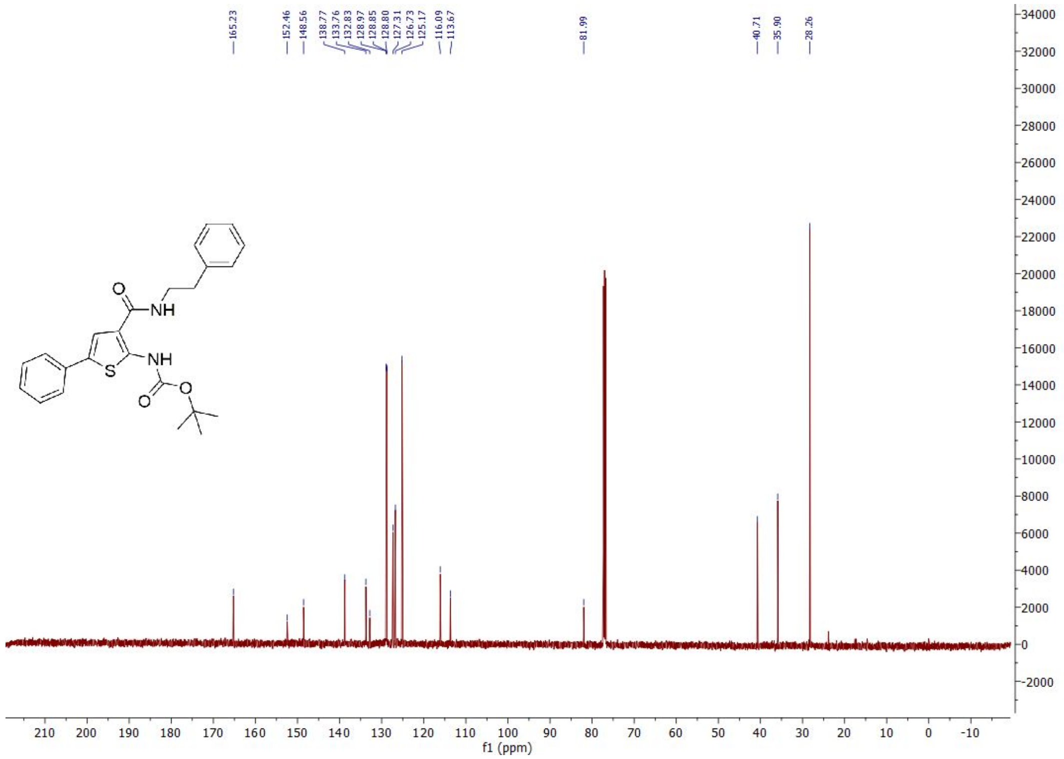

^{1}H NMR spectrum of **BDA-05** (500 MHz, $CDCl_3$)

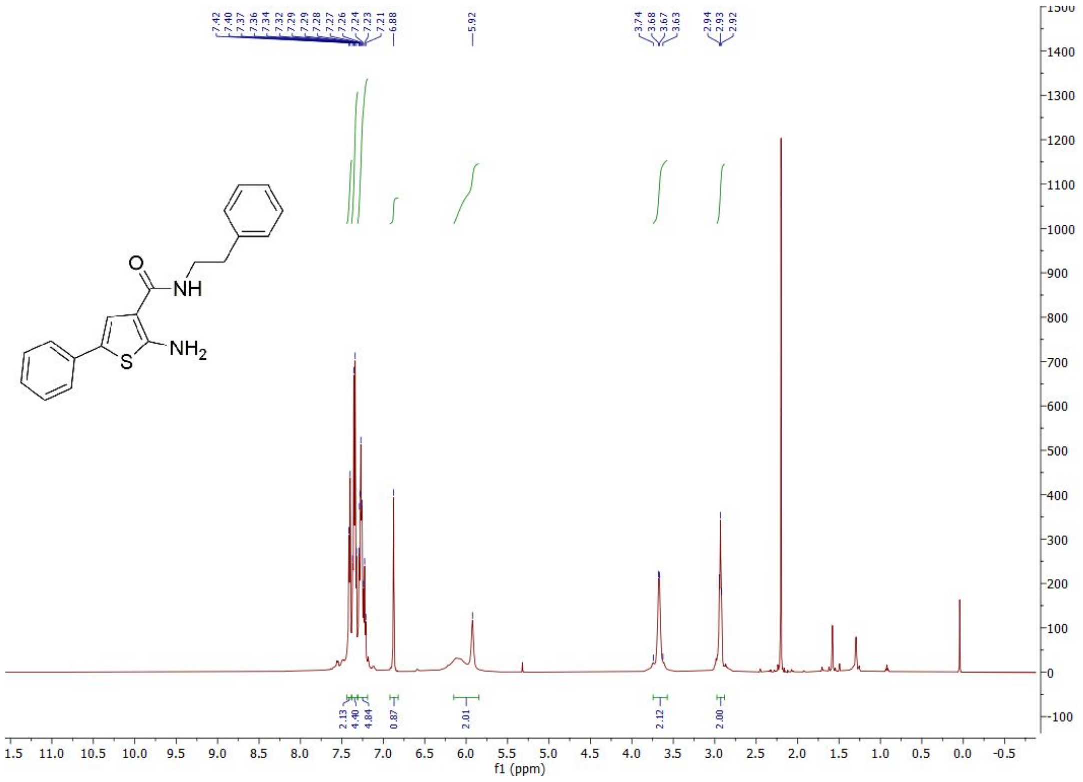

$^{13}C\{^{1}H\}$ NMR spectrum of **BDA-05** (126 MHz, $CDCl_3$)

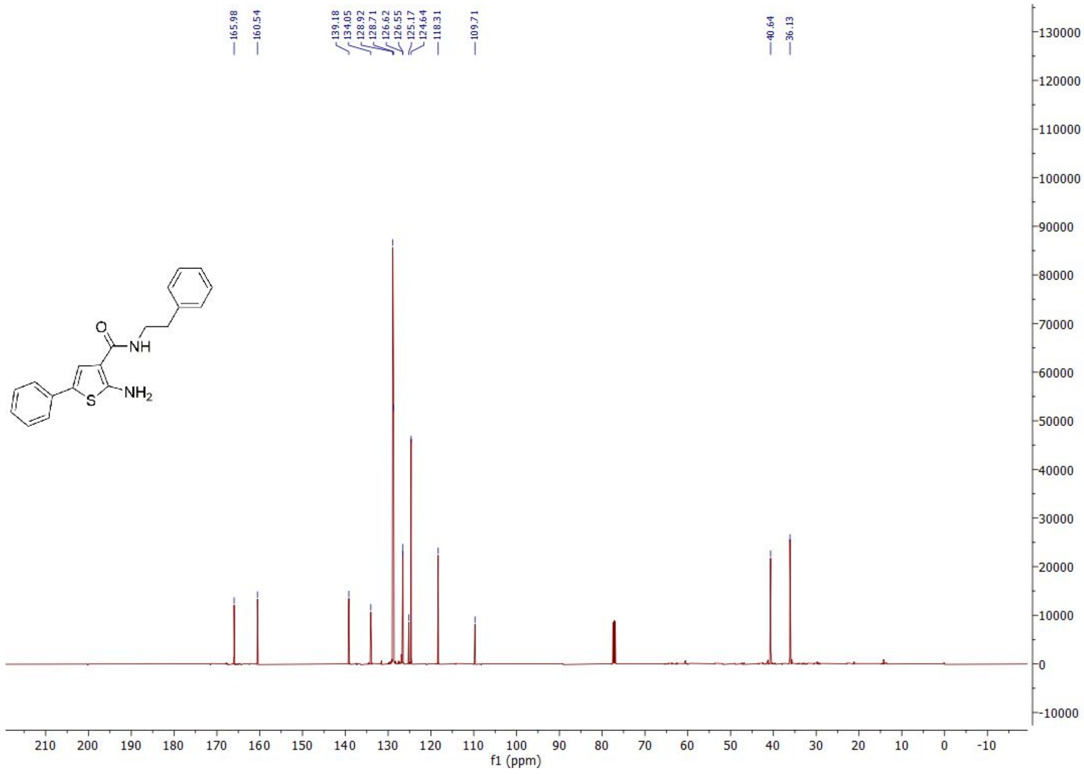

^{1}H NMR spectrum of **BDA-06** (500 MHz, DMSO-d$_6$)

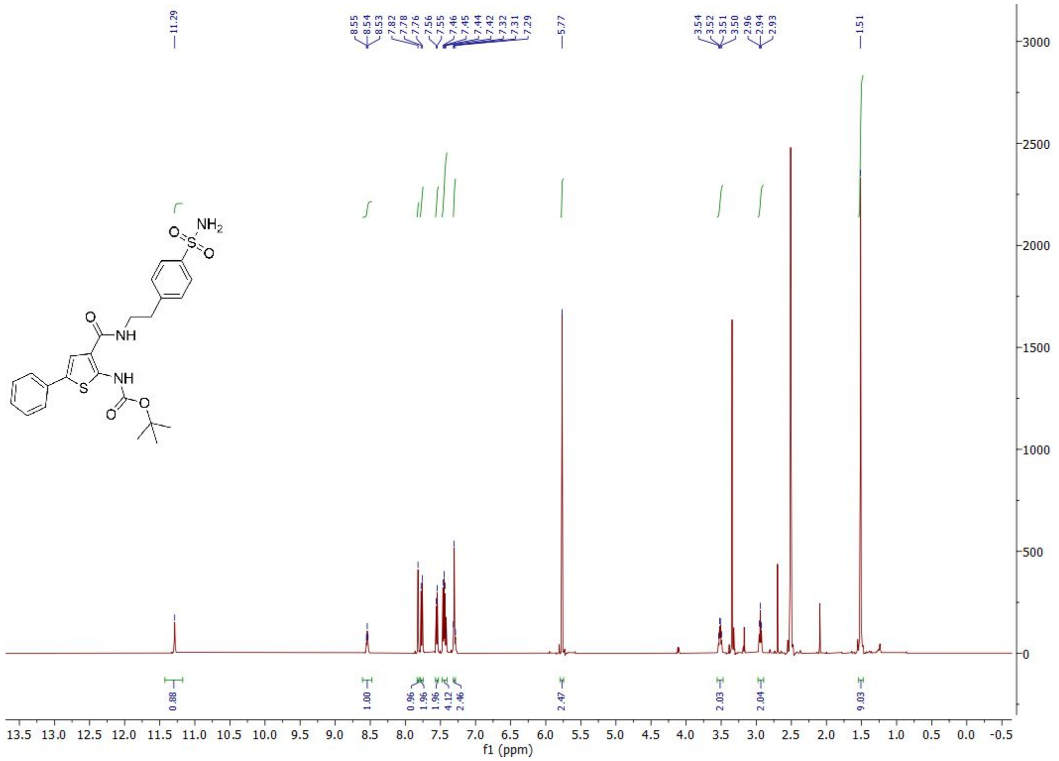

^{13}C{^{1}H} NMR spectrum of **BDA-06** (126 MHz, DMSO-d$_6$)

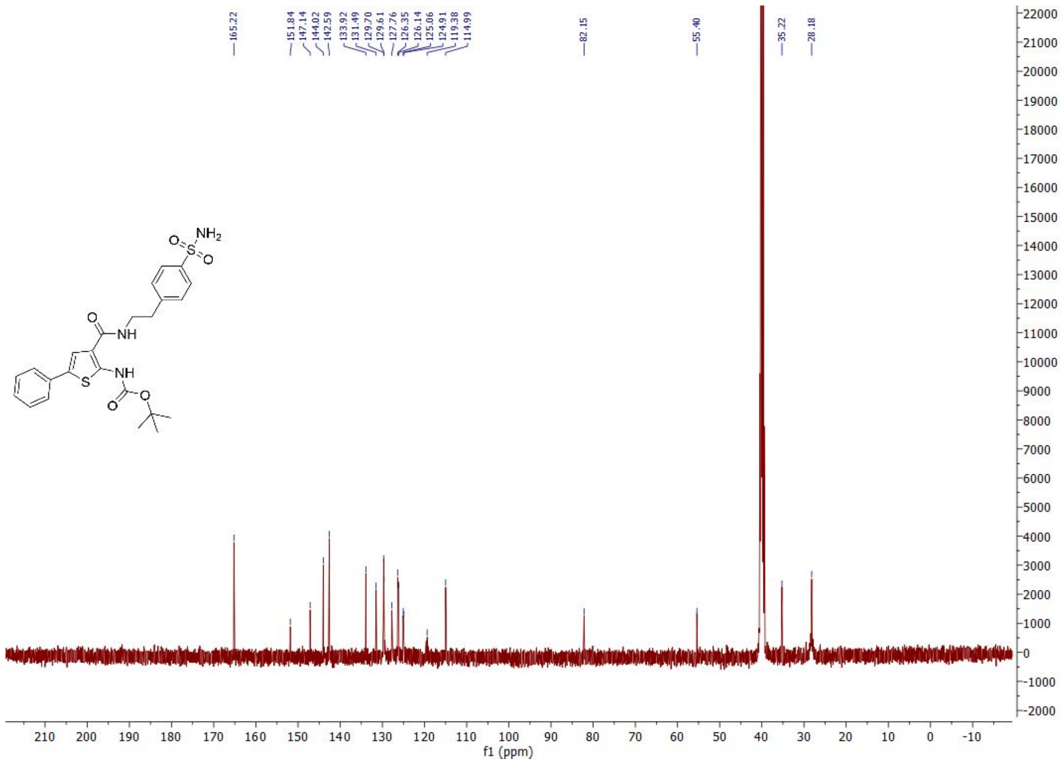

^{1}H NMR spectrum of **BDA-07** (500 MHz, DMSO-d_6)

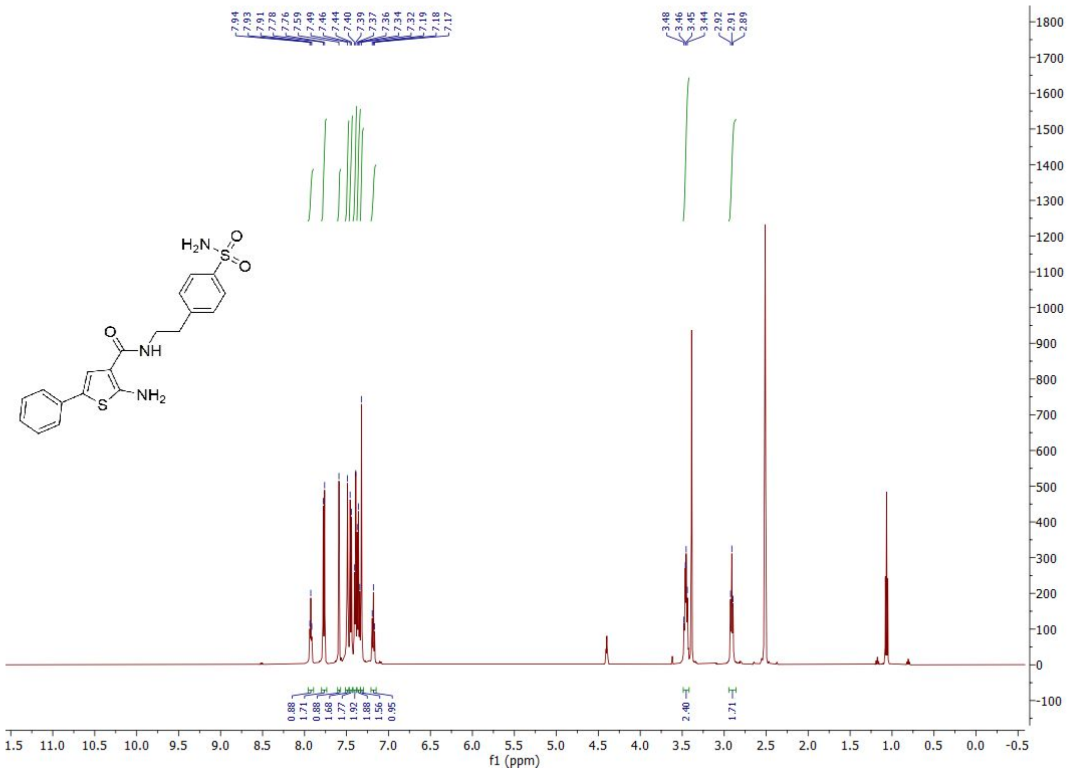

$^{13}C\{^{1}H\}$ NMR spectrum of **BDA-07** (126 MHz, DMSO-d_6)

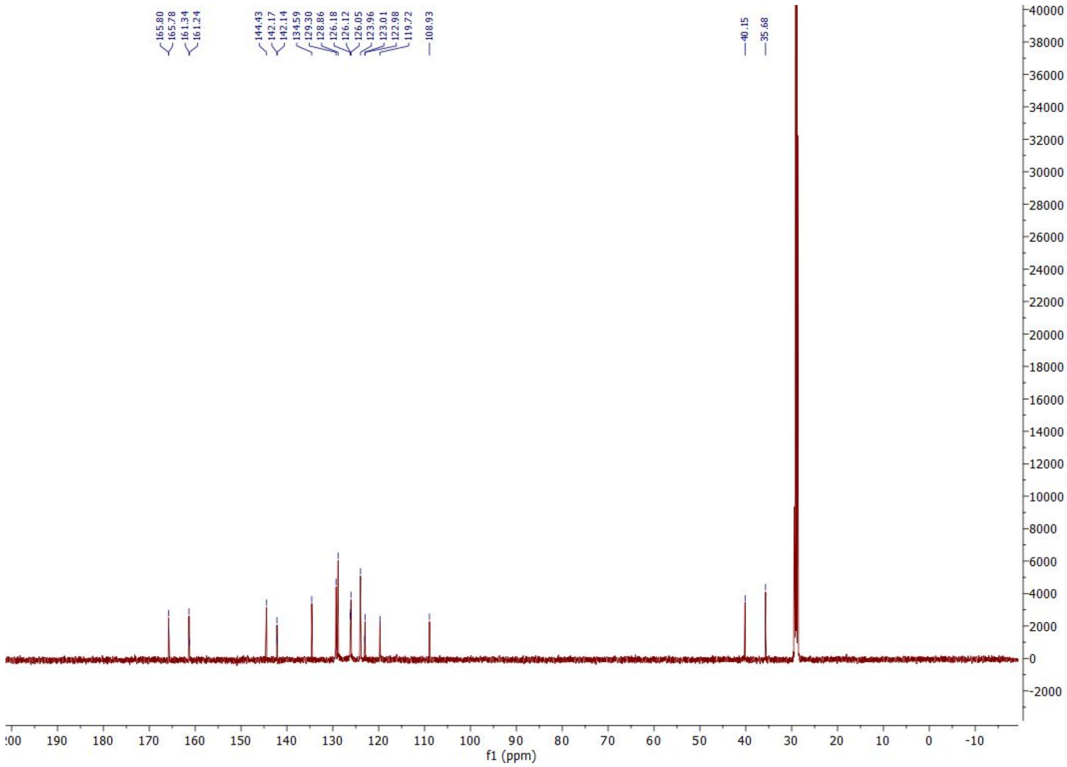

^{1}H NMR spectrum of **BDA-08** (500 MHz, DMSO-d_6)

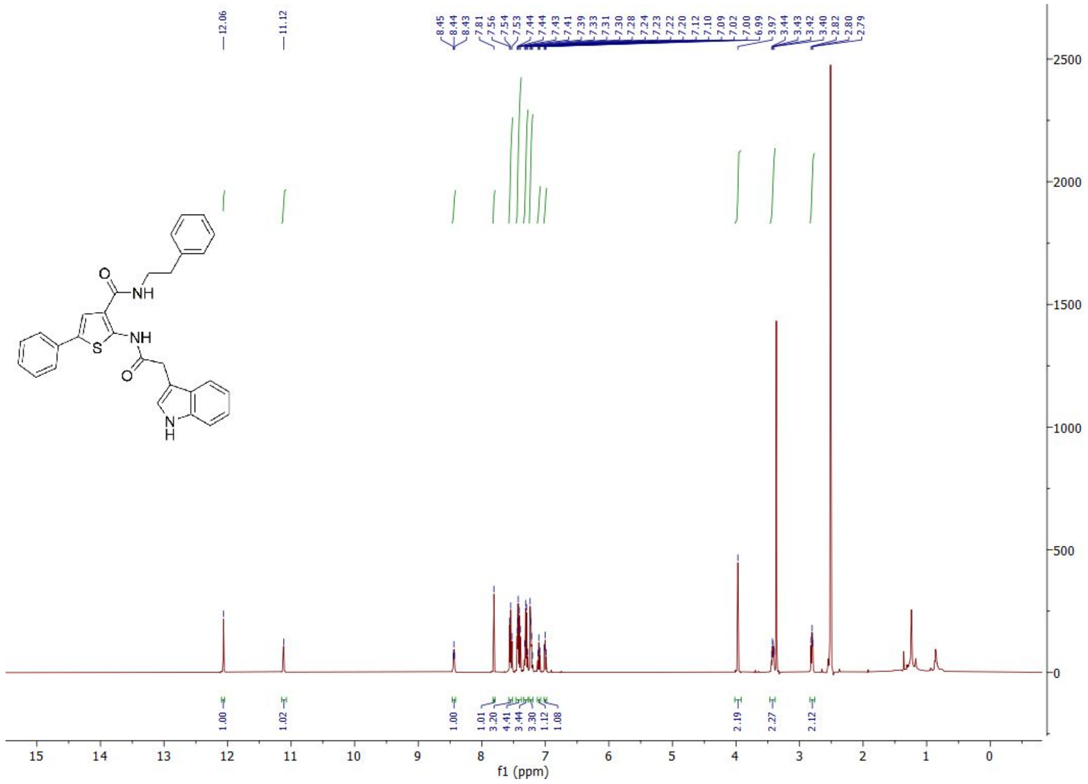

$^{13}C\{^{1}H\}$ NMR spectrum of **BDA-08** (126 MHz, DMSO-d_6)

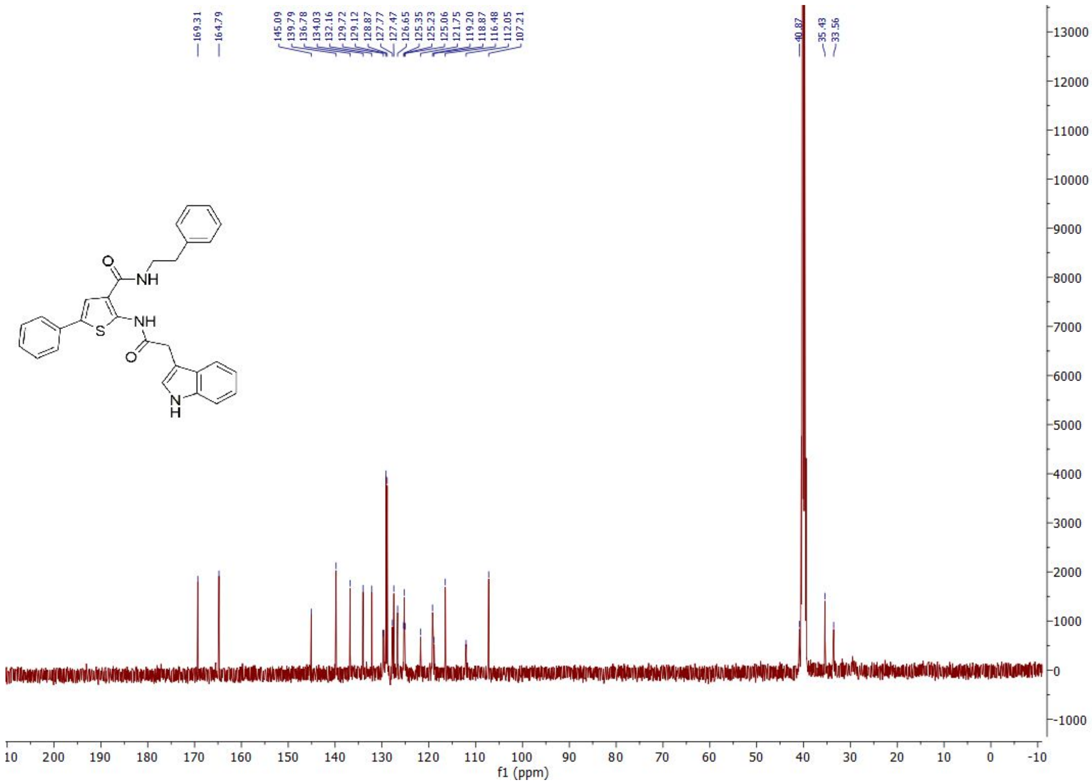

^{1}H NMR spectrum of **BDA-09** (500 MHz, $CDCl_3$)

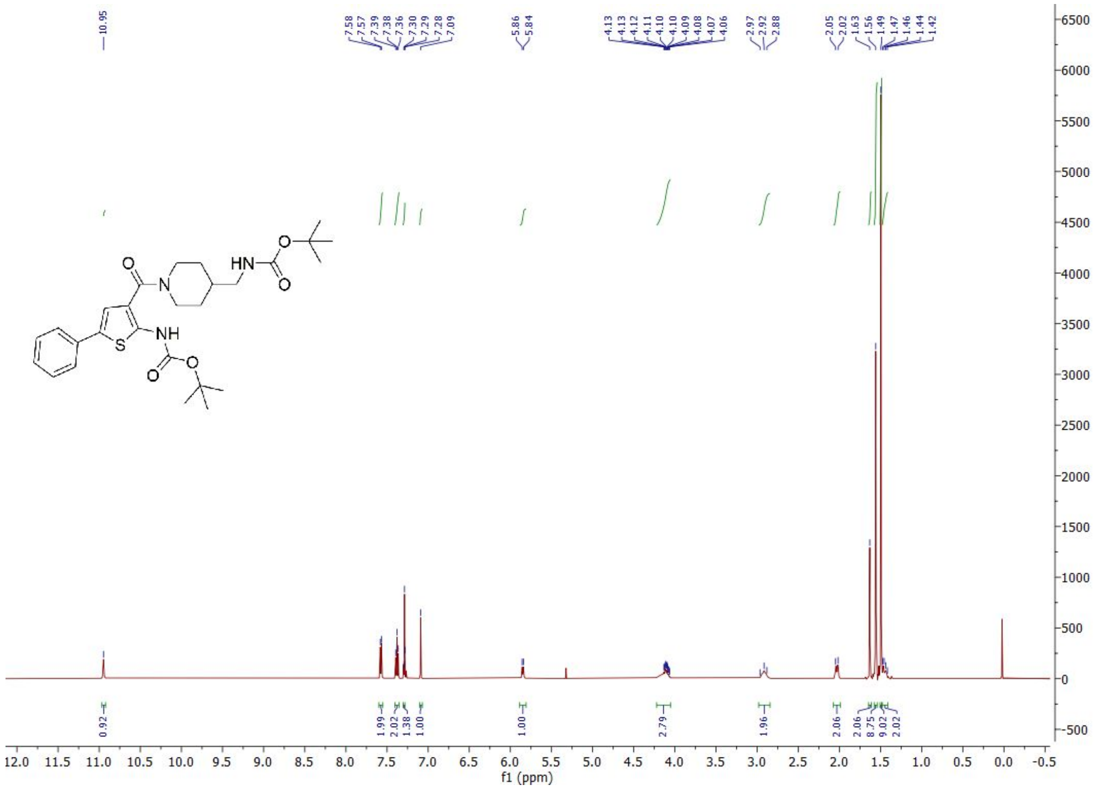

$^{13}C\{^{1}H\}$ NMR spectrum of **BDA-09** (126 MHz, $CDCl_3$)

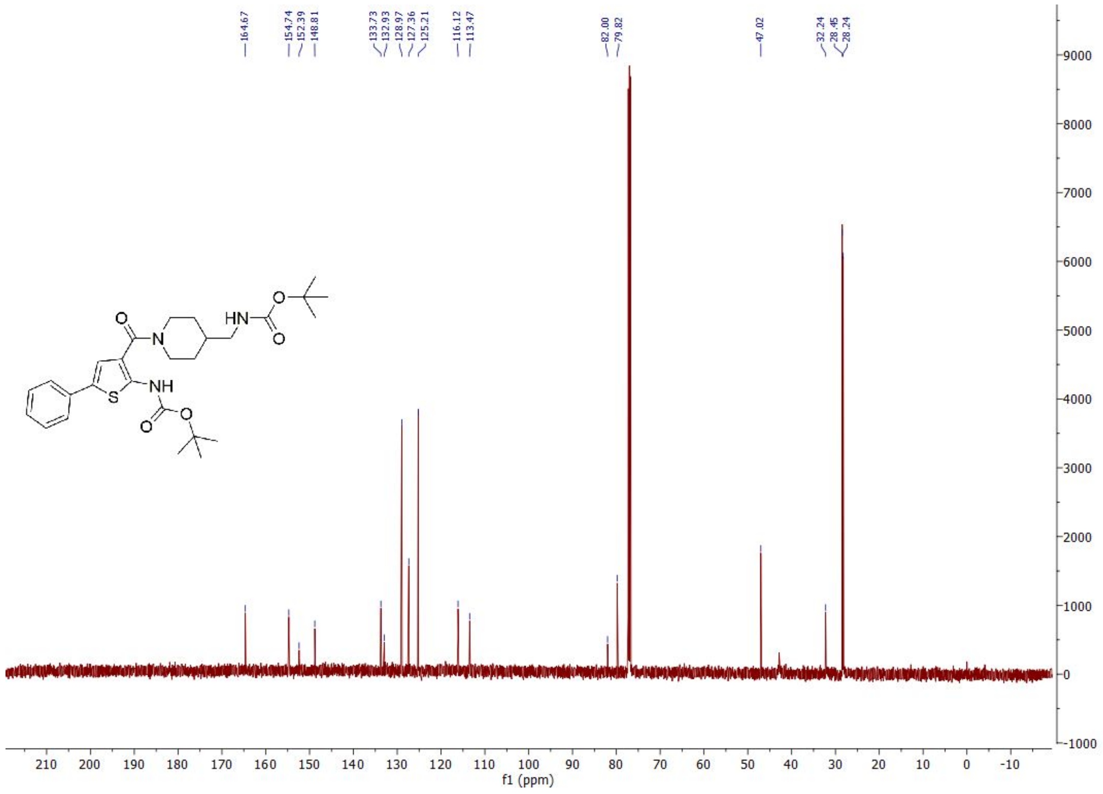

^{1}H NMR spectrum of **BDA-10** (500 MHz, $CDCl_3$)

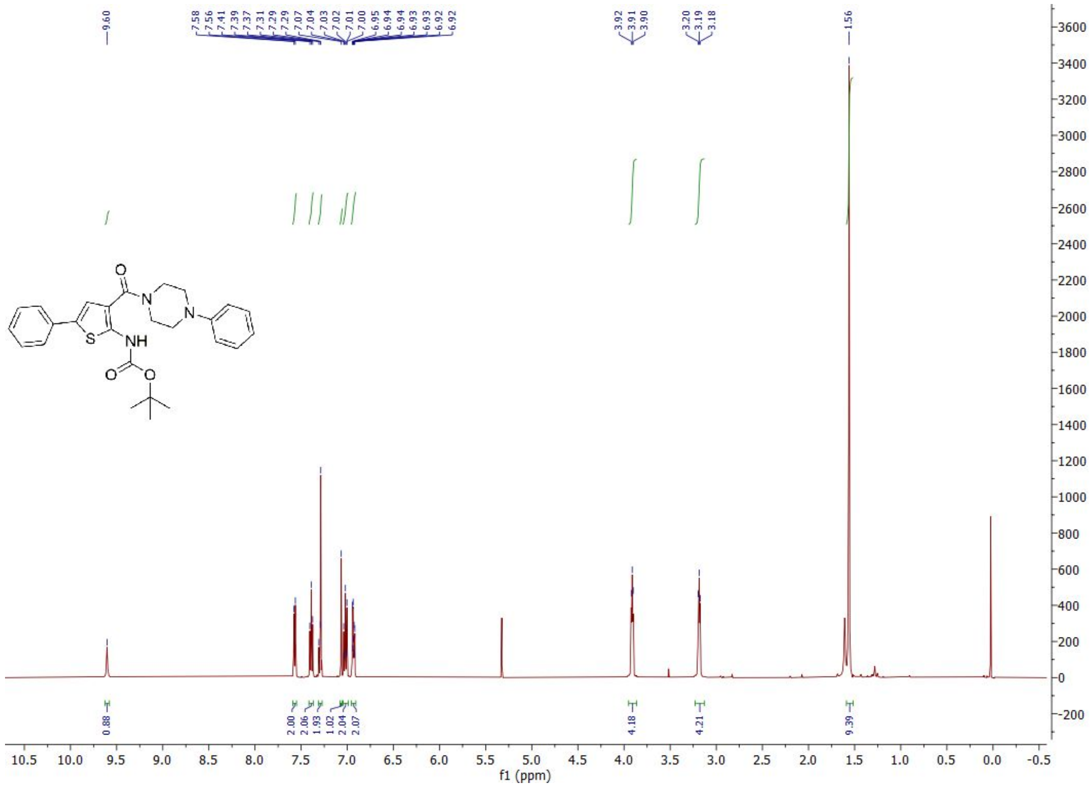

$^{13}C\{^{1}H\}$ NMR spectrum of **BDA-10** (126 MHz, $CDCl_3$)

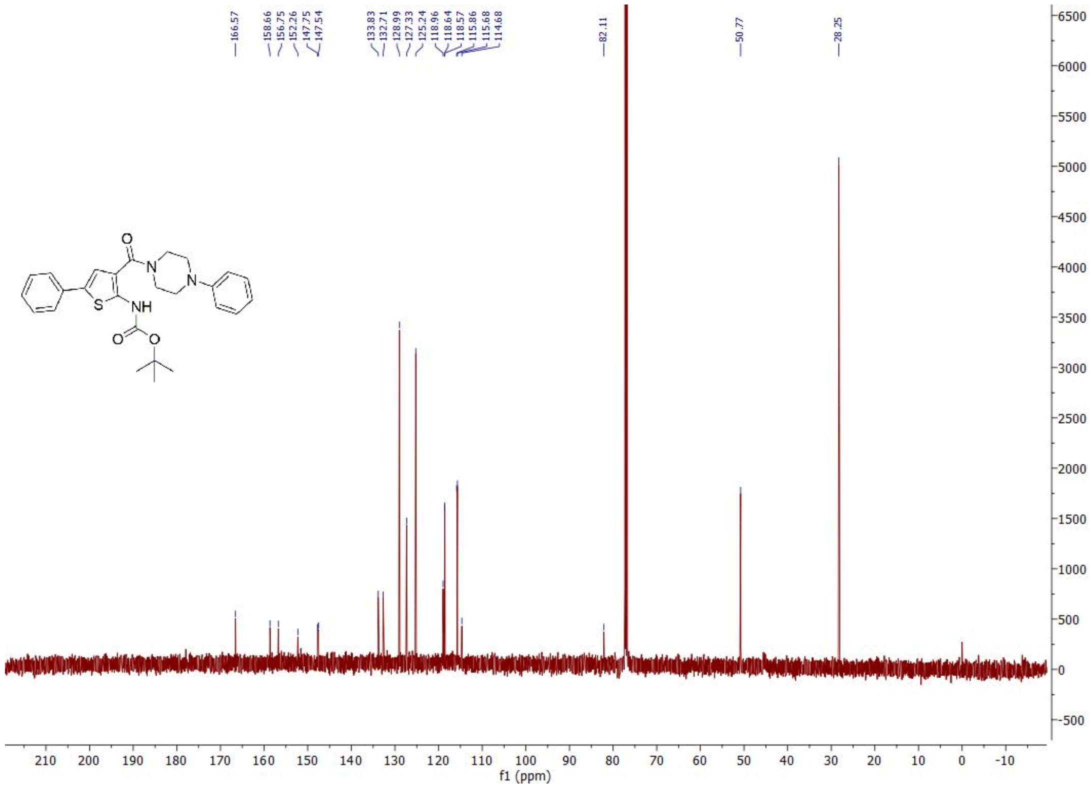

^{1}H NMR spectrum of **BDA-11** (500 MHz, $CDCl_3$)

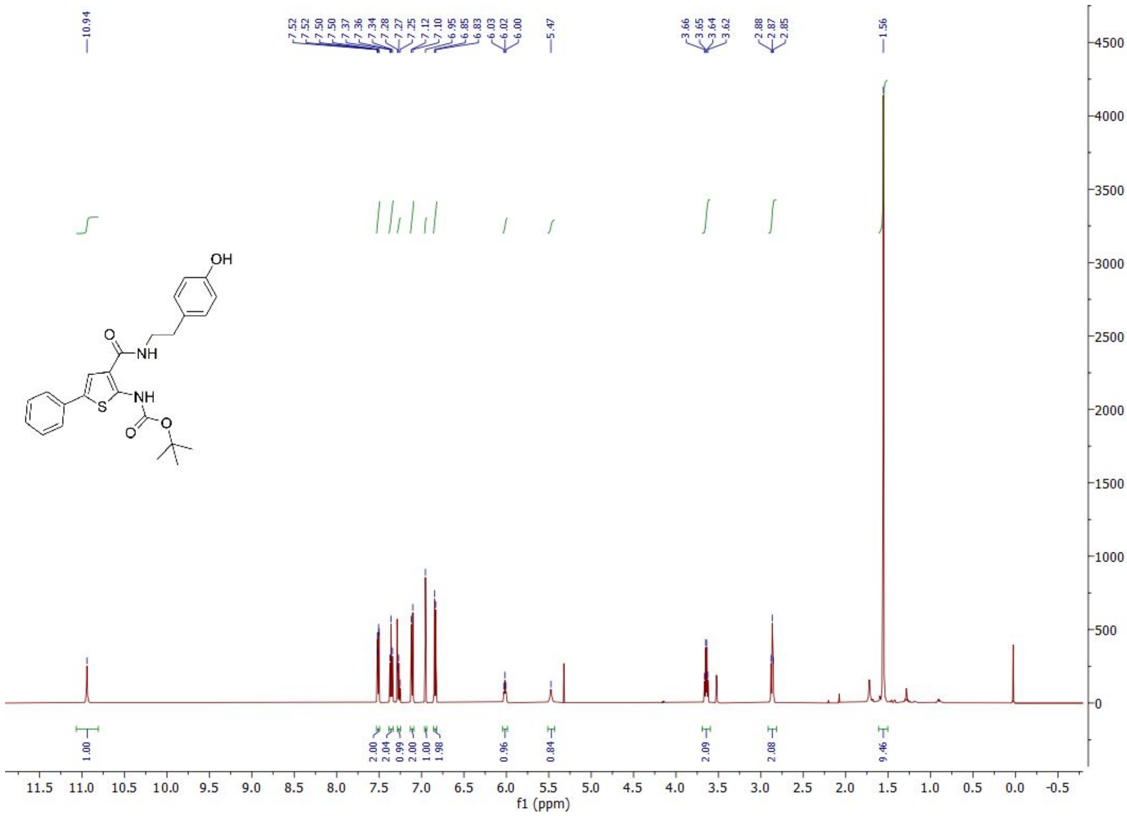

$^{13}C\{^{1}H\}$ NMR spectrum of **BDA-11** (126 MHz, $CDCl_3$)

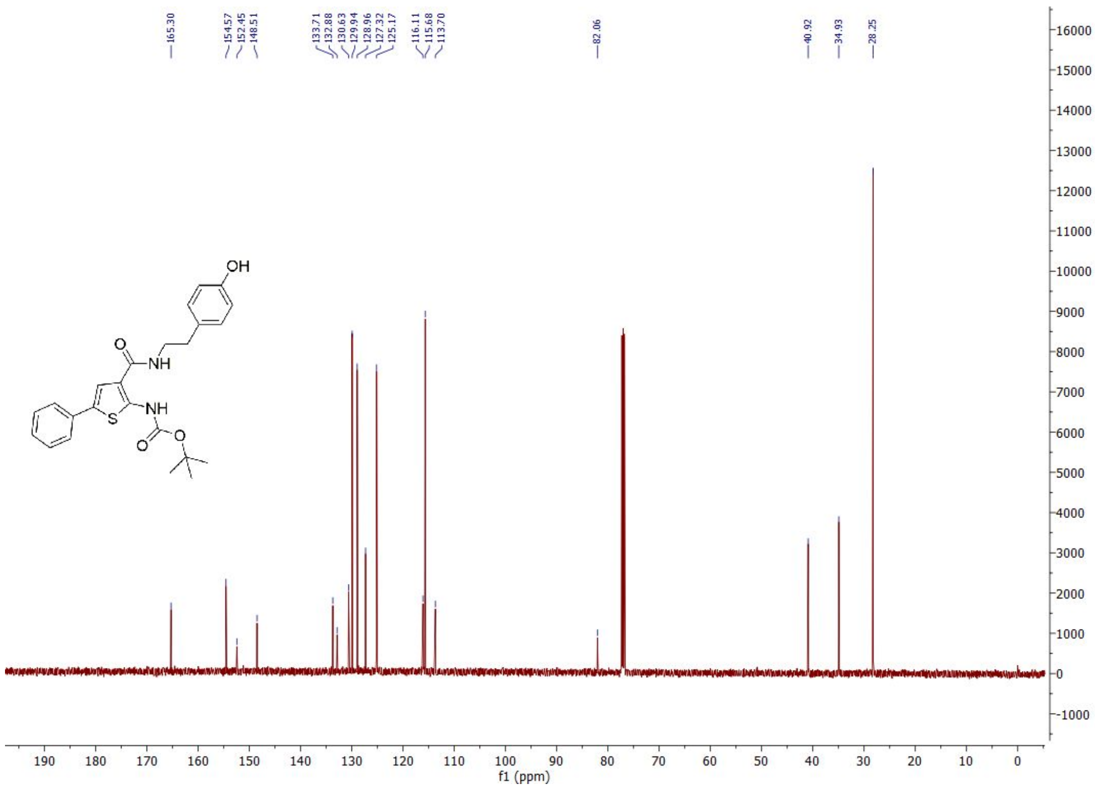

^{1}H NMR spectrum of **BDA-12** (500 MHz, $CDCl_3$)

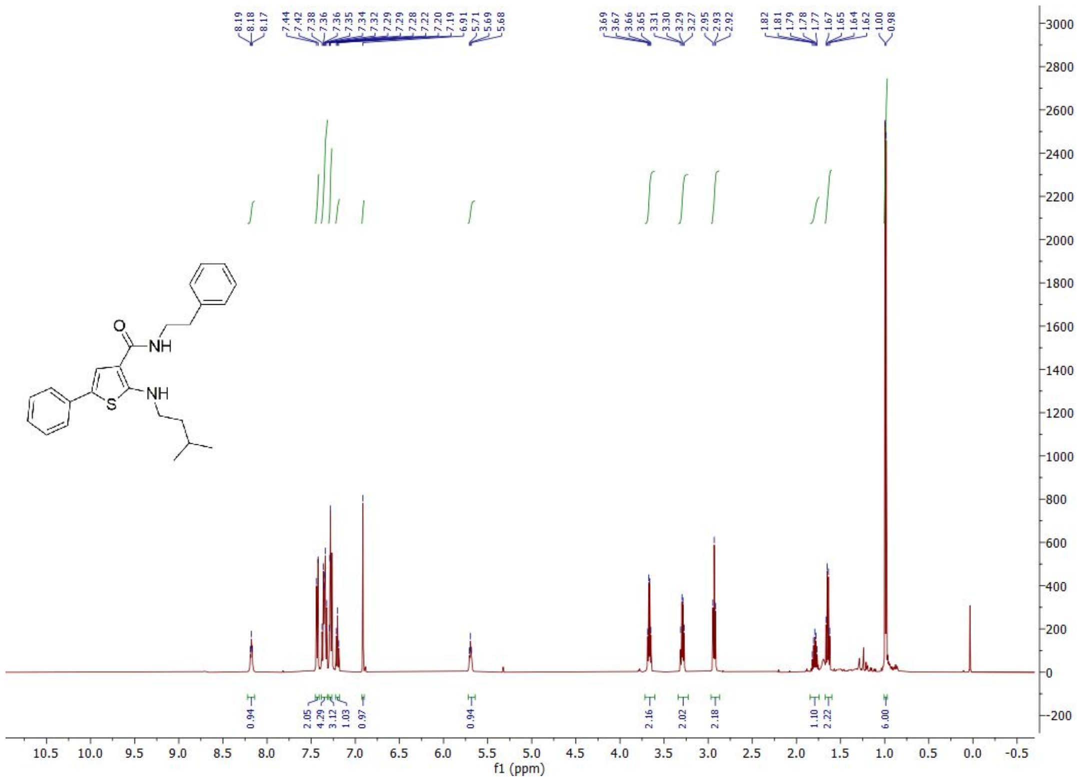

^{13}C{^{1}H} NMR spectrum of **BDA-12** (126 MHz, $CDCl_3$)

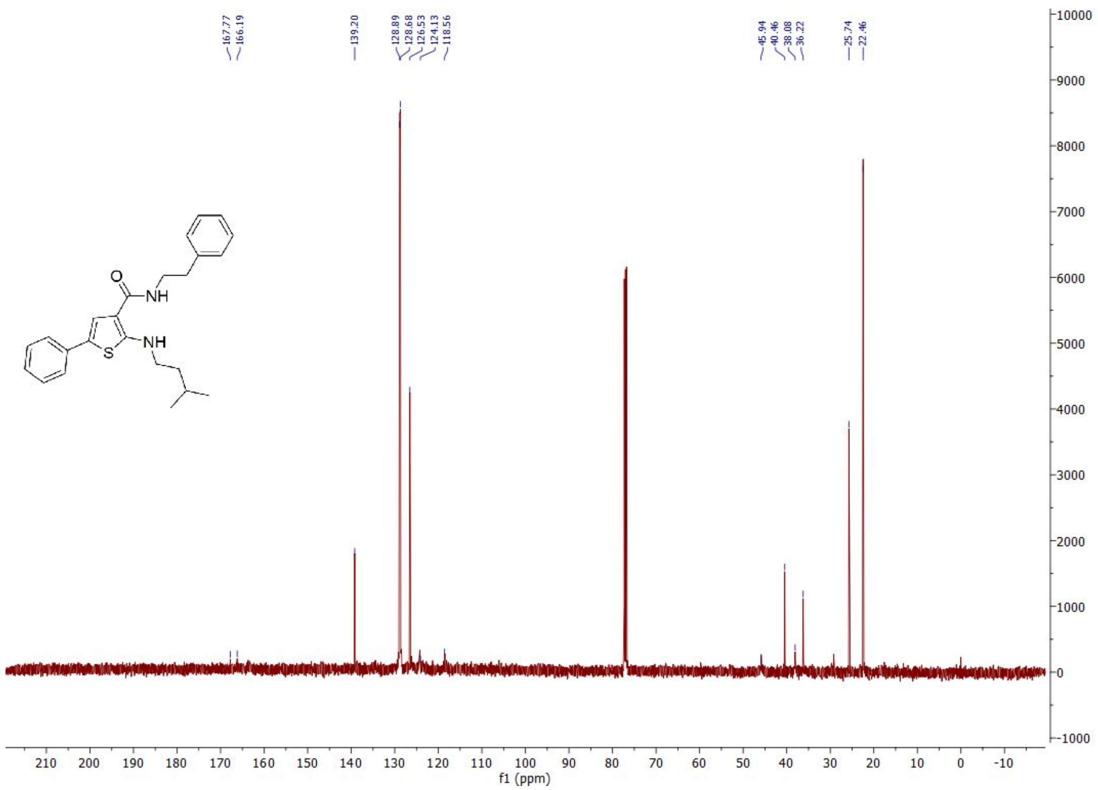

^{1}H NMR spectrum of **BDA-13** (500 MHz, $CDCl_3$)

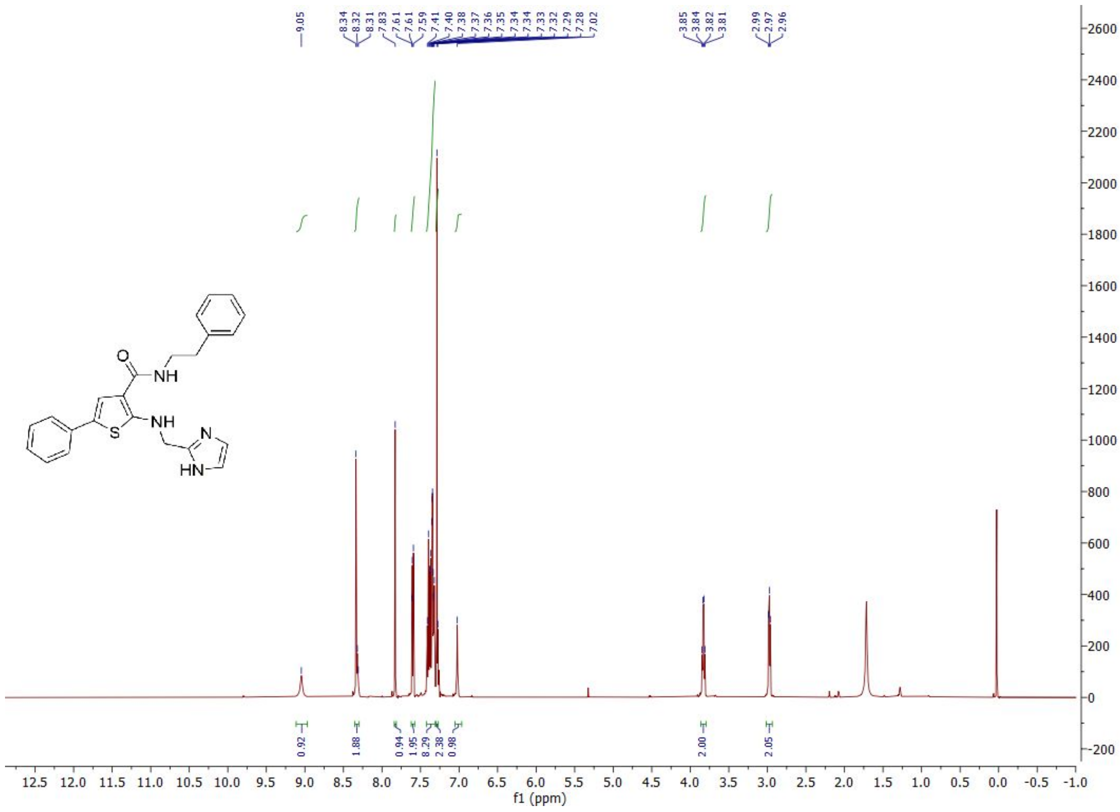

$^{13}C\{^1H\}$ NMR spectrum of **BDA-13** (126 MHz, $CDCl_3$)

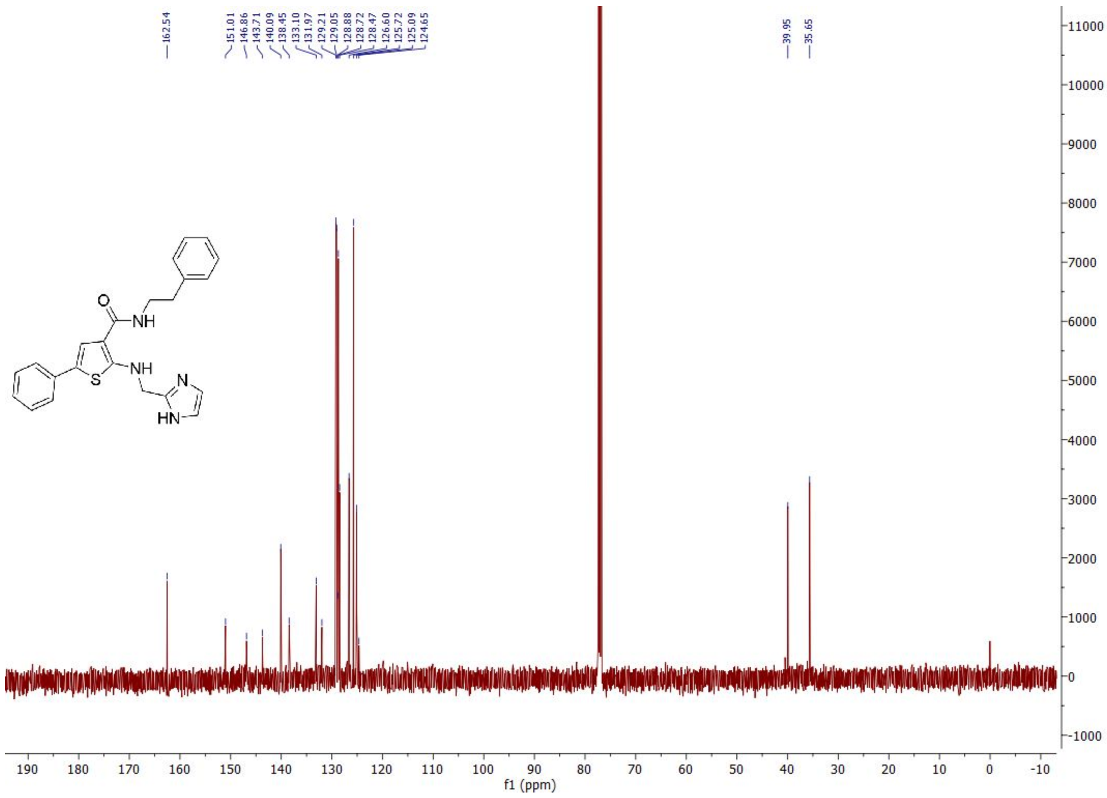

^{1}H NMR spectrum of **BDA-14** (500 MHz, $CDCl_3$)

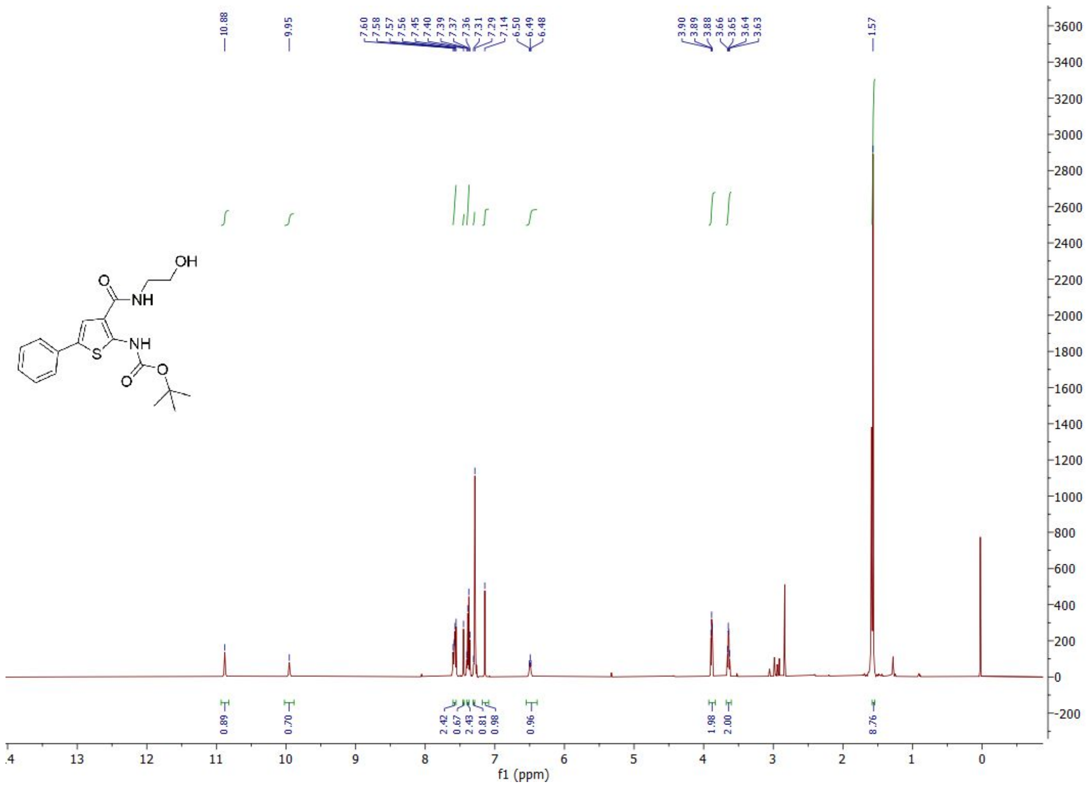

$^{13}C\{^{1}H\}$ NMR spectrum of **BDA-14** (126 MHz, $CDCl_3$)

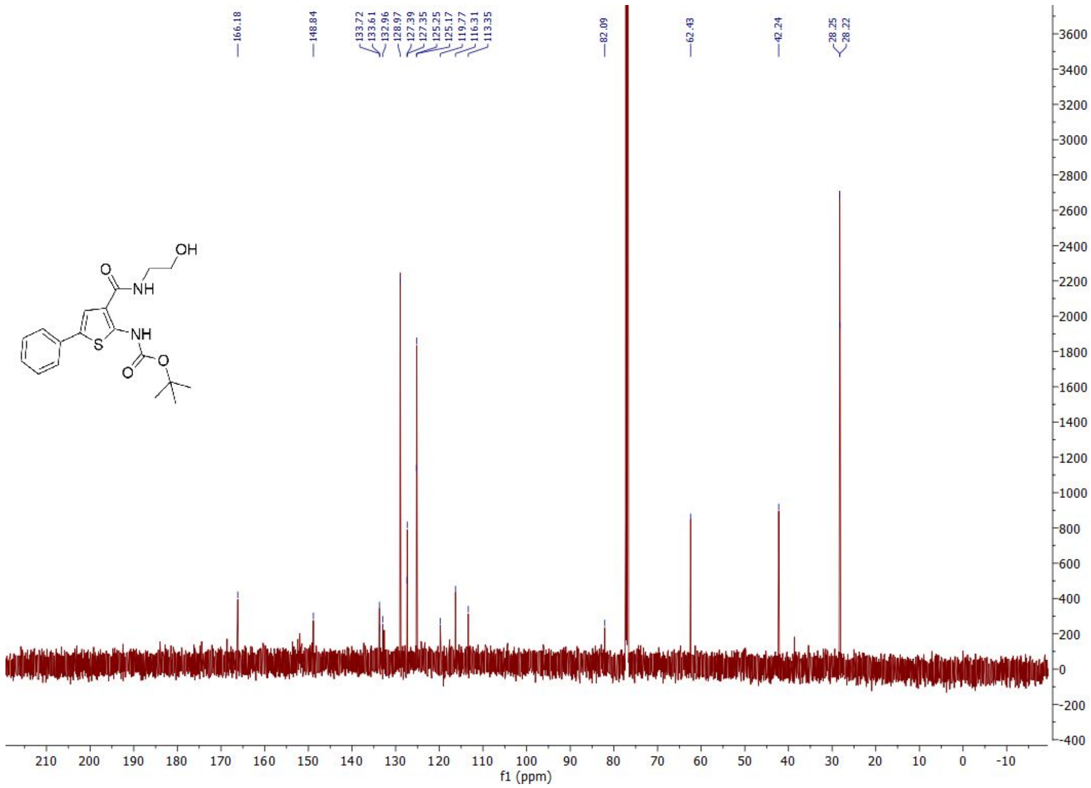

^{1}H NMR spectrum of **BDA-15** (500 MHz, $CDCl_3$)

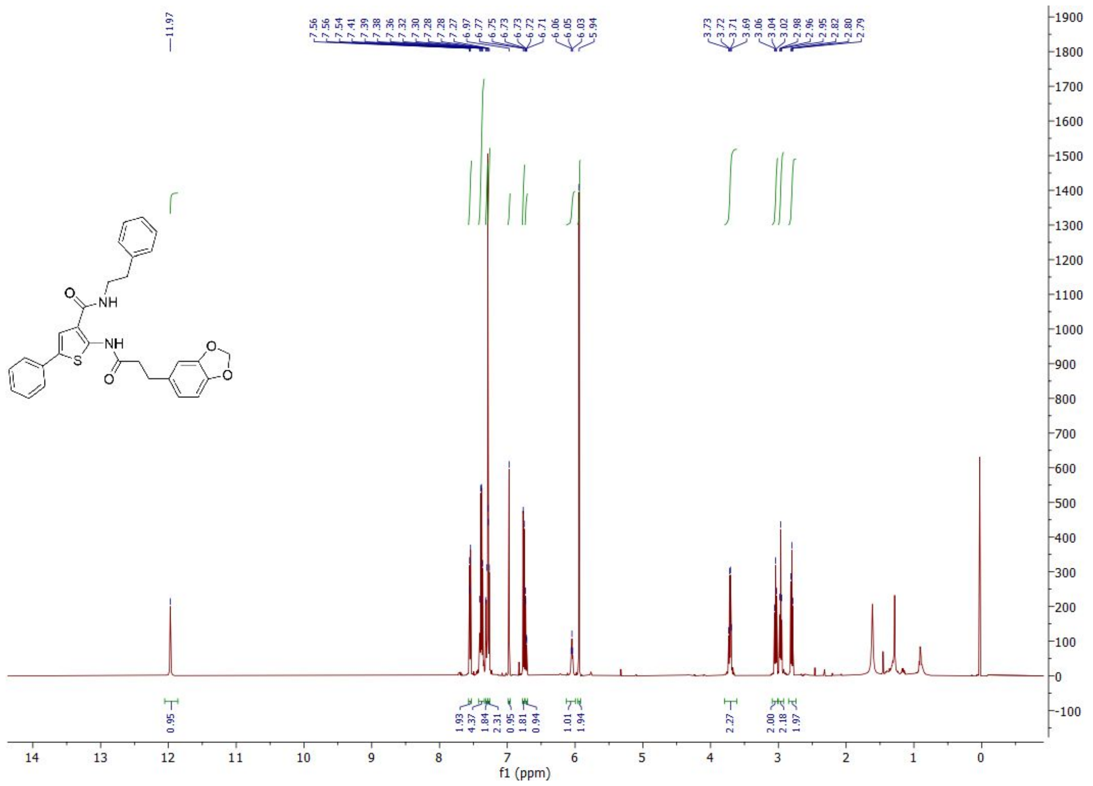

$^{13}C\{^{1}H\}$ NMR spectrum of **BDA-15** (126 MHz, $CDCl_3$)

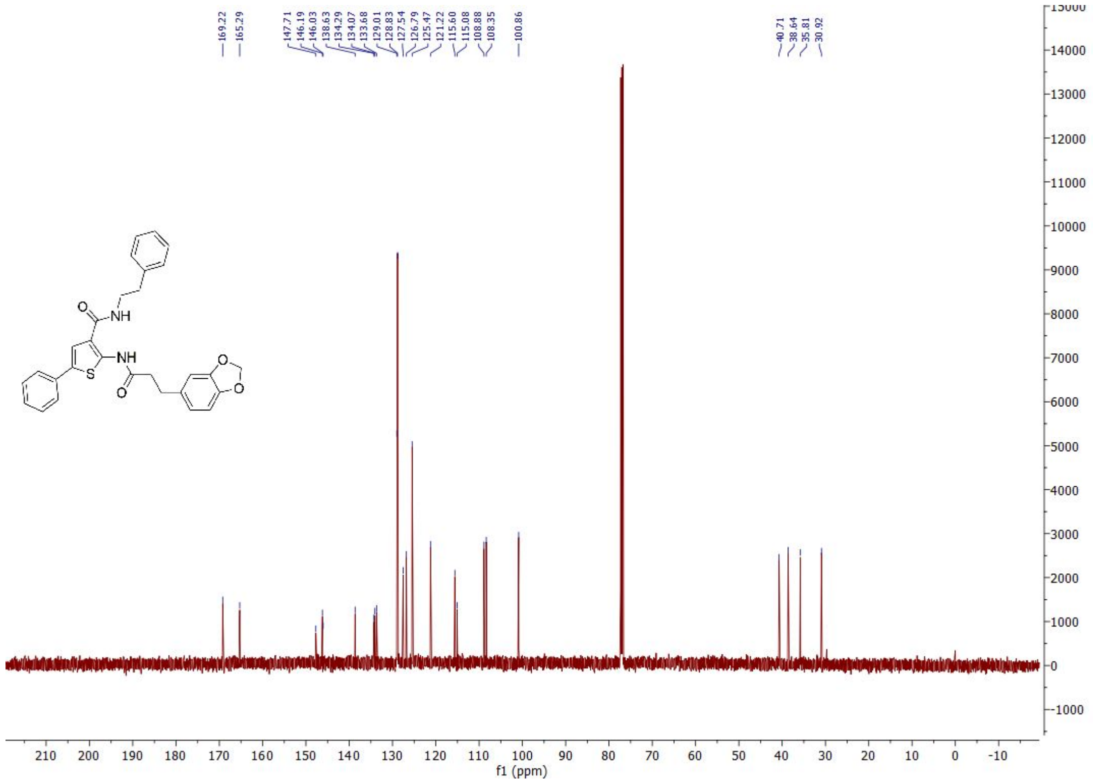

^{1}H NMR spectrum of **BDA-16** (500 MHz, DMSO-d_6)

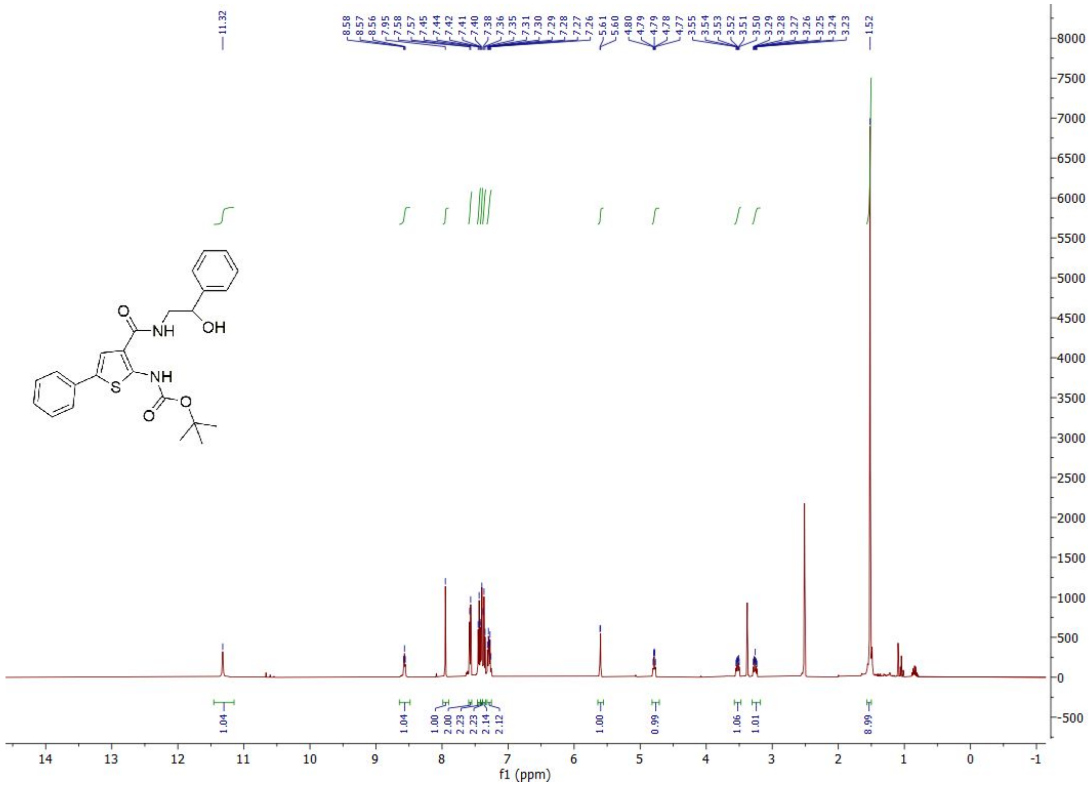

$^{13}C\{^{1}H\}$ NMR spectrum of **BDA-16** (126 MHz, DMSO-d_6)

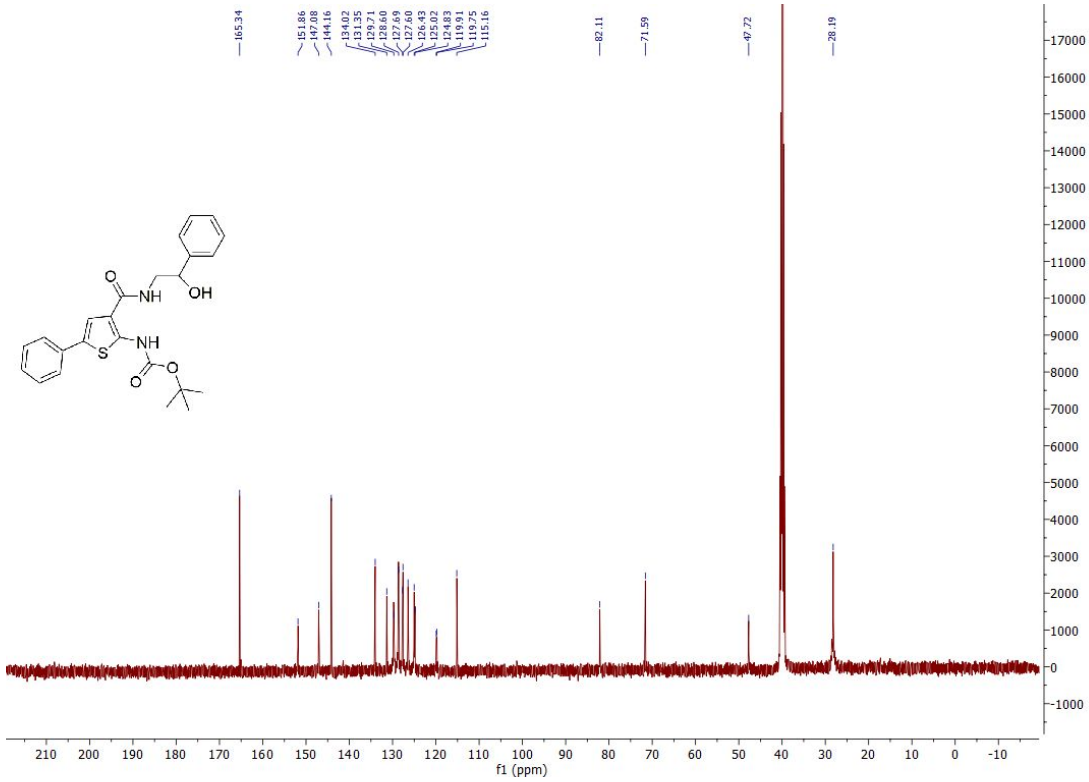

^{1}H NMR spectrum of **BDA-17** (500 MHz, $CDCl_3$)

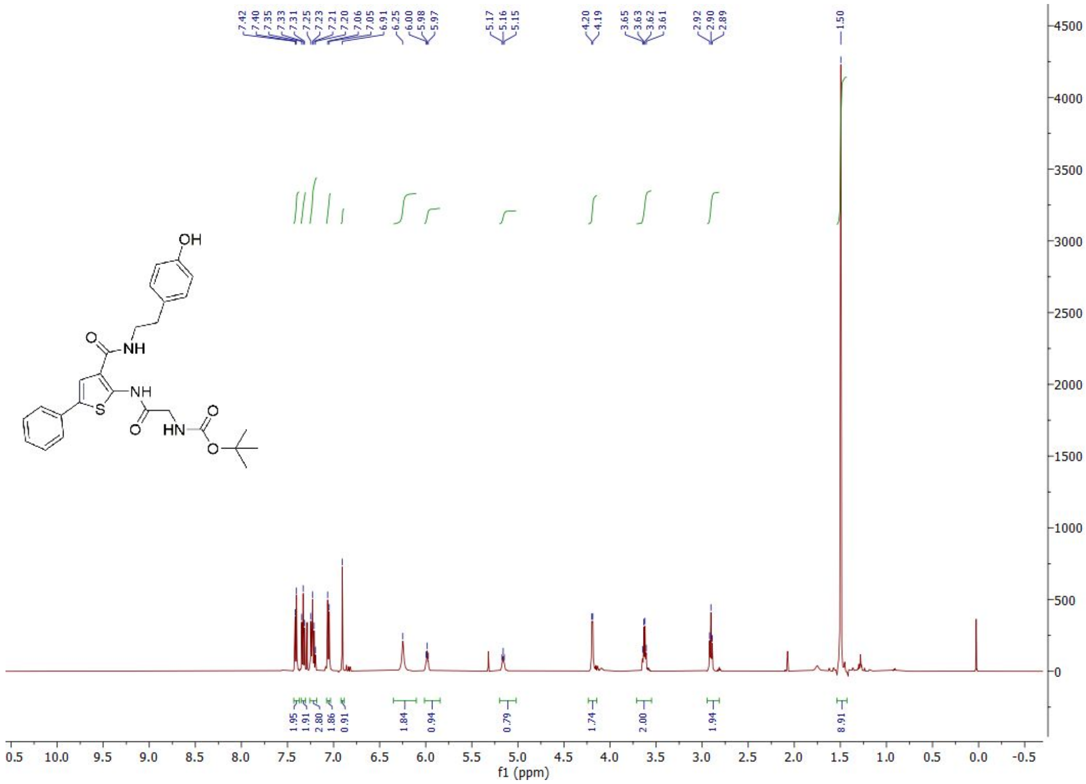

$^{13}C\{^{1}H\}$ NMR spectrum of **BDA-17** (126 MHz, $CDCl_3$)

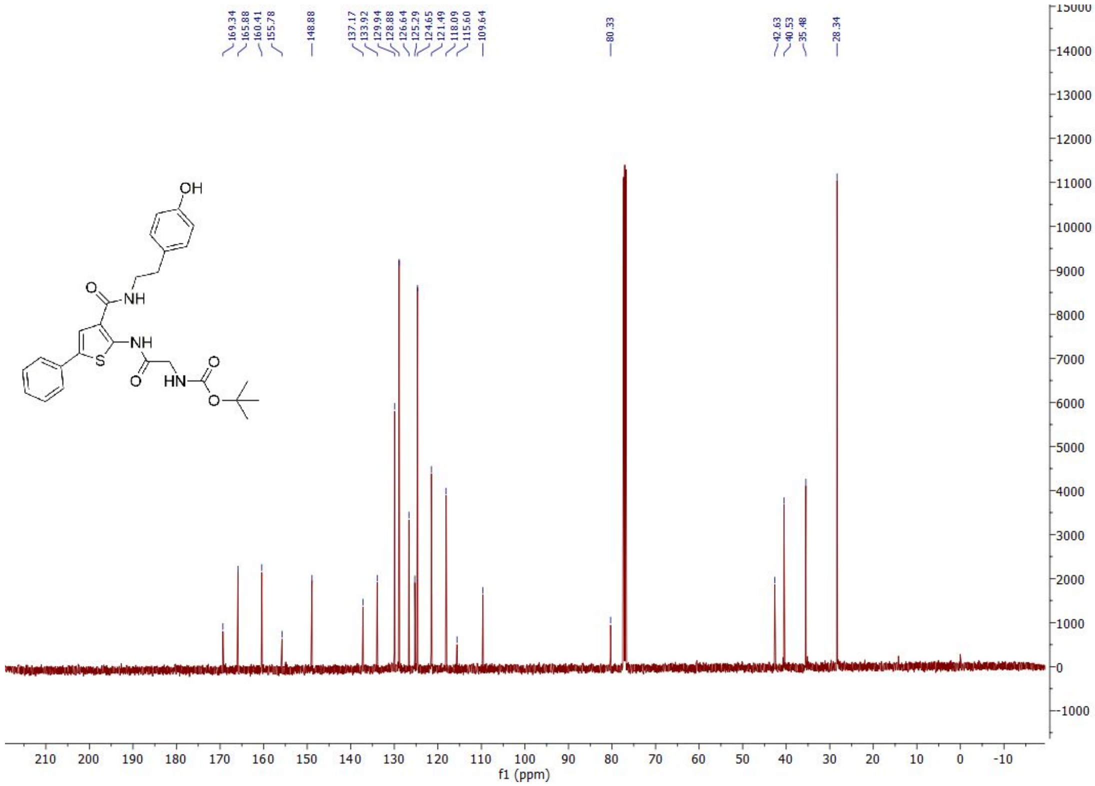

^{1}H NMR spectrum of **BDA-18** (500 MHz, $CDCl_3$)

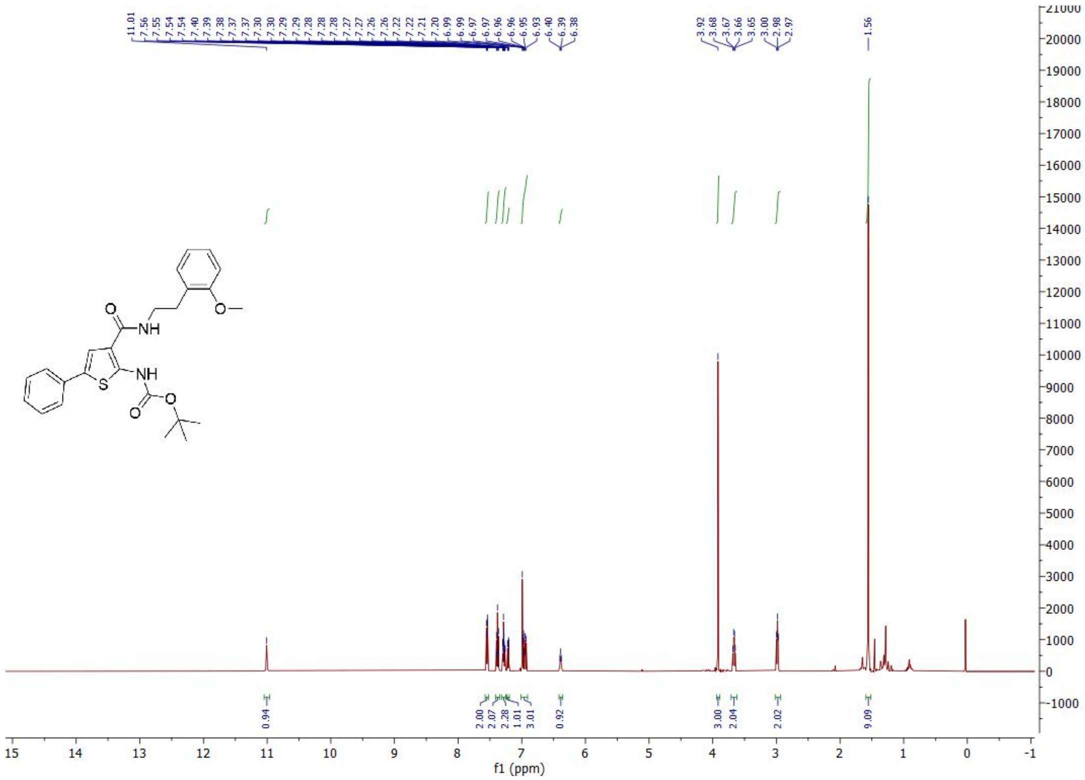

$^{13}C\{^{1}H\}$ NMR spectrum of **BDA-18** (126 MHz, $CDCl_3$)

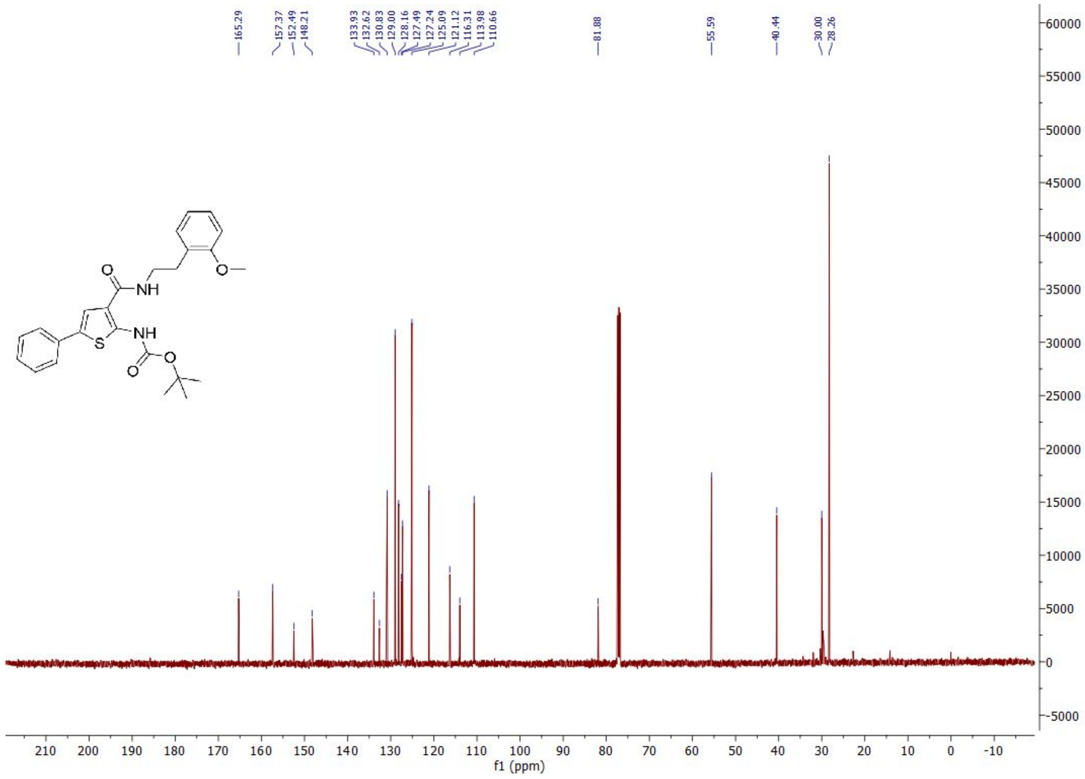

^{1}H NMR spectrum of **BDA-19** (500 MHz, $CDCl_3$)

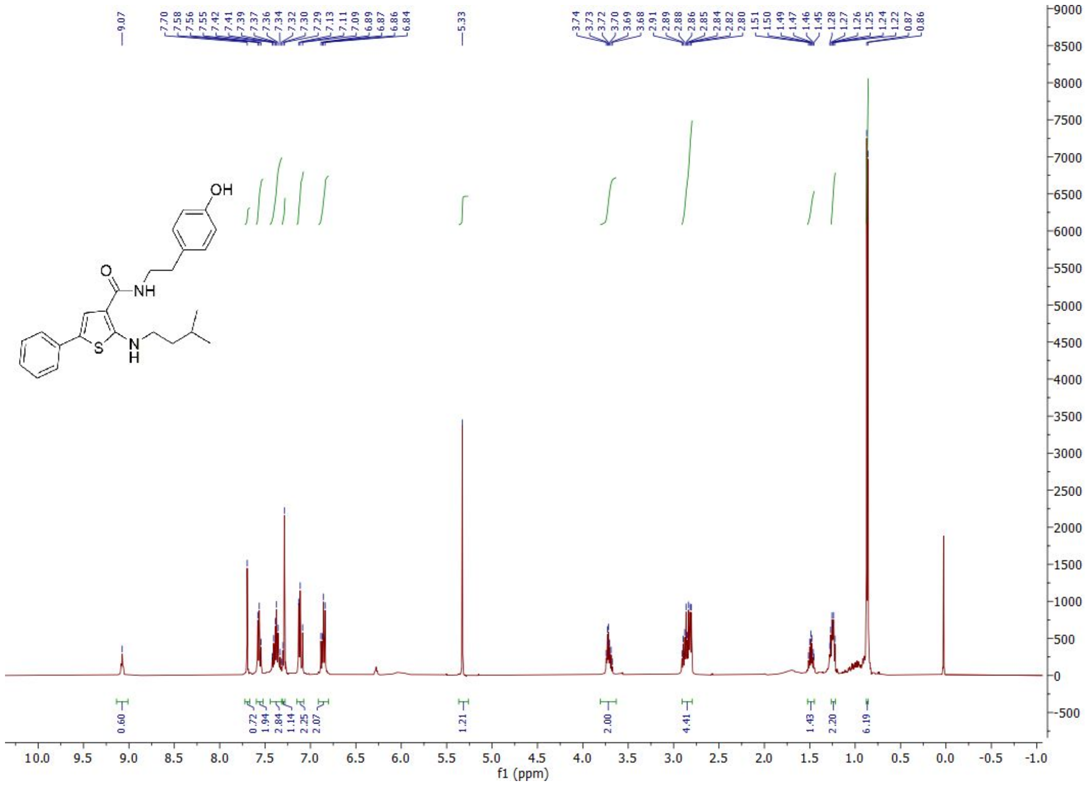

$^{13}C\{^{1}H\}$ NMR spectrum of **BDA-19** (126 MHz, $CDCl_3$)

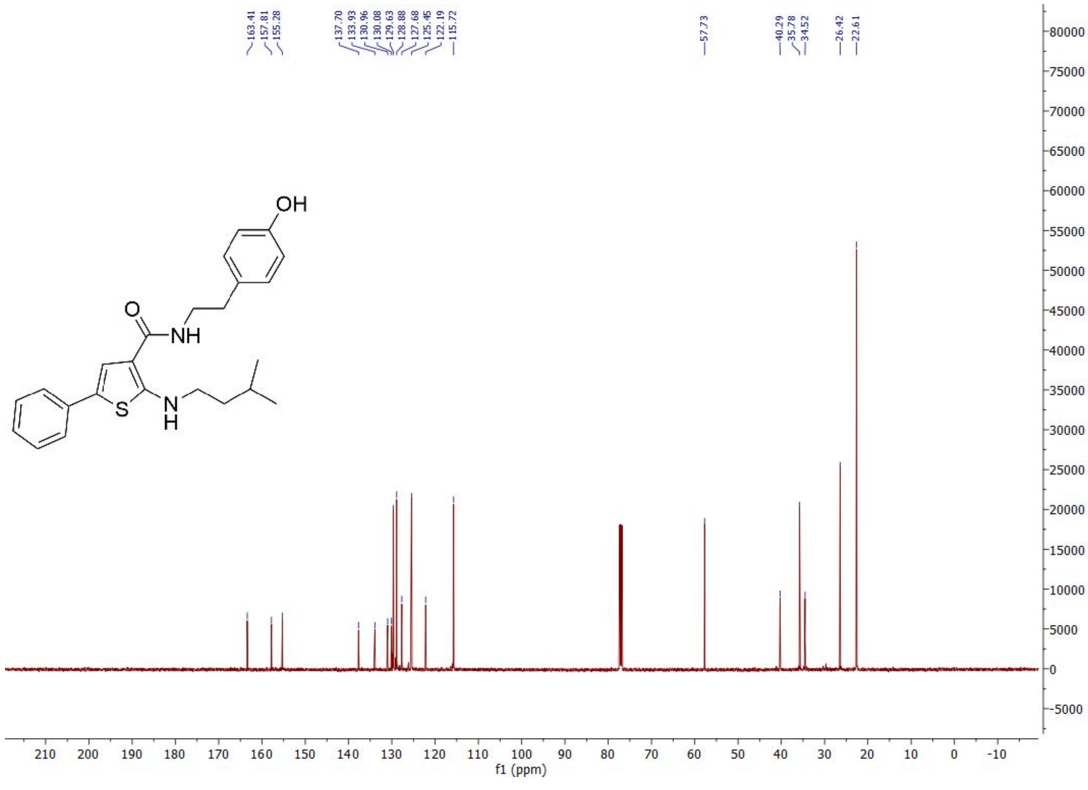

^{1}H NMR spectrum of **BDA-20** (500 MHz, $CDCl_3$)

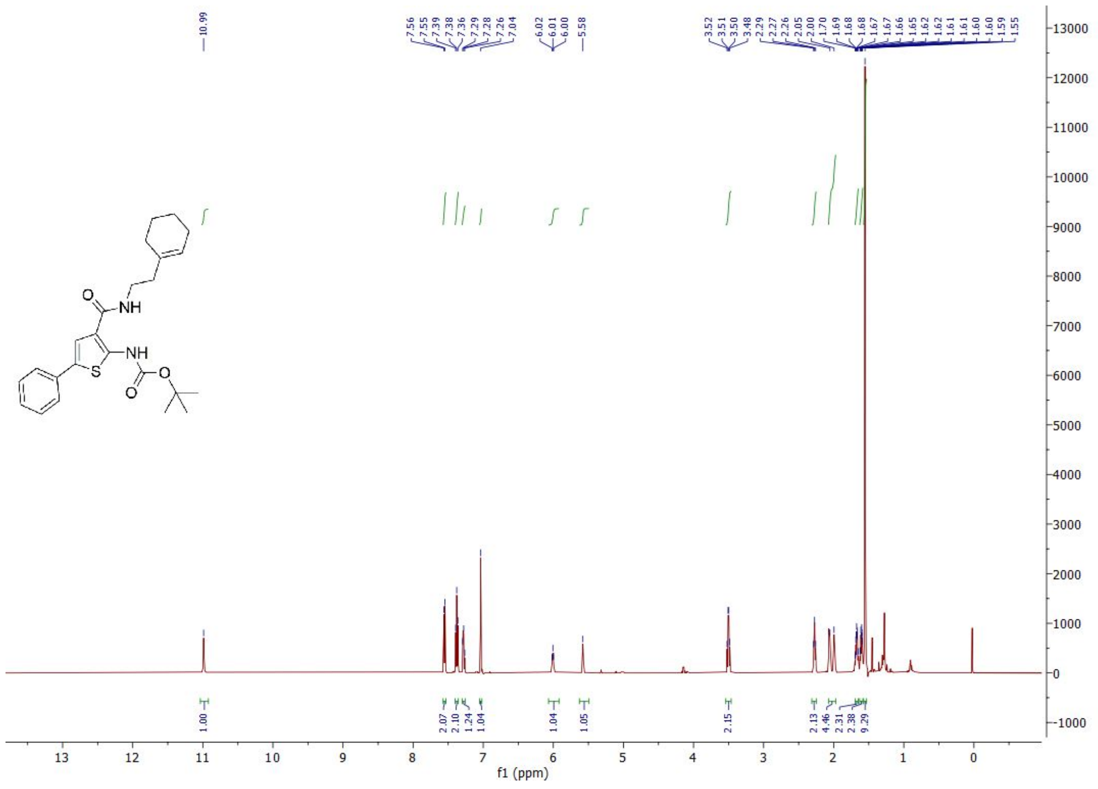

$^{13}C\{^{1}H\}$ NMR spectrum of **BDA-20** (126 MHz, $CDCl_3$)

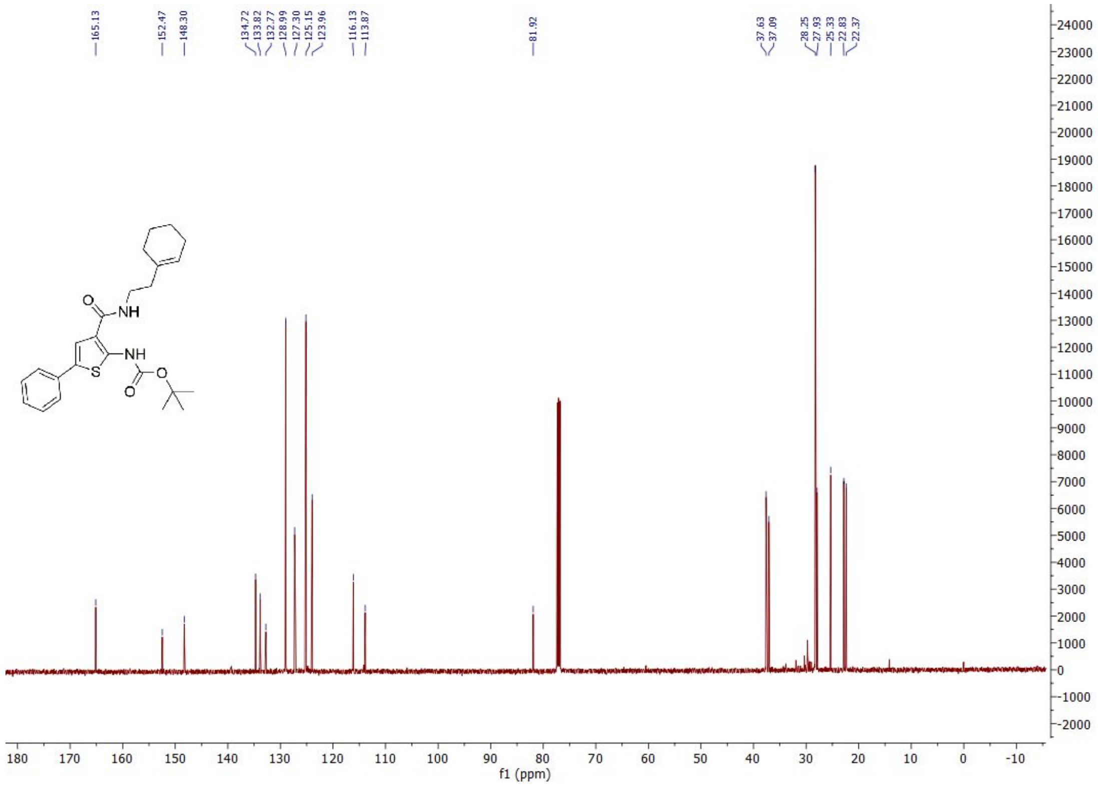

^{1}H NMR spectrum of **BDA-21** (500 MHz, $CDCl_3$)

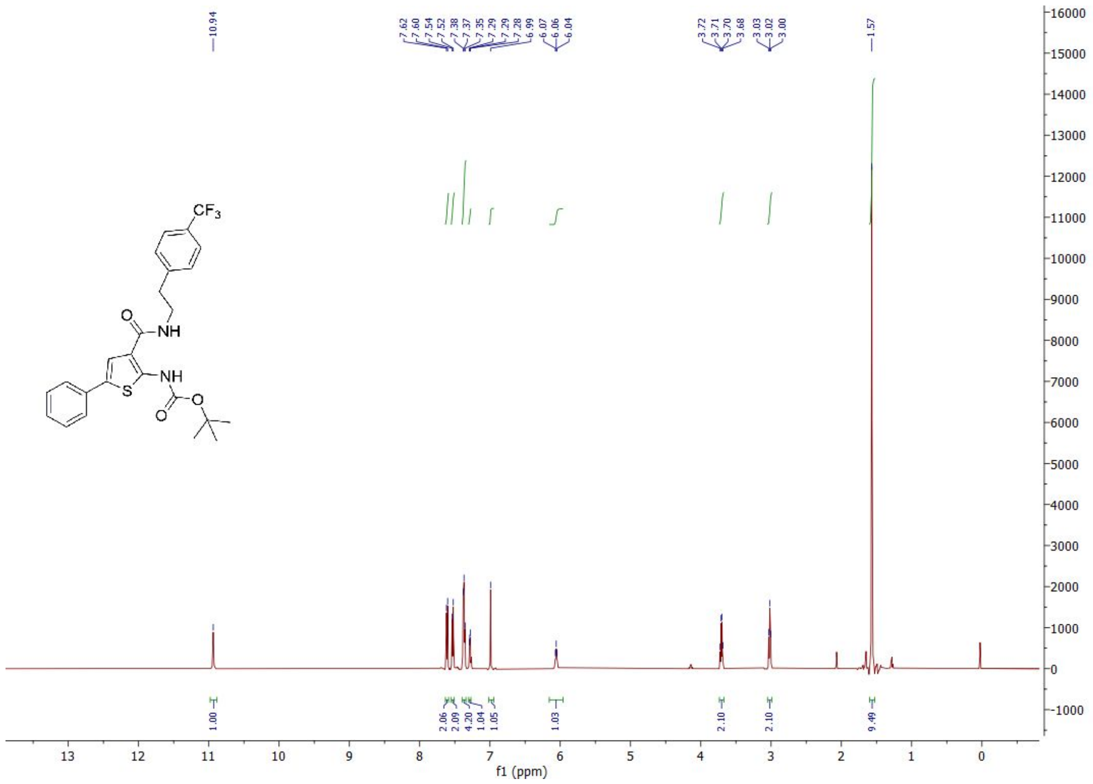

$^{13}C\{^{1}H\}$ NMR spectrum of **BDA-21** (126 MHz, $CDCl_3$)

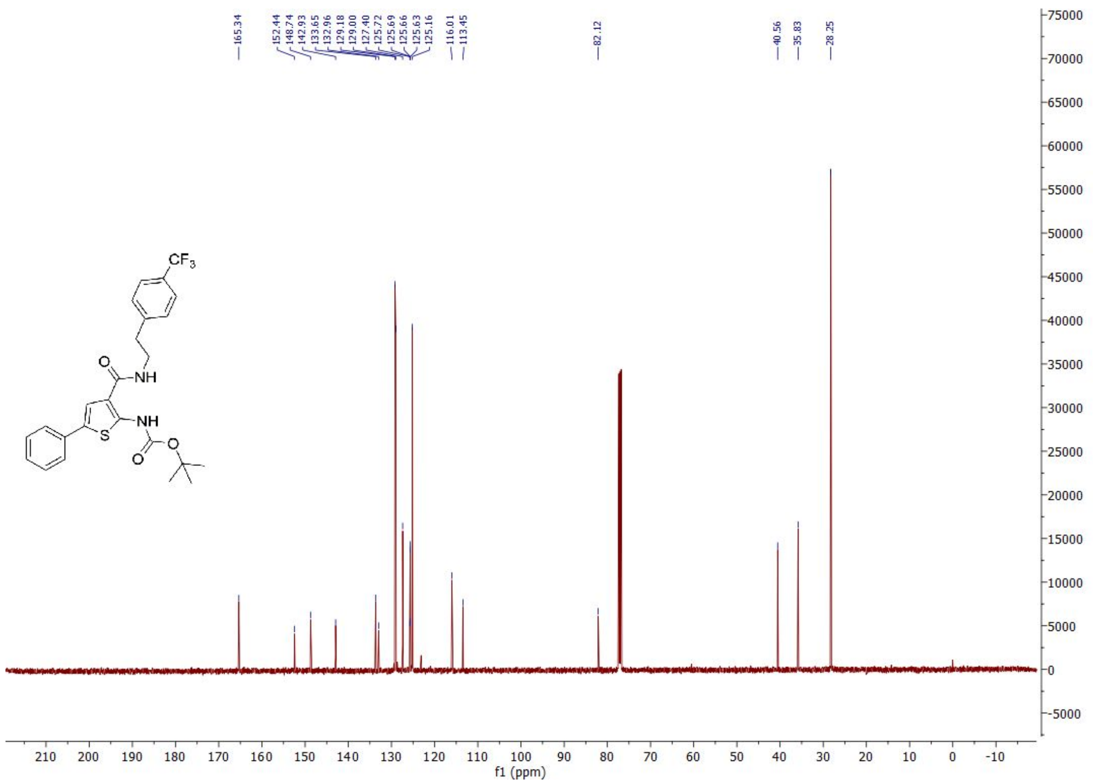

^{1}H NMR spectrum of **BDA-22** (500 MHz, $CDCl_3$)

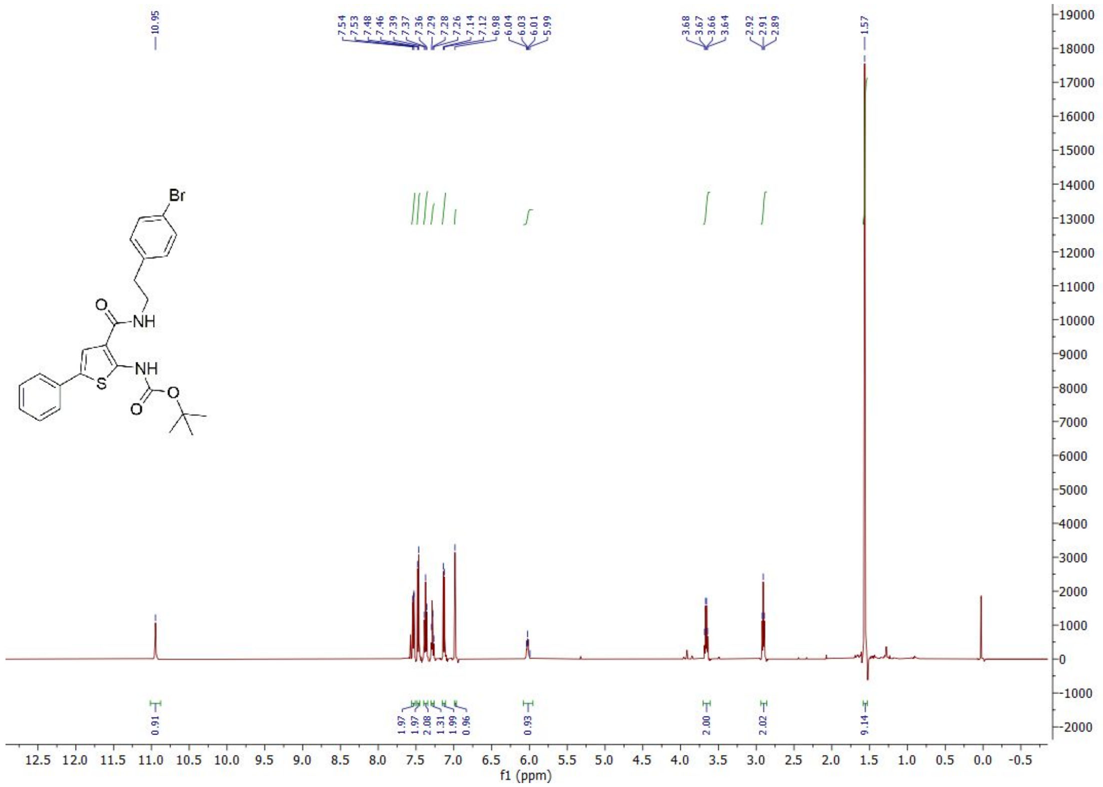

^{13}C{^{1}H} NMR spectrum of **BDA-22** (126 MHz, $CDCl_3$)

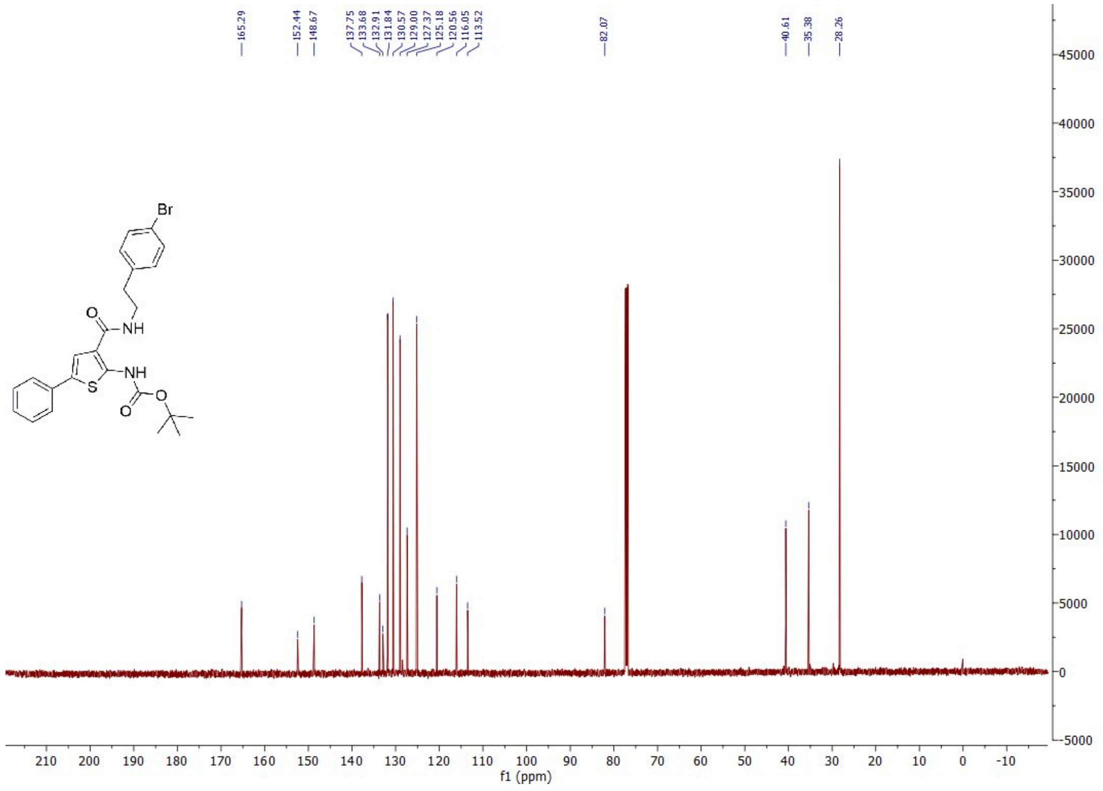

^{1}H NMR spectrum of **BDA-23** (500 MHz, $CDCl_3$)

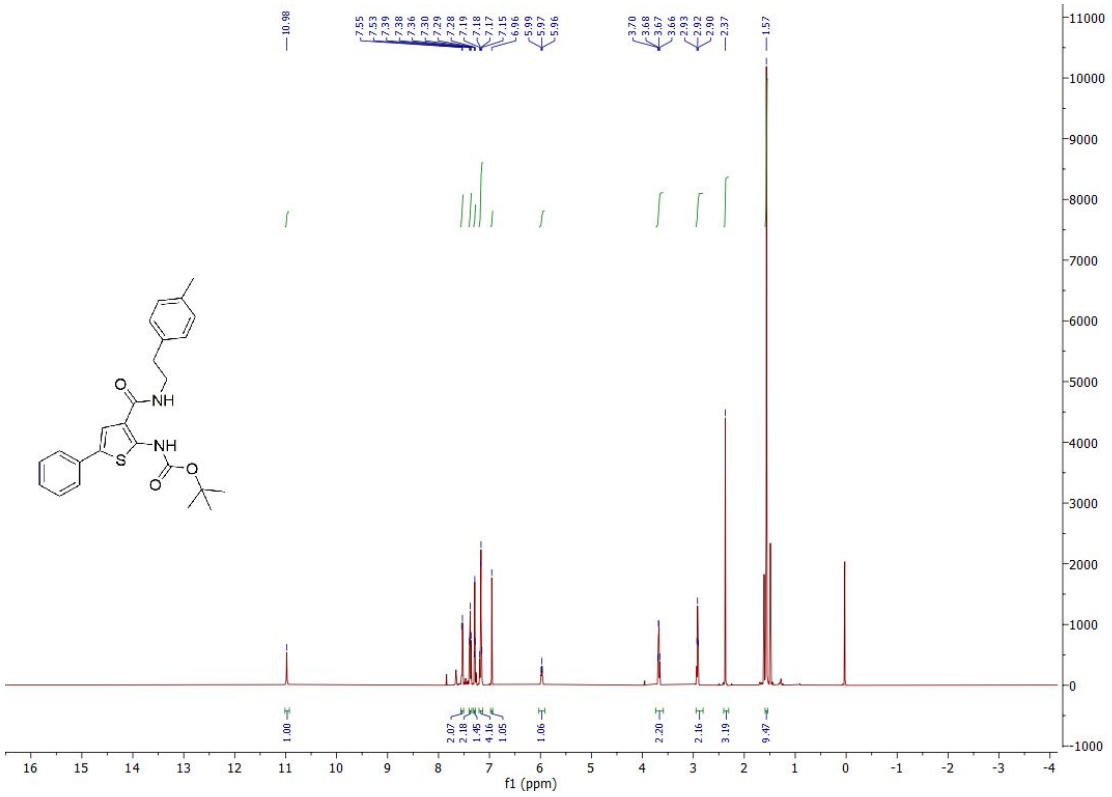

$^{13}C\{^{1}H\}$ NMR spectrum of **BDA-23** (126 MHz, $CDCl_3$)

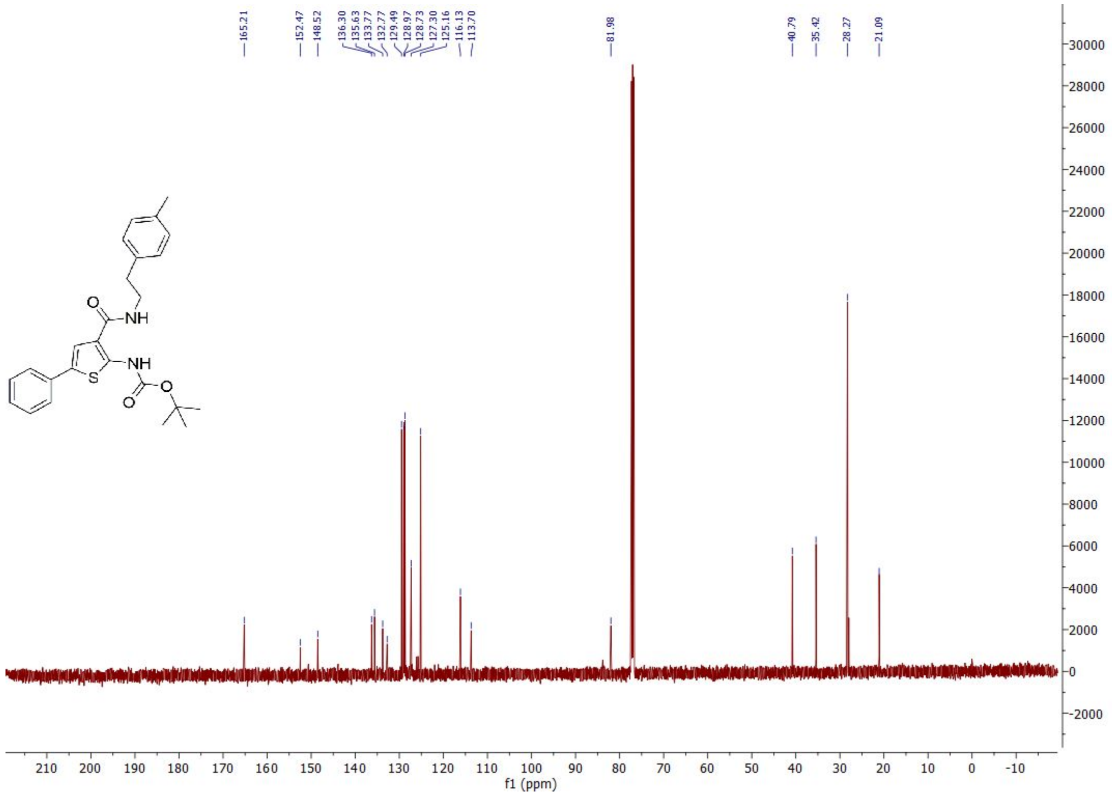

^{1}H NMR spectrum of **BDA-24** (500 MHz, $CDCl_3$)

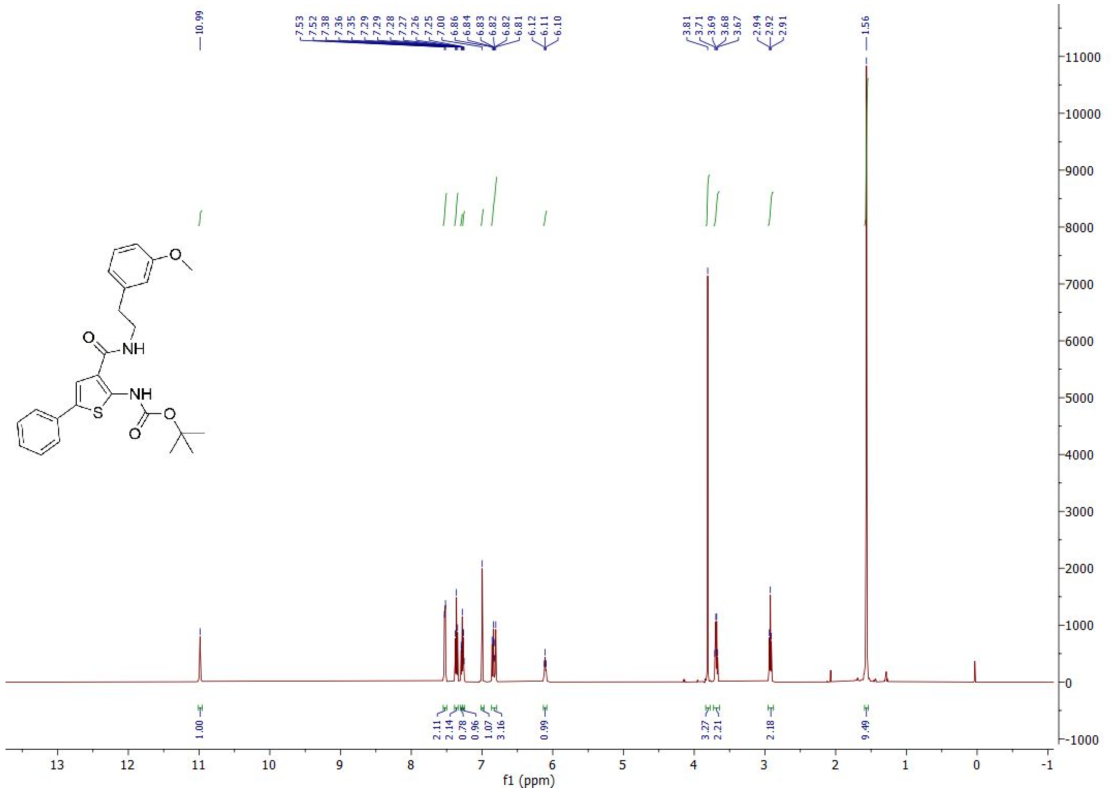

$^{13}C\{^{1}H\}$ NMR spectrum of **BDA-24** (126 MHz, $CDCl_3$)

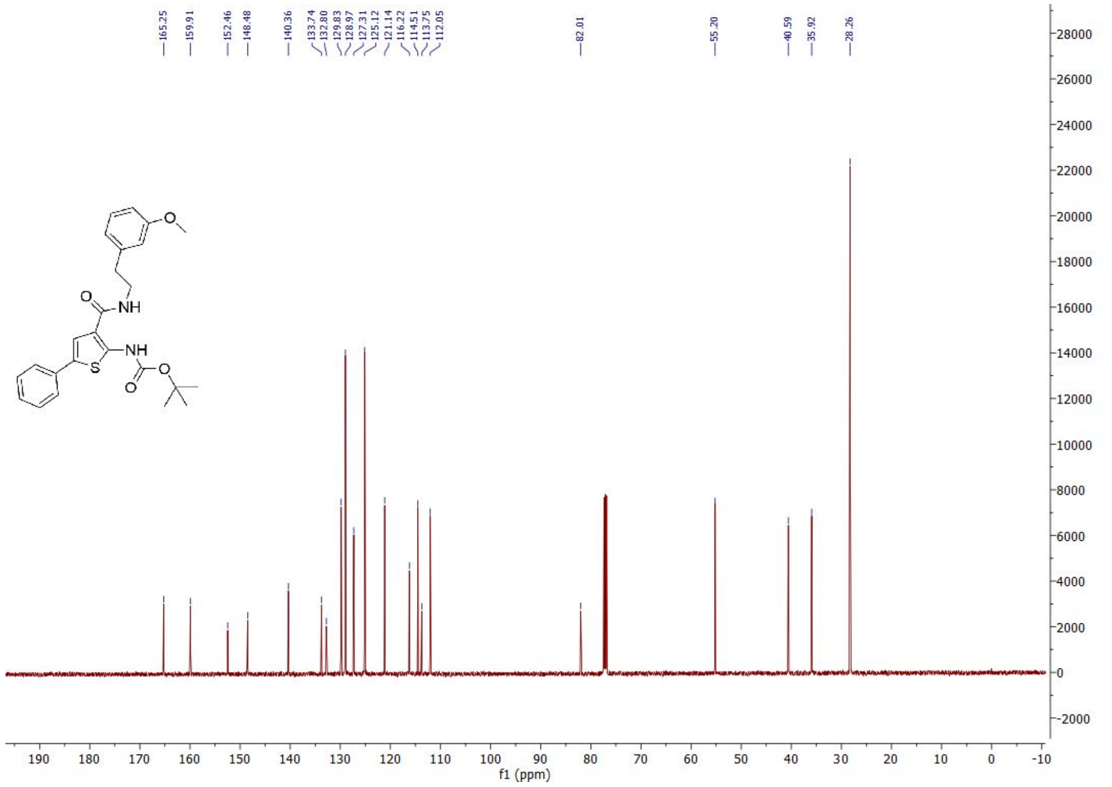

^{1}H NMR spectrum of **BDA-25** (500 MHz, $CDCl_3$)

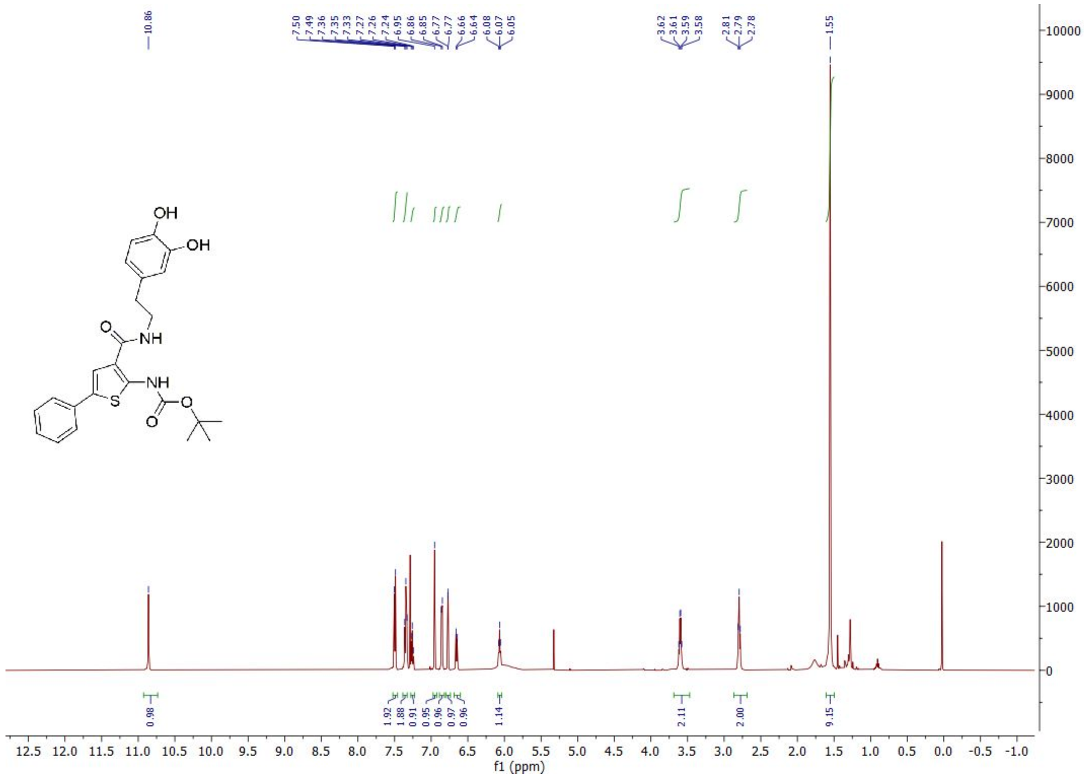

$^{13}C\{^{1}H\}$ NMR spectrum of **BDA-25** (126 MHz, $CDCl_3$)

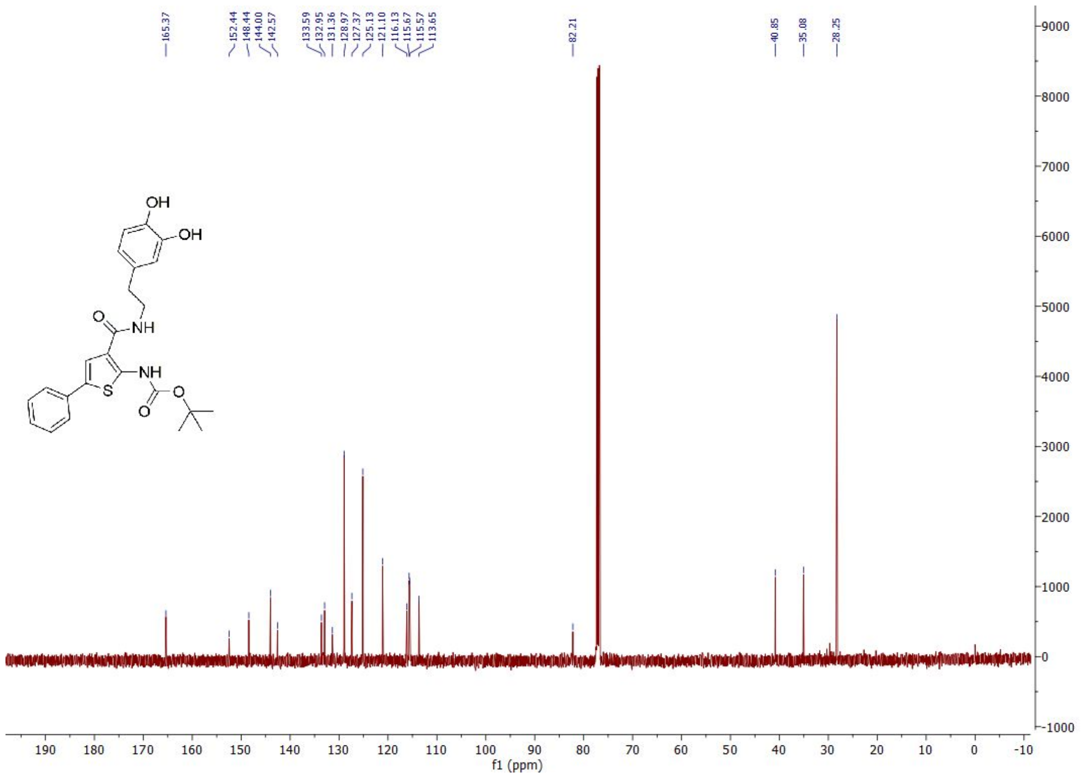

^{1}H NMR spectrum of **BDA-26** (500 MHz, $CDCl_3$)

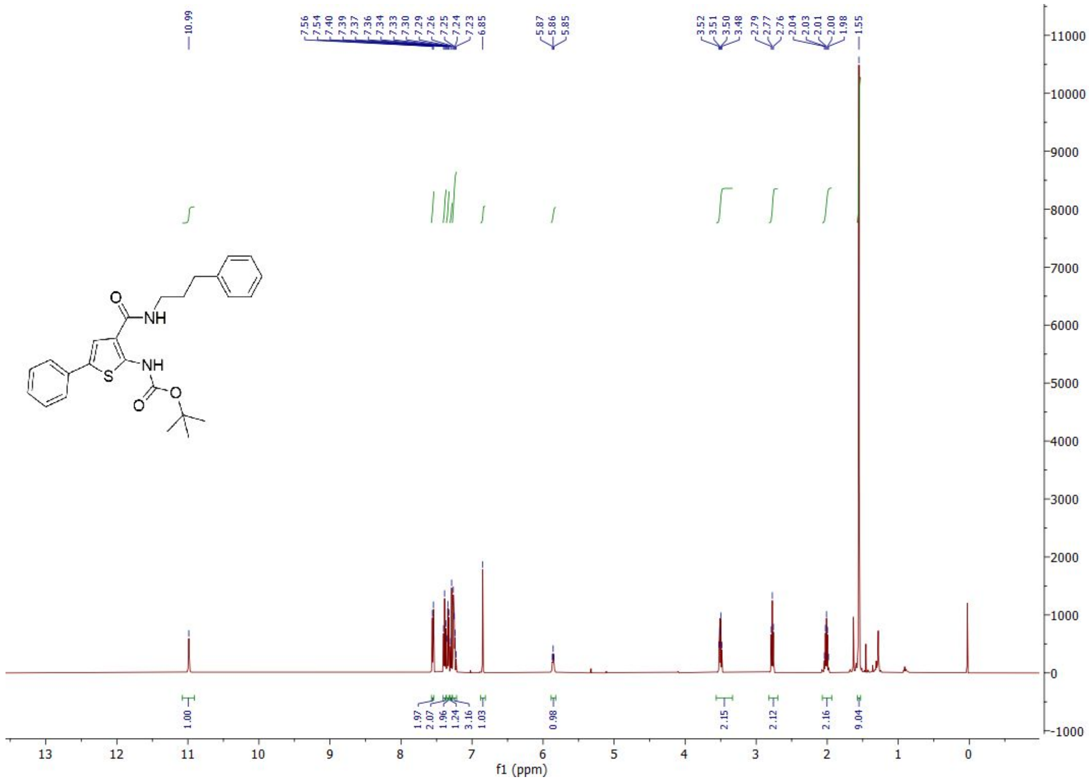

$^{13}C\{^1H\}$ NMR spectrum of **BDA-26** (126 MHz, $CDCl_3$)

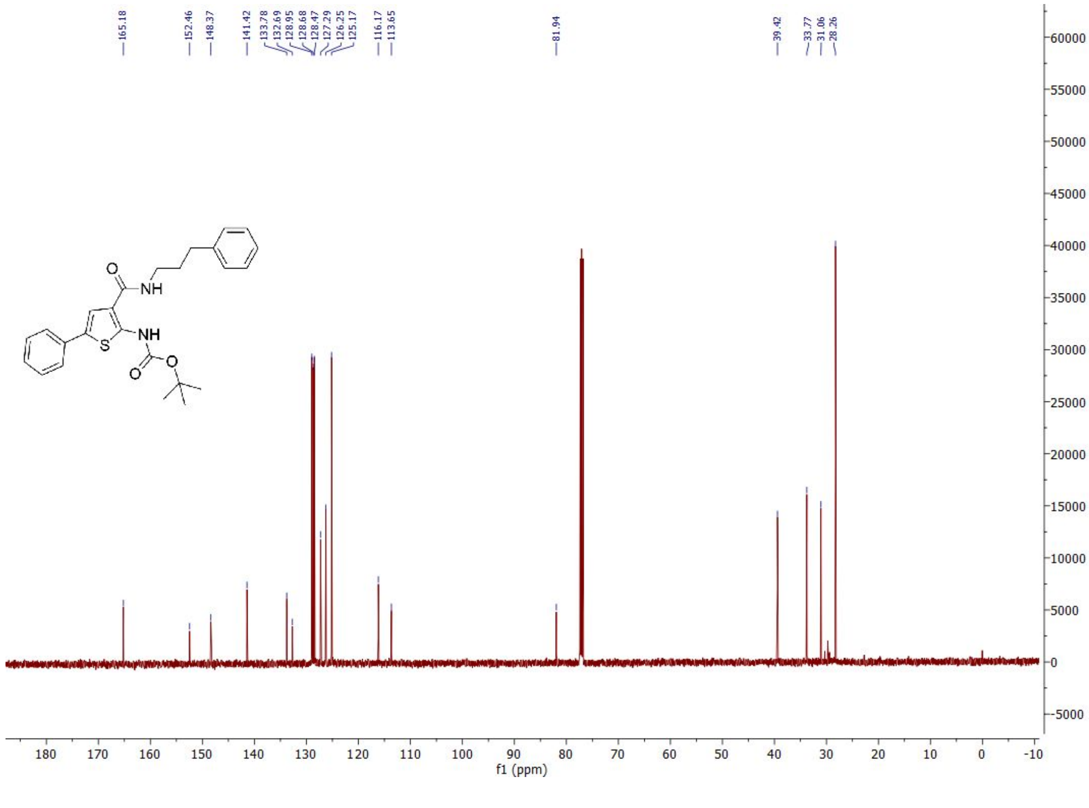

^{1}H NMR spectrum of **BDA-27** (500 MHz, $CDCl_3$)

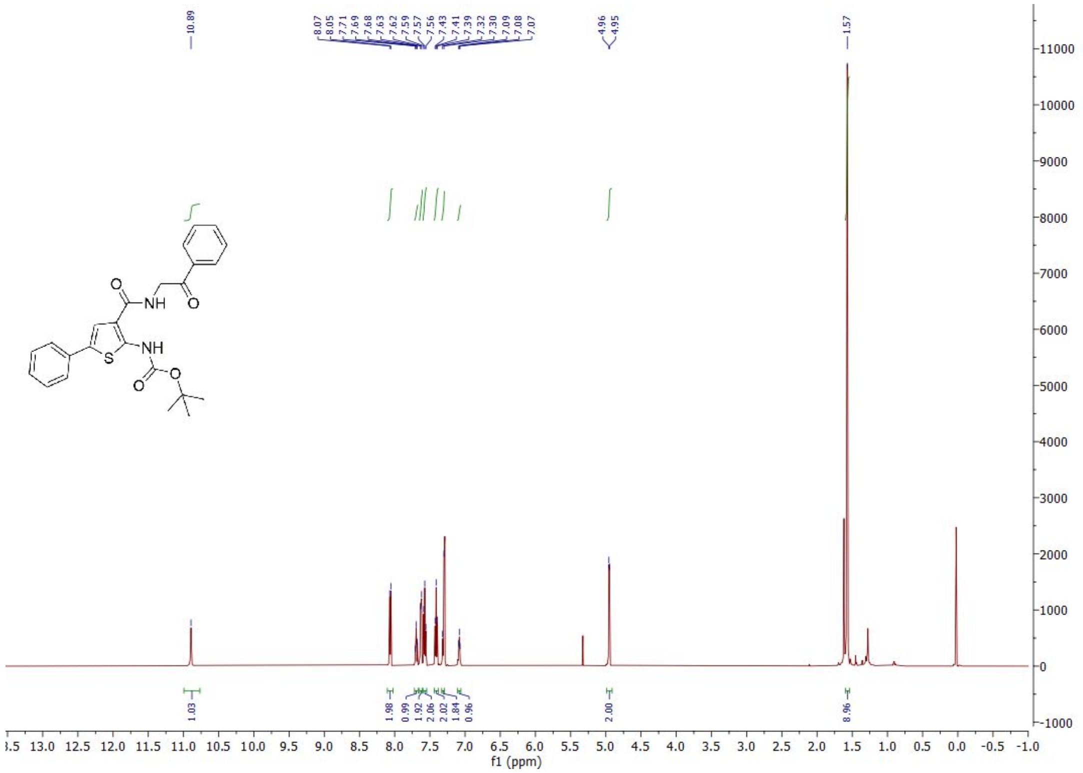

$^{13}C\{^{1}H\}$ NMR spectrum of **BDA-27** (126 MHz, $CDCl_3$)

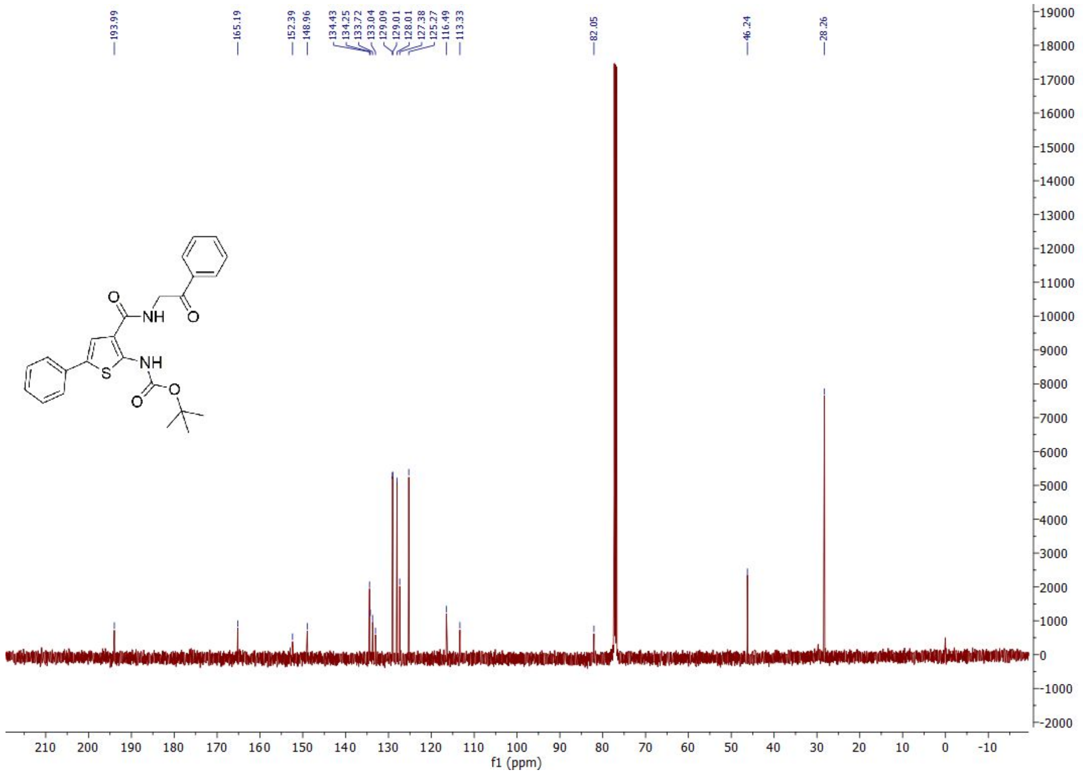

^{1}H NMR spectrum of **BDA-28** (500 MHz, $CDCl_3$)

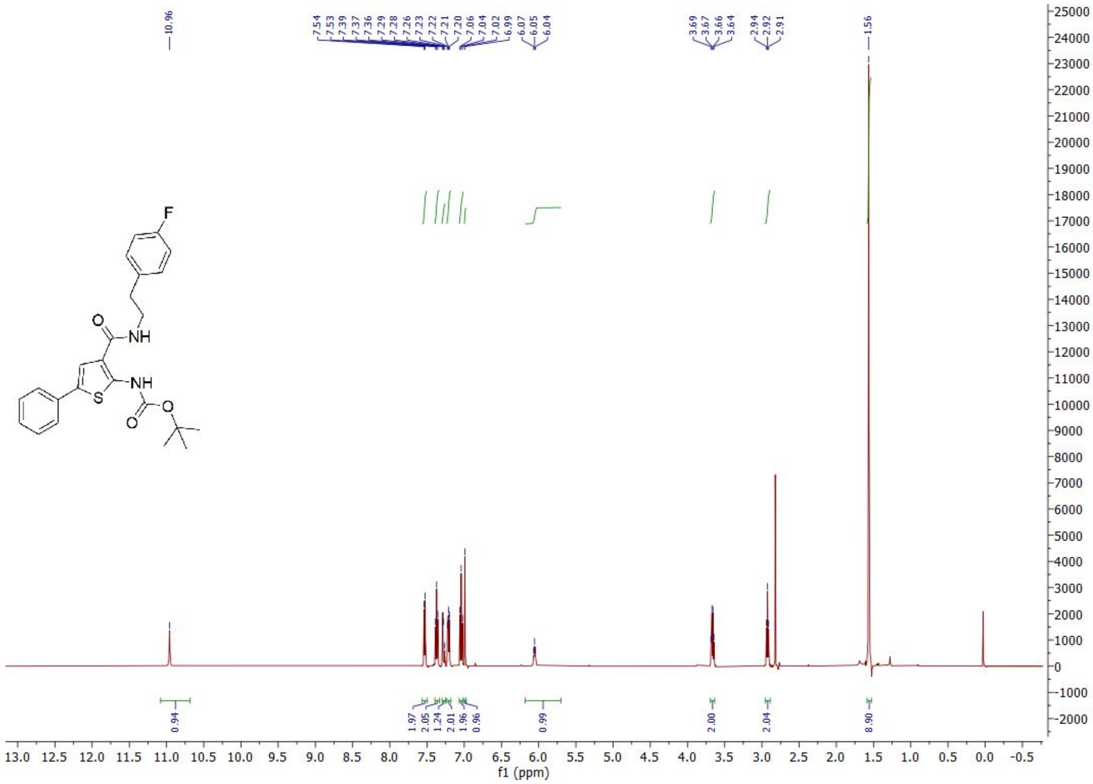

$^{13}C\{^{1}H\}$ NMR spectrum of **BDA-28** (126 MHz, $CDCl_3$)

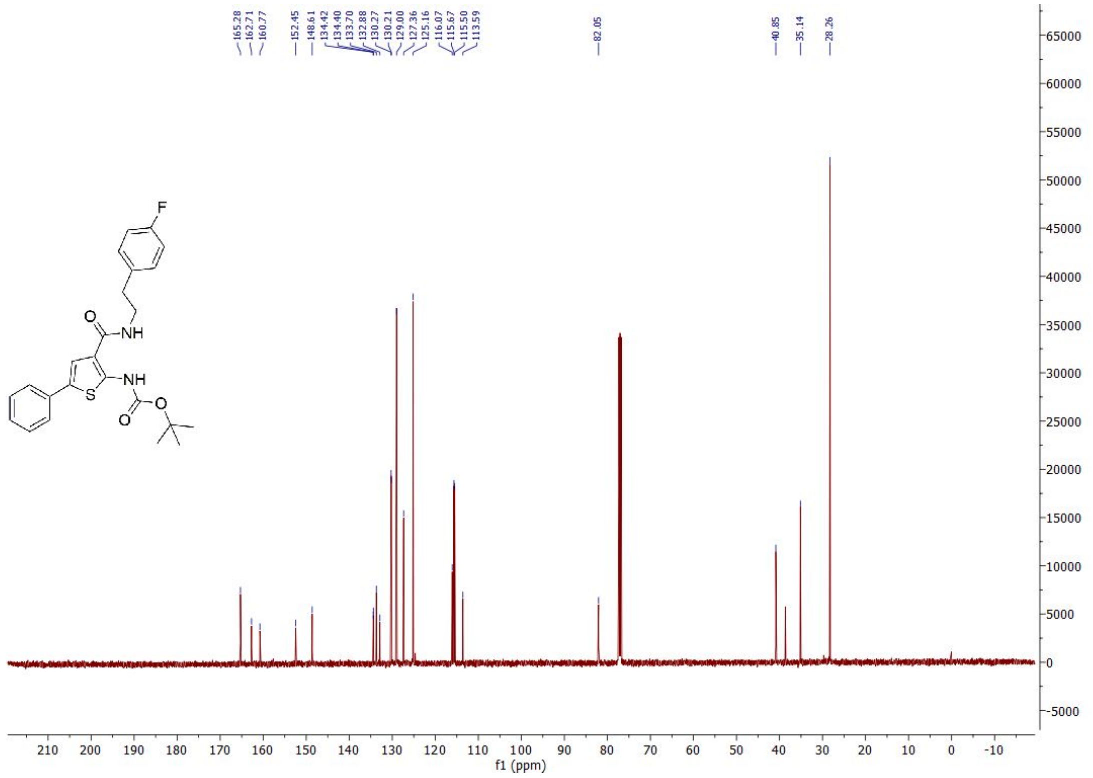

^{1}H NMR spectrum of **BDA-29** (500 MHz, $CDCl_3$)

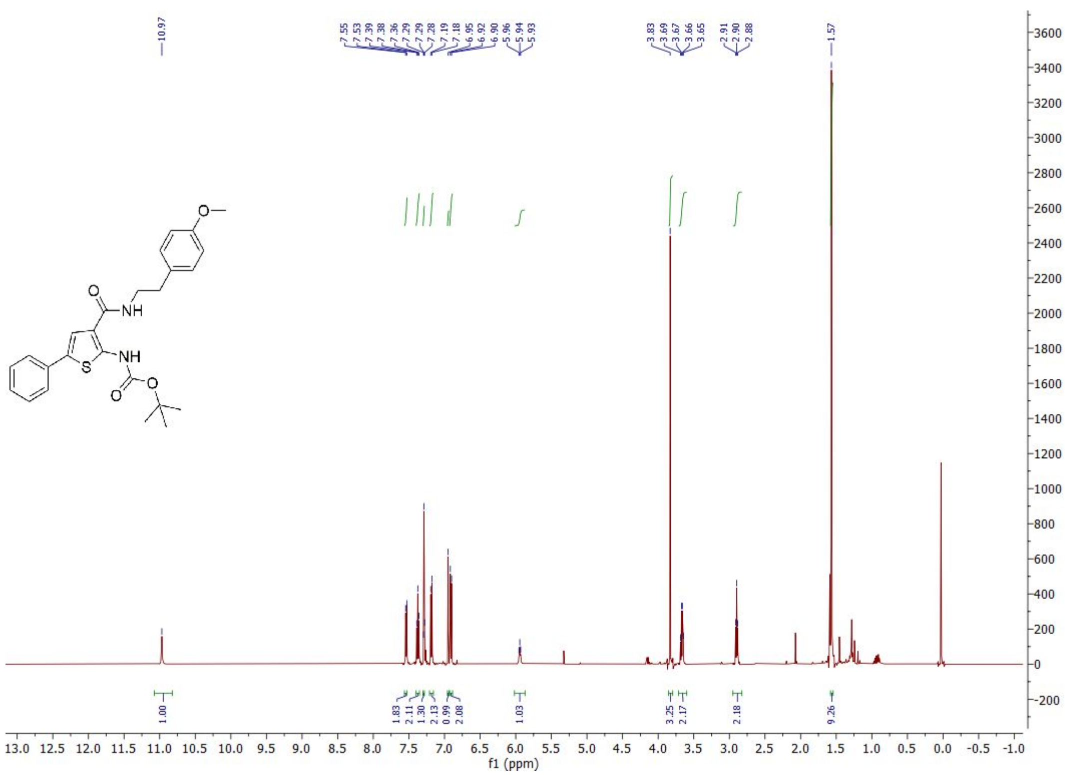

^{13}C{^{1}H} NMR spectrum of **BDA-29** (126 MHz, $CDCl_3$)

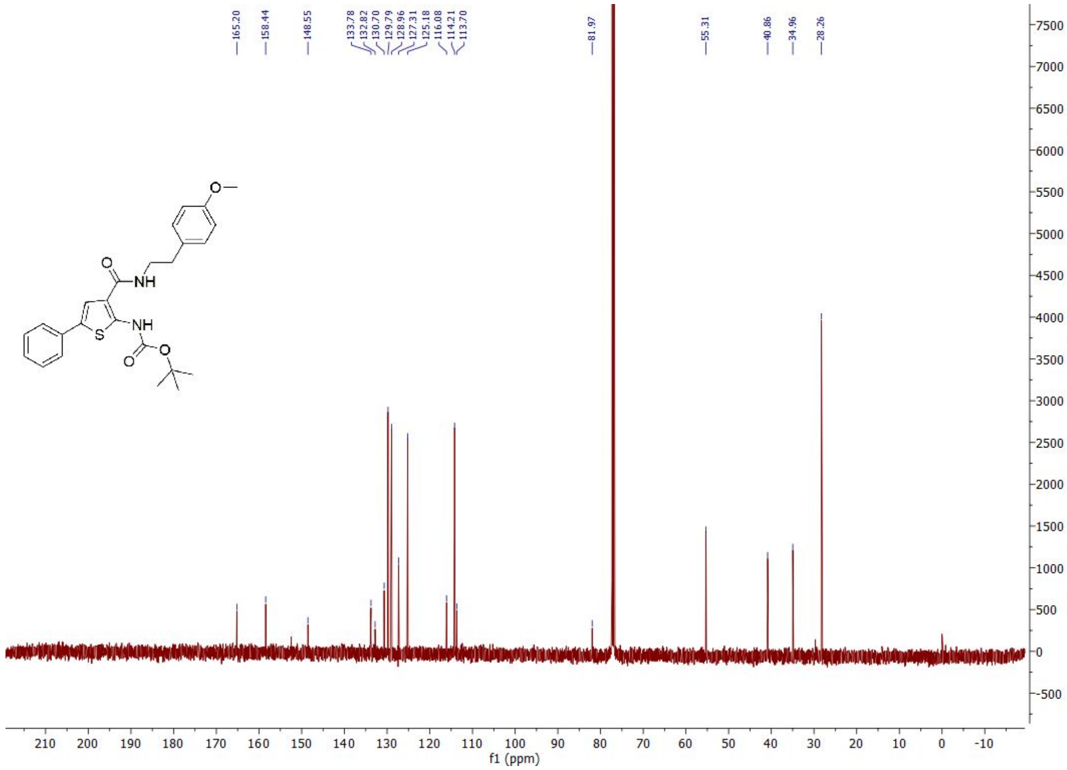

^{1}H NMR spectrum of **BDA-30** (500 MHz, $CDCl_3$)

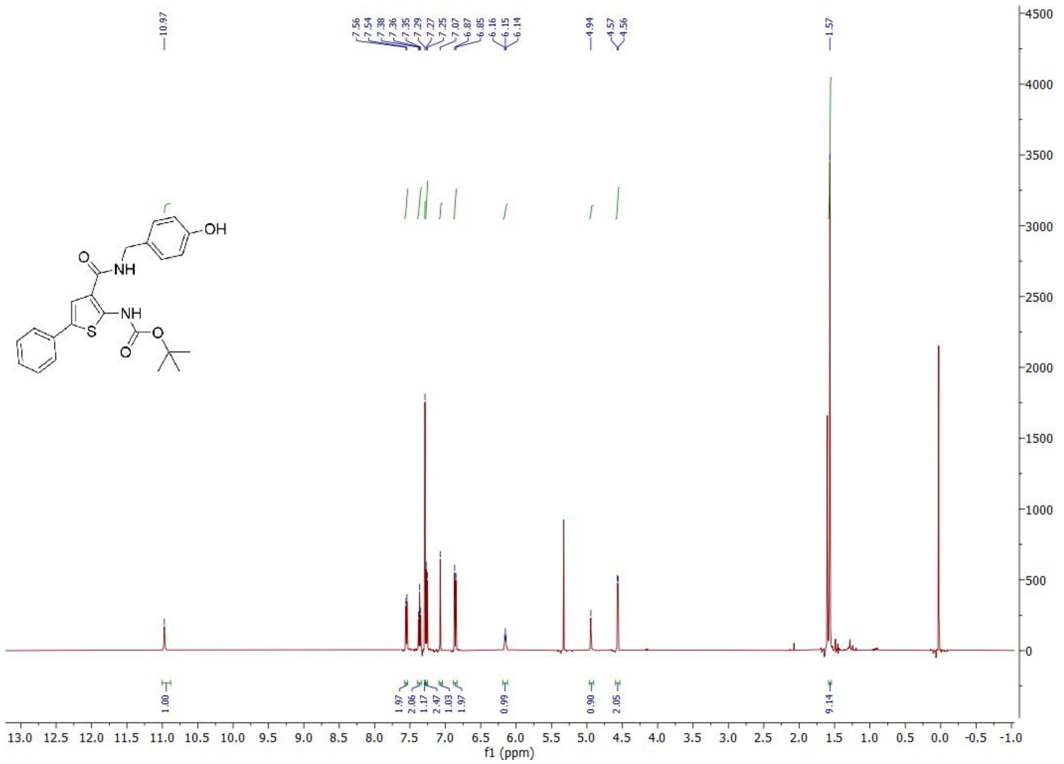

$^{13}C\{^{1}H\}$ NMR spectrum of **BDA-30** (126 MHz, $CDCl_3$)

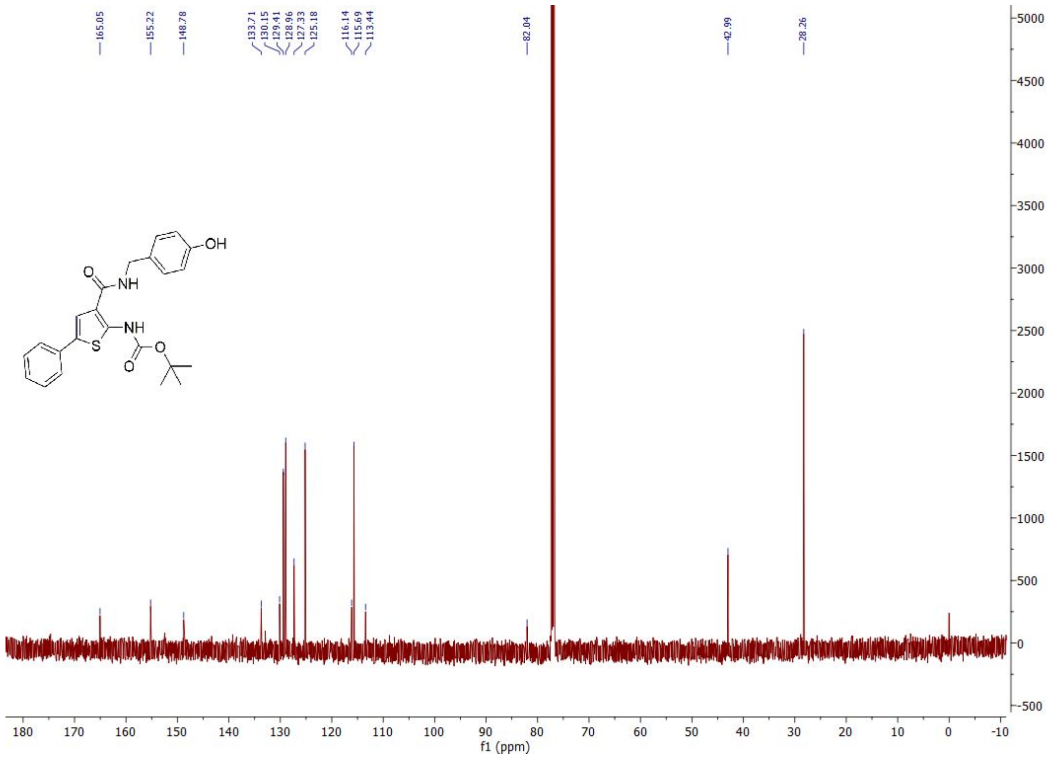

^{1}H NMR spectrum of **BDA-31** (500 MHz, $CDCl_3$)

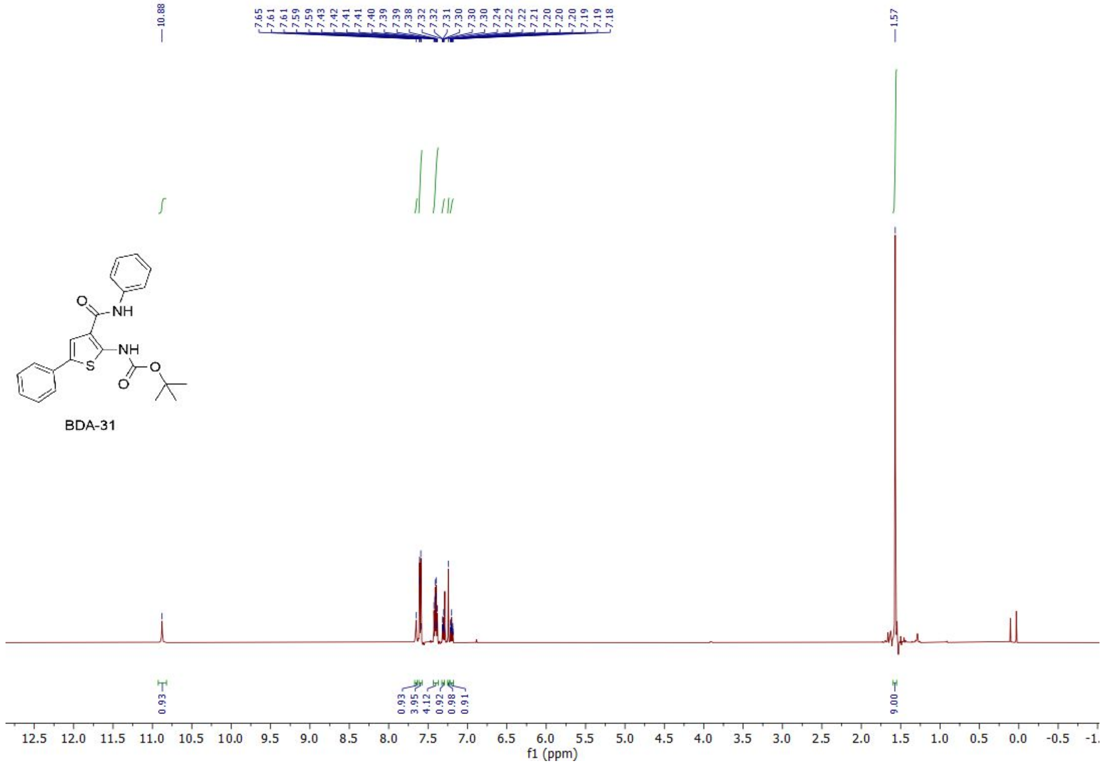

$^{13}C\{^1H\}$ NMR spectrum of **BDA-31** (126 MHz, $CDCl_3$)

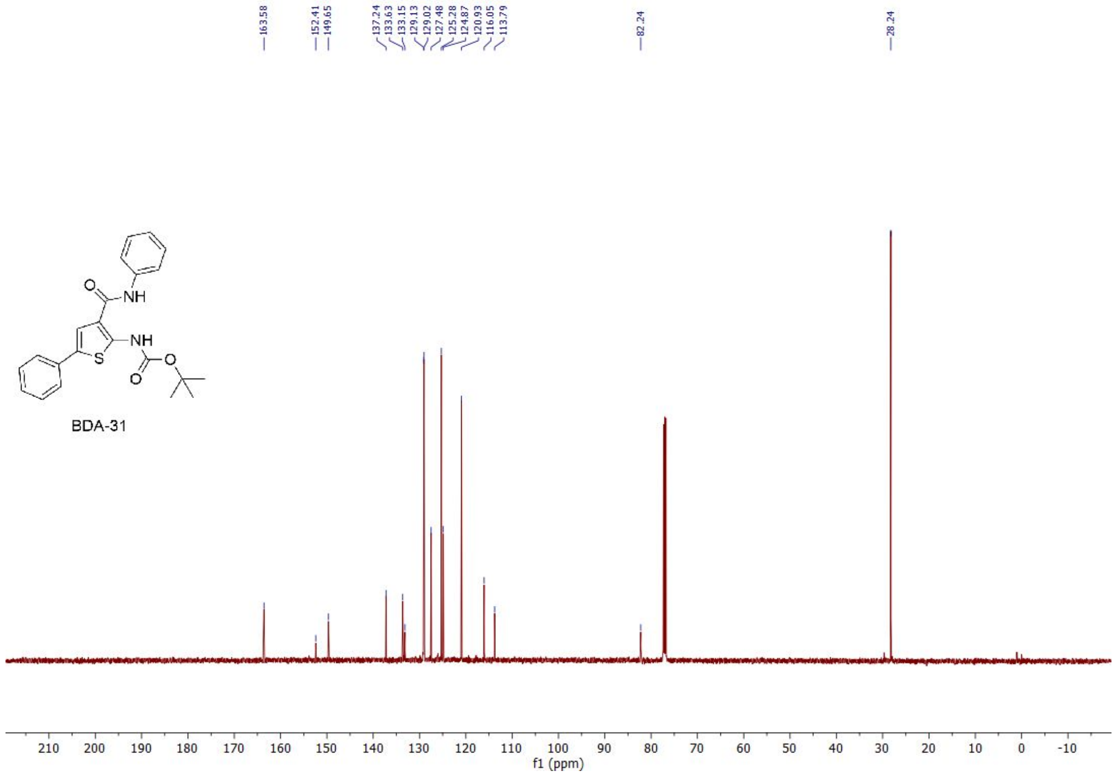

^{1}H NMR spectrum of **BDA-32** (500 MHz, $CDCl_3$)

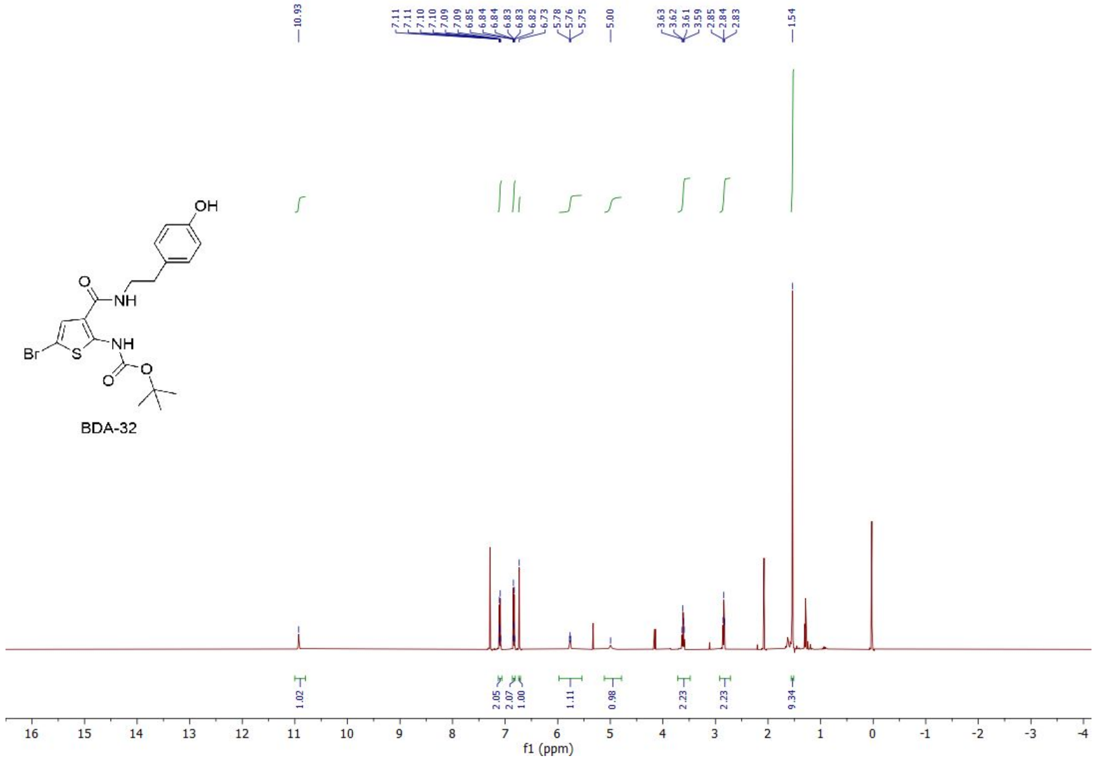

$^{13}C\{^{1}H\}$ NMR spectrum of **BDA-32** (126 MHz, $CDCl_3$)

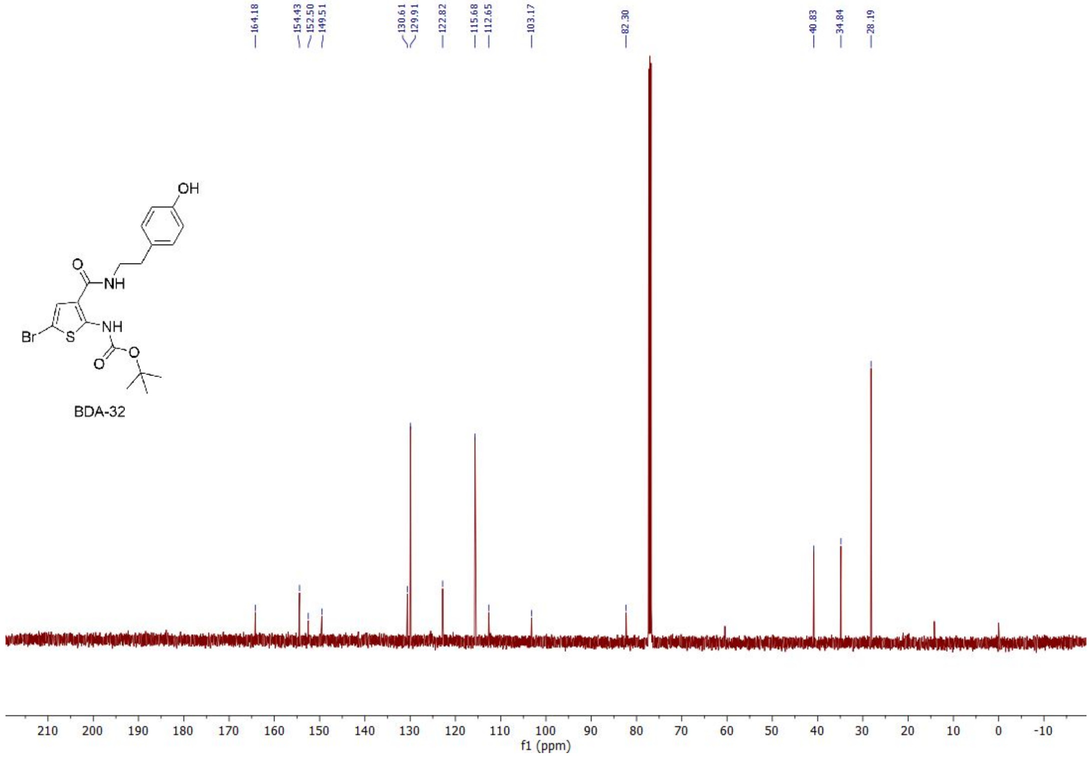

^{1}H NMR spectrum of **BDA-33** (500 MHz, $CDCl_3$)

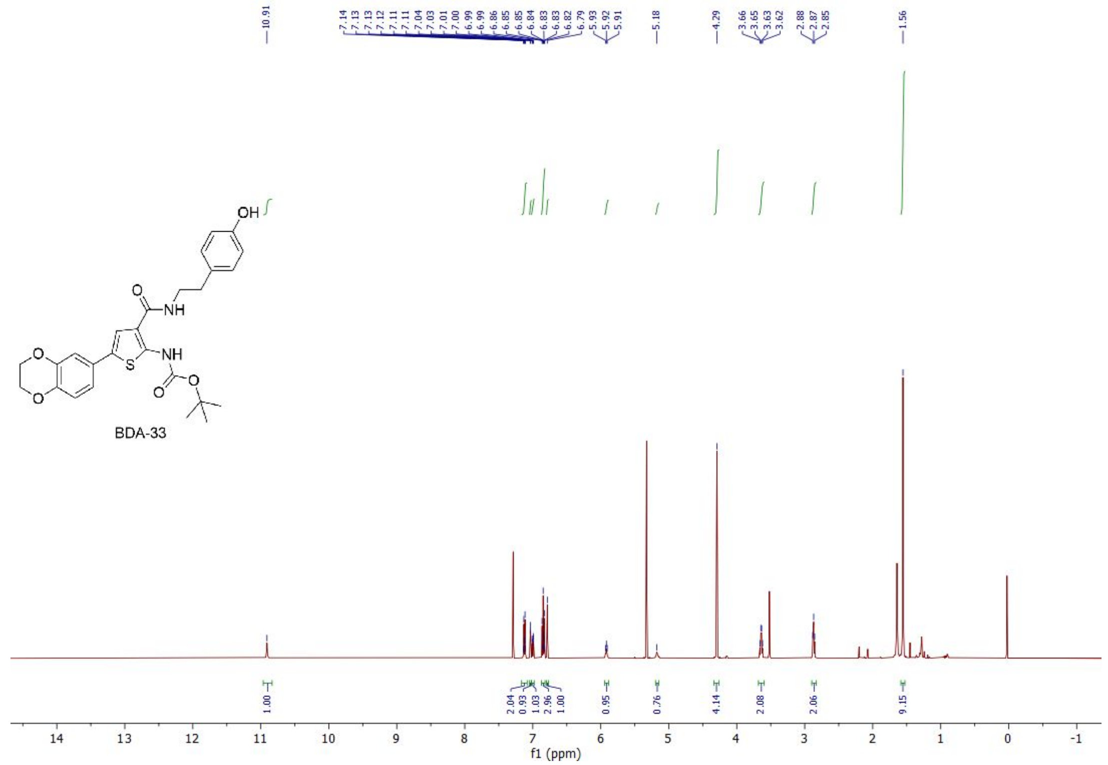

$^{13}C\{^{1}H\}$ NMR spectrum of **BDA-33** (126 MHz, $CDCl_3$)

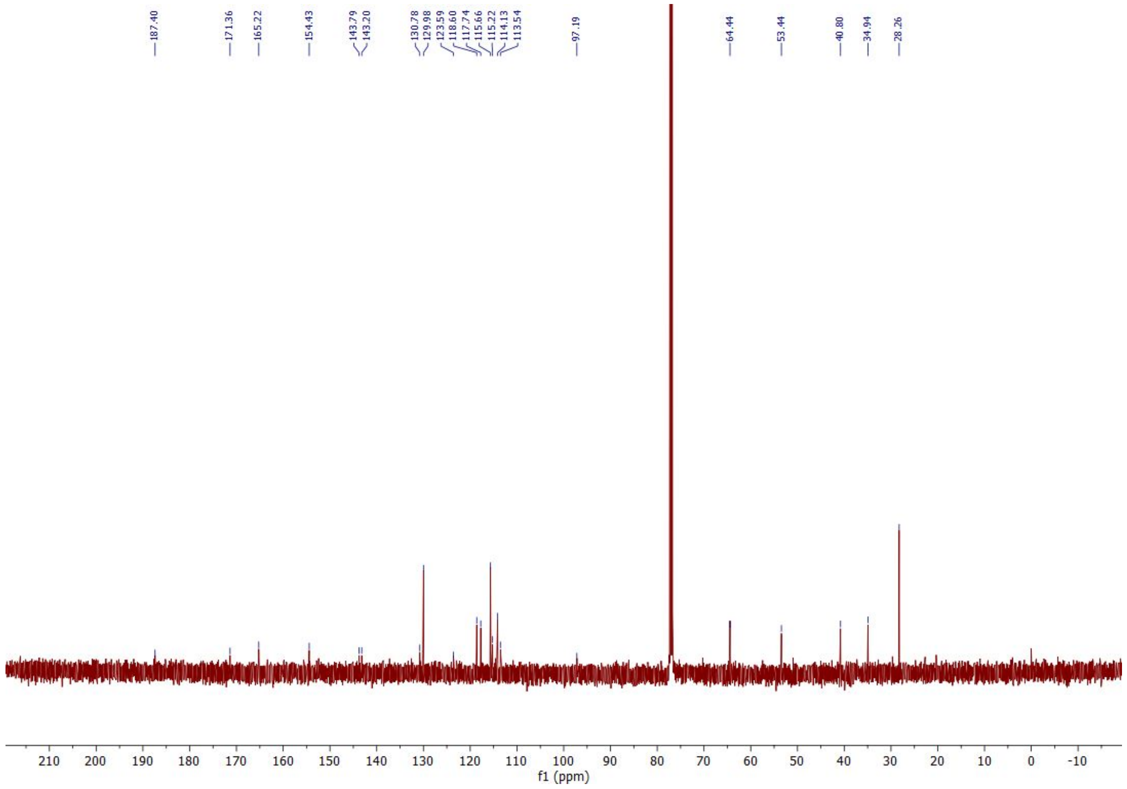

^{1}H NMR spectrum of **BDA-34** (500 MHz, $CDCl_3$)

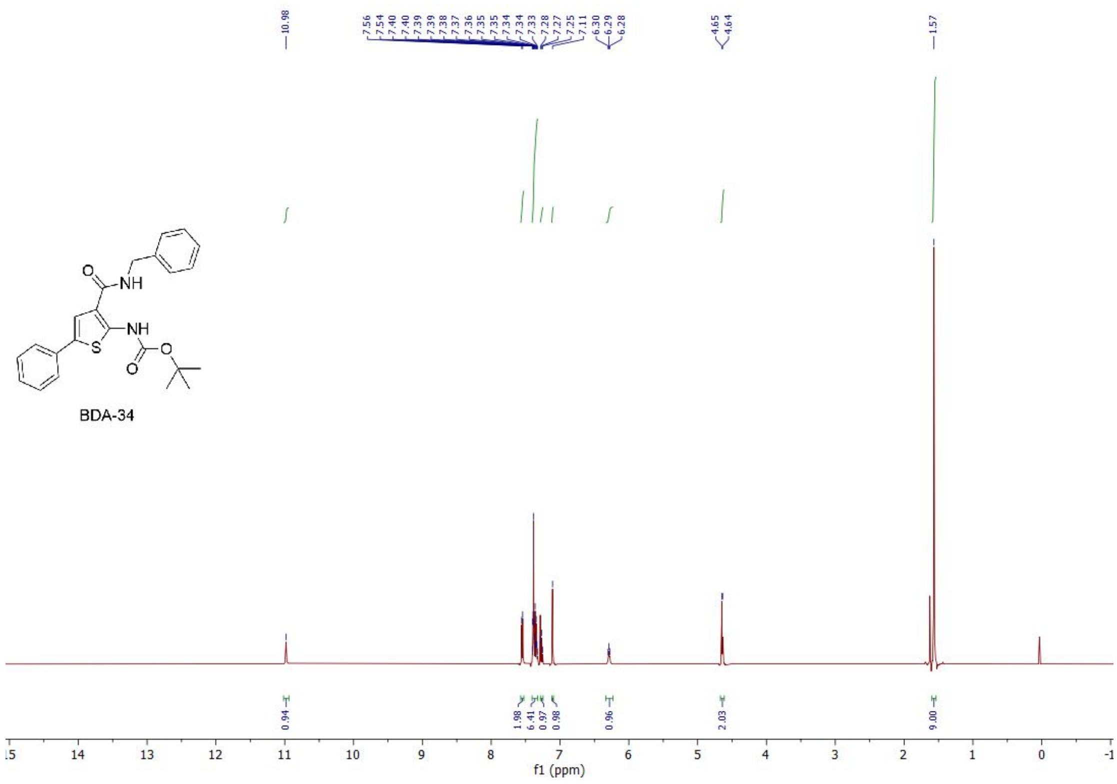

$^{13}C\{^{1}H\}$ NMR spectrum of **BDA-34** (126 MHz, $CDCl_3$)

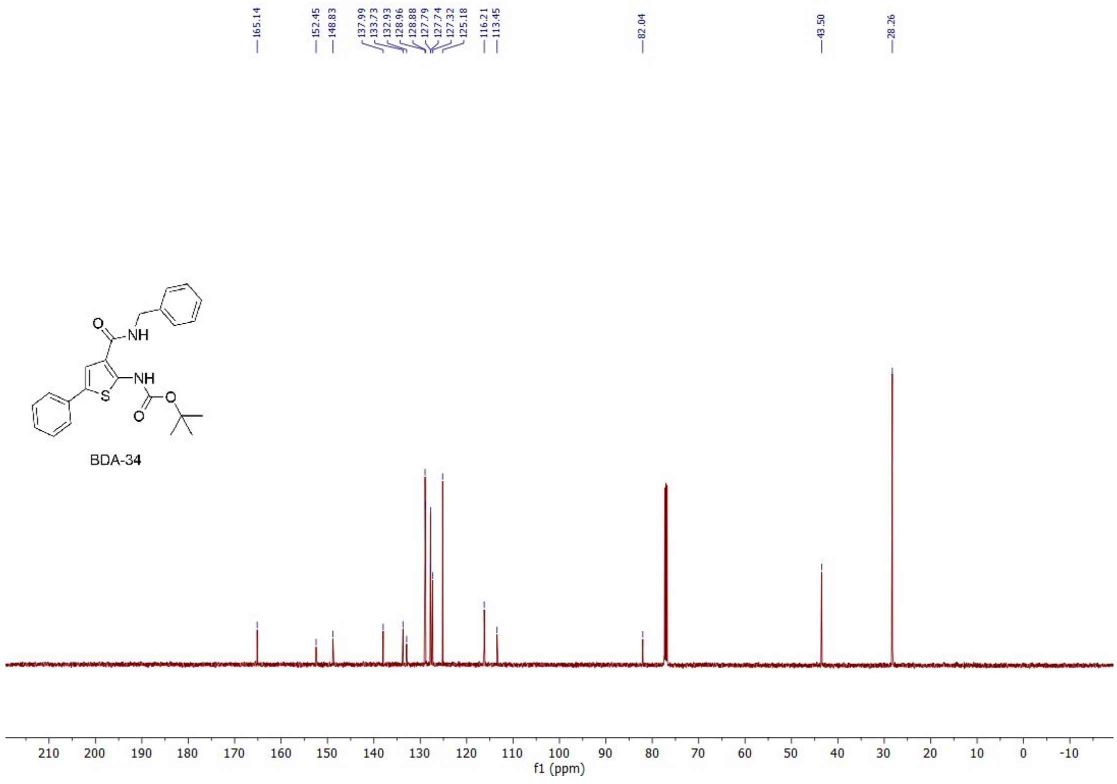

^{1}H NMR spectrum of **BDA-35** (500 MHz, DMSO-d_6)

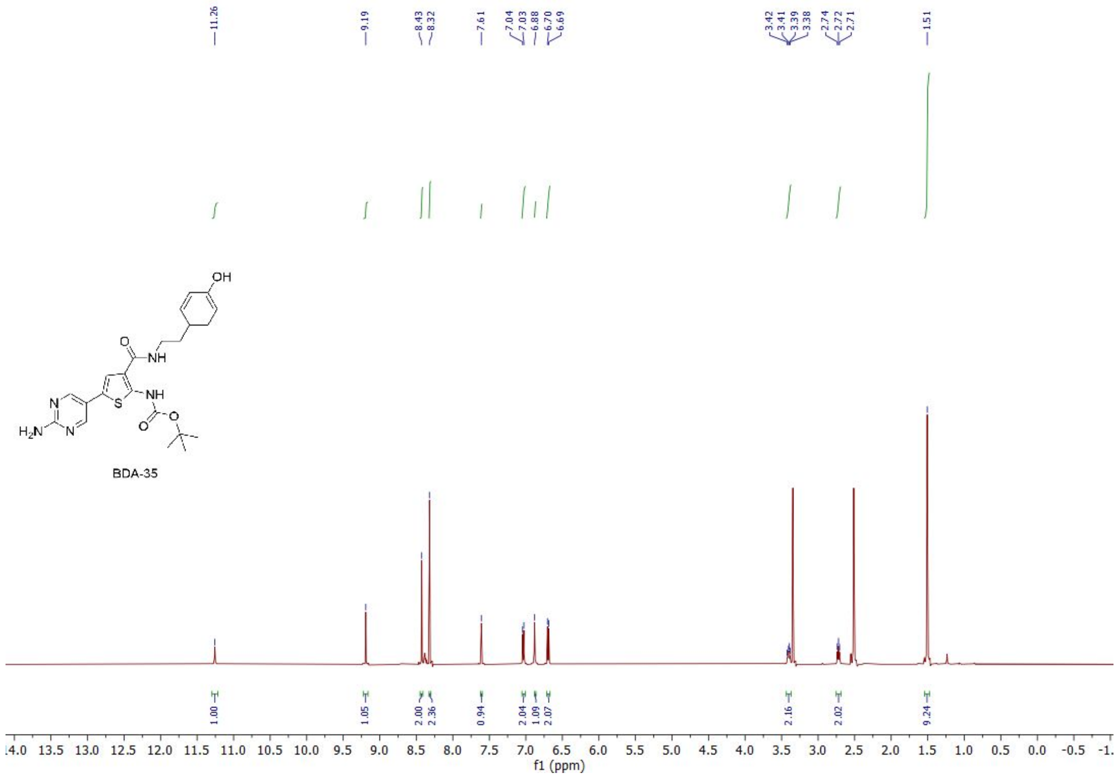

^{13}C{^{1}H} NMR spectrum of **BDA-35** (126 MHz, DMSO-d_6)

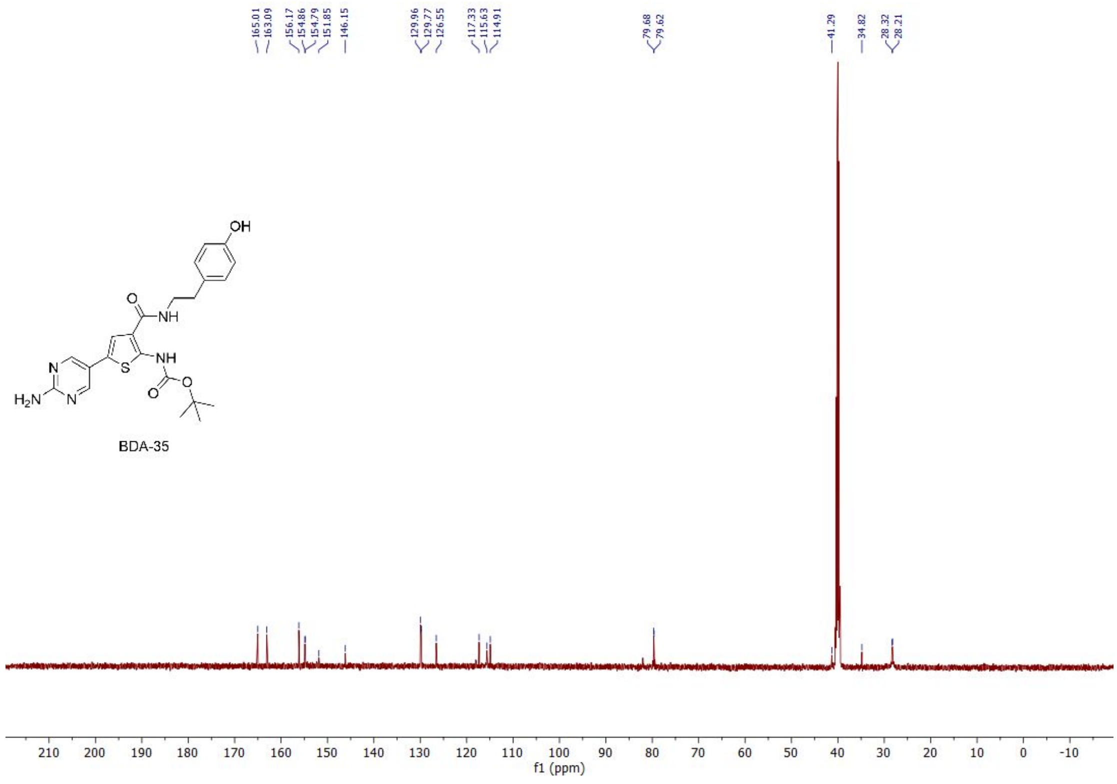

^{1}H NMR spectrum of **BDA-36** (500 MHz, $CDCl_3$)

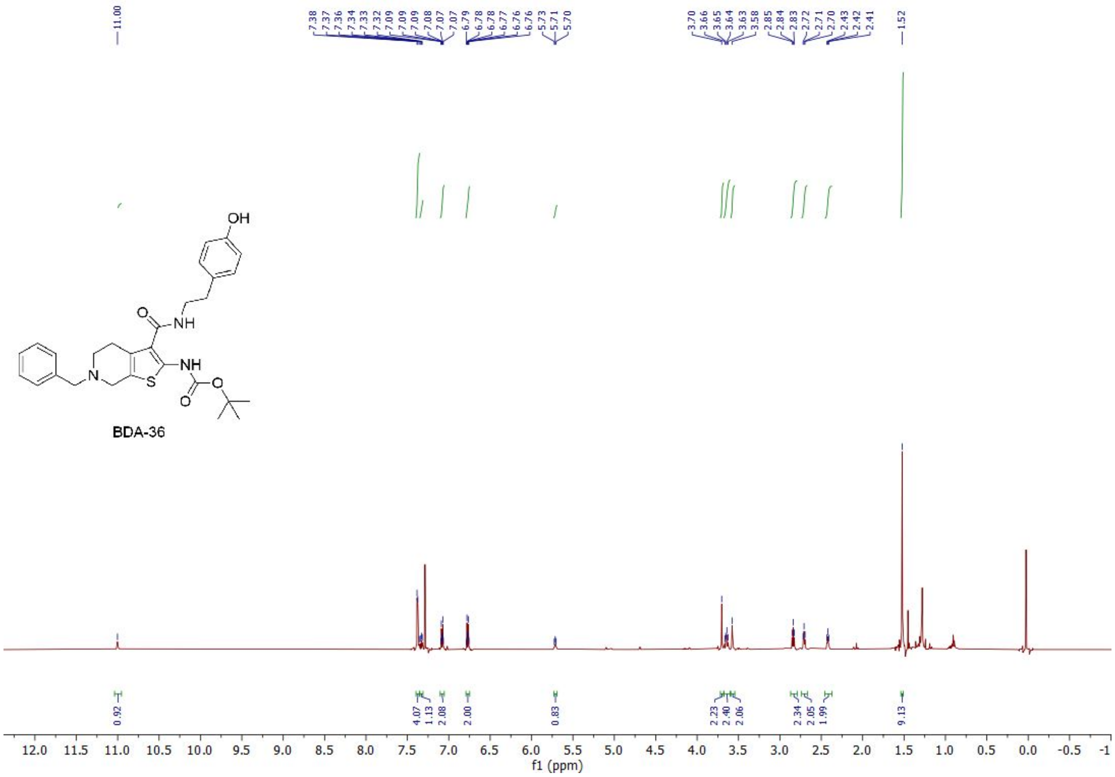

$^{13}C\{^{1}H\}$ NMR spectrum of **BDA-36** (126 MHz, $CDCl_3$)

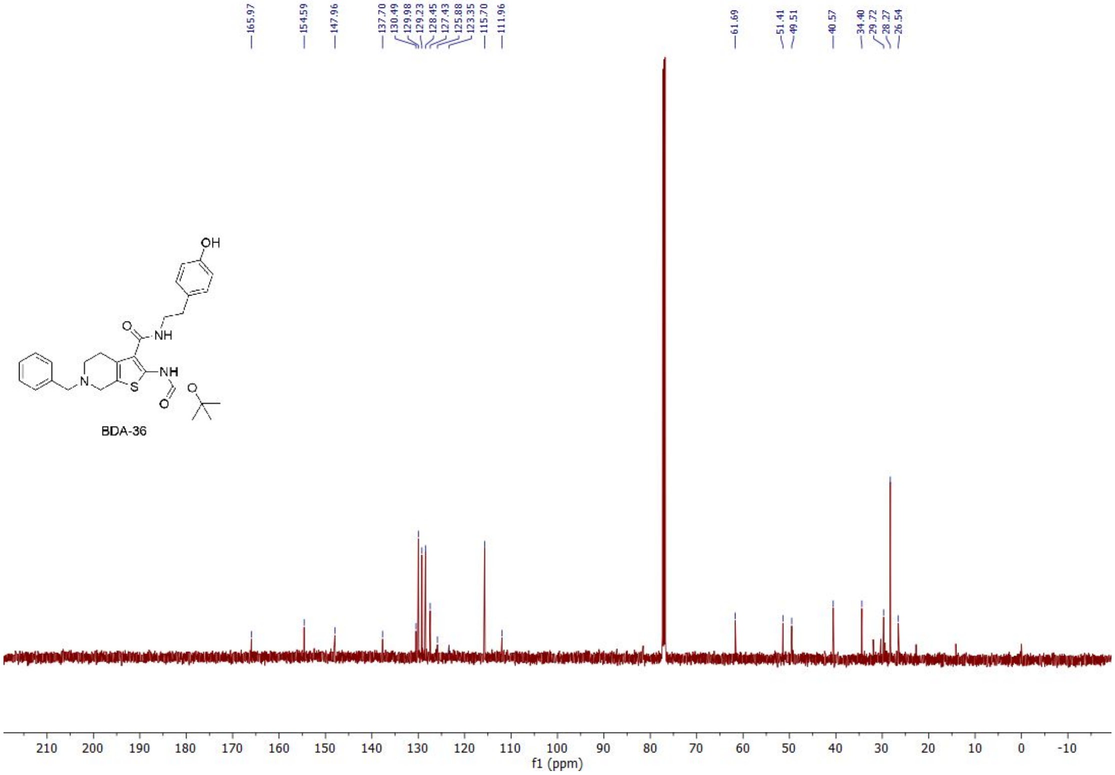

^{1}H NMR spectrum of **BDA-37** (500 MHz, $CDCl_3$)

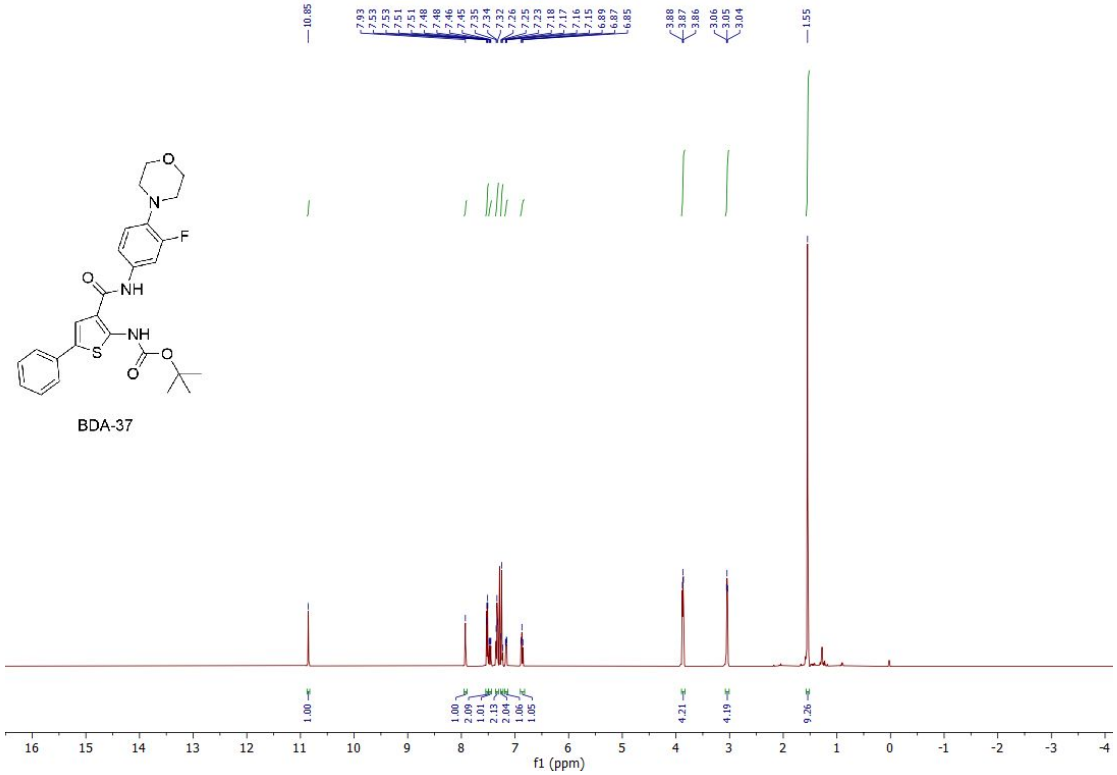

$^{13}C\{^1H\}$ NMR spectrum of **BDA-37** (126 MHz, $CDCl_3$)

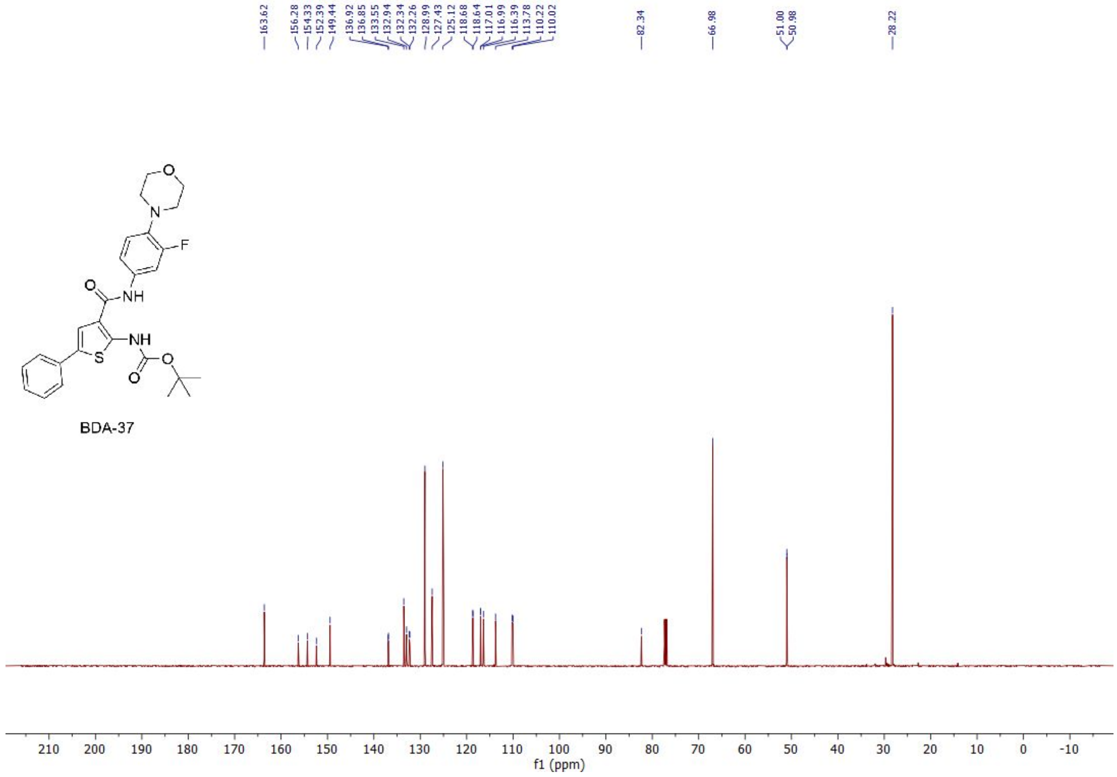

^{1}H NMR spectrum of **BDA-38** (500 MHz, $CDCl_3$)

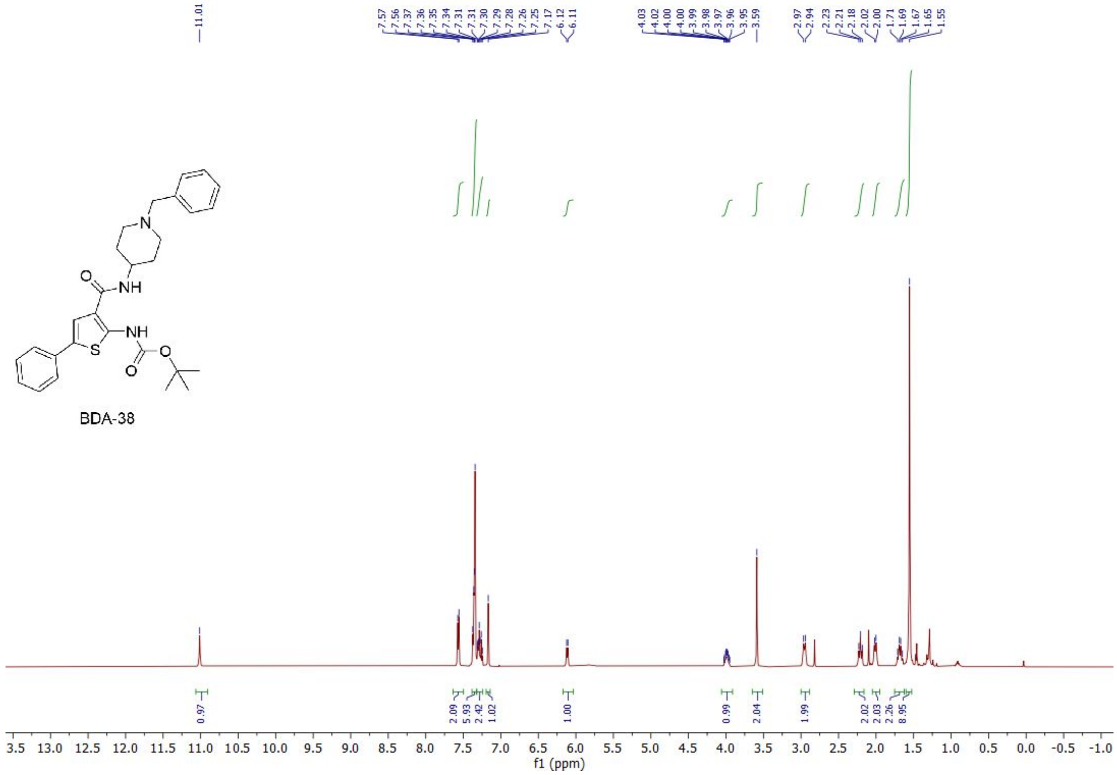

$^{13}C\{^{1}H\}$ NMR spectrum of **BDA-38** (126 MHz, $CDCl_3$)

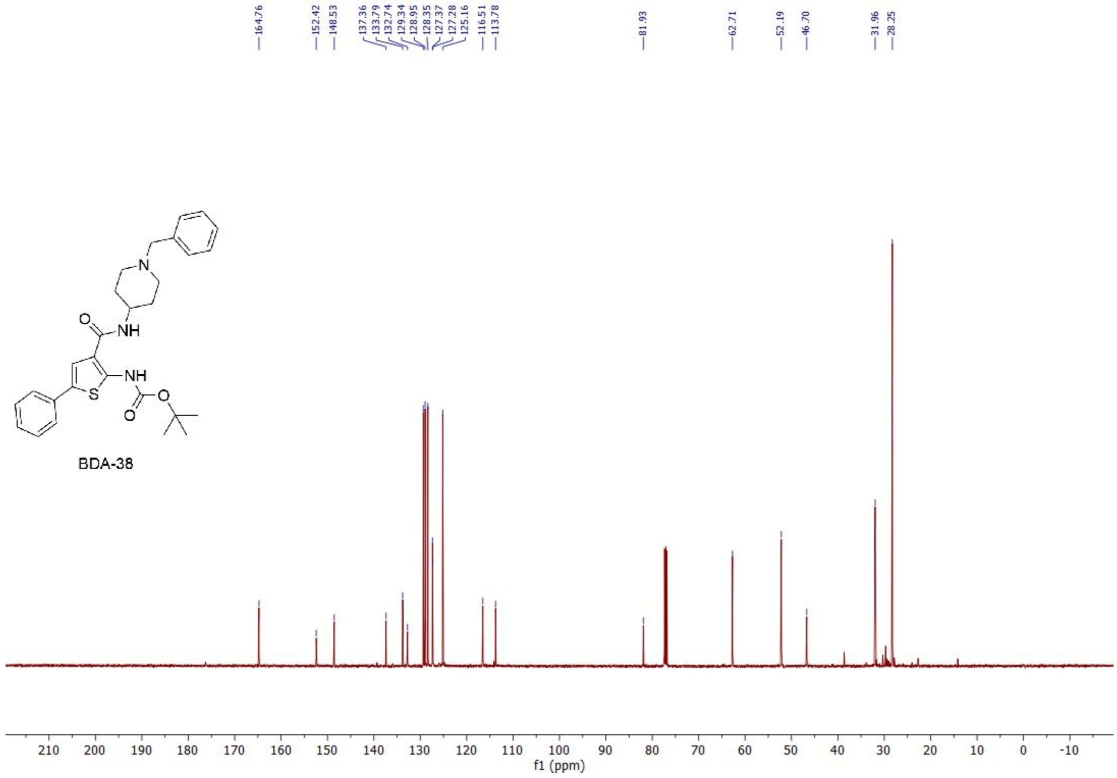

^{1}H NMR spectrum of **BDA-39** (500 MHz, $CDCl_3$)

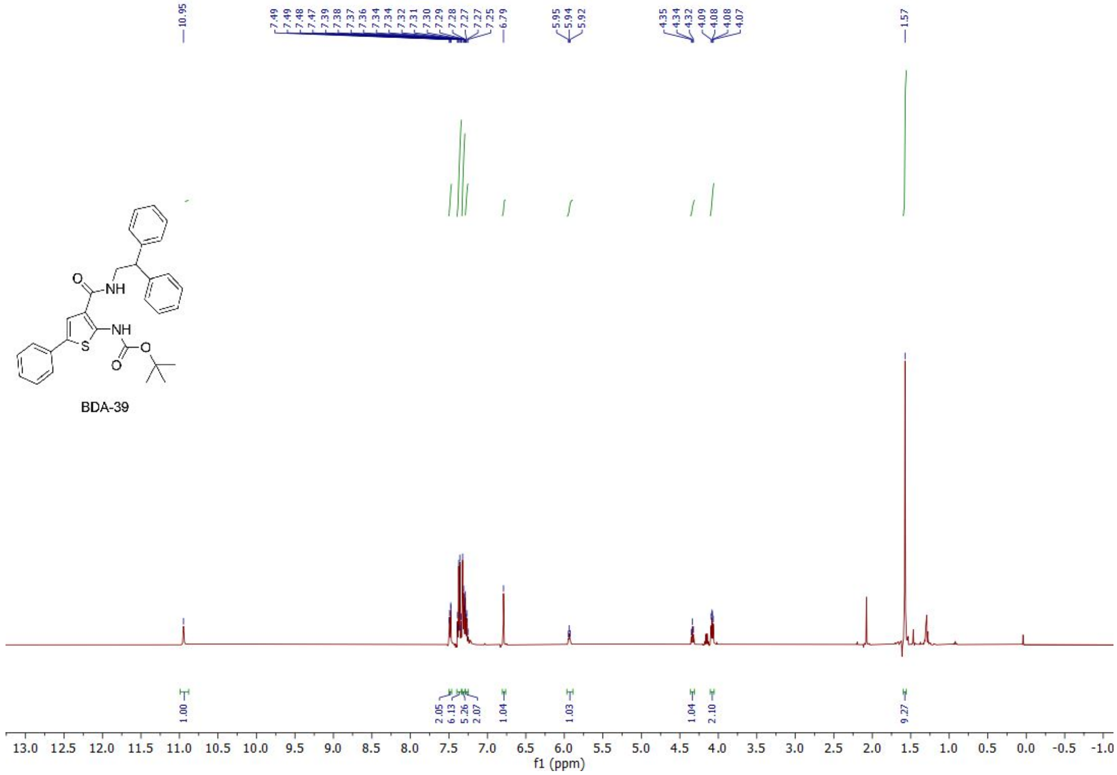

$^{13}C\{^{1}H\}$ NMR spectrum of **BDA-39** (126 MHz, $CDCl_3$)

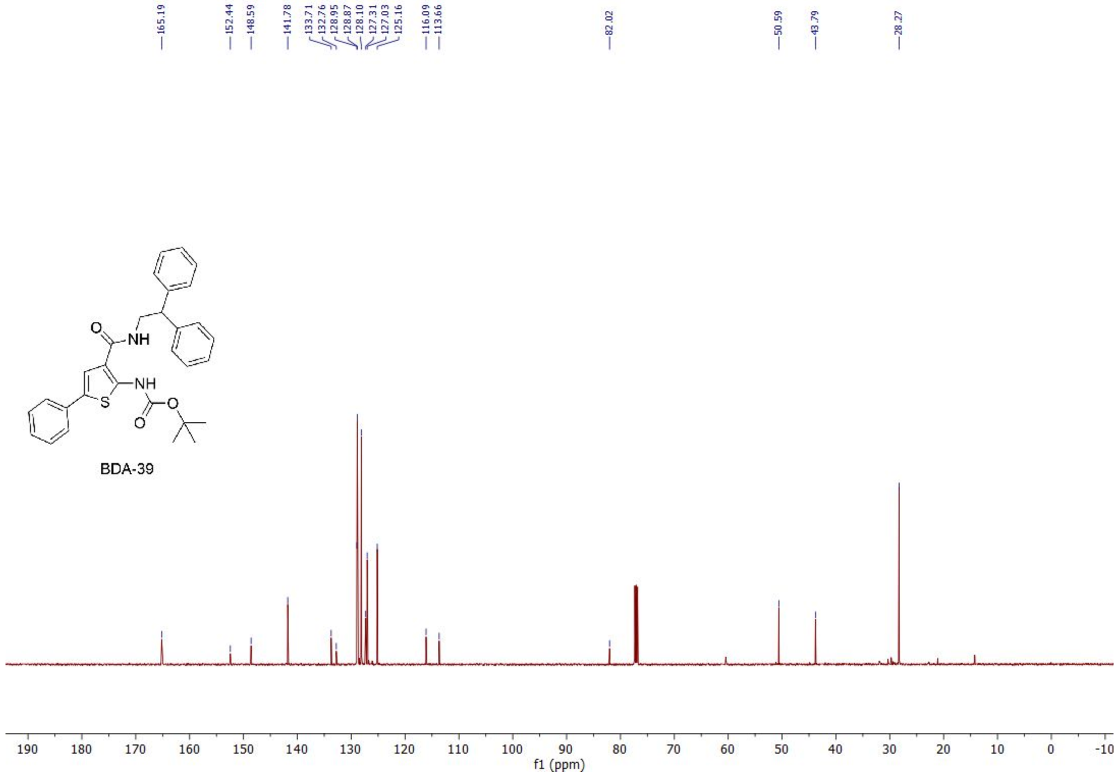

^{1}H NMR spectrum of **BDA-40** (500 MHz, $CDCl_3$)

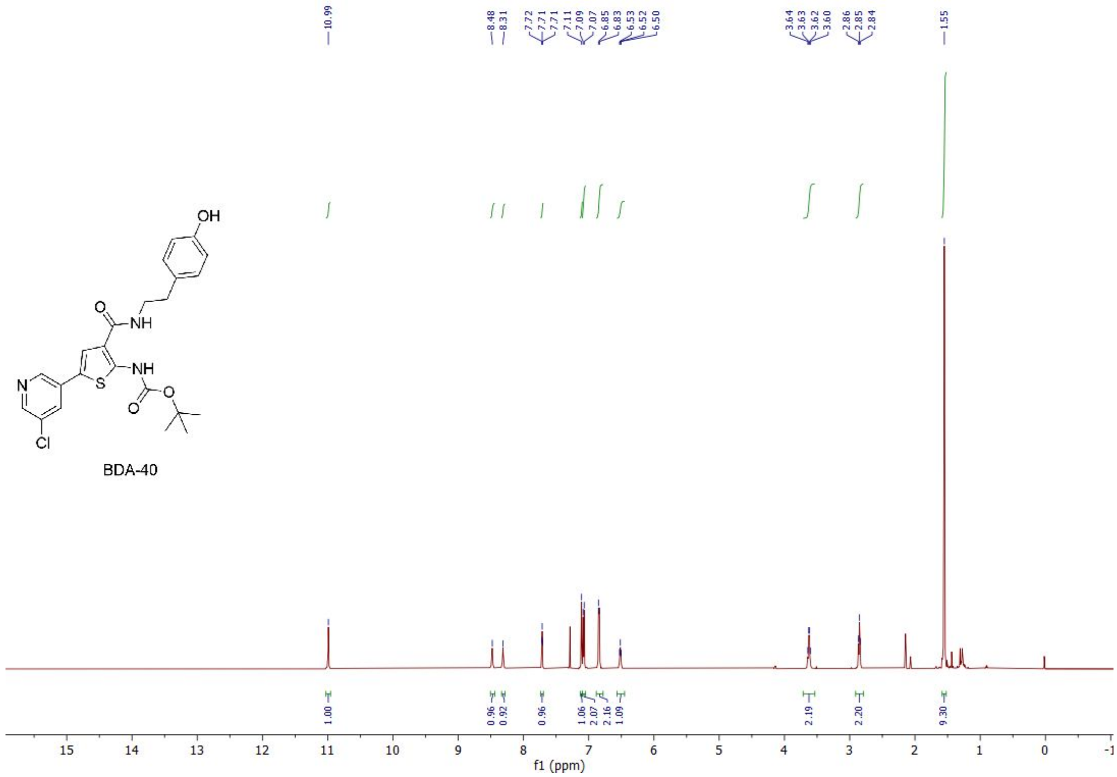

$^{13}C\{^1H\}$ NMR spectrum of **BDA-40** (126 MHz, $CDCl_3$)

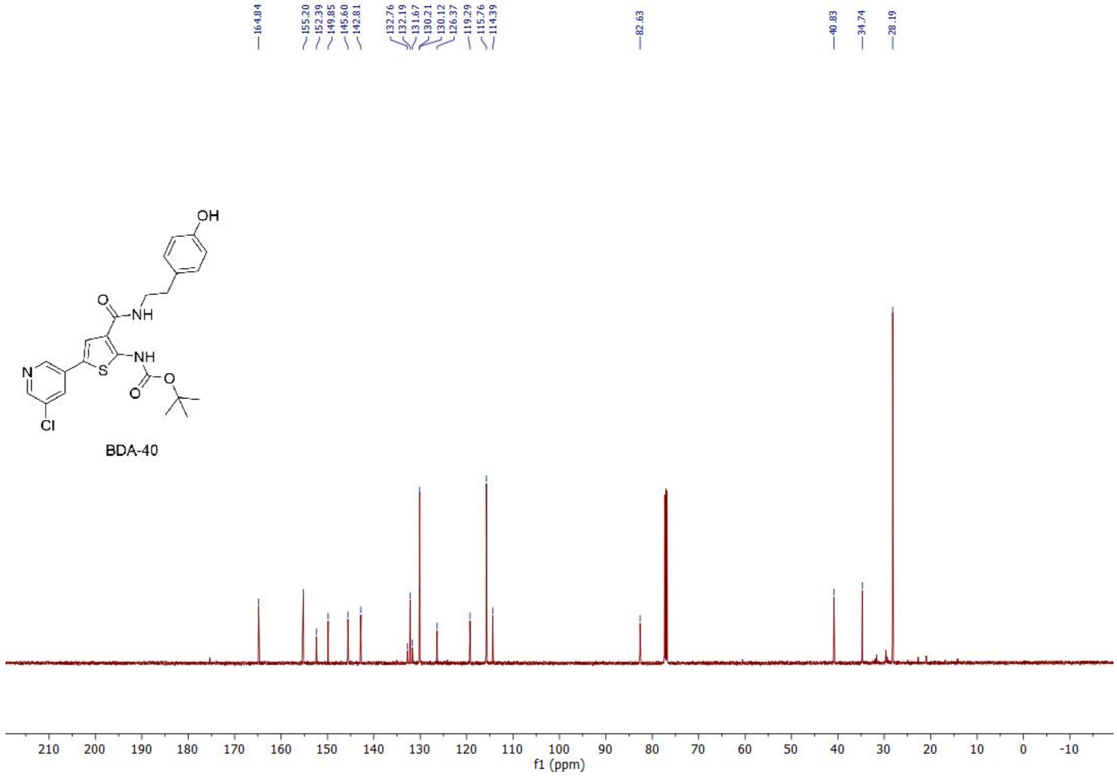

^{1}H NMR spectrum of **BDA-41** (500 MHz, DMSO-d_6)

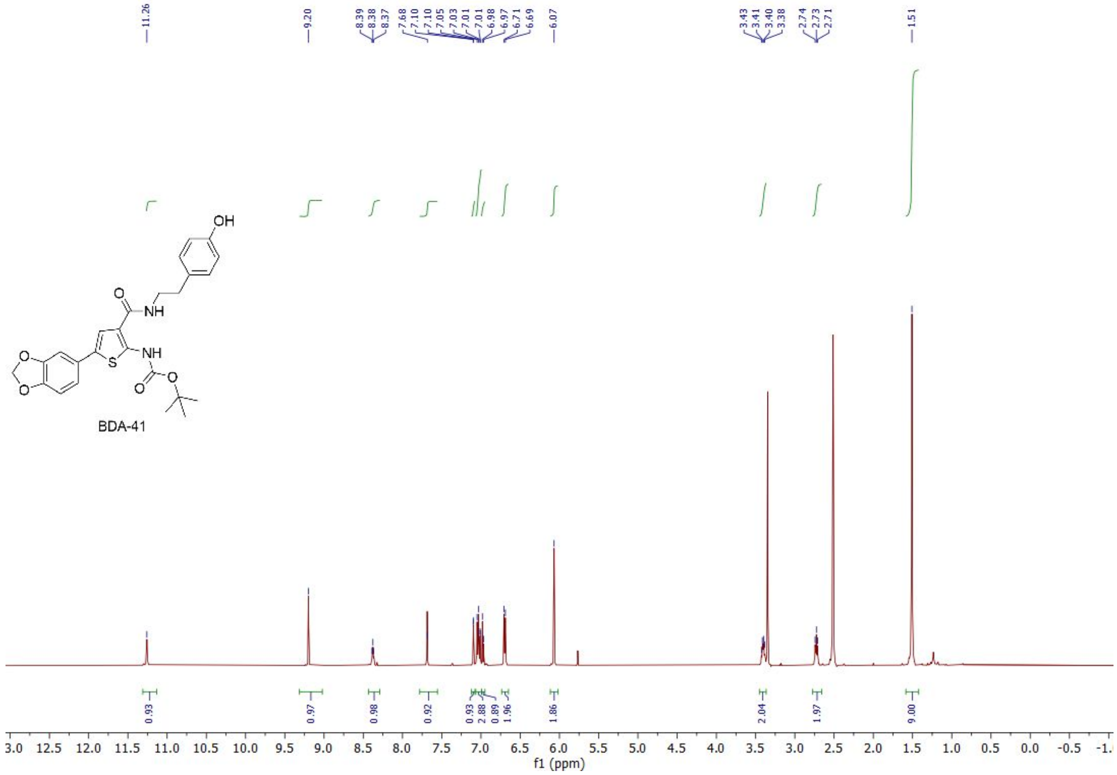

$^{13}C\{^{1}H\}$ NMR spectrum of **BDA-41** (126 MHz, DMSO-d_6)

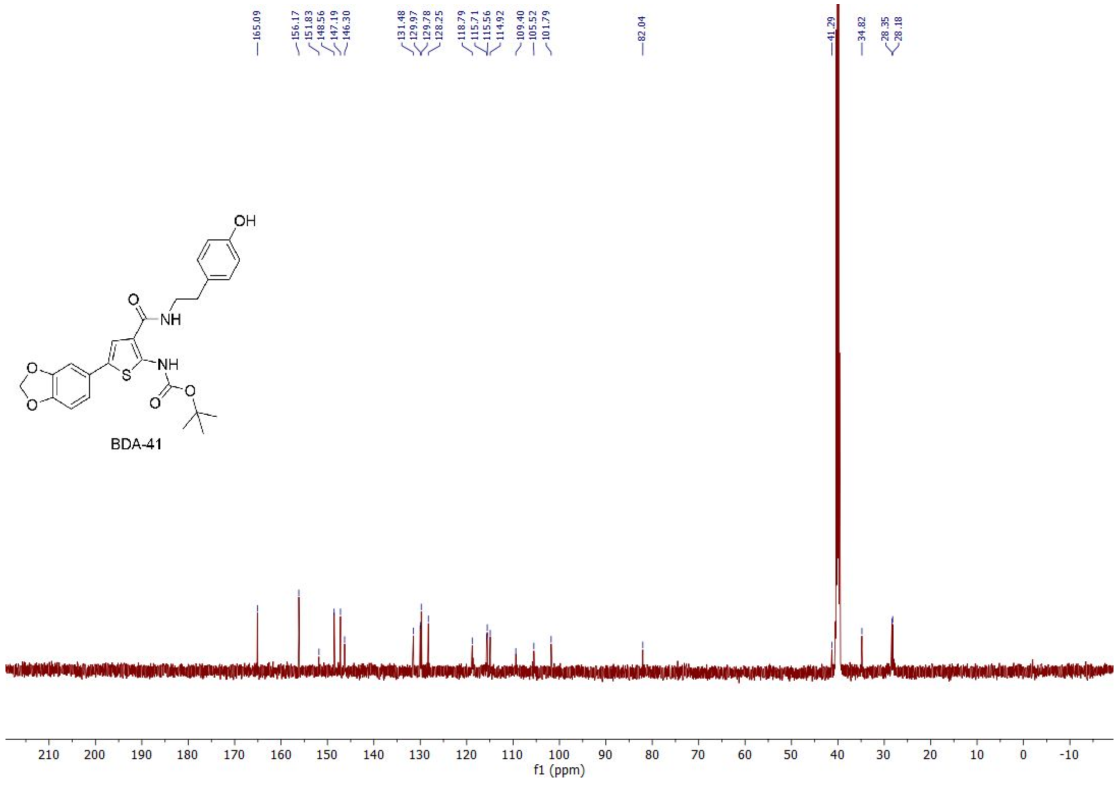

^{1}H NMR spectrum of **BDA-42** (500 MHz, $CDCl_3$)

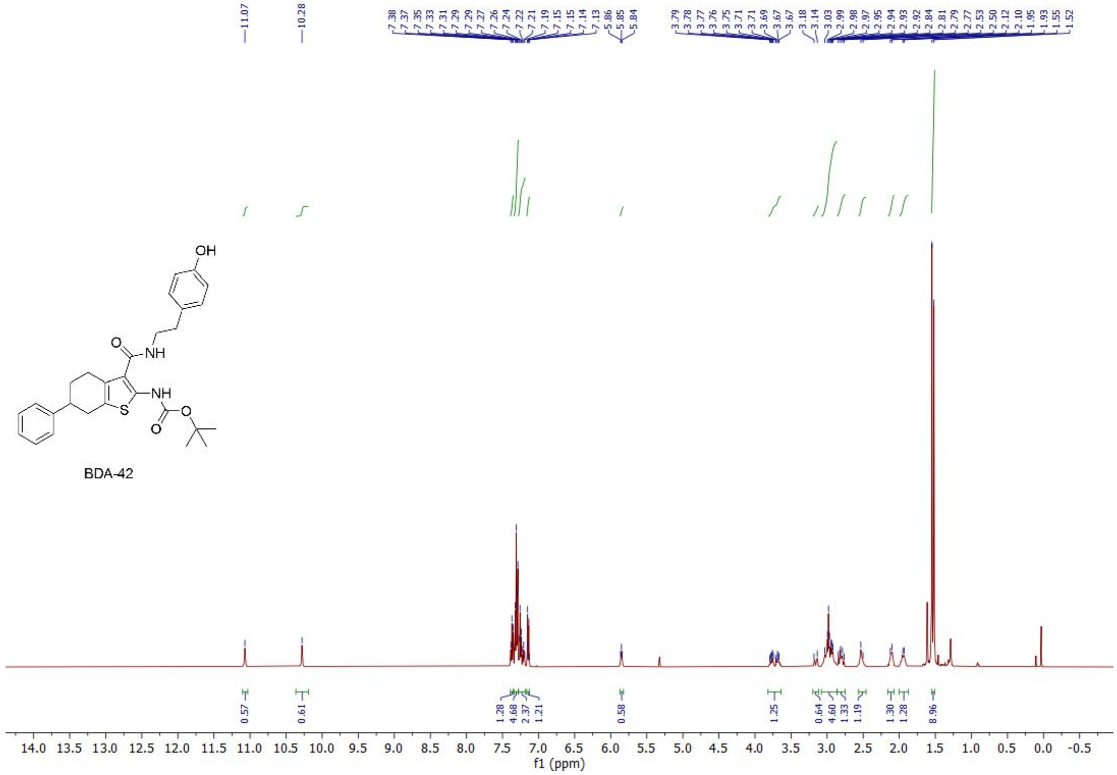

$^{13}C\{^{1}H\}$ NMR spectrum of **BDA-42** (126 MHz, $CDCl_3$)

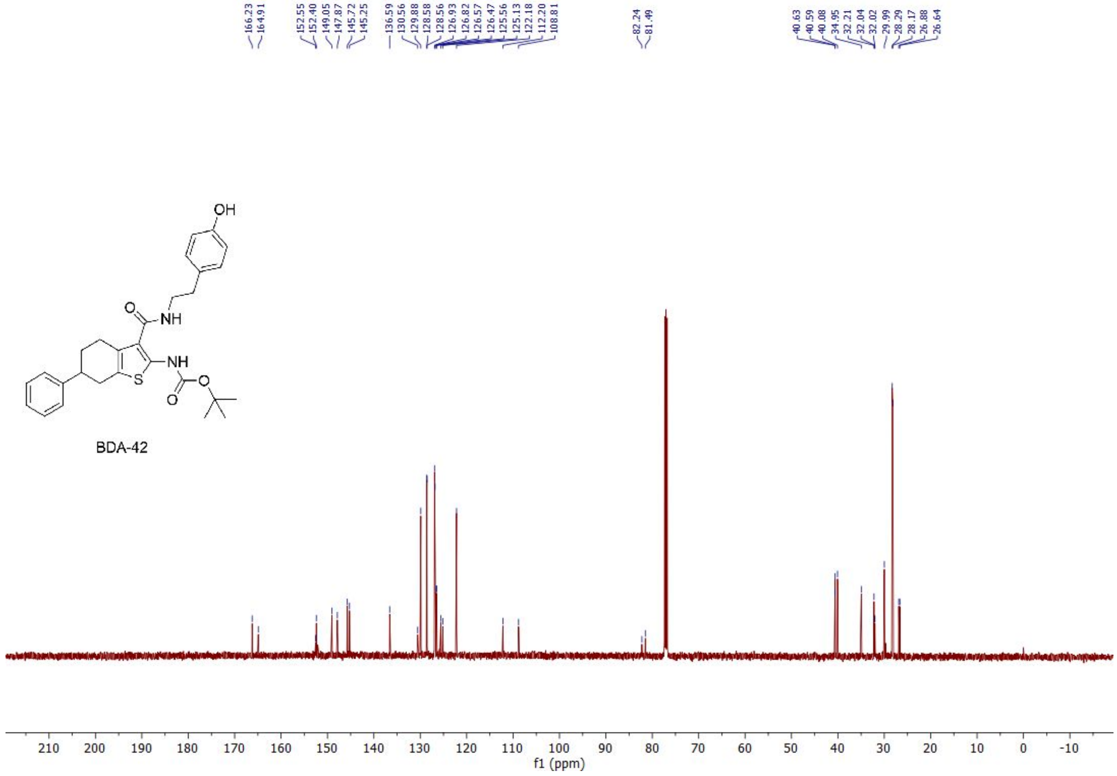

^{1}H NMR spectrum of **BDA-43** (500 MHz, $CDCl_3$)

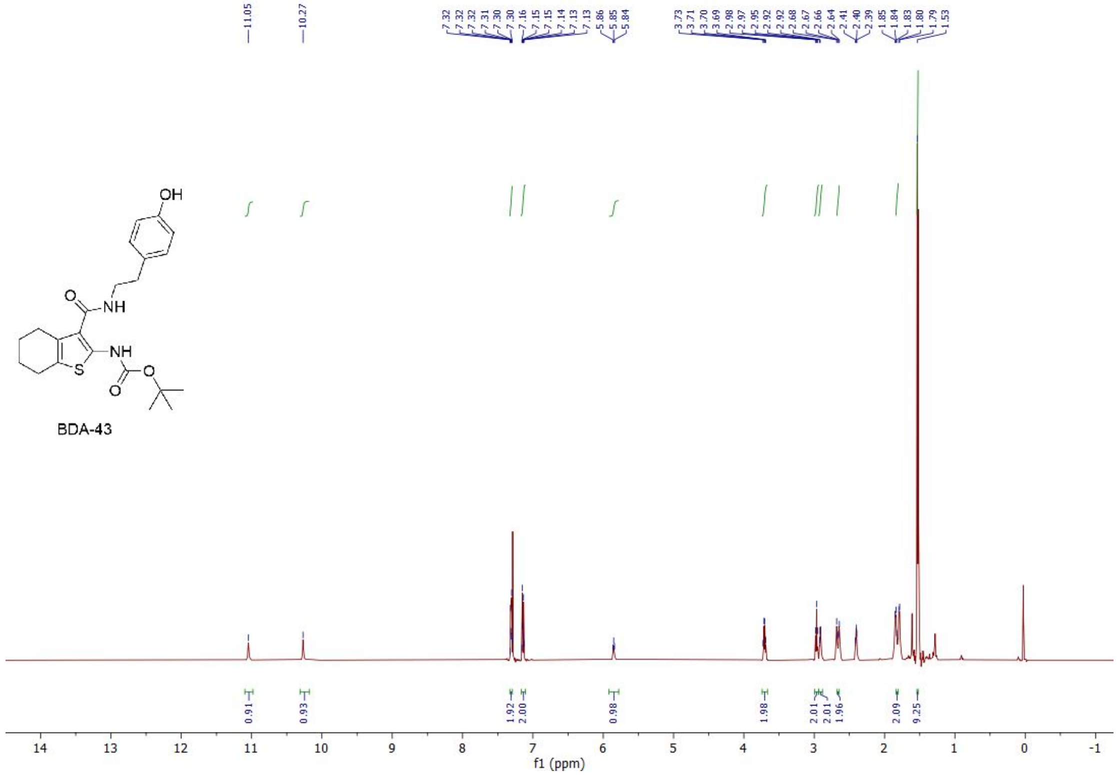

$^{13}C\{^{1}H\}$ NMR spectrum of **BDA-43** (126 MHz, $CDCl_3$)

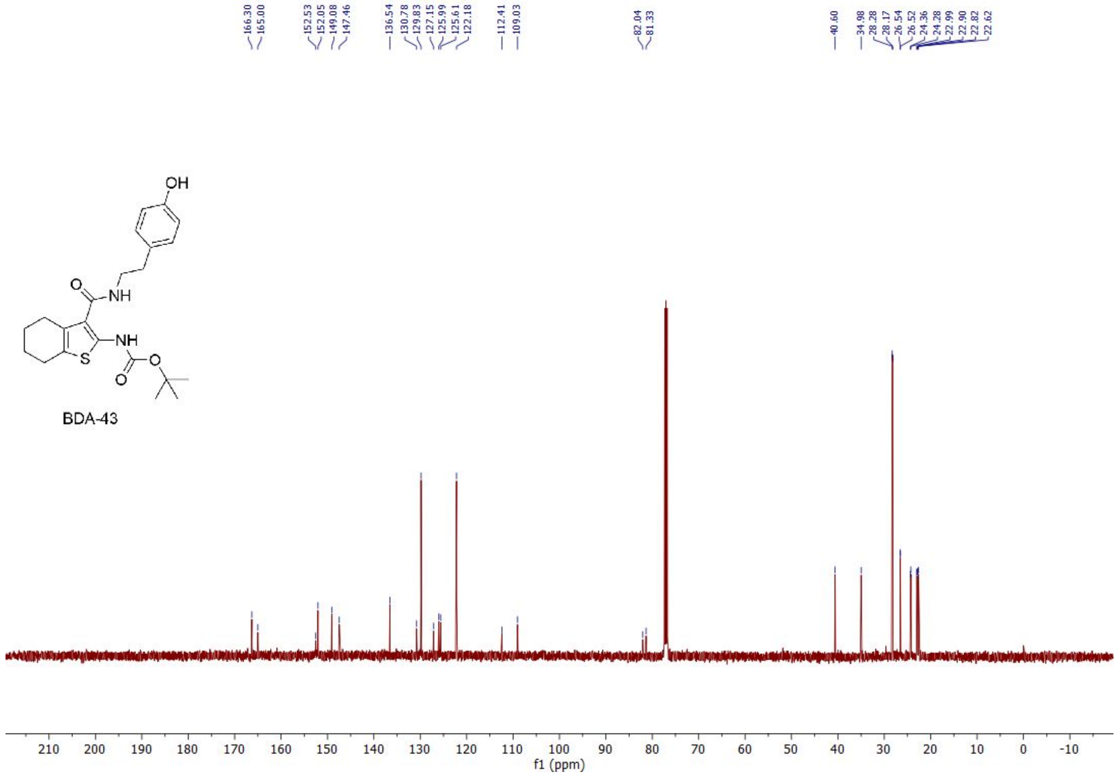

^{1}H NMR spectrum of **BDA-44** (500 MHz, DMSO- d_6)

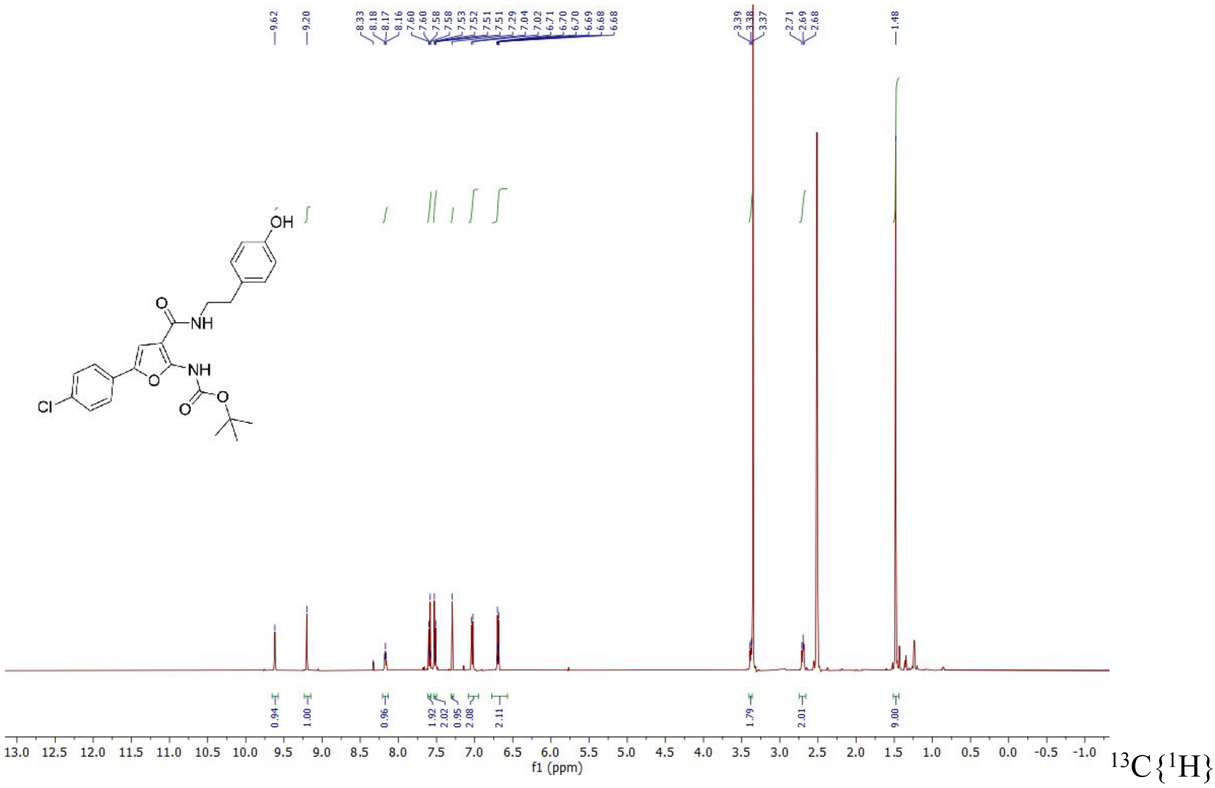

^{13}C{^{1}H} NMR spectrum of **BDA-44** (126 MHz, DMSO- d_6)

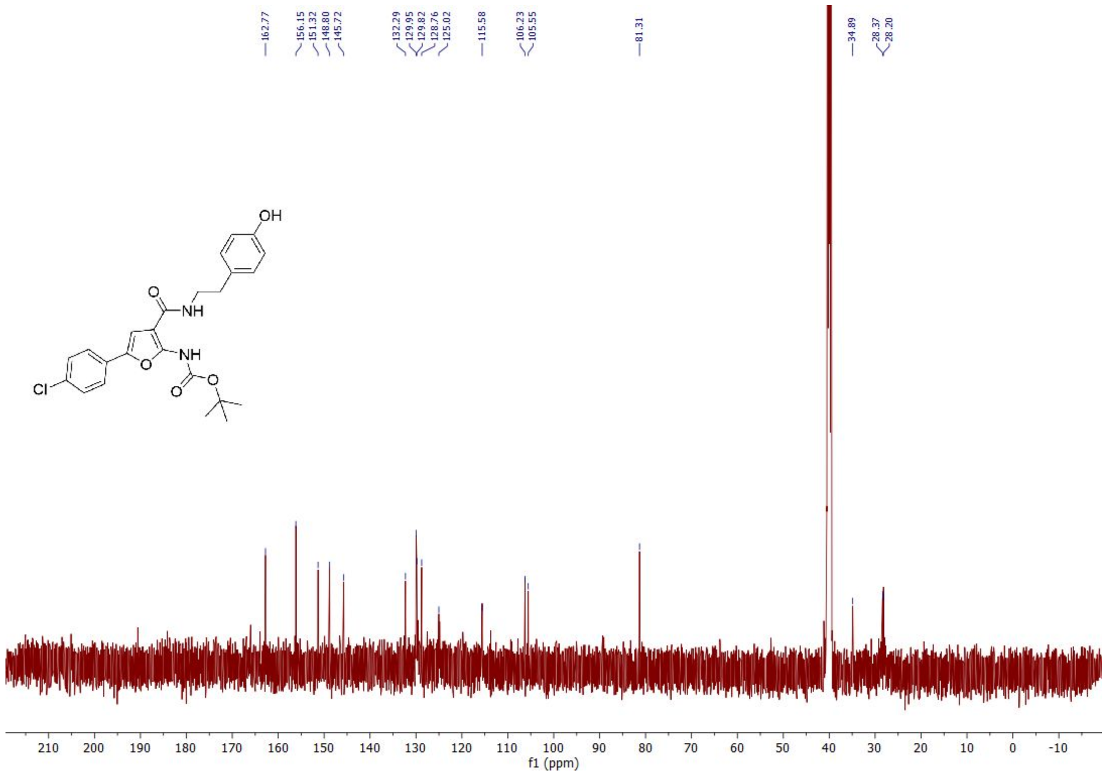

^{1}H NMR spectrum of **BDA-45** (500 MHz, DMSO-d_6)

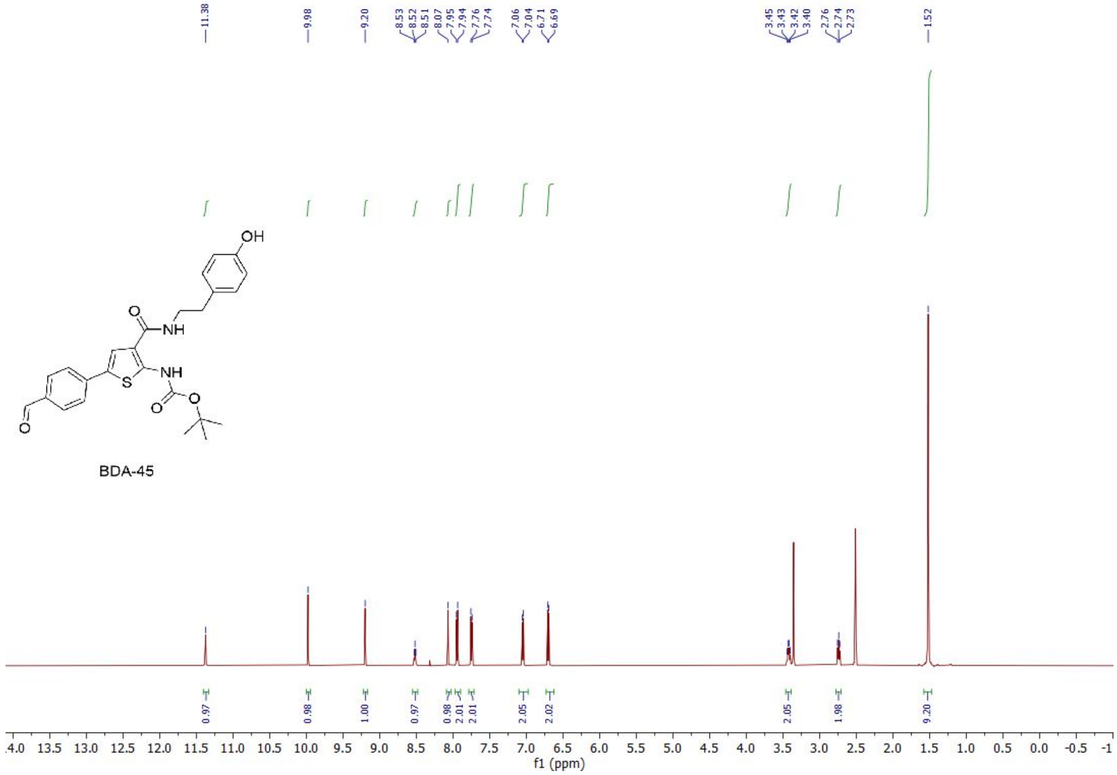

$^{13}C\{^{1}H\}$ NMR spectrum of **BDA-45** (126 MHz, DMSO-d_6)

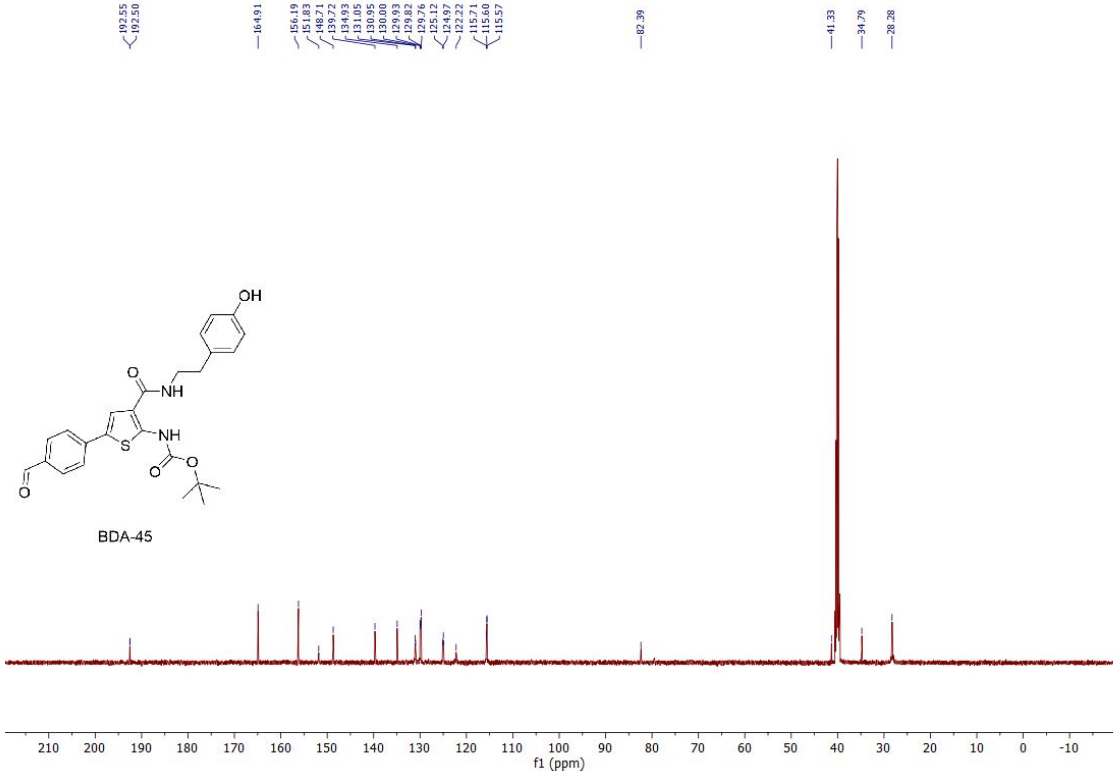

^{1}H NMR spectrum of **BDA-46** (500 MHz, $CDCl_3$)

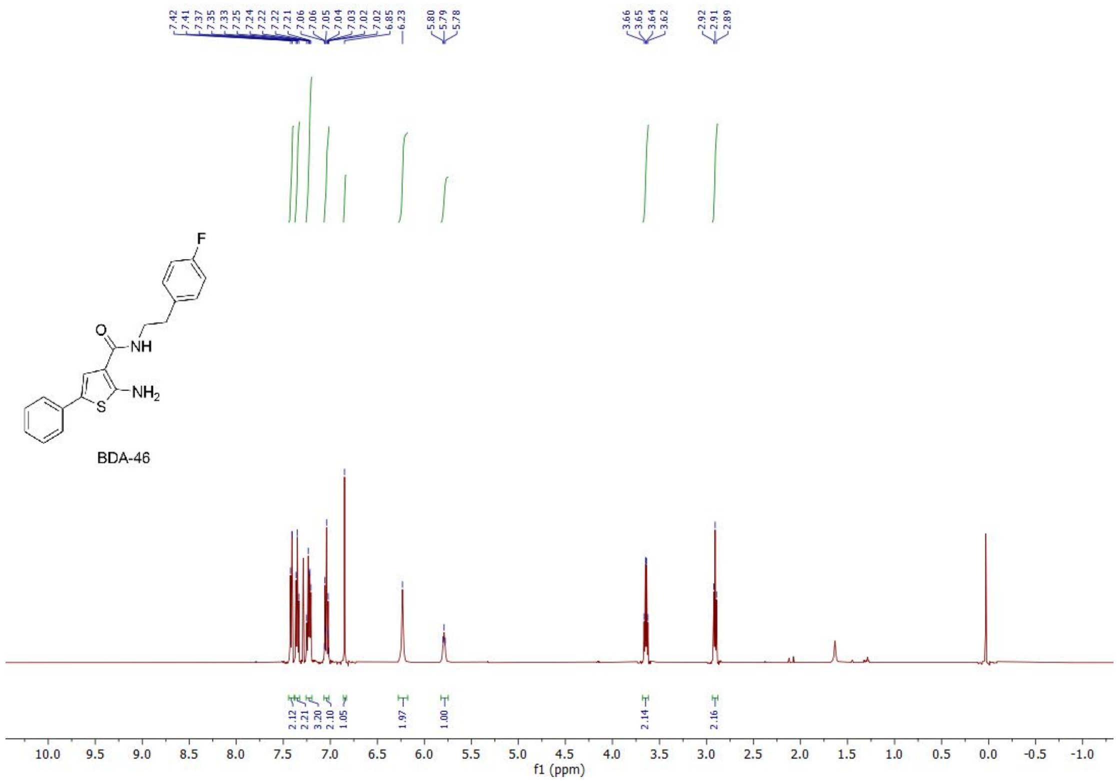

$^{13}C\{^{1}H\}$ NMR spectrum of **BDA-46** (126 MHz, $CDCl_3$)

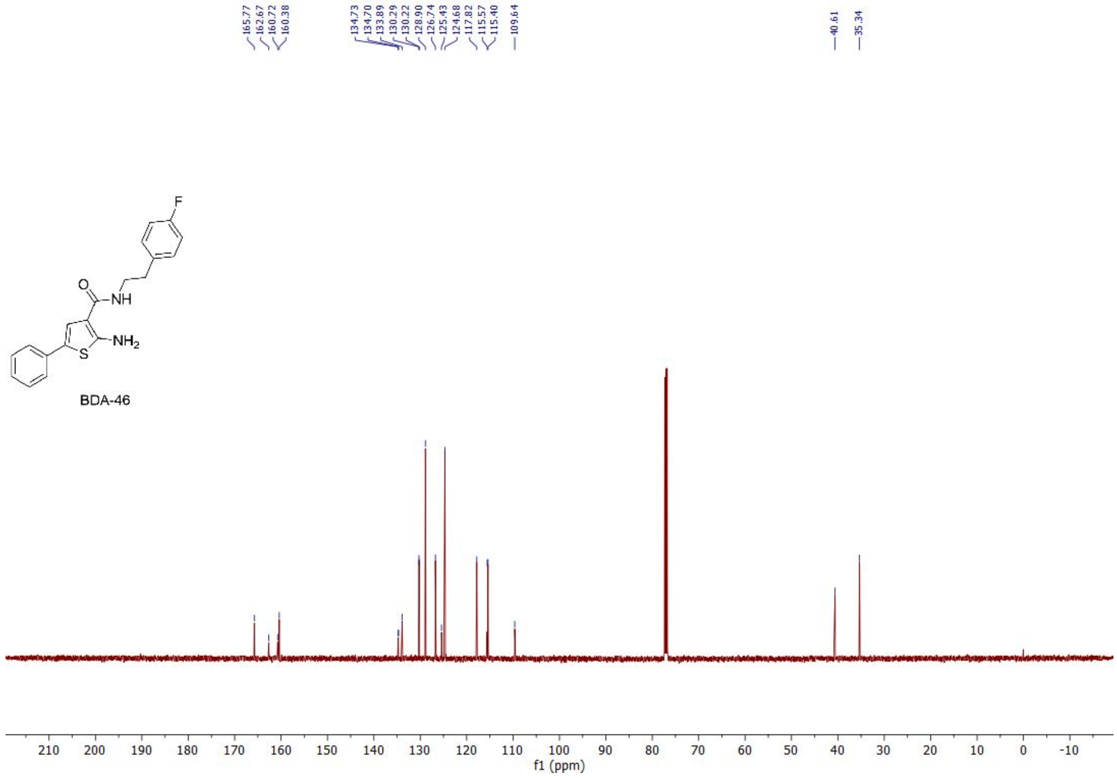

^{1}H NMR spectrum of **BDA-47** (500 MHz, DMSO-d_6)

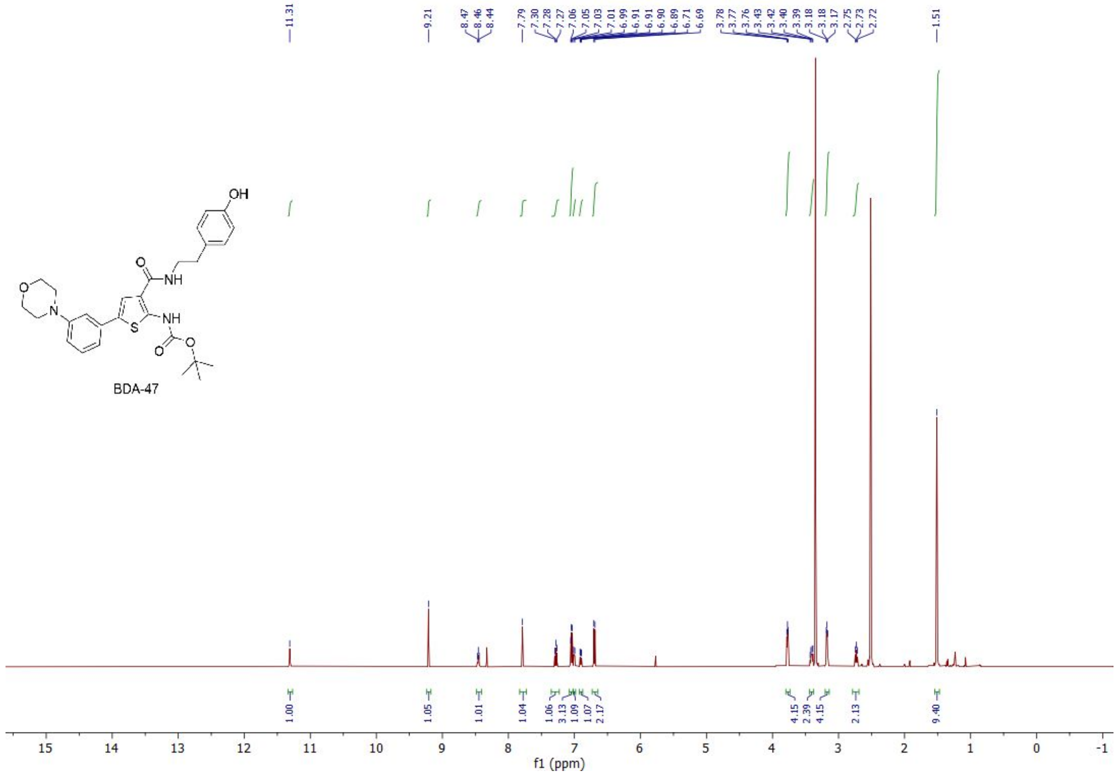

$^{13}C\{^{1}H\}$ NMR spectrum of **BDA-47** (126 MHz, DMSO-d_6)

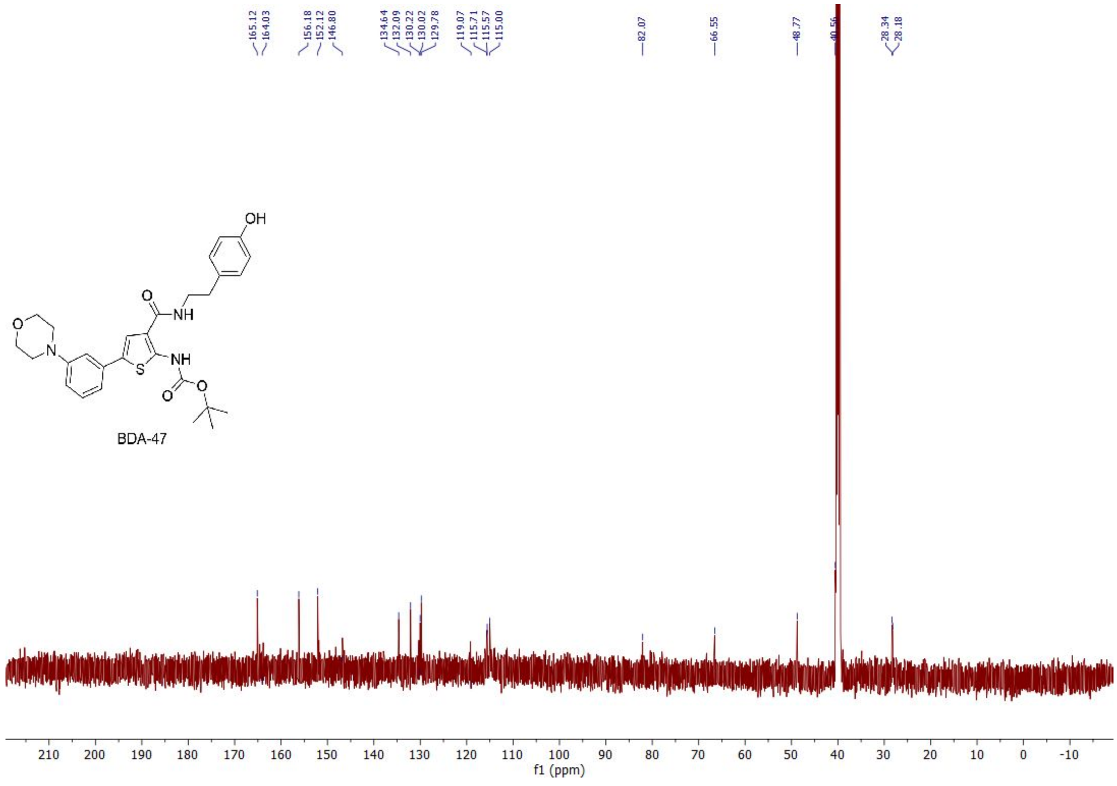

^{1}H NMR spectrum of **BDA-48** (500 MHz, $CDCl_3$)

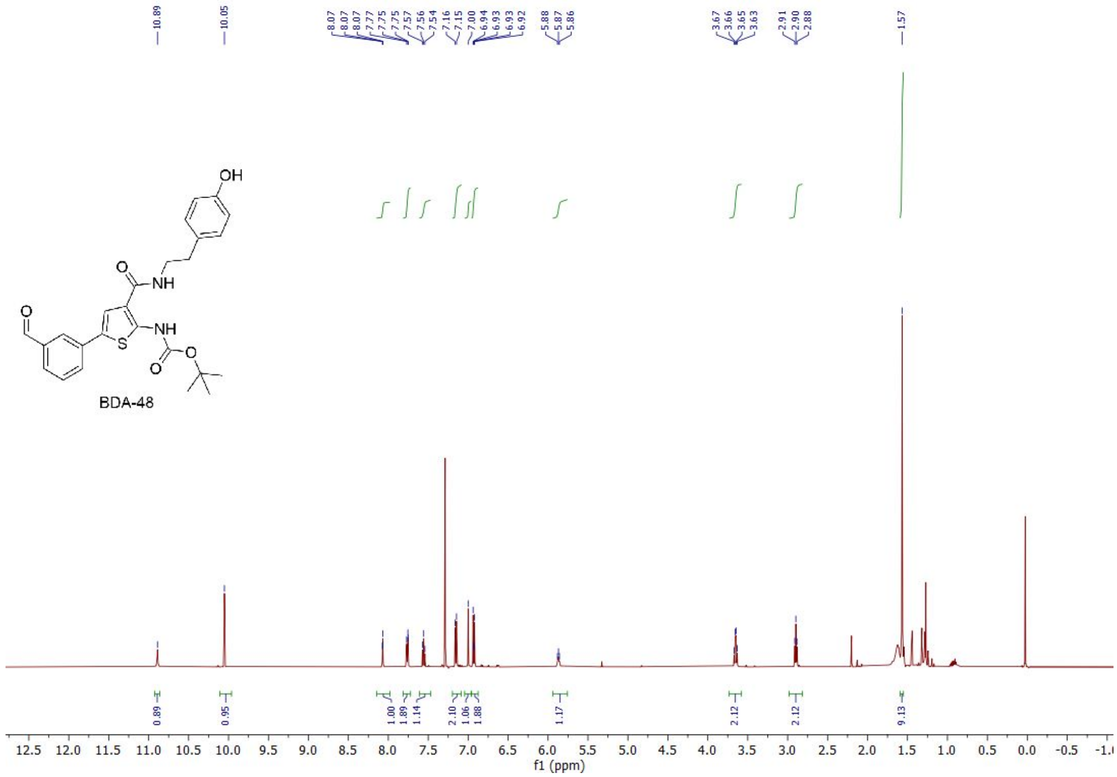

^{13}C{^{1}H} NMR spectrum of **BDA-48** (126 MHz, $CDCl_3$)

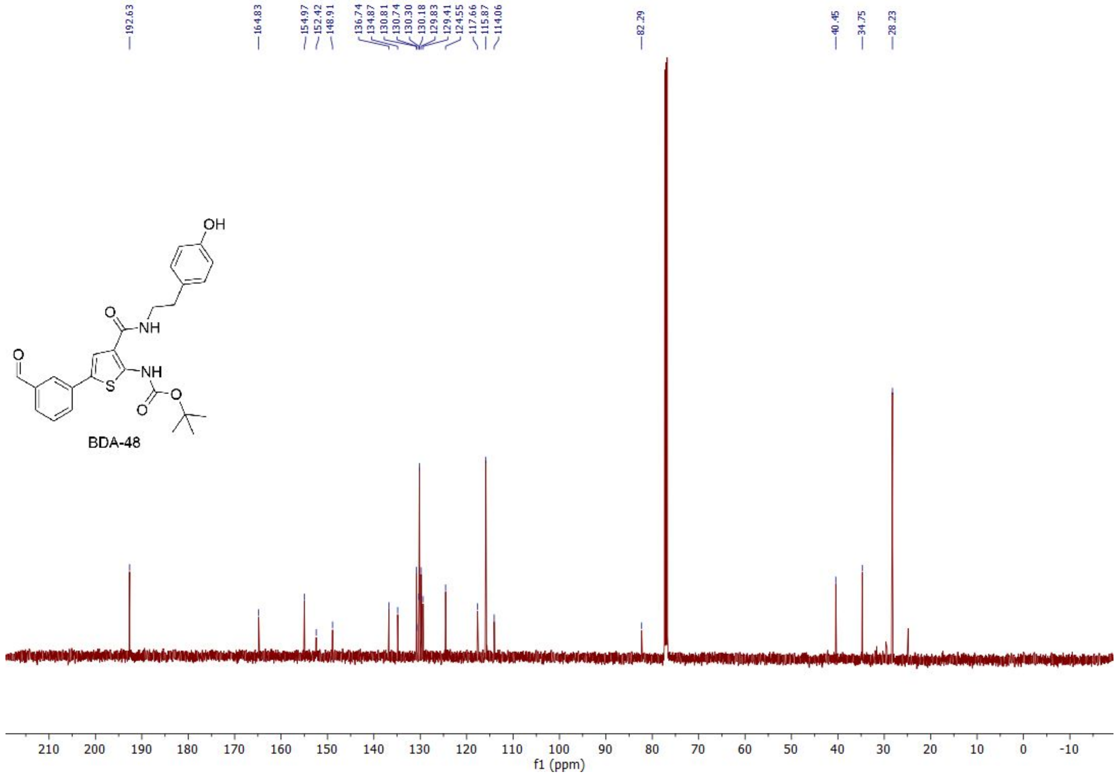

^{1}H NMR spectrum of **BDA-49** (500 MHz, $CDCl_3$)

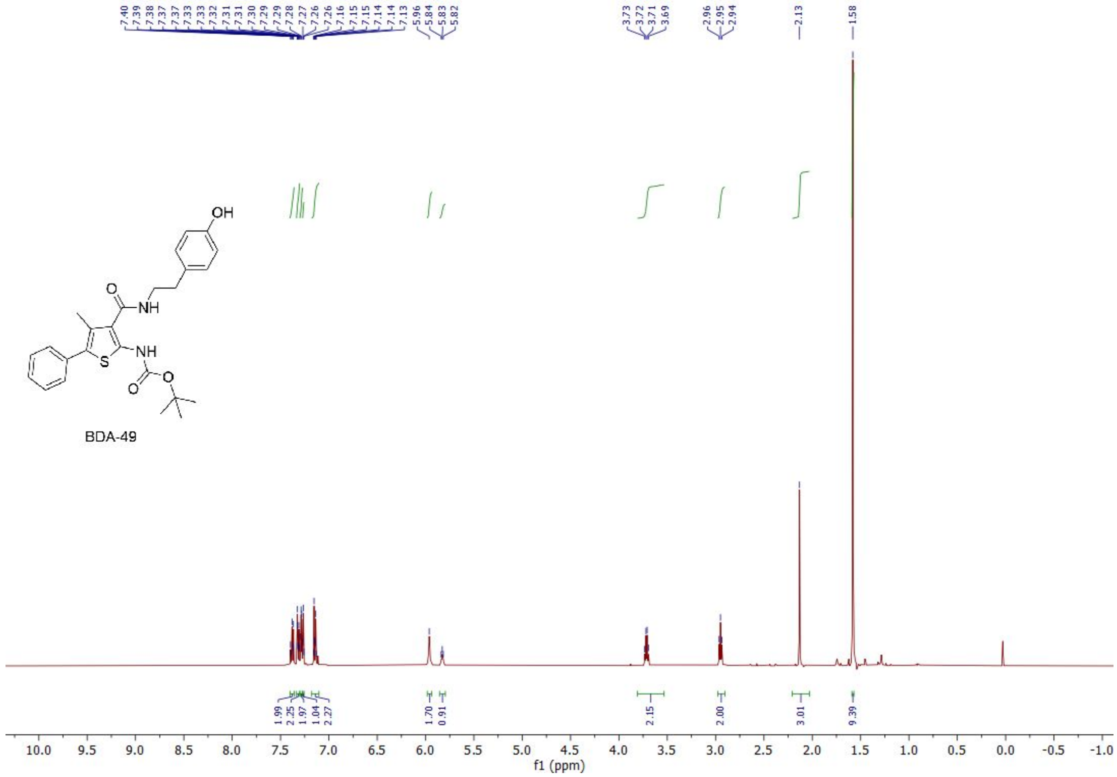

$^{13}C\{^{1}H\}$ NMR spectrum of **BDA-49** (126 MHz, $CDCl_3$)

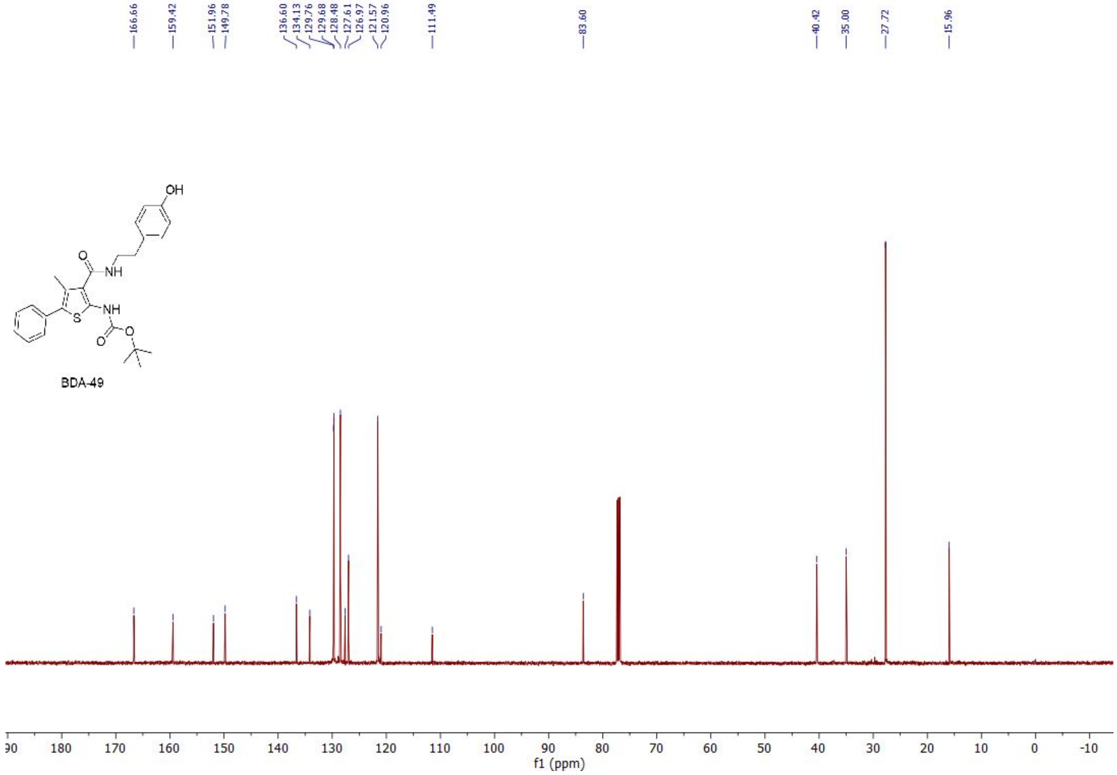

^{1}H NMR spectrum of **BDA-50** (500 MHz, DMSO-d_6)

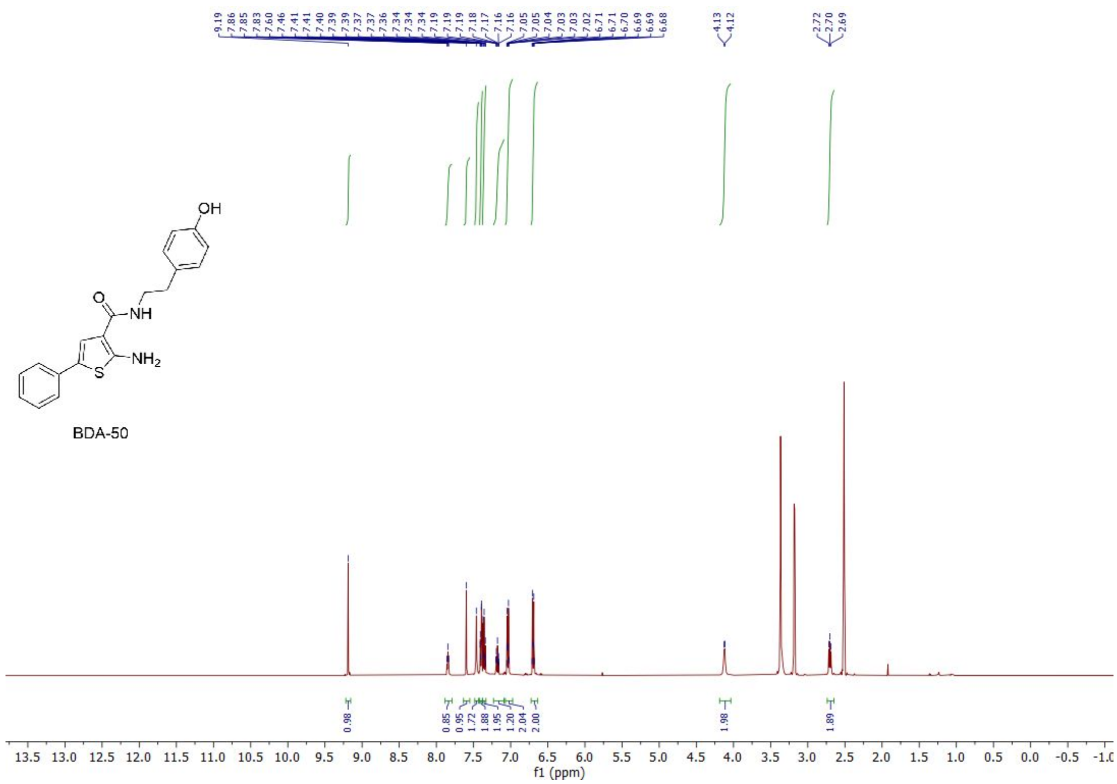

$^{13}C\{^{1}H\}$ NMR spectrum of **BDA-50** (126 MHz, DMSO-d_6)

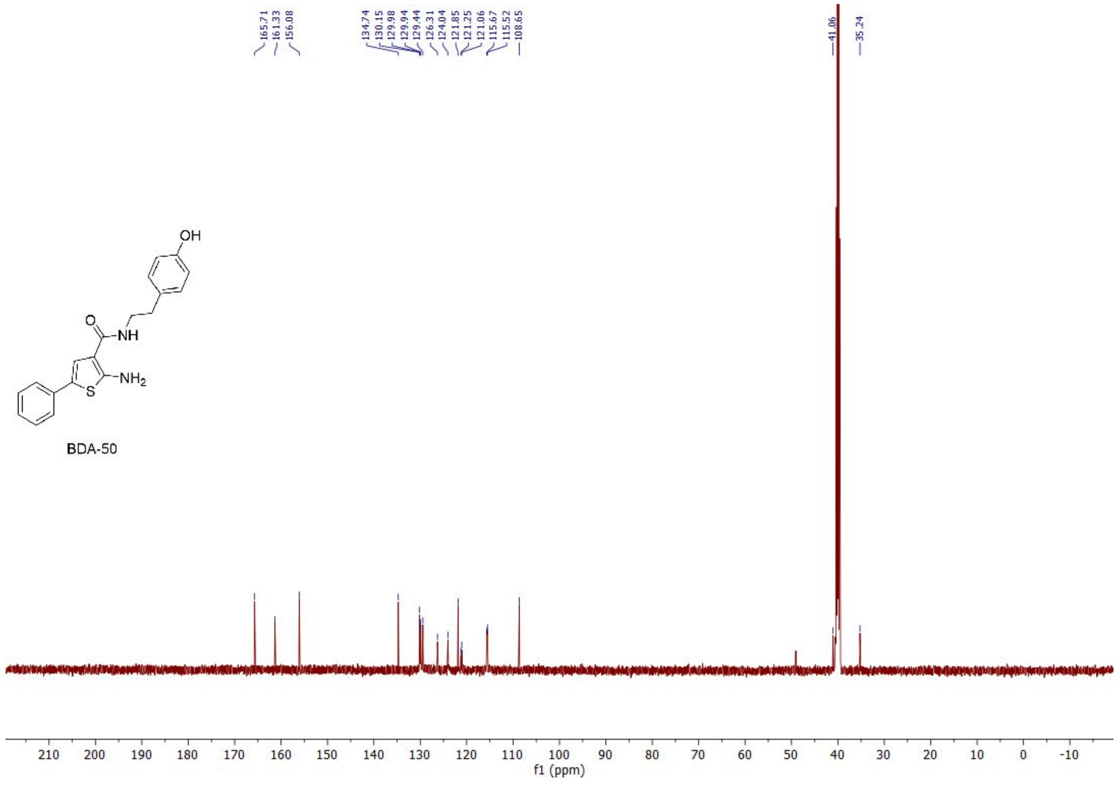

^{1}H NMR spectrum of **BDA-51** (500 MHz, DMSO-d_6)

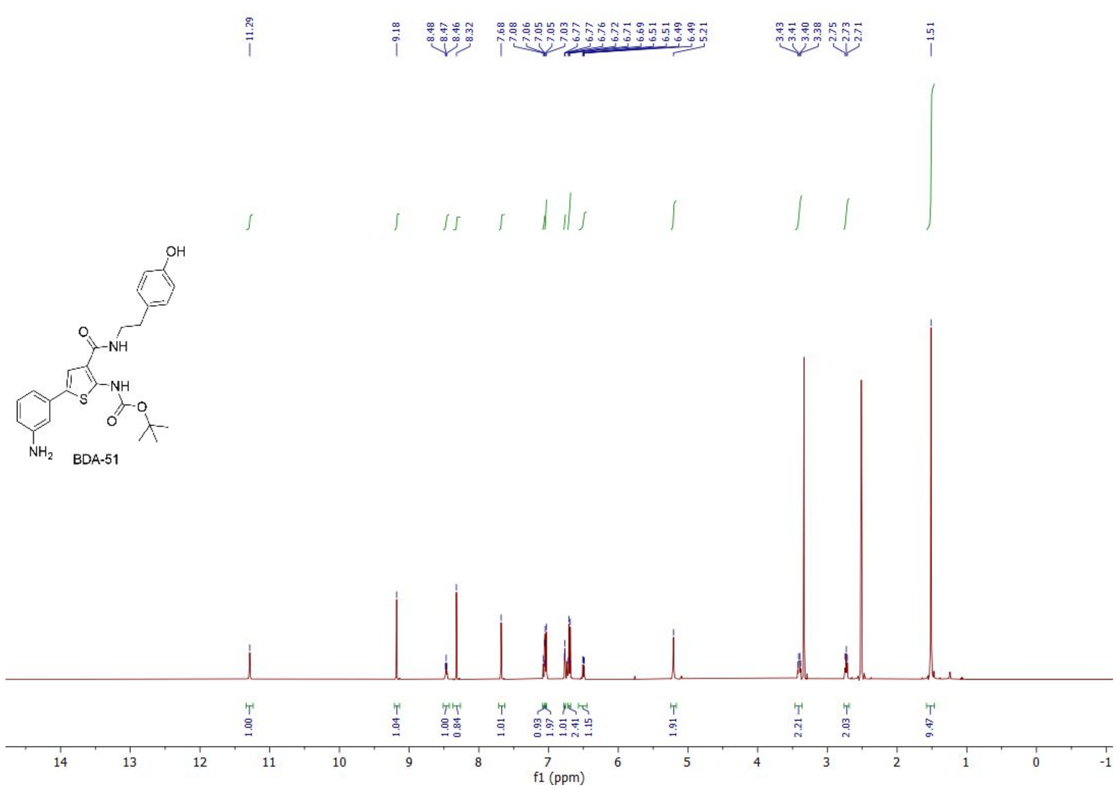

^{13}C{^{1}H} NMR spectrum of **BDA-51** (126 MHz, DMSO-d_6)

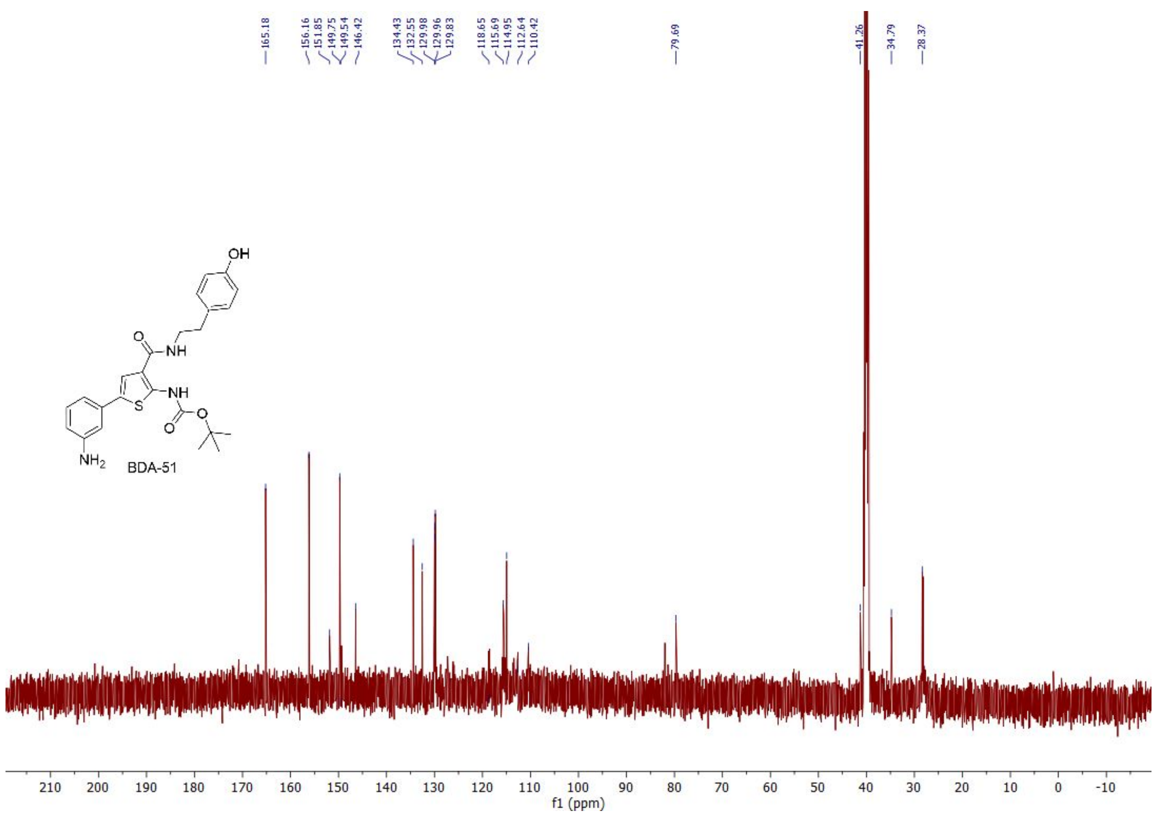

^{1}H NMR spectrum of **BDA-52** (500 MHz, $CDCl_3$)

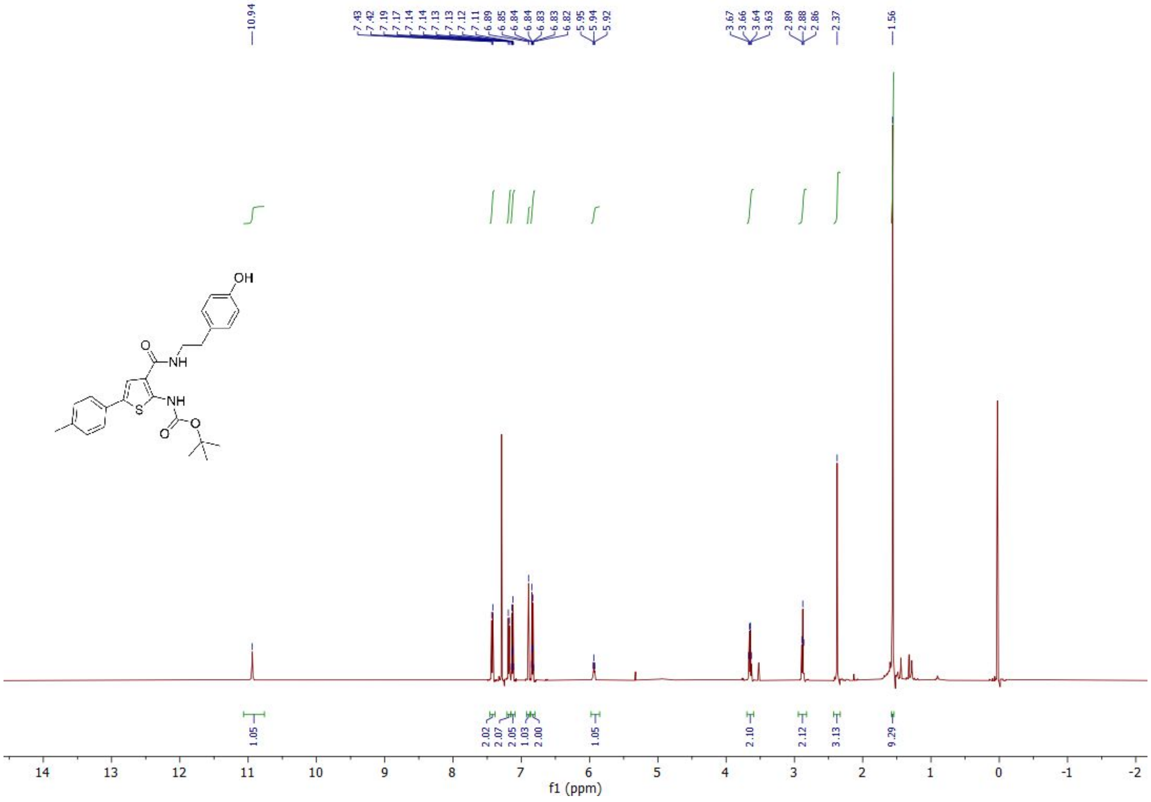

$^{13}C\{^{1}H\}$ NMR spectrum of **BDA-52** (126 MHz, $CDCl_3$)

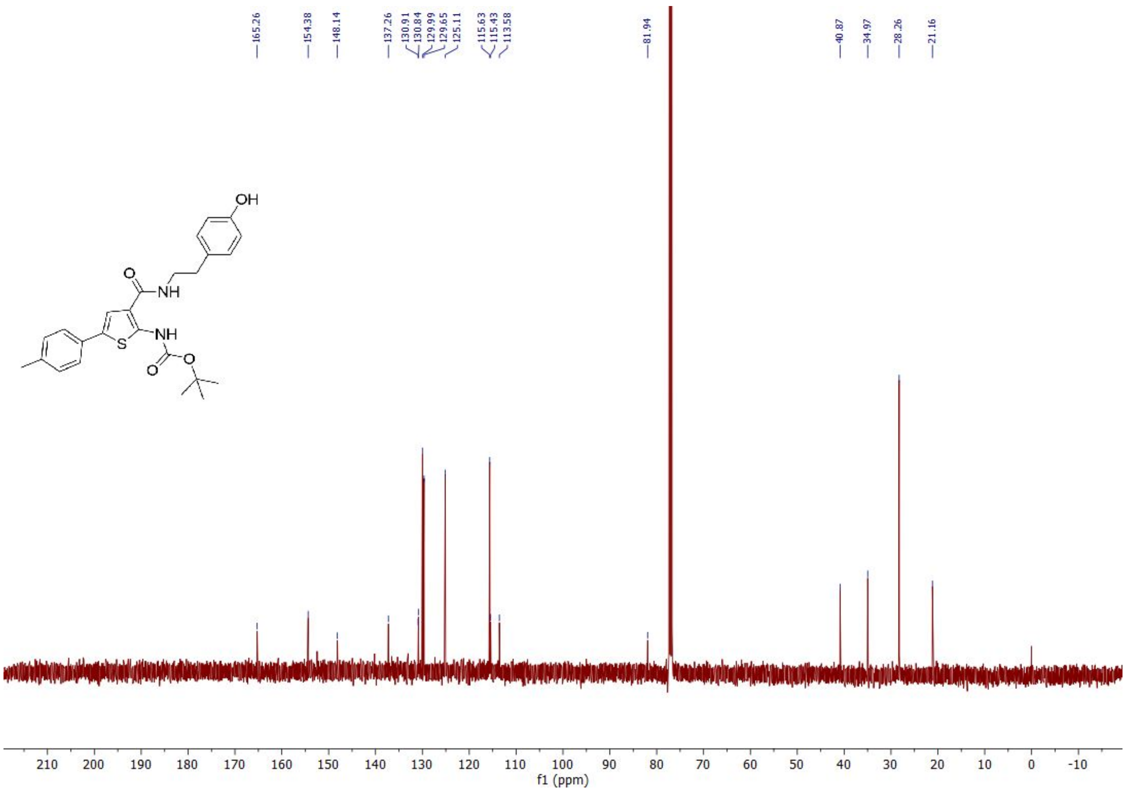

^{1}H NMR spectrum of **BDA-53** (500 MHz, DMSO-d_6)

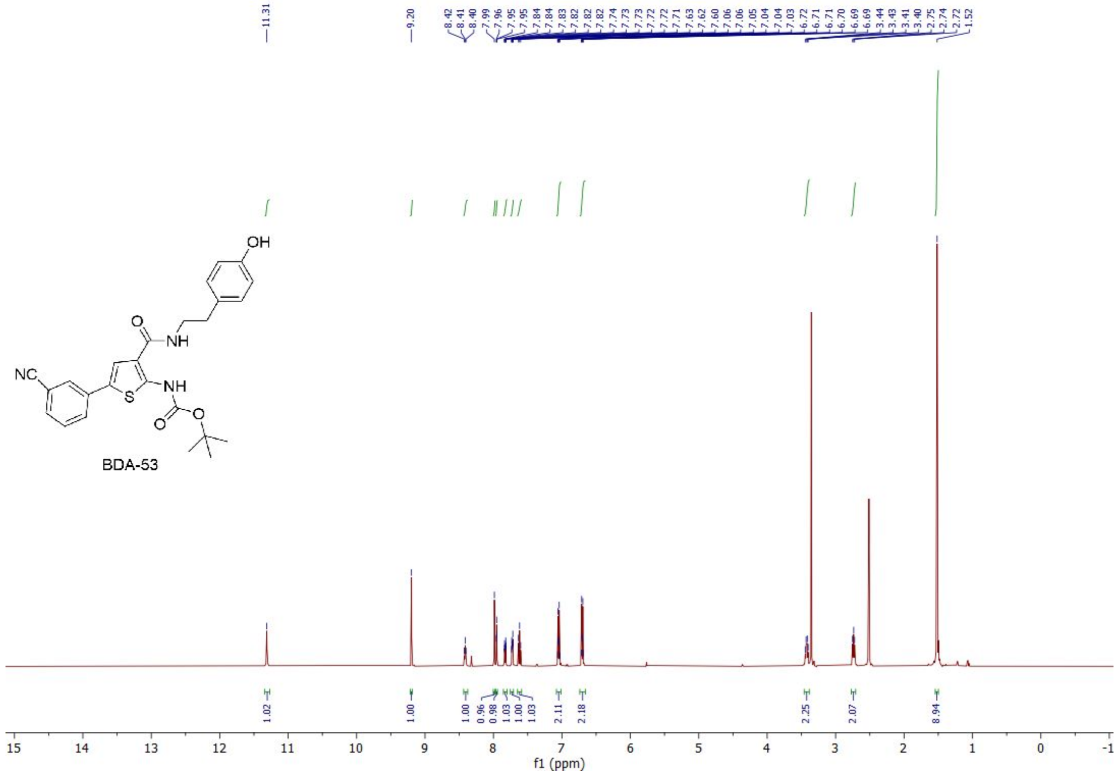

$^{13}C\{^{1}H\}$ NMR spectrum of **BDA-53** (126 MHz, DMSO-d_6)

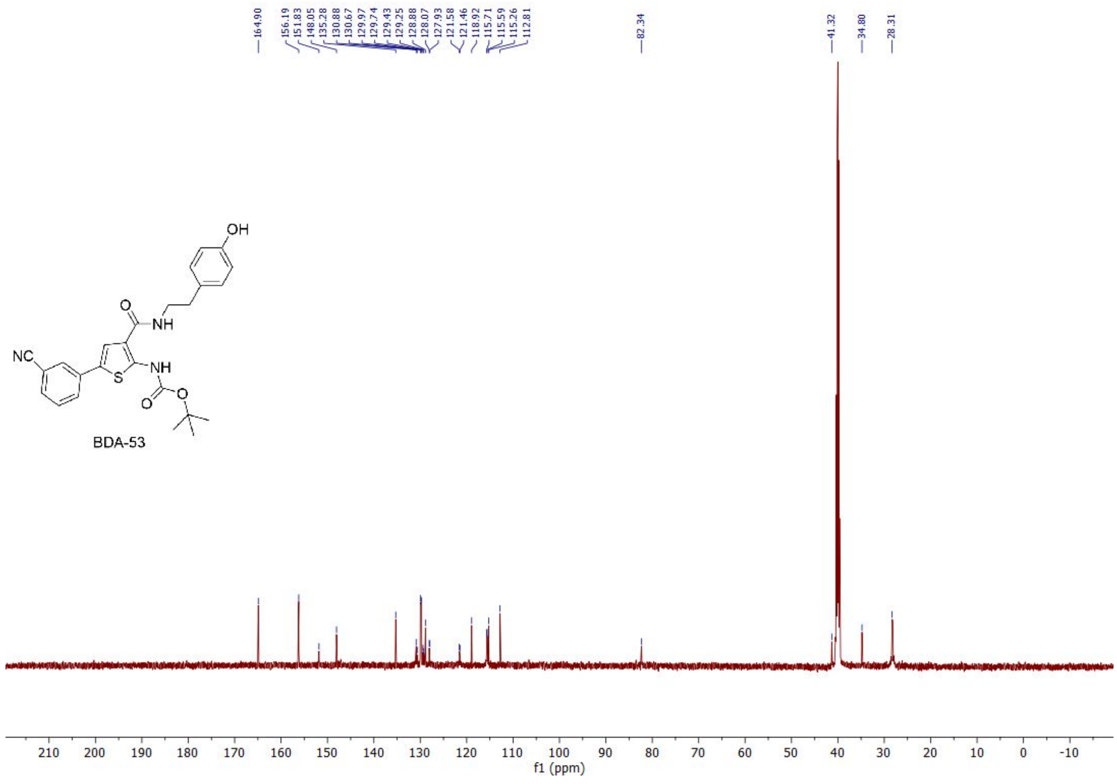

^{1}H NMR spectrum of **BDA-54** (500 MHz, $CDCl_3$)

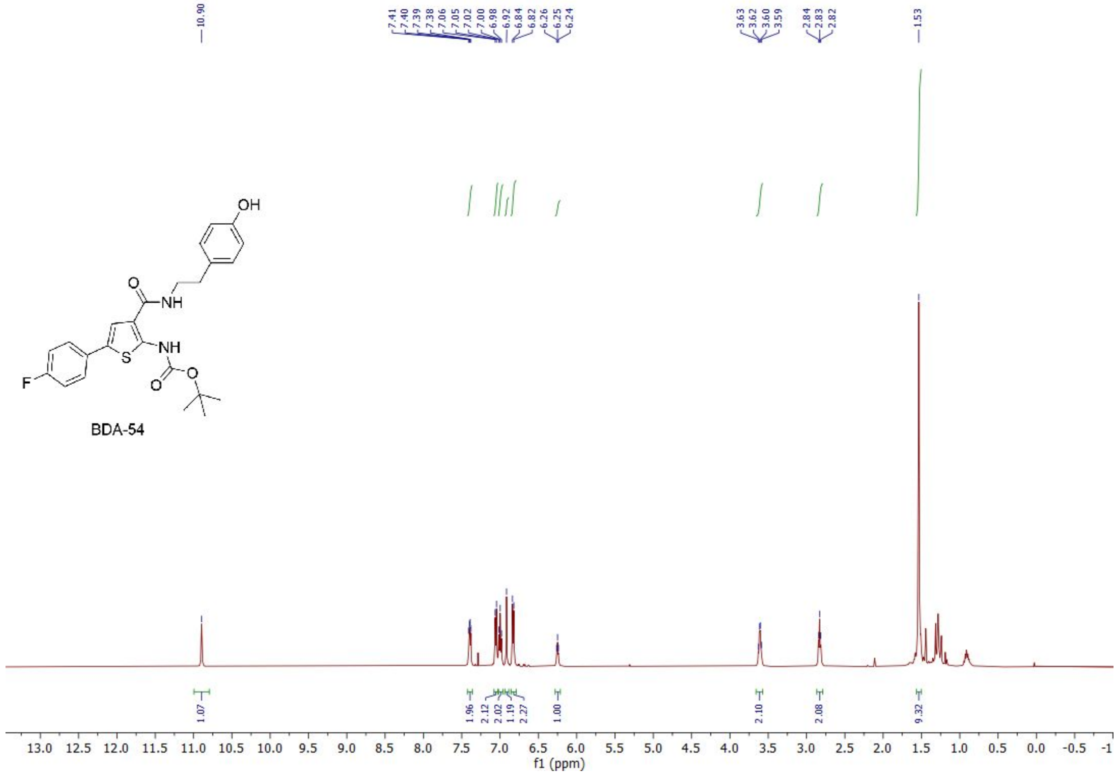

$^{13}C\{^{1}H\}$ NMR spectrum of **BDA-54** (126 MHz, $CDCl_3$)

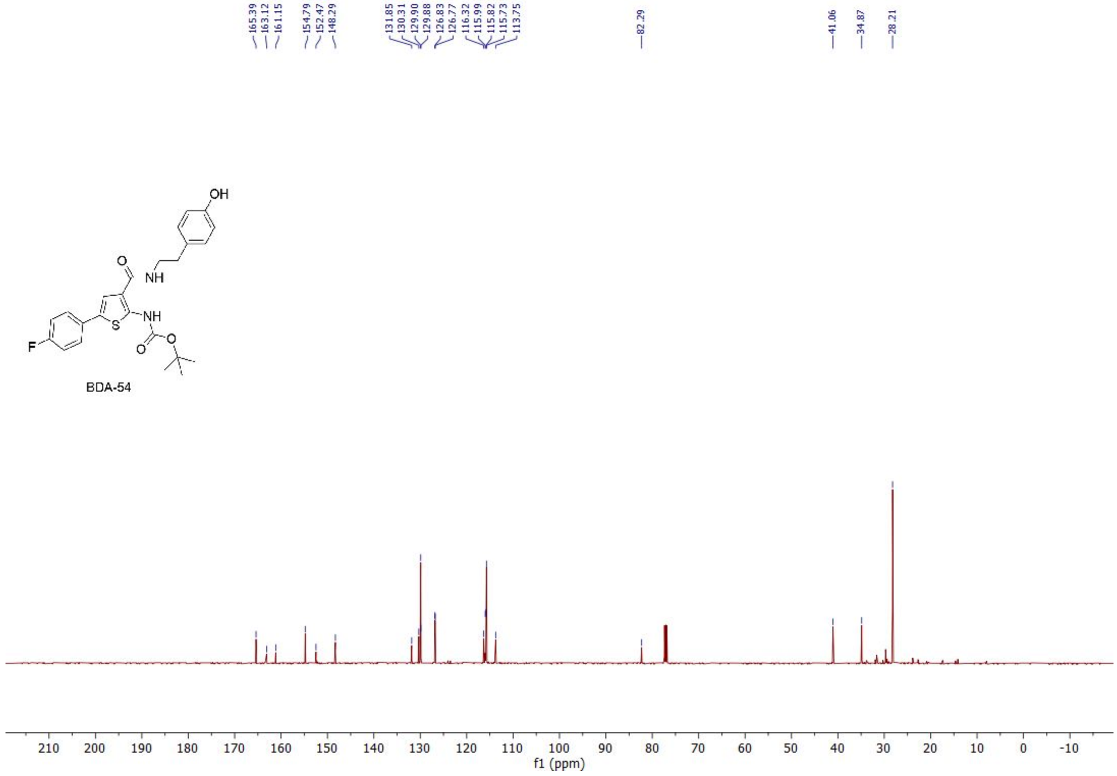

^{1}H NMR spectrum of **BDA-55** (500 MHz, DMSO-d_6)

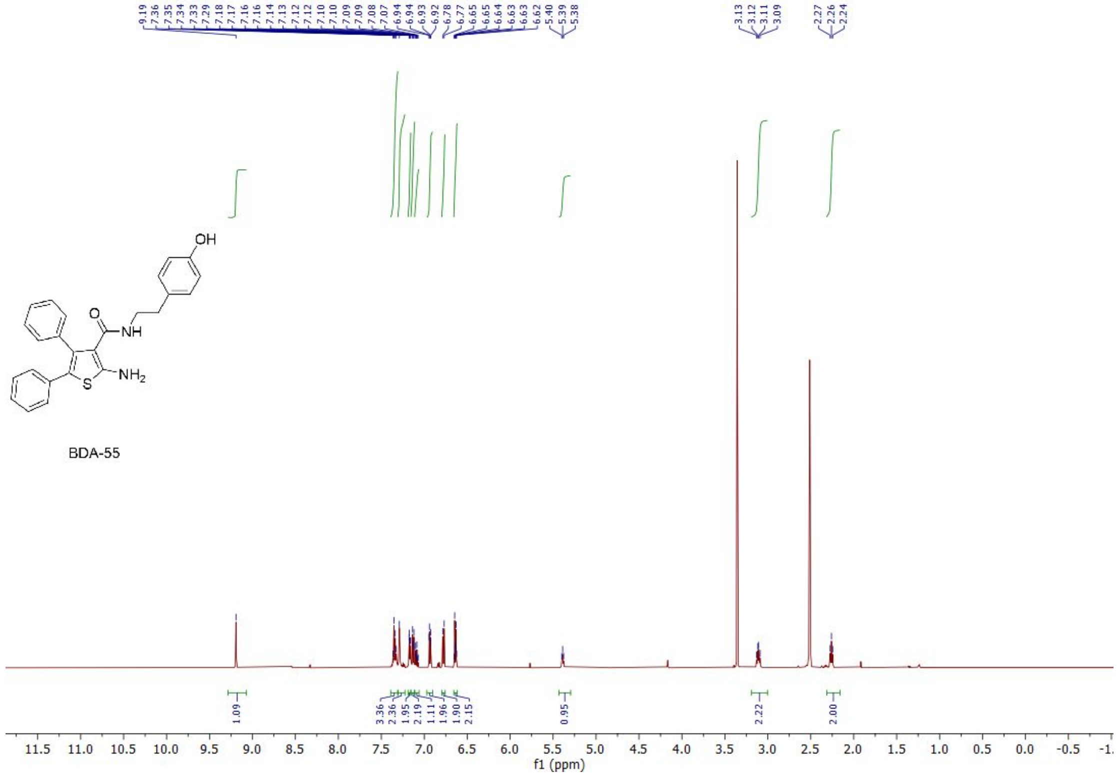

$^{13}C\{^{1}H\}$ NMR spectrum of **BDA-55** (126 MHz, DMSO-d_6)

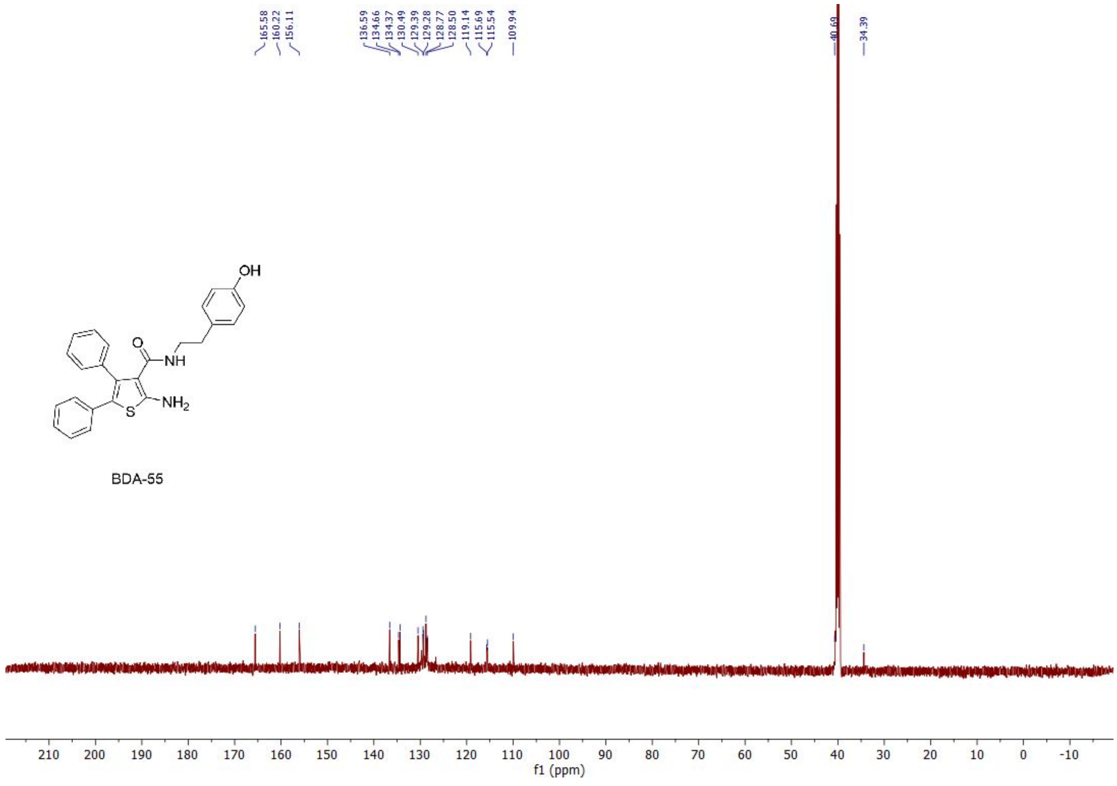

^{1}H NMR spectrum of **BDA-56** (500 MHz, $CDCl_3$)

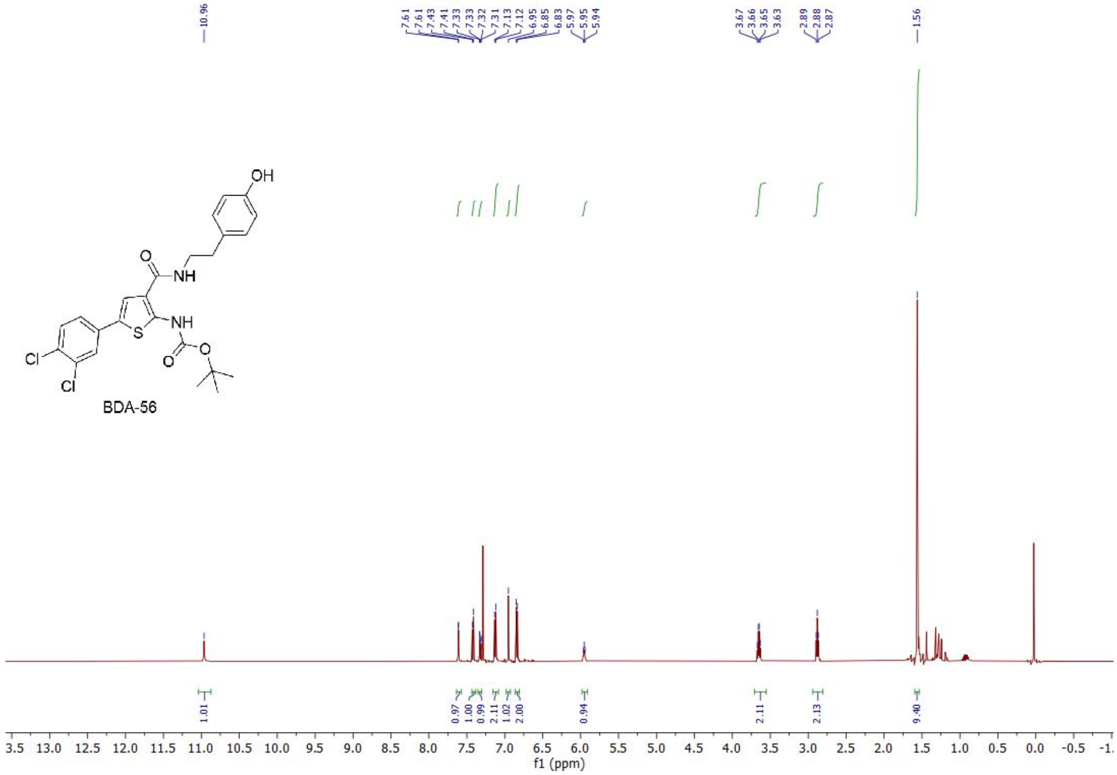

$^{13}C\{^{1}H\}$ NMR spectrum of **BDA-56** (126 MHz, $CDCl_3$)

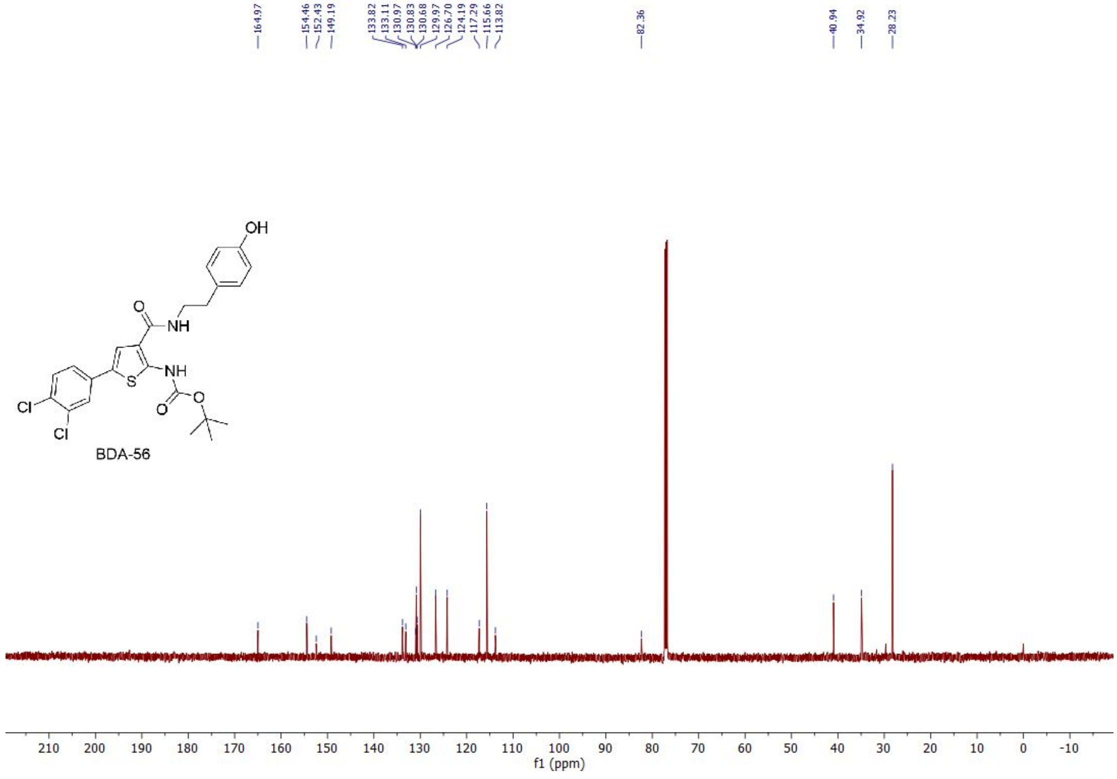

^{1}H NMR spectrum of **BDA-57** (500 MHz, $CDCl_3$)

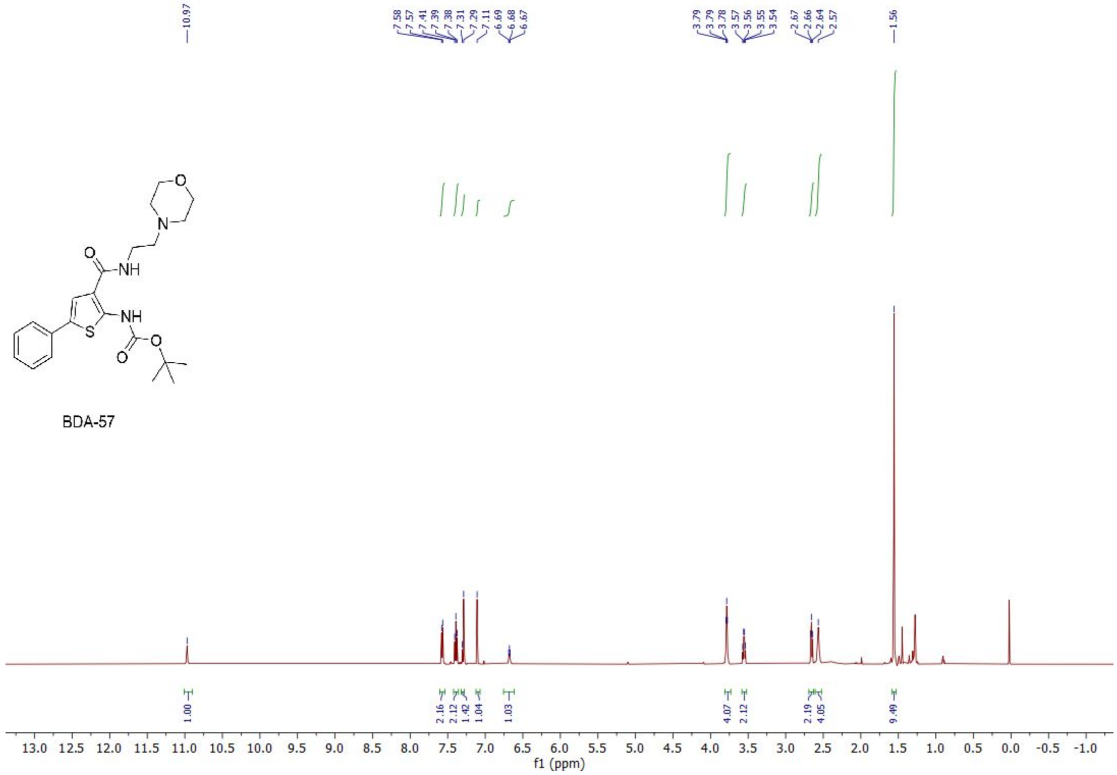

$^{13}C\{^{1}H\}$ NMR spectrum of **BDA-57** (126 MHz, $CDCl_3$)

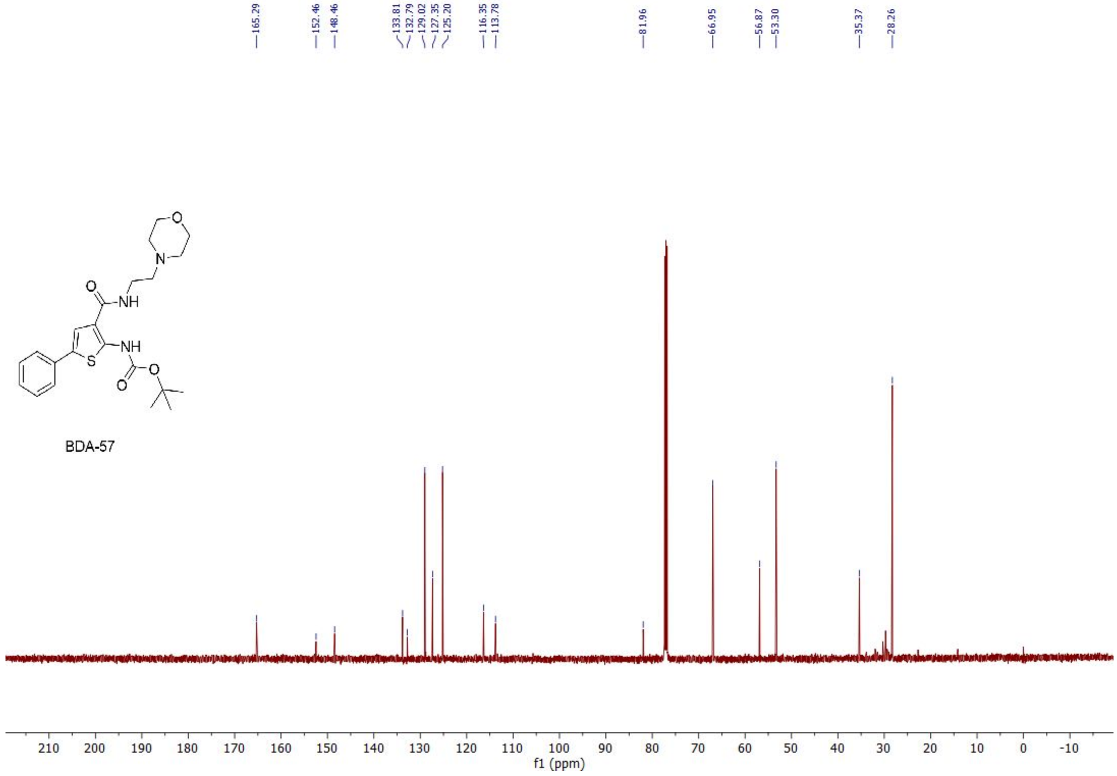

^{1}H NMR spectrum of **BDA-58** (500 MHz, $CDCl_3$)

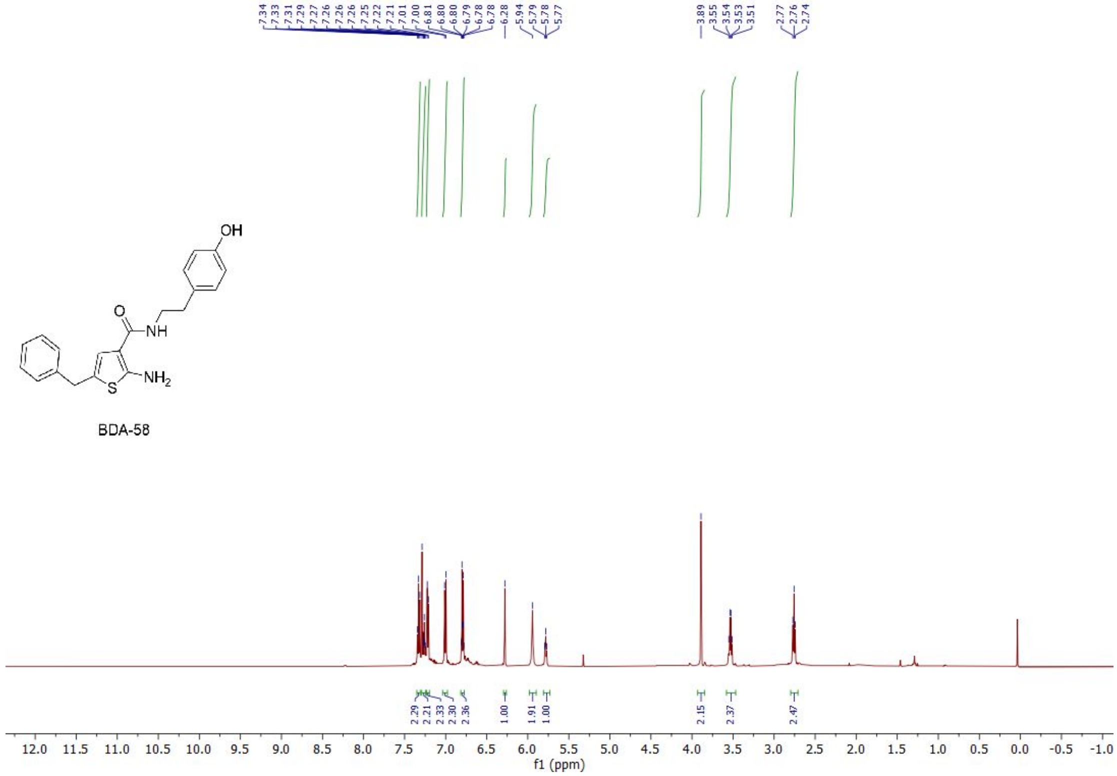

$^{13}C\{^{1}H\}$ NMR spectrum of **BDA-58** (126 MHz, $CDCl_3$)

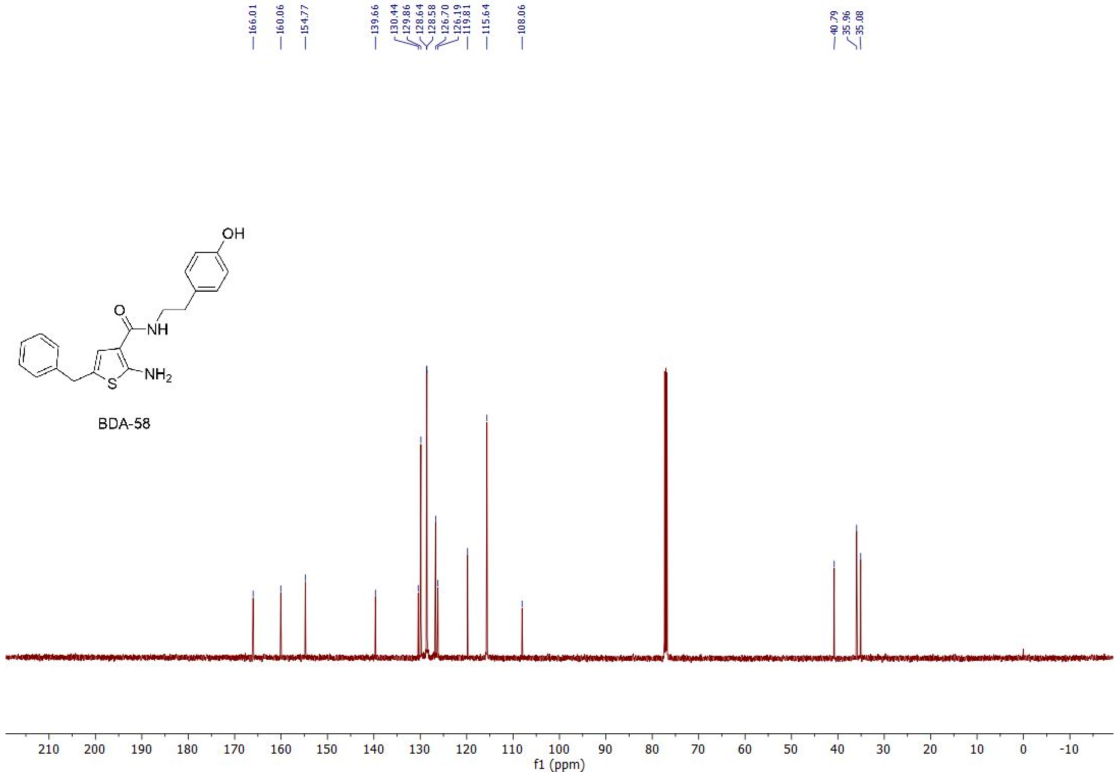

^{1}H NMR spectrum of **BDA-59** (500 MHz, $CDCl_3$)

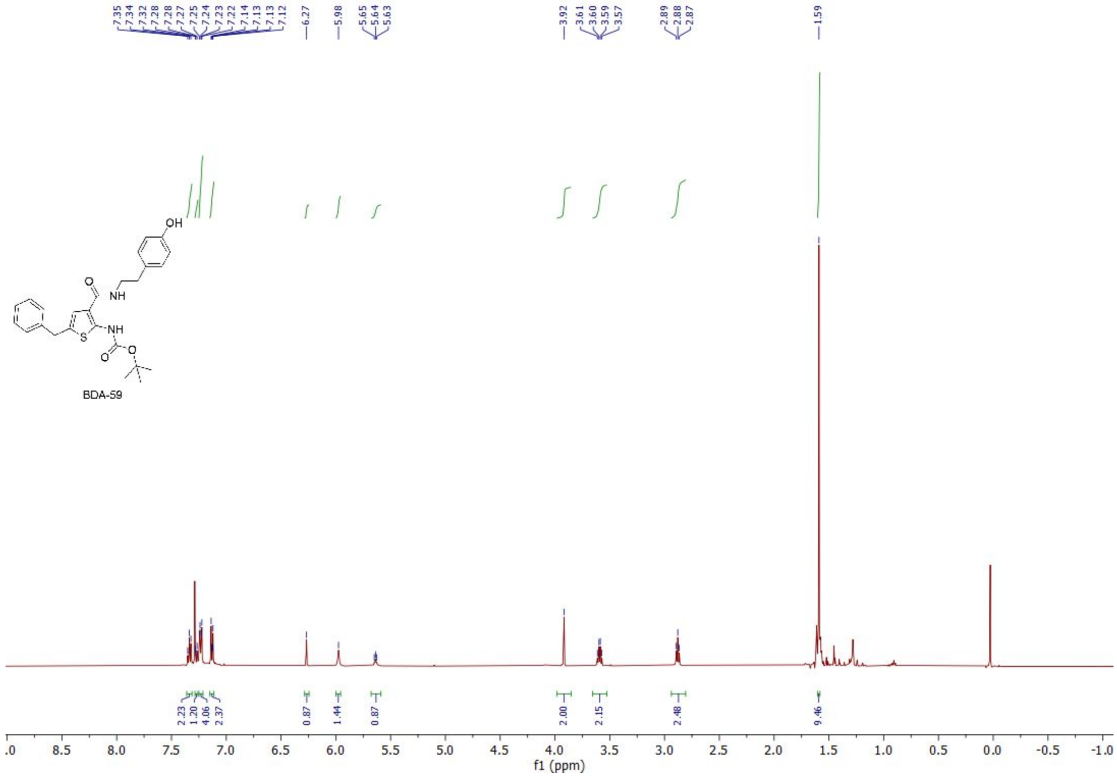

$^{13}C\{^{1}H\}$ NMR spectrum of **BDA-59** (126 MHz, $CDCl_3$)

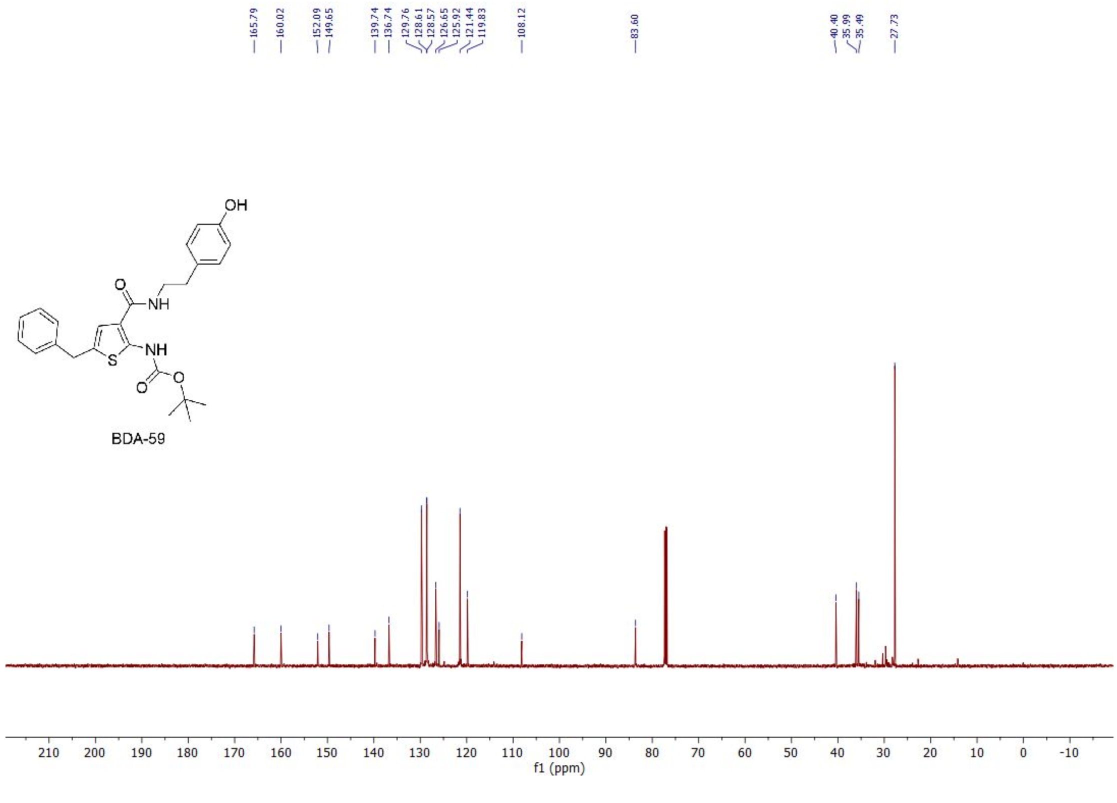

^{1}H NMR spectrum of **BDA-60** (500 MHz, $CDCl_3$)

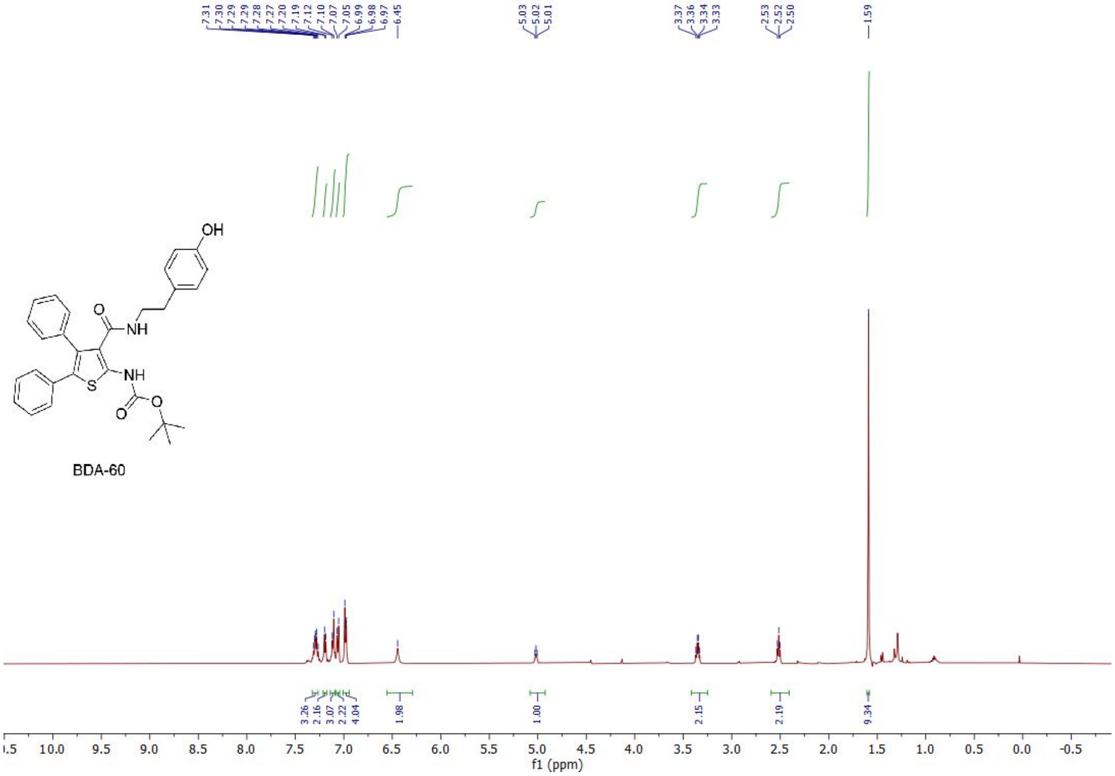

$^{13}C\{^{1}H\}$ NMR spectrum of **BDA-60** (126 MHz, $CDCl_3$)

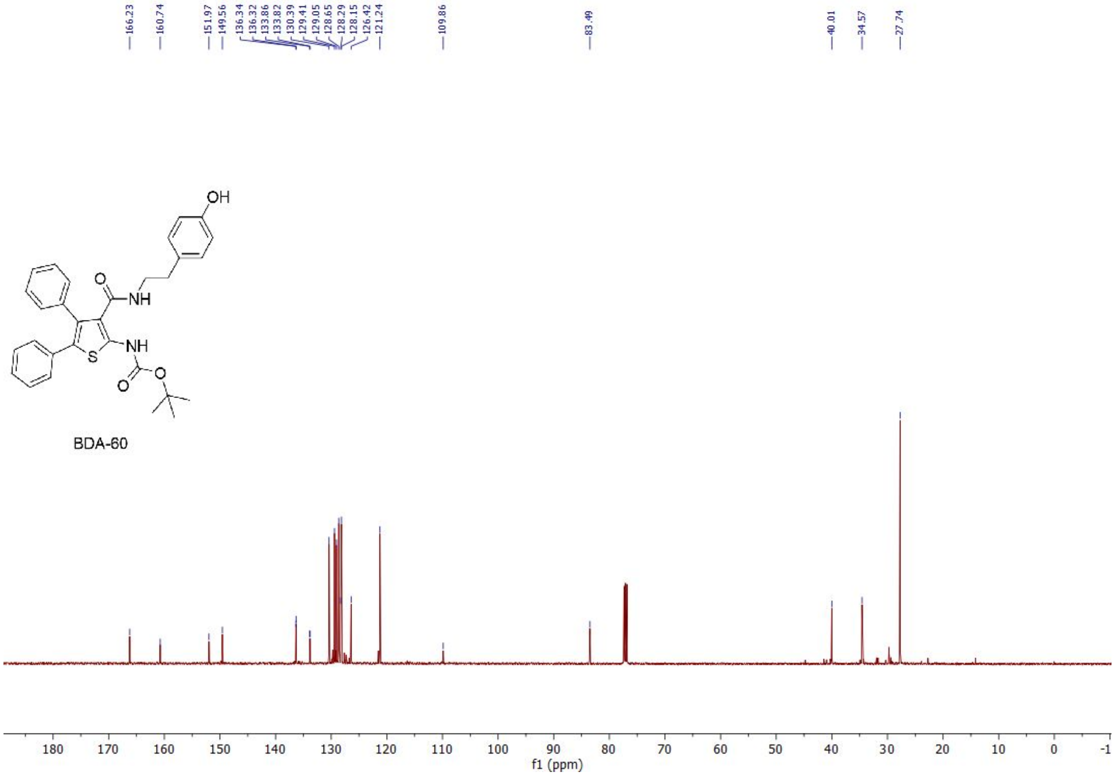

^{1}H NMR spectrum of **BDA-61** (500 MHz, DMSO-d_6)

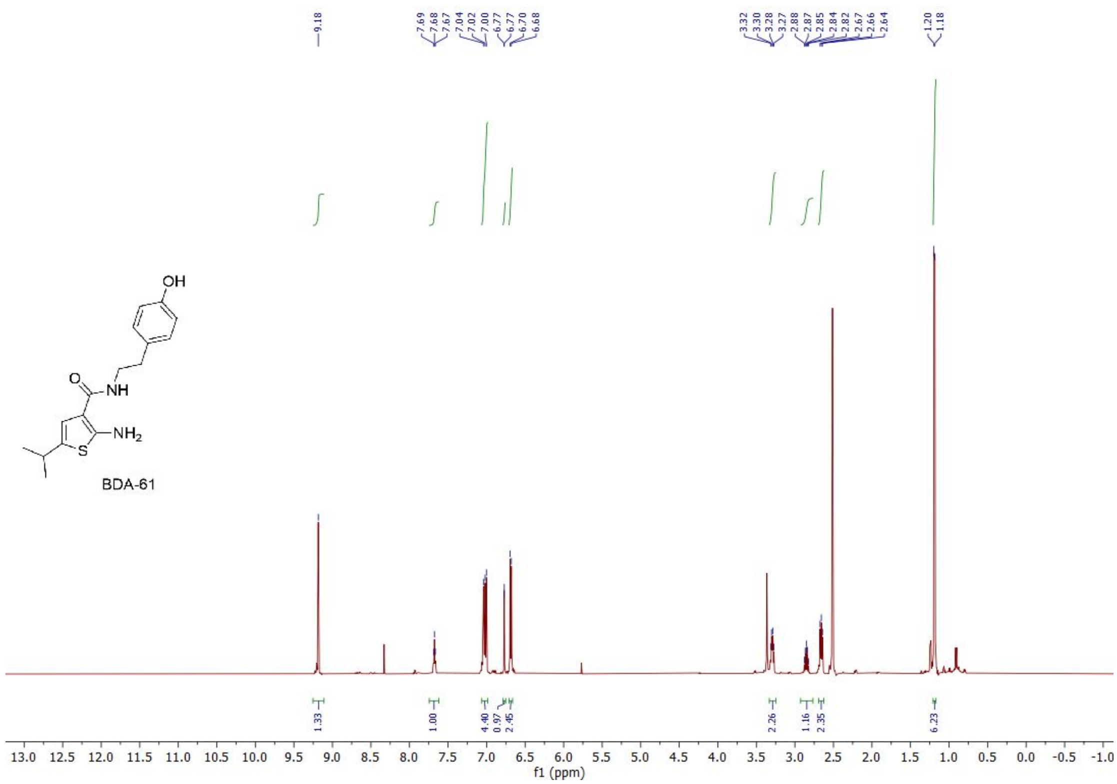

$^{13}C\{^{1}H\}$ NMR spectrum of **BDA-61** (126 MHz, DMSO-d_6)

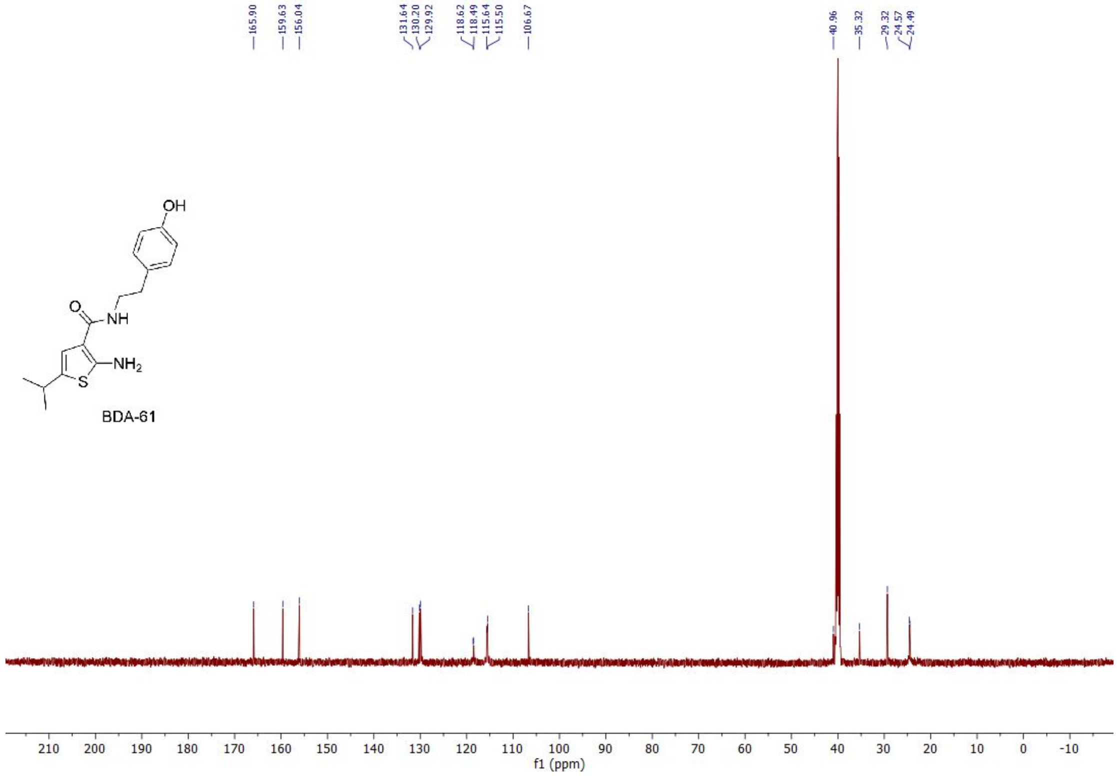

^{1}H NMR spectrum of **BDA-62** (500 MHz, $CDCl_3$)

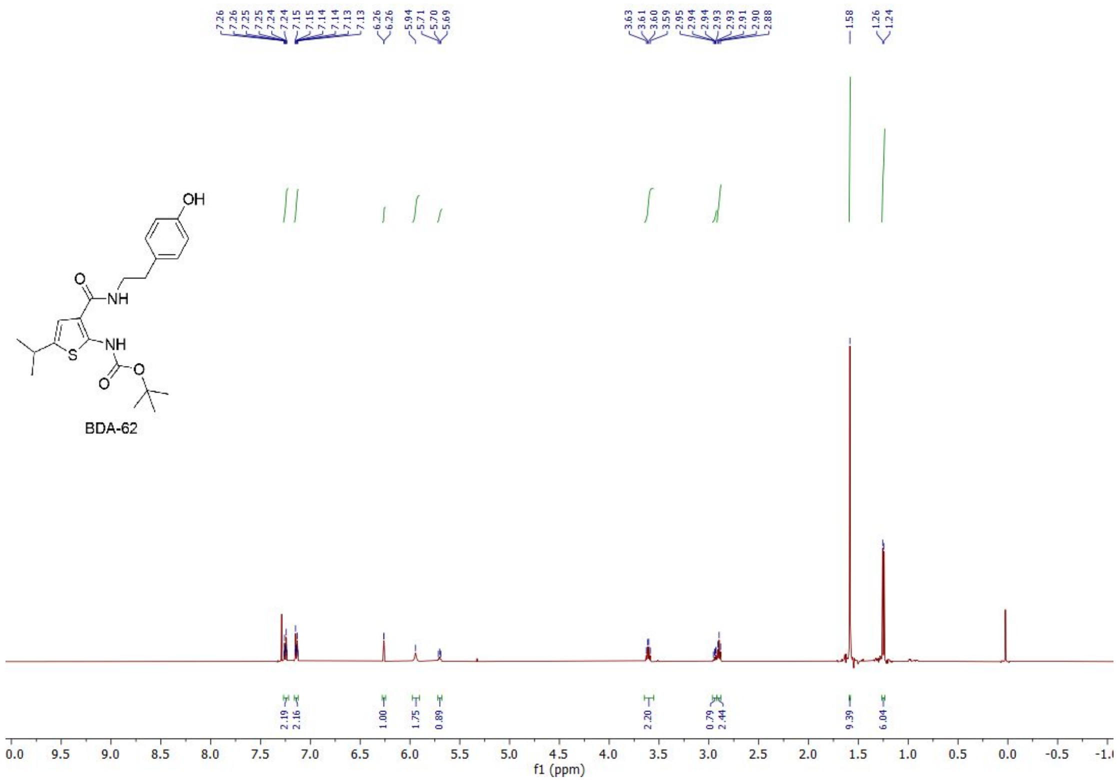

$^{13}C\{^{1}H\}$ NMR spectrum of **BDA-62** (126 MHz, $CDCl_3$)

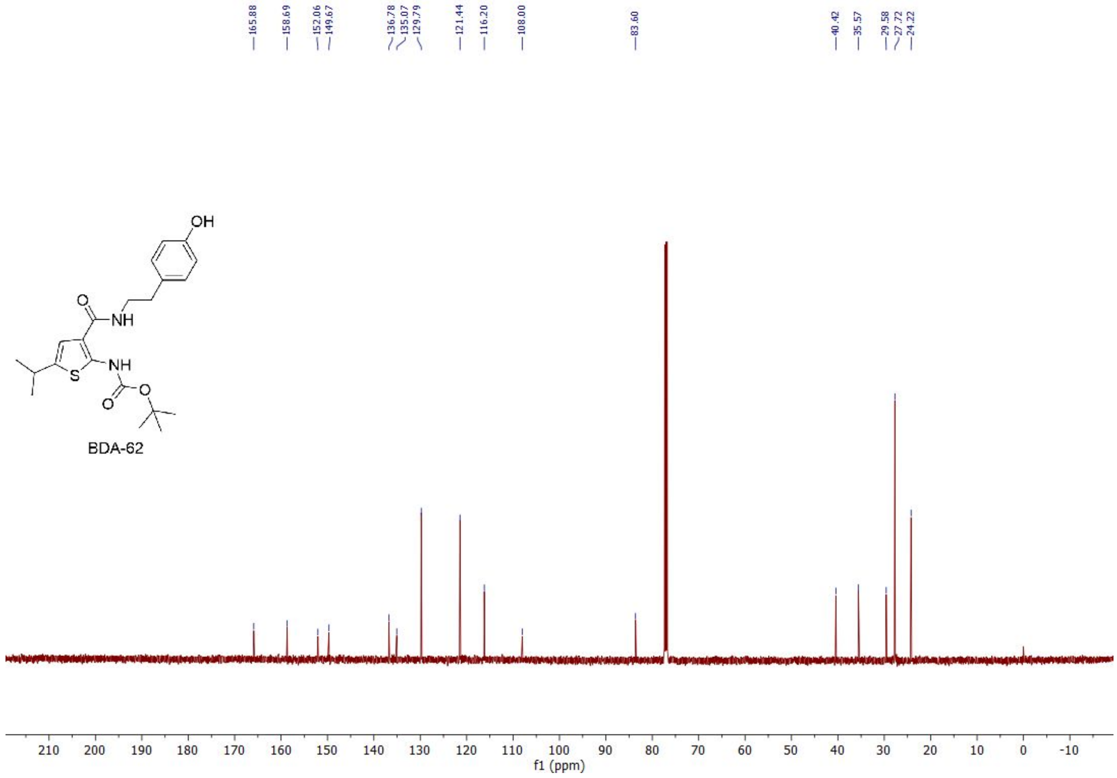

^{1}H NMR spectrum of **BDA-63** (500 MHz, DMSO-d_6)

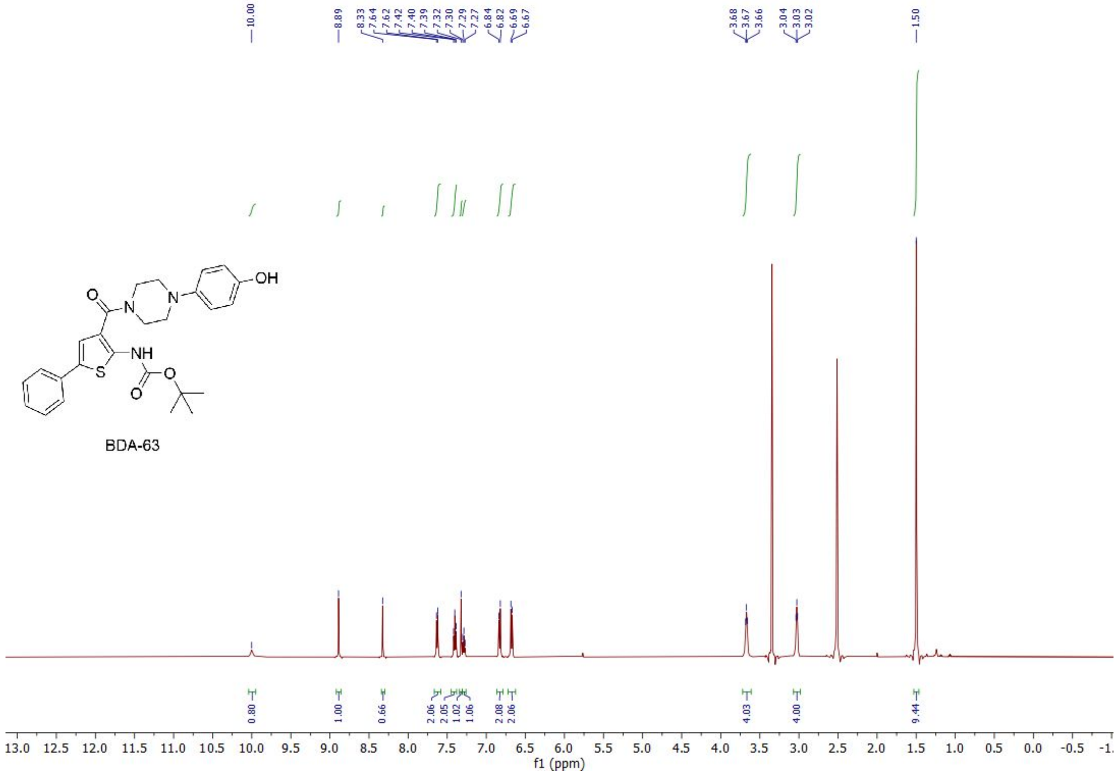

$^{13}C\{^{1}H\}$ NMR spectrum of **BDA-63** (126 MHz, DMSO-d_6)

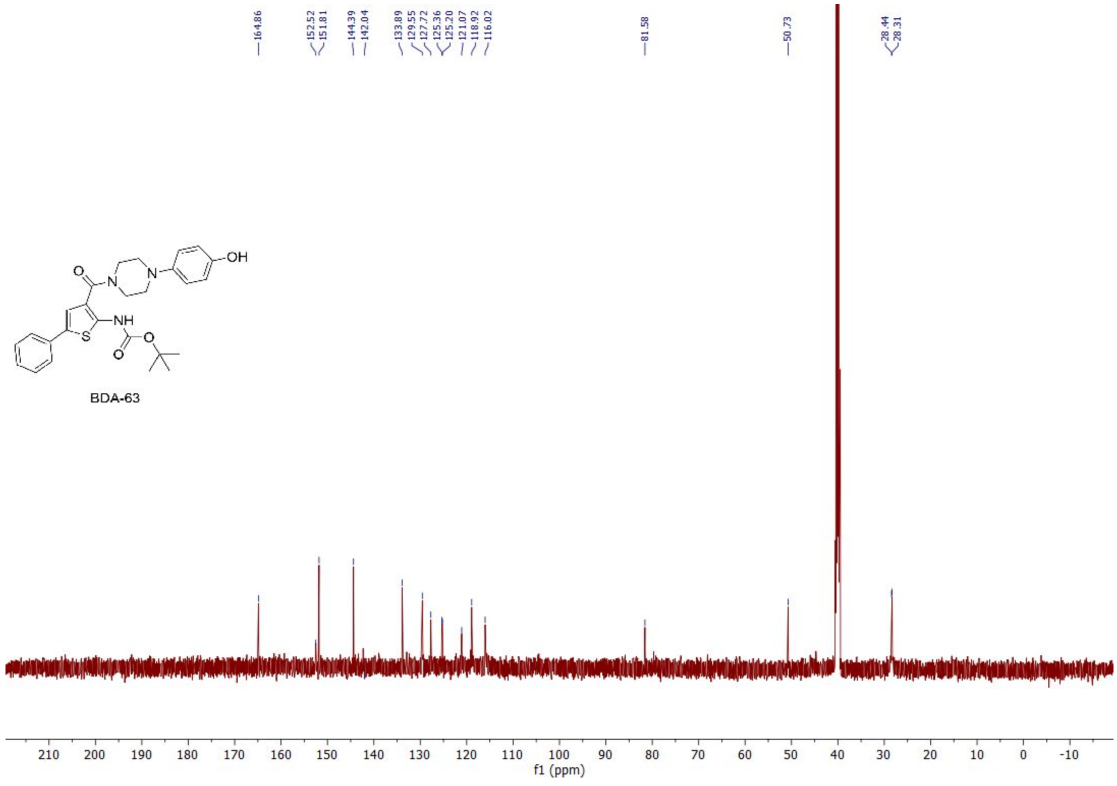

^{1}H NMR spectrum of **BDA-64** (500 MHz, $CDCl_3$)

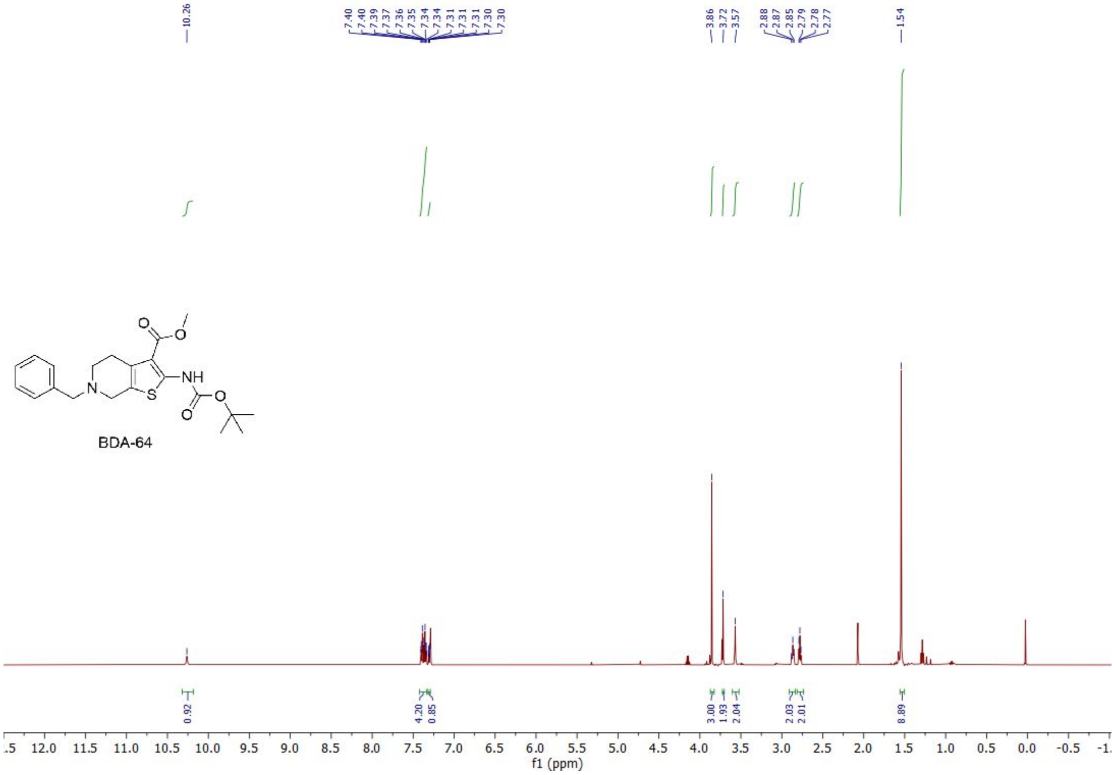

$^{13}C\{^{1}H\}$ NMR spectrum of **BDA-64** (126 MHz, $CDCl_3$)

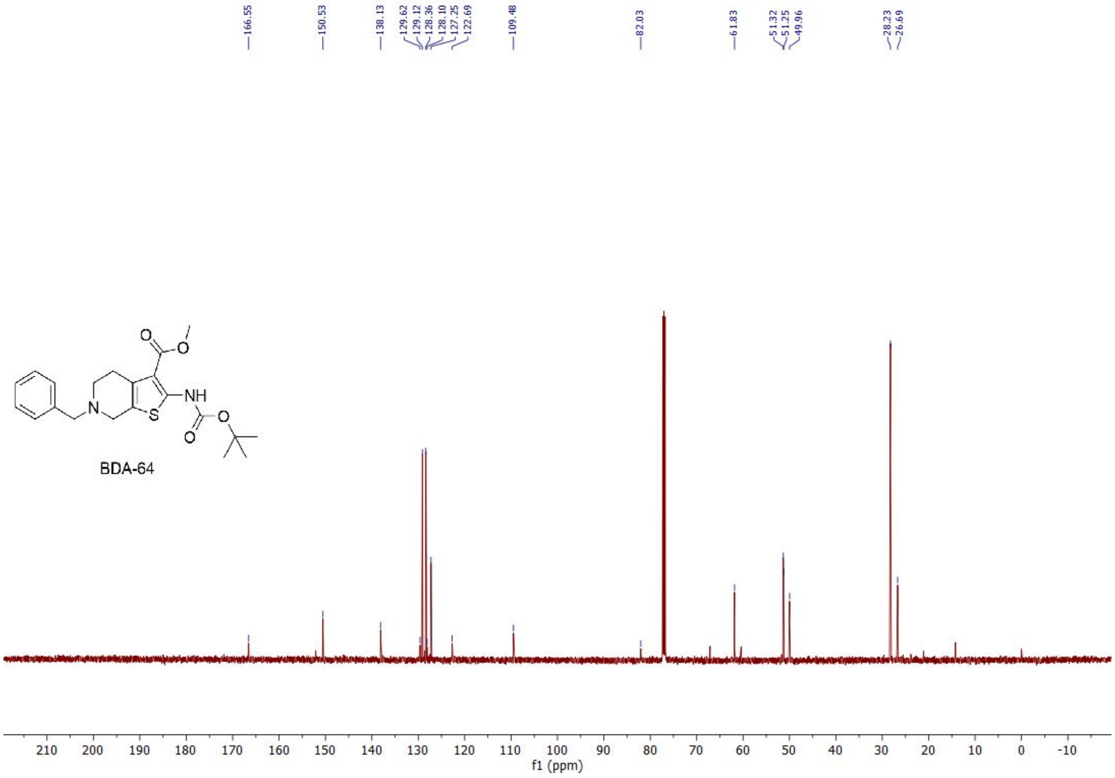

^{1}H NMR spectrum of **BDA-65** (500 MHz, $CDCl_3$)

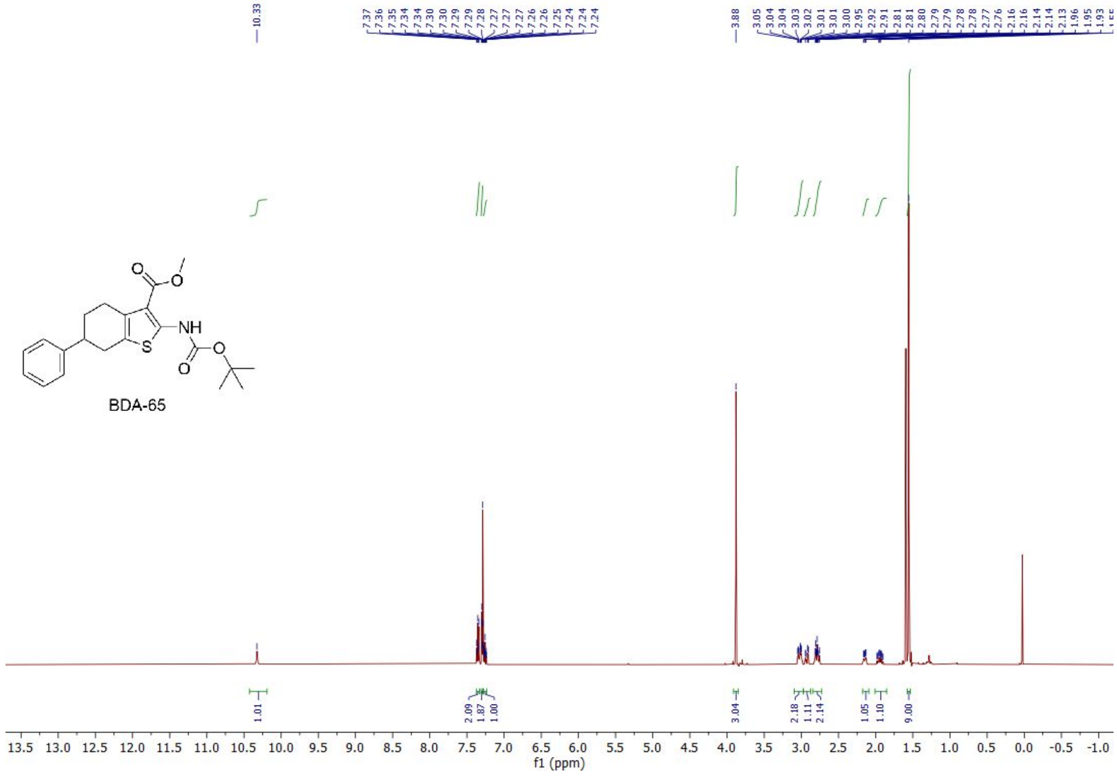

$^{13}C\{^{1}H\}$ NMR spectrum of **BDA-65** (126 MHz, $CDCl_3$)

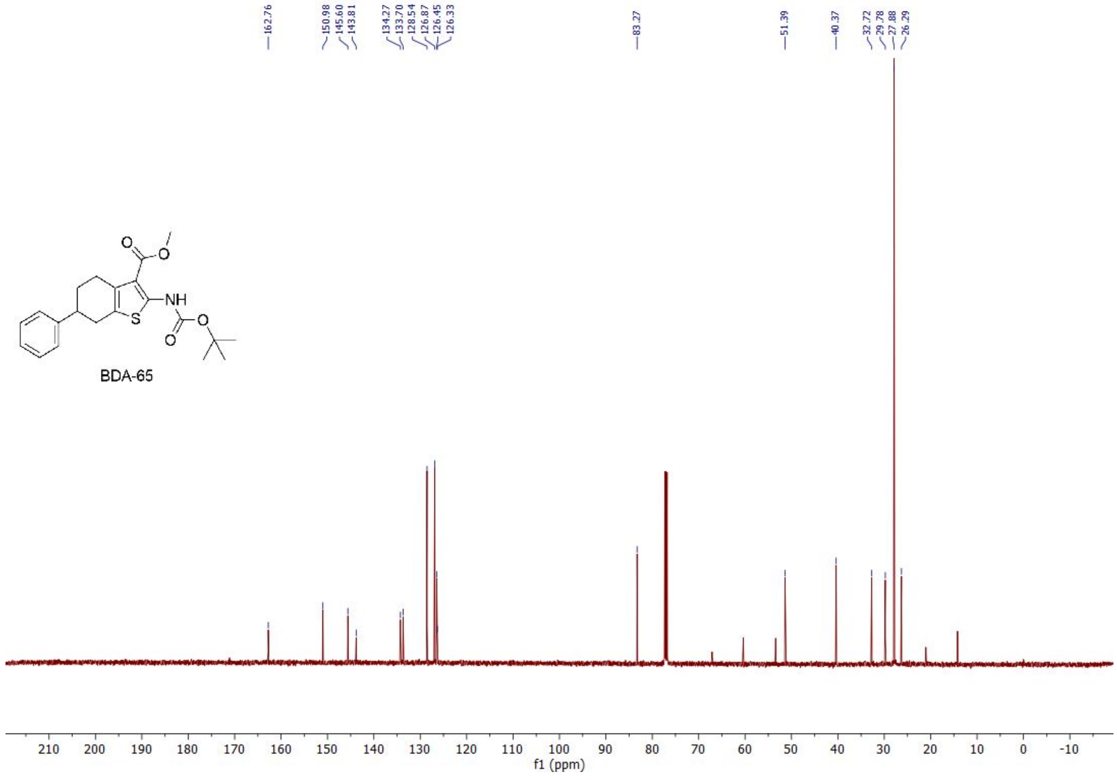

^{1}H NMR spectrum of **BDA-66** (500 MHz, $CDCl_3$)

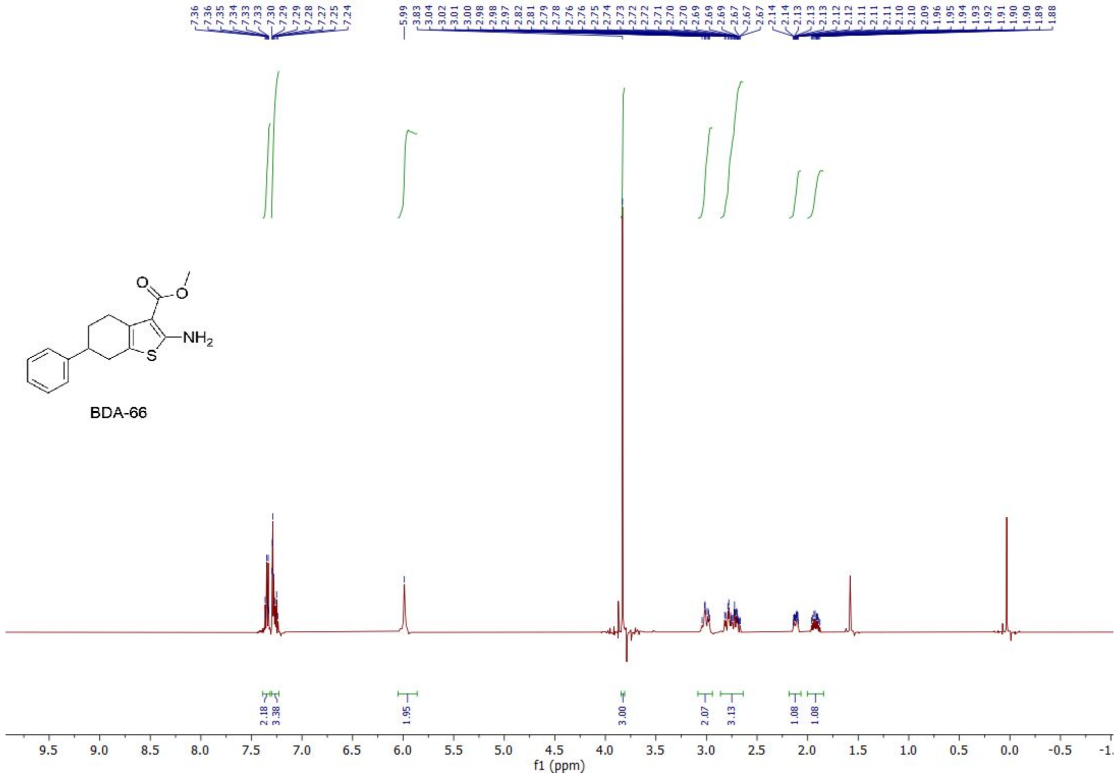

$^{13}C\{^{1}H\}$ NMR spectrum of **BDA-66** (126 MHz, $CDCl_3$)

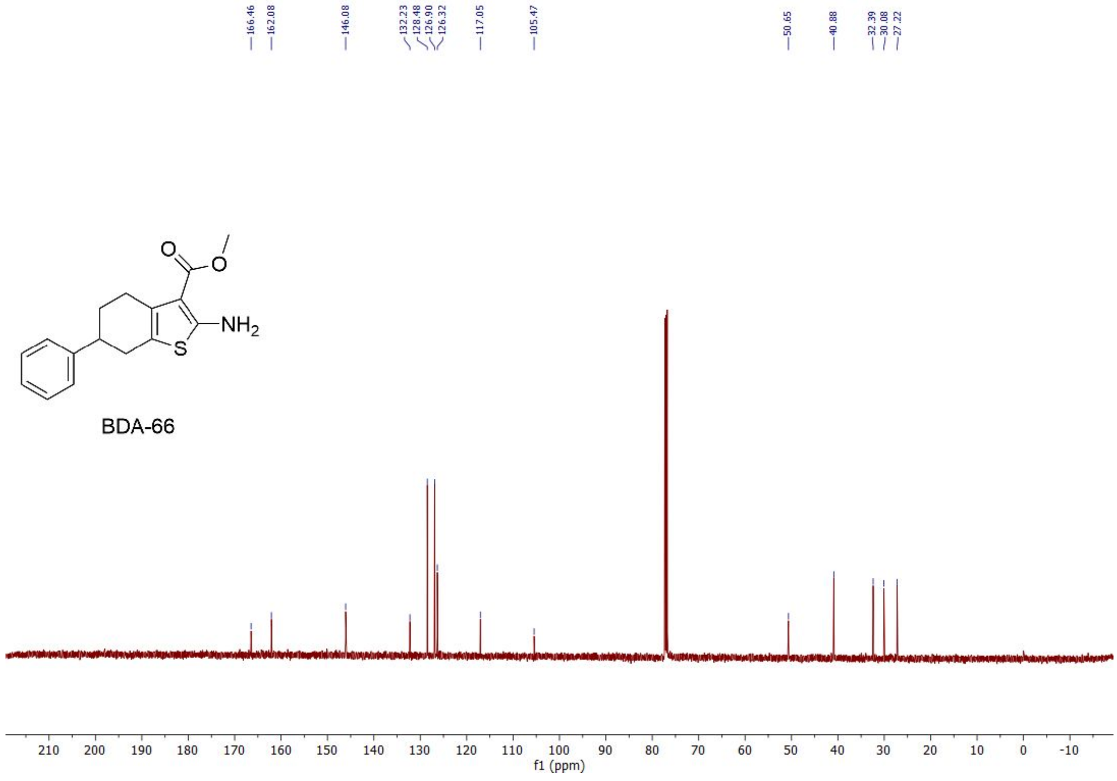

^{1}H NMR spectrum of **BDA-67** (500 MHz, $CDCl_3$)

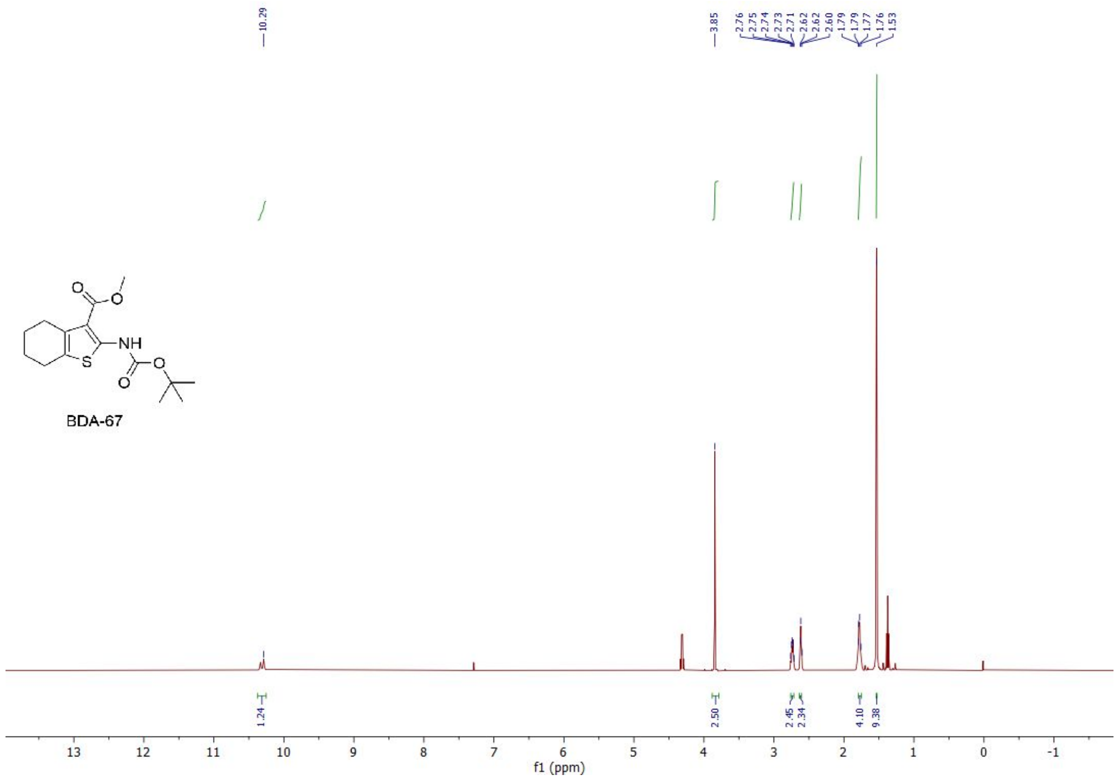

$^{13}C\{^{1}H\}$ NMR spectrum of **BDA-67** (126 MHz, $CDCl_3$)

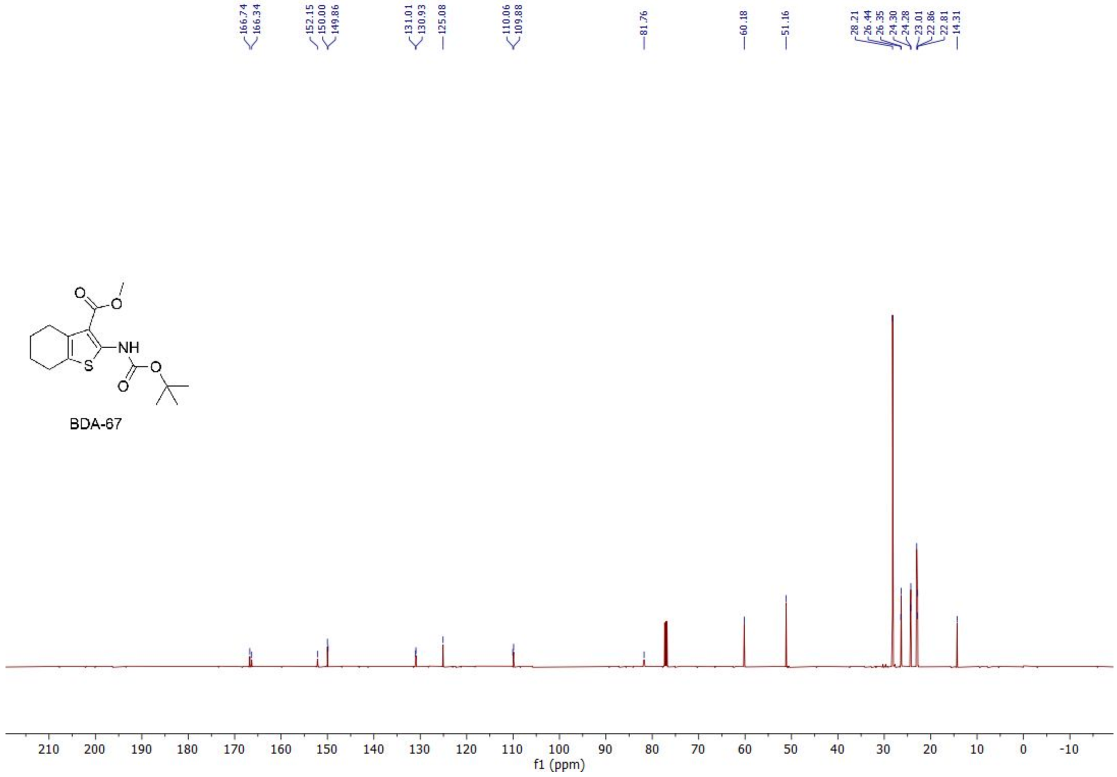

^{1}H NMR spectrum of **BDA-68** (500 MHz, $CDCl_3$)

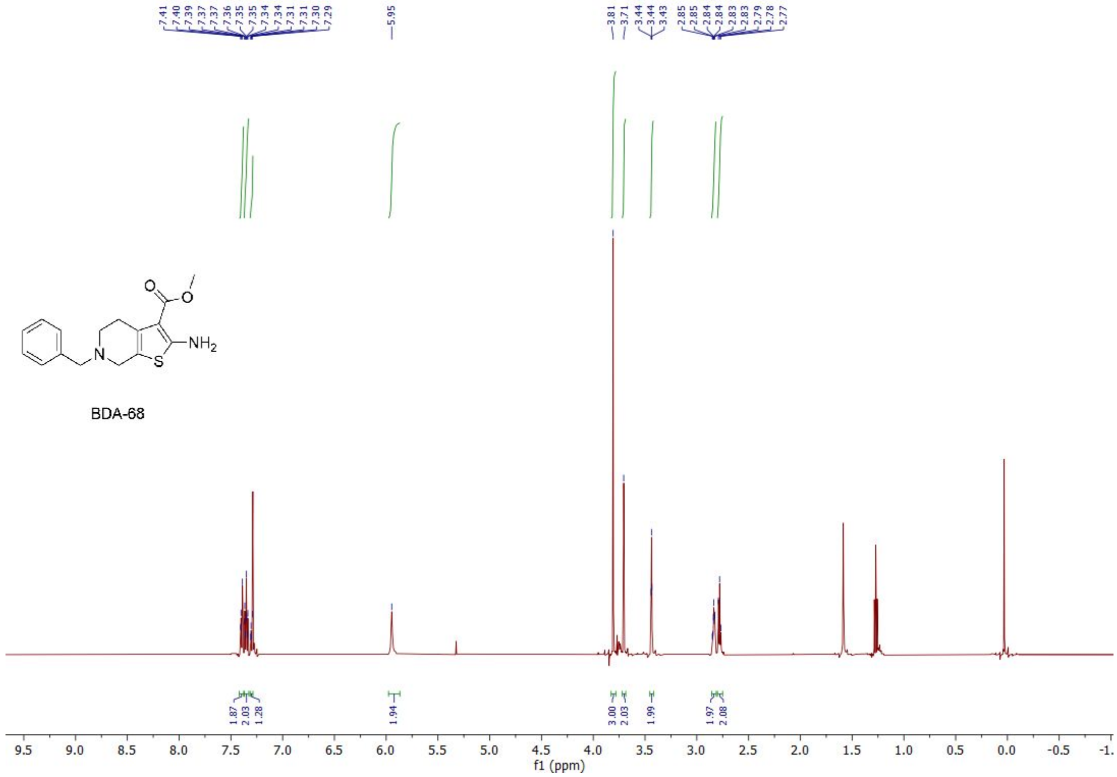

^{13}C{^{1}H} NMR spectrum of **BDA-68** (126 MHz, $CDCl_3$)

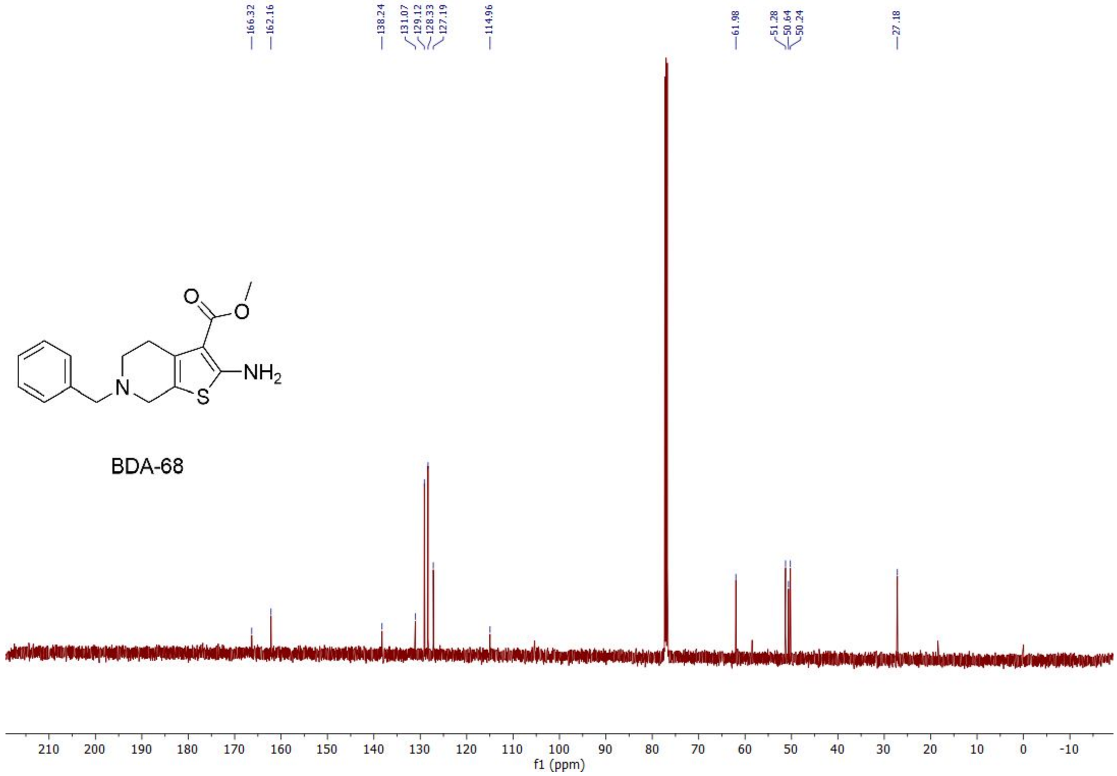

^{1}H NMR spectrum of **BDA-69** (500 MHz, $CDCl_3$)

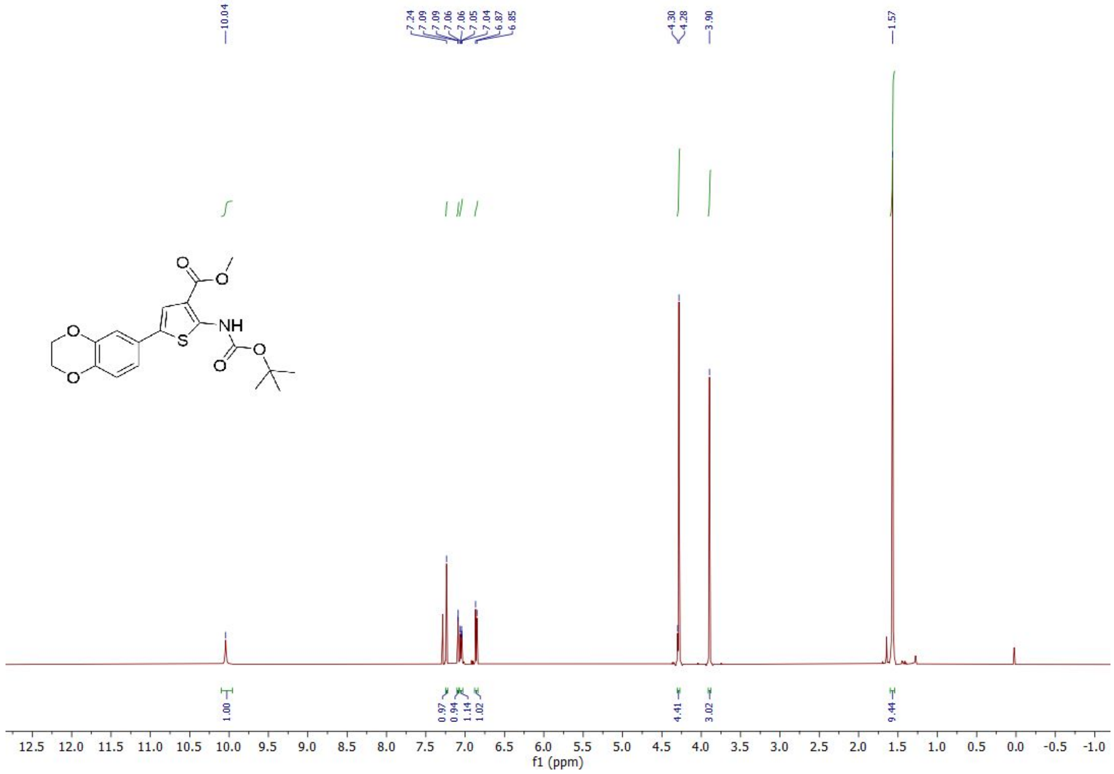

^{13}C{^{1}H} NMR spectrum of **BDA-69** (126 MHz, $CDCl_3$)

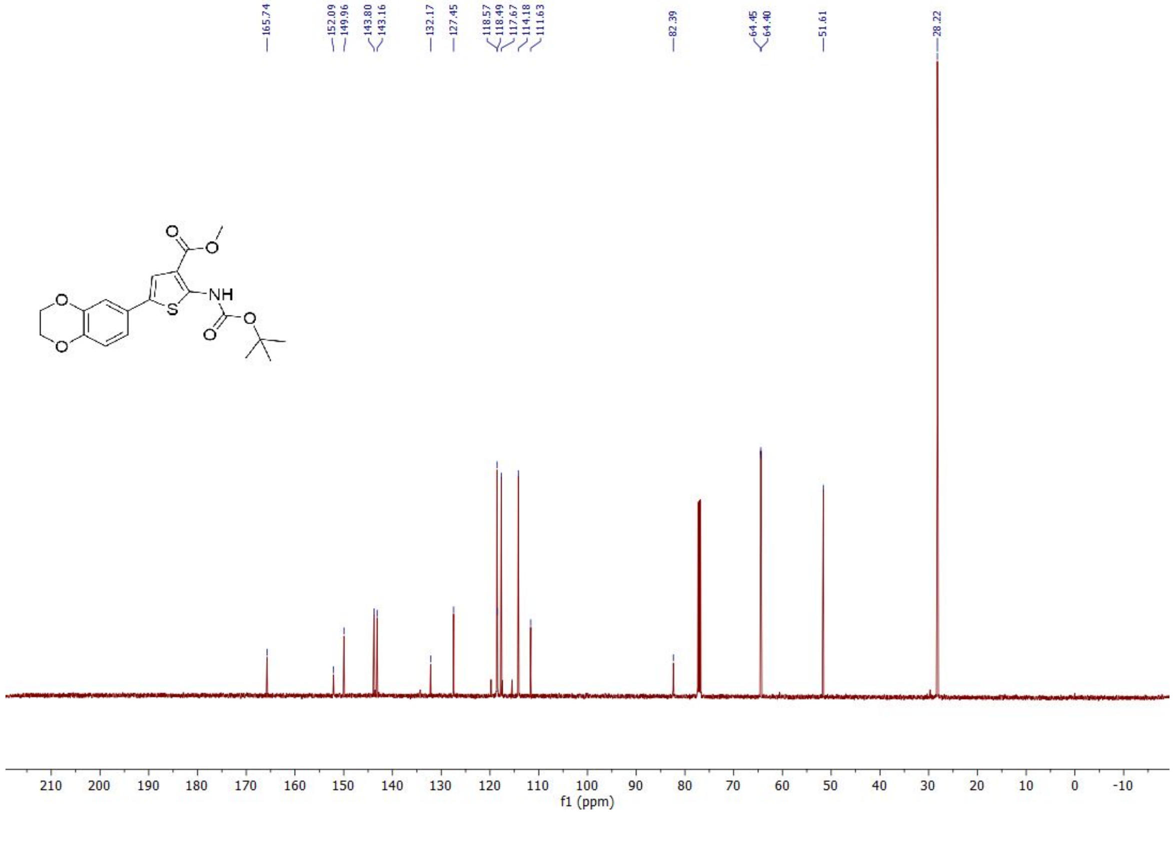

^{1}H NMR spectrum of **BDA-70** (500 MHz, $CDCl_3$)

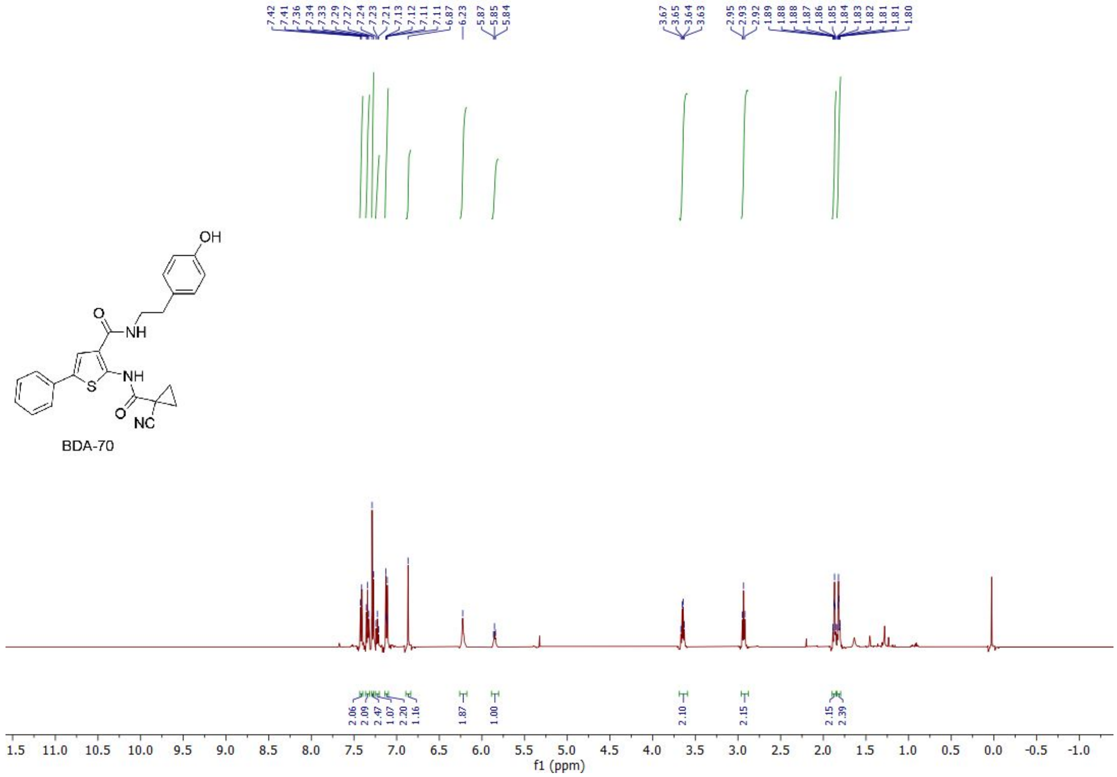

$^{13}C\{^{1}H\}$ NMR spectrum of **BDA-70** (126 MHz, $CDCl_3$)

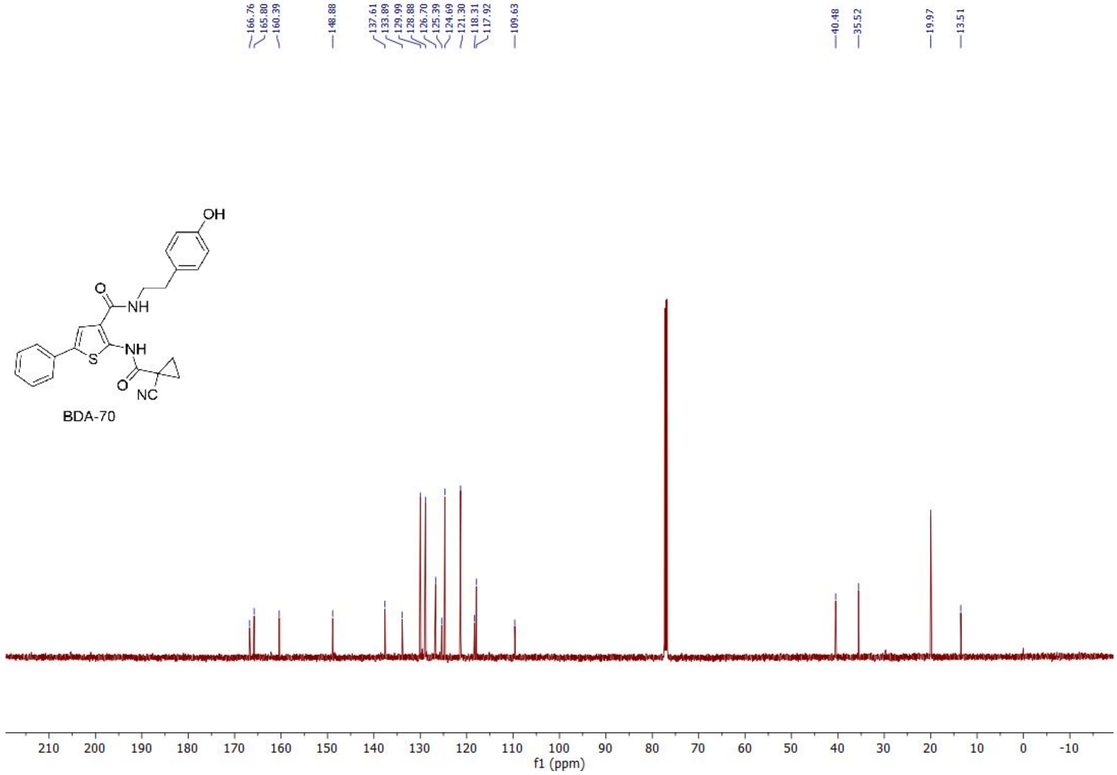

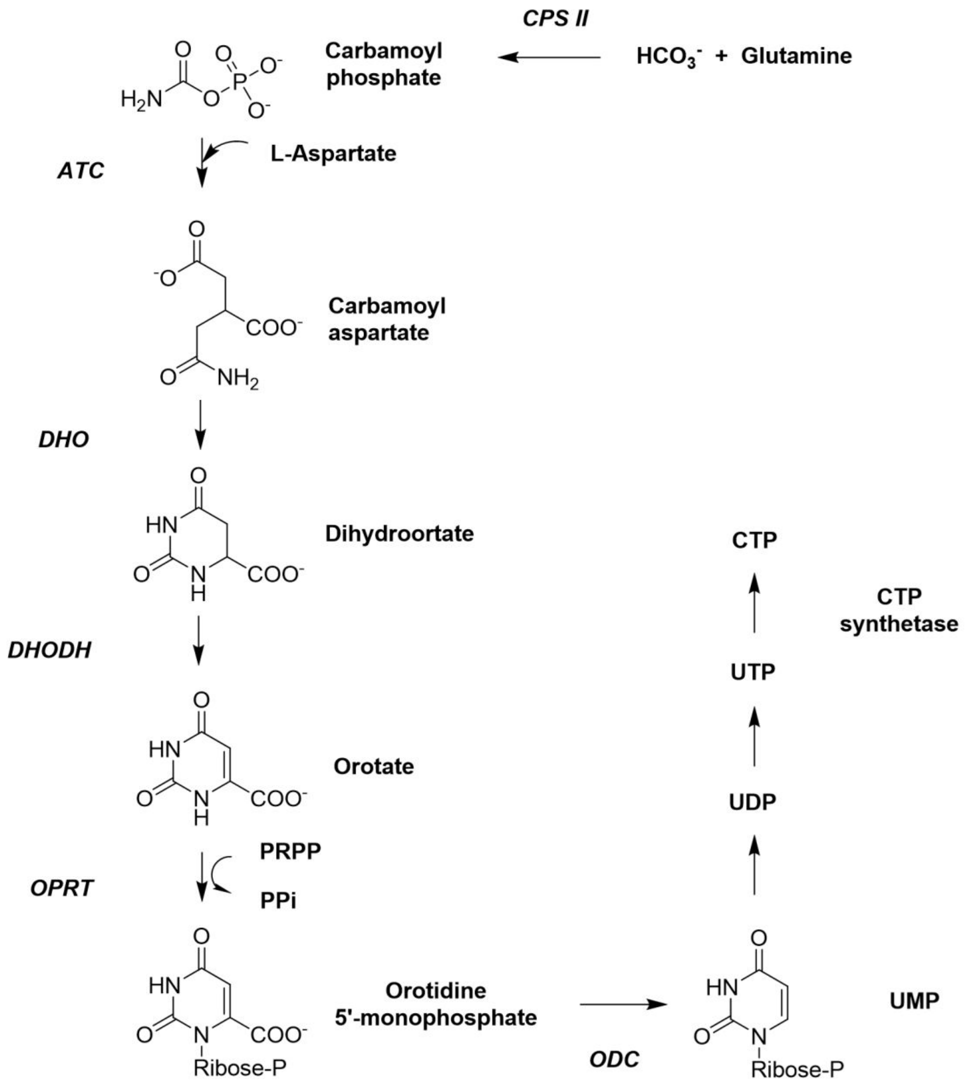

Supporting information Figure 1. The *de novo* pyrimidine biosynthesis pathway. Enzymes: CPS II, carbamoyl phosphate synthetase II; ATC, aspartate transcarbamoylase; DHO, dihydroorotase; DHODH, dihydroorotate dehydrogenase; OPRT, orotate phosphoribosyl transferase; ODC, orotidine 5’-monophosphate decarboxylase.

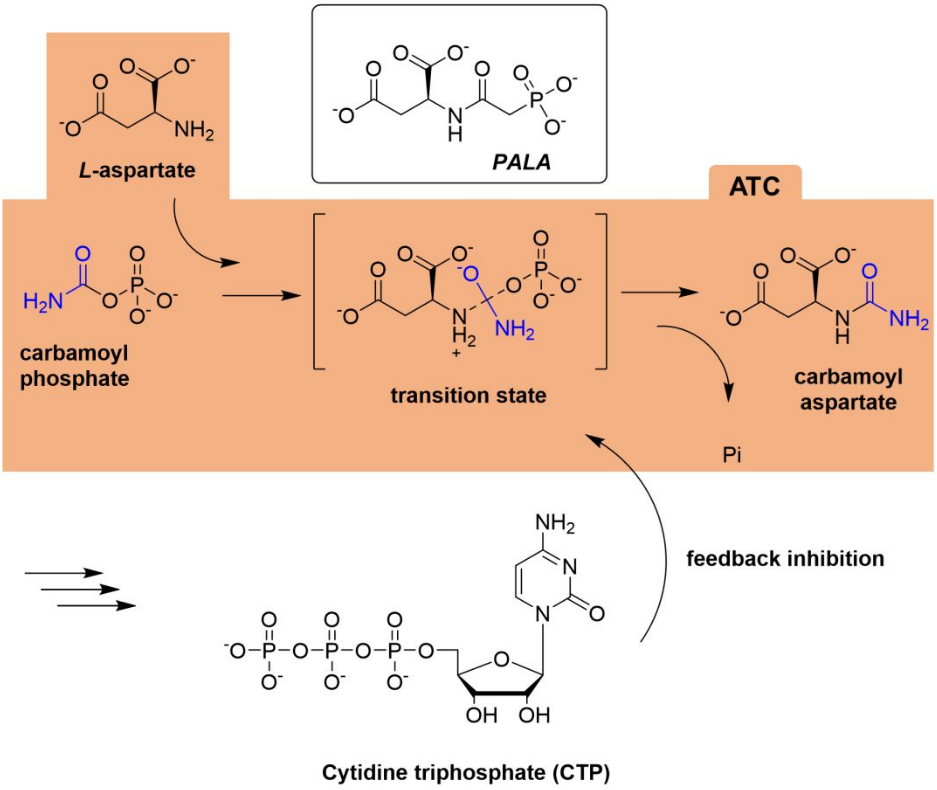

Supporting information Figure 2. The reaction catalyzed by ATC and feedback regulation mechanism in *de novo* pyrimidine biosynthesis pathway and structure of *PALA*. Aspartate Transcarbamoylase (ATC) combines L-aspartate and carbamoyl phosphate into carbamoyl aspartate through an enzyme stabilized transition state and inhibition feedback by CTP. The ATC inhibitor PALA closely resembles this transition state intermediate. CTP (a product of the pyrimidine biosynthesis pathway) provides feedback inhibition of ATC activity.

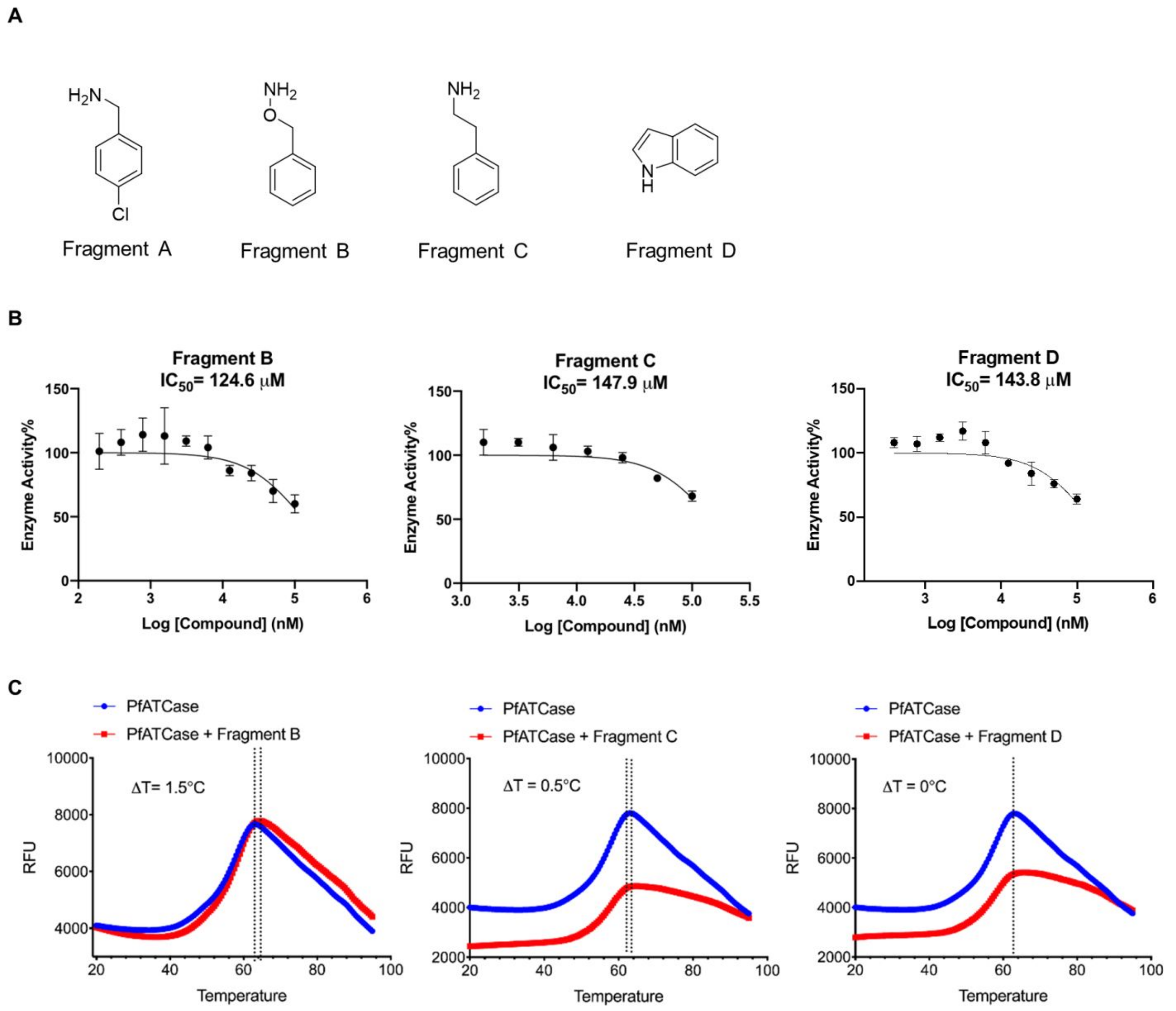

Supporting information Figure 3. Fragments bind directly to an allosteric pocket. (A) The chemical structure of Fragment A-D. (B) *In vitro* enzyme assay to assess the *in vitro* activity of Fragment B-D against *Pf*ATC (50 nM). (C) Differential scanning Fluorimentry (DSF) result showing the thermal stabilization of *Pf*ATC (blue) and *Pf*ATCase in presence of Fragment B, C and D (red).

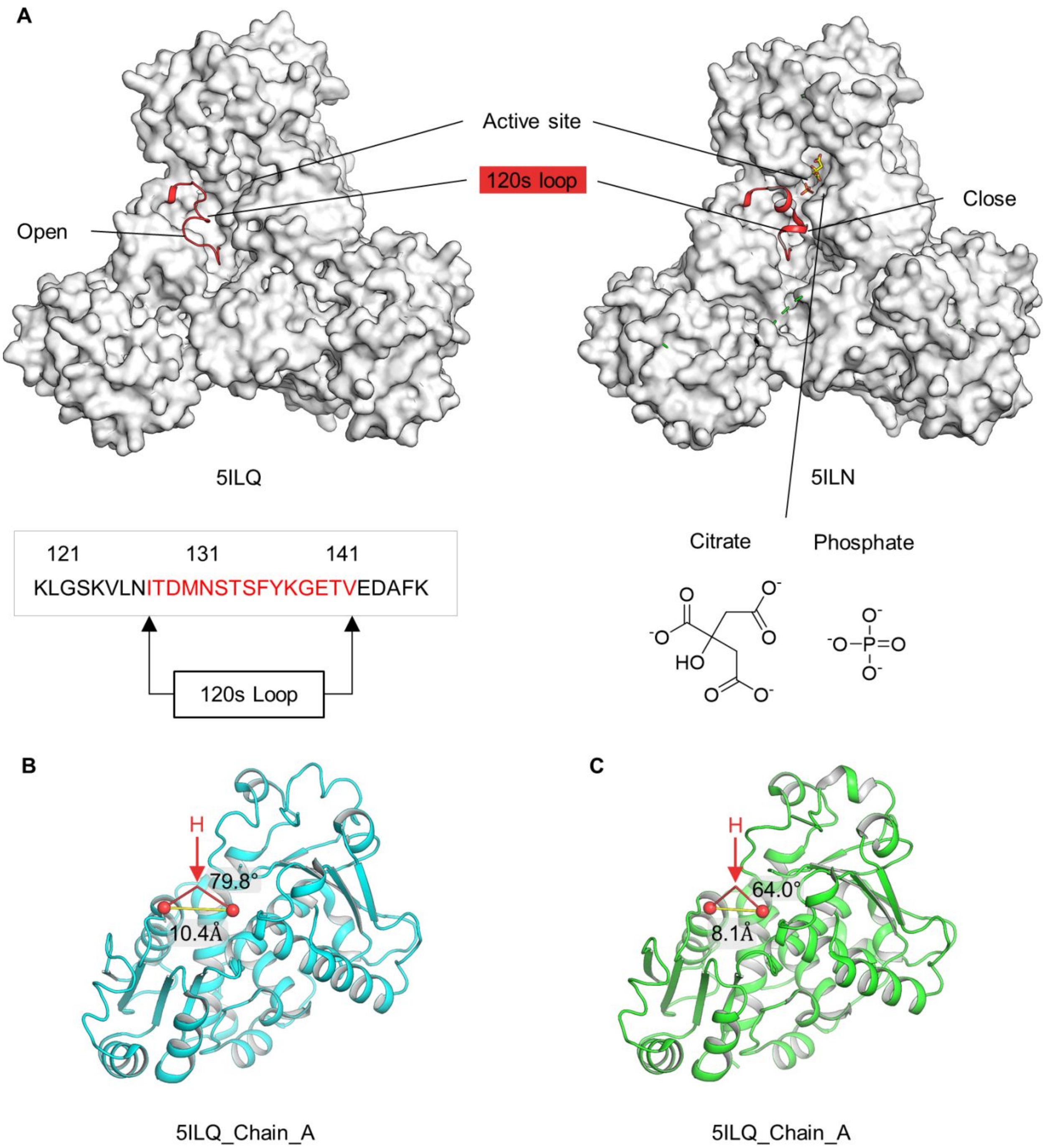

Supporting information Figure 4. Structure of T-state *Pf*ATC and R-state *Pf*ATC. (A) Apo-crystal structure of *Pf*ATC (left; PDB ID: 5ILQ) and the crystal structure of *Pf*ATC in complex with citrate (right; PDB ID: 5ILN), representing the T- and R-states, respectively. The 120s loop is highlighted in red, the open and close state of 120s loop are labeled. (B) Ribbon diagram representation of *Pf*ATC inactive monomer, the distance and angle between the domains are

measured from the center of mass of Asp and CP domain (red dot) and a hinge point. (C) Ribbon diagram representation of *Pf*ATC active monomer, the distance and angle between the domains are measured from the center of mass of Asp and CP domain (red dot) and a hinge point.

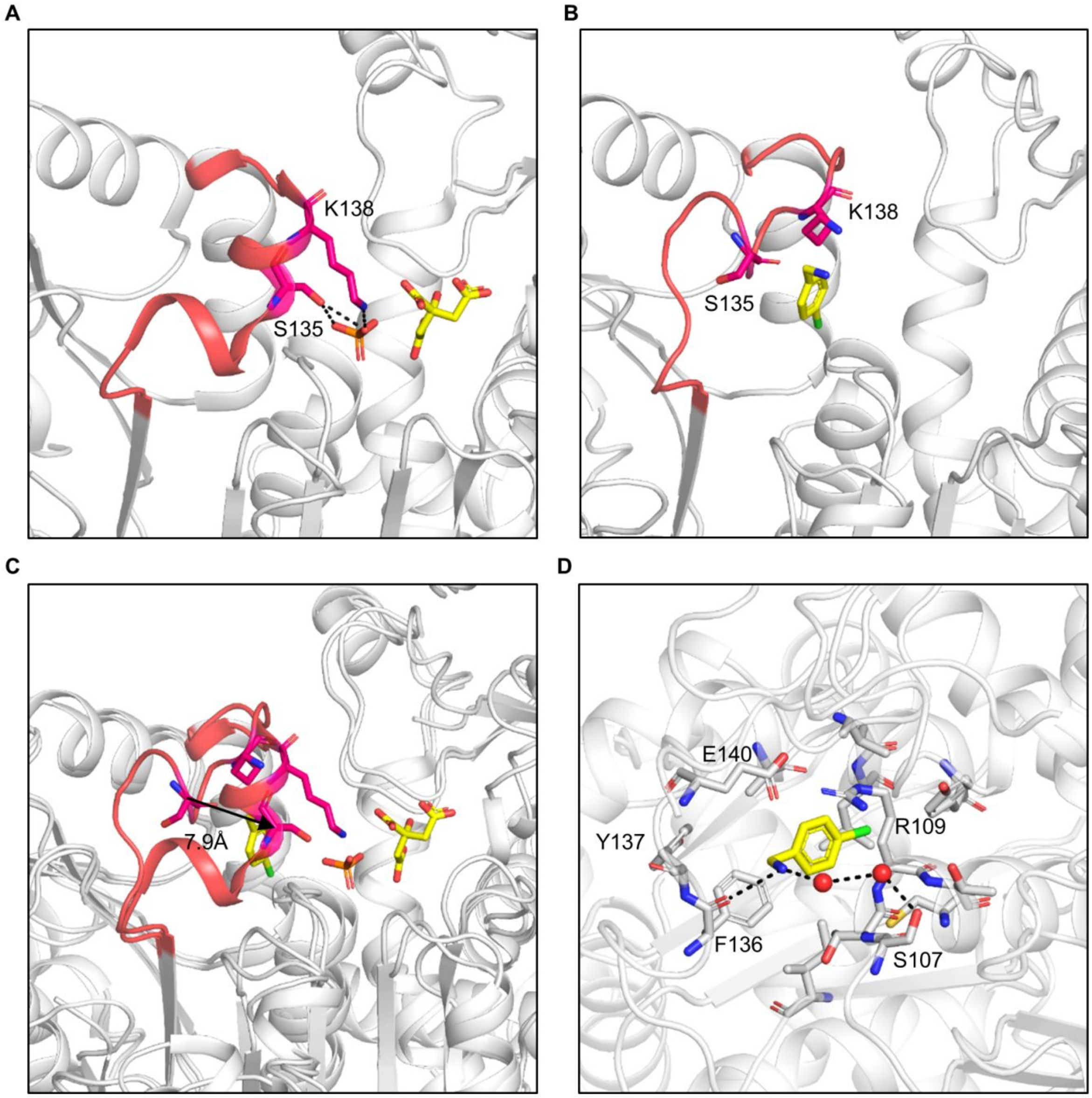

Supporting information Figure 5. Fragments target the allosteric pocket stabilize the inactive state of *Pf*ATC. (A) The crystal structure of the citrate:*Pf*ATC complex (PDB ID: 5ILN), representing the R-state (high substrate affinity, high activity) of *Pf*ATC, showing the important interactions of the 120s loop (highlighted in red) with phosphate. Citrate and phosphate are shown in sticks. (B) Crystal structure of the Fragment A:*Pf*ATC complex (PDB ID: 7ZCZ), representing the T-state (low substrate affinity, low activity) of *Pf*ATC, the residues Ser135 and Lys138 that form polar interactions with phosphate in the R-state *Pf*ATC are labeled. (C) Alignment of the crystal structure of Fragment A: *Pf*ATC complex (white; PDB ID: 7ZCZ) with the citrate:*Pf*ATC

complex (light blue), the RMSD on α-carbon is 0.226 Å, the 120s loop is highlighted in red. The 120s loop shifts by 8Å between the α-carbons of Ser135 in the T-state to R-state. (D) Ribbon and stick representation showing the key interactions between allosteric binding site of *Pf*ATC and Fragment A.

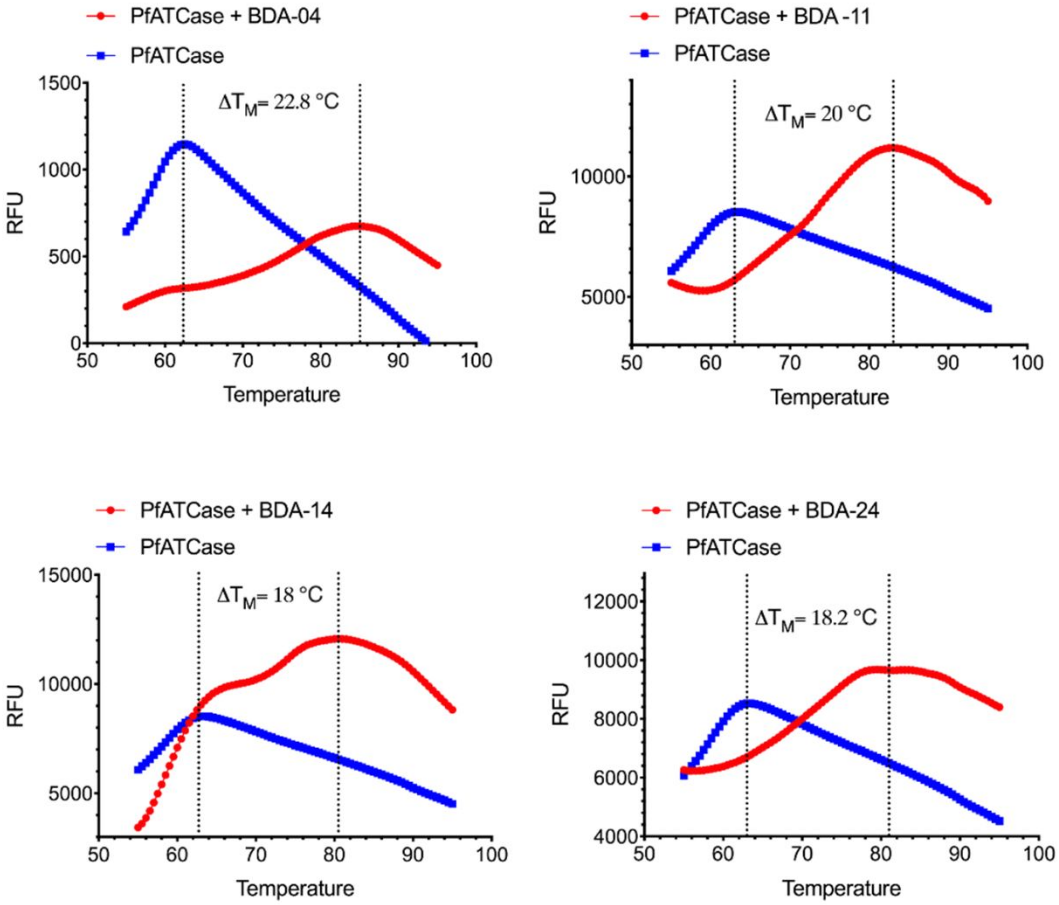

Supporting information Figure 6. Differential scanning Fluorimetry (DSF) results showing the thermal stabilization of *Pf*ATCase (blue) and *Pf*ATCase in presence of BDA-04, BDA-11, BDA-14, BDA-24. The T_M value of *Pf*ATCase increases by 22.8, 20, 18 and 18.2 degrees, respectively, after incubation with BDAs**.**

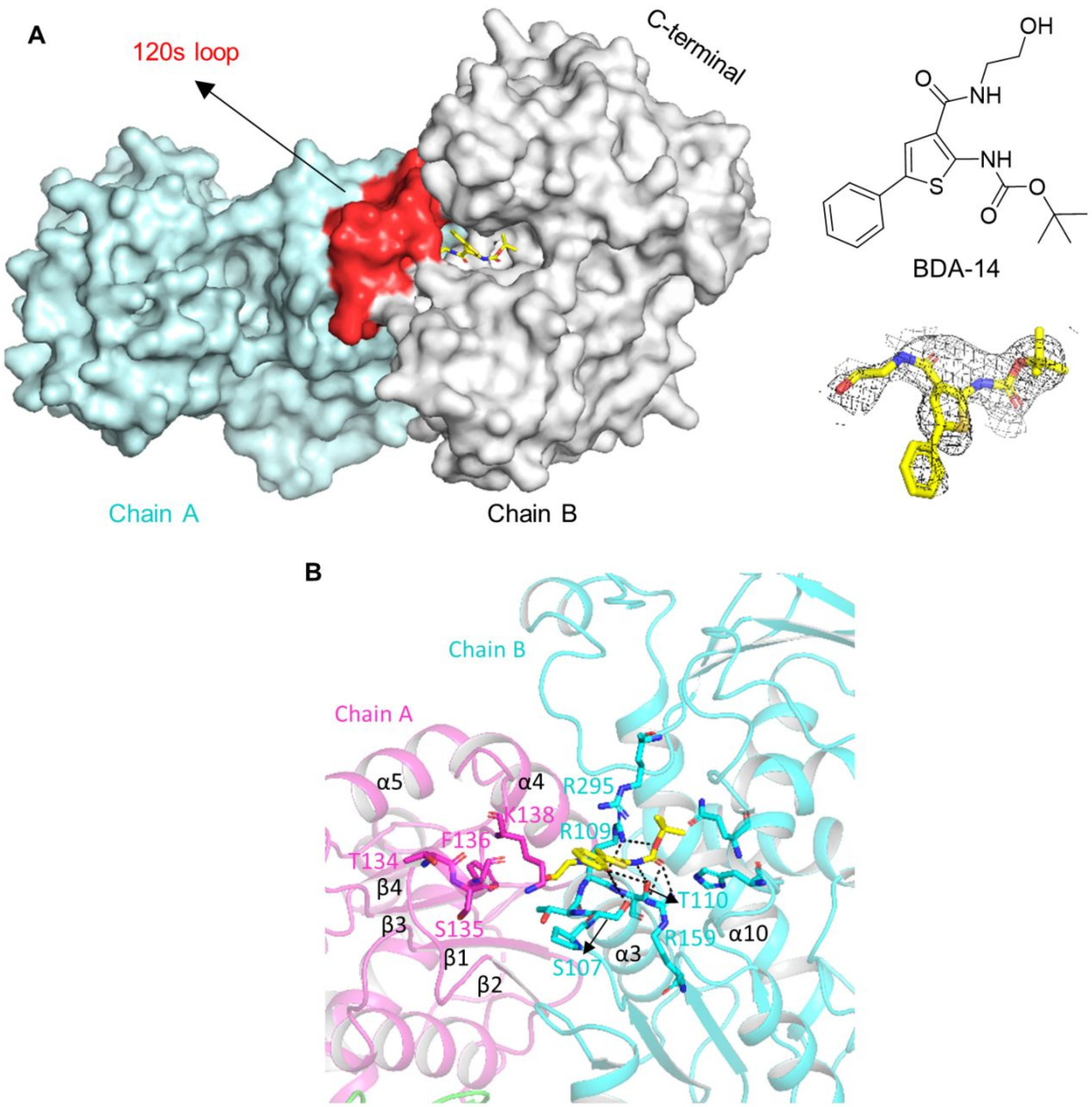

Supporting information Figure 7. BDA-14 binds at the allosteric pocket of *Pf*ATC. (A) Structure of *Pf*ATC in complex with BDA-14 showing the binding site of BDA-14. Two monomers of the trimer are shown. the 2Fc - Fo density map of BDA-04 is contoured at 0.7 σ. the chemical structure of BDA-14 (right). (B) A stick representation showing the key interactions between the binding site of *Pf*ATC and BDA-14.

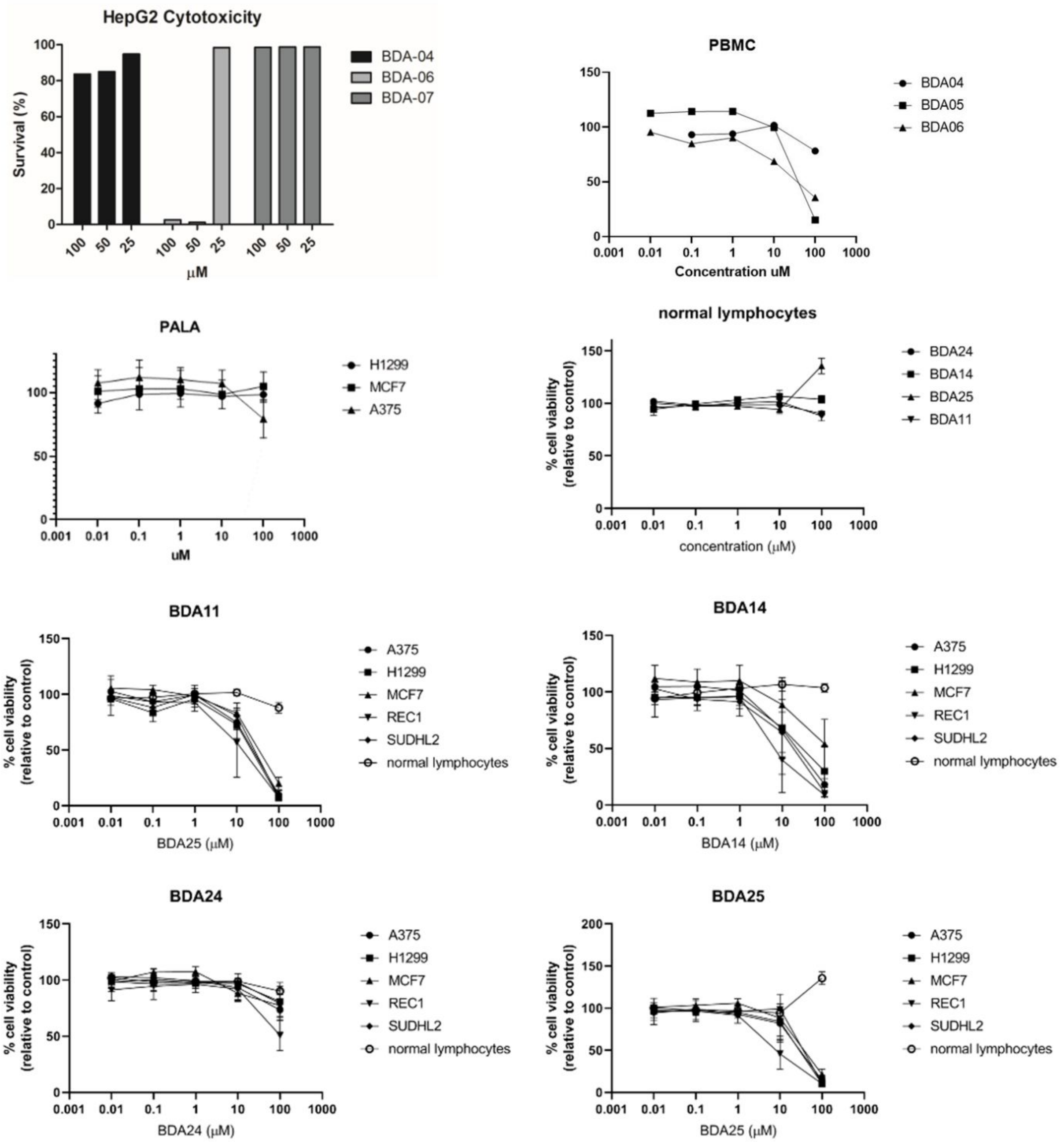

Supporting information Figure 8. Cytoxicity study of BDAs Dose-response of BDAs vs human cell lines.

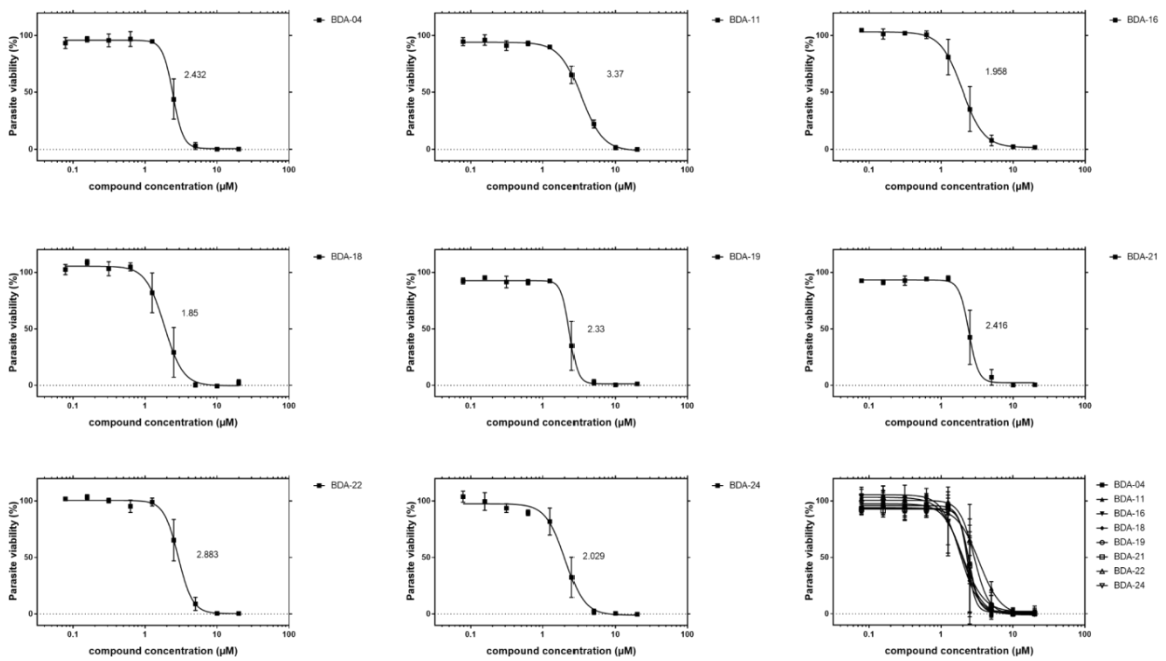

Supporting information Figure 9. Dose-response of BDAs vs ***P. falciparum*** 3D7(wild type) cell lines. EC_{50} value of BDA-04, BDA-11, BDA-16, BDA-18, BDA-19, BDA-21, BDA-22 and BDA-24 is 2.43 μM, 3.37 μM, 1.96 μM, 1.85 μM, 2.33 μM, 2.42 μM, 2.89 μM and 2.03 μM respectively.

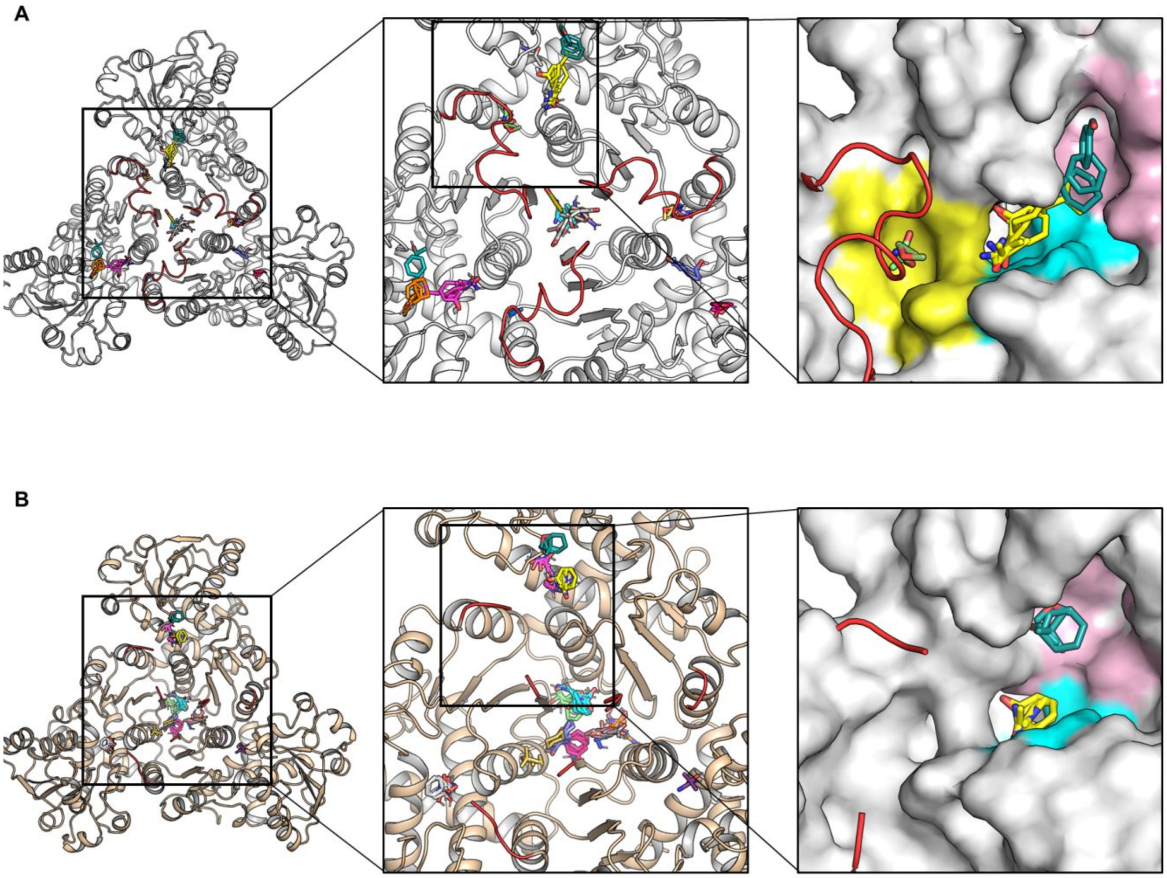

C

PDB entry	Structural notes	Allosteric site (site A)	Activity site (site B + C)	Other sites	Total probe cluster count
5ILQ	*Pf*ATC, apo	18	42	30	90
5G1O	*Hs*ATC, apo	0	27	68	95

Supporting information Figure 10. Potential off-target effect of BDA-04 supported by mapping the apo structure (apo-*Pf*ATC: 5ILQ; apo-*Hs*ATC: 5G1O) using FTMap. (A) Probe clusters in apo-*Pf*ATC crystal structure. Probe clusters are shown in sticks and 120s loop highlighted in red. Allosteric pocket (site A) and Active site (site B and site C) are colored as follows: site A, yellow; site B, blue; site C, pink. (B) Probe clusters in apo-*Hs*ATC crystal structure. Active site (site B and site C) are colored as follows: site B, blue; site C, pink. (C) Summary of FTMap mapping results. 18 probe clusters binding to *Pf*ATC allosteric pocket while 0 probe cluster binding to HsATCase allosteric pocket (120s loop of *Hs*ATC is partially missing as shown in (B)).

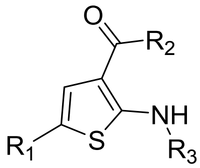

1859

Cpd	Structure	*Pf*ATC<br>IC_{50}(nM)	*Hs*ATC<br>IC_{50}(nM)
**BDA-01**	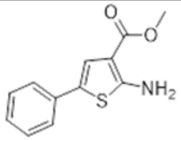	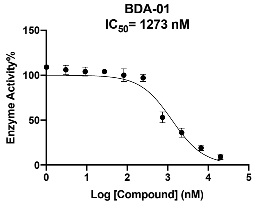	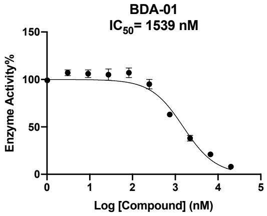
**BDA-02**	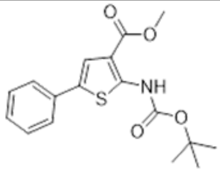	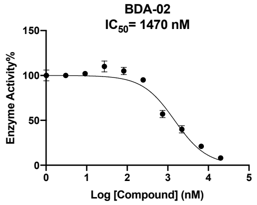	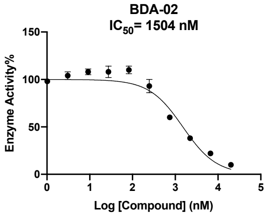
**BDA-03**	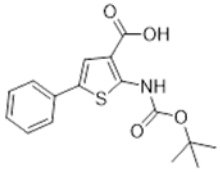	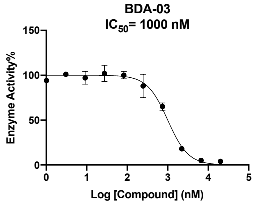	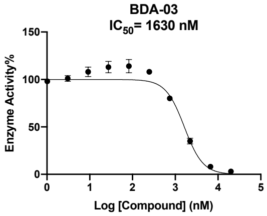
**BDA-04**	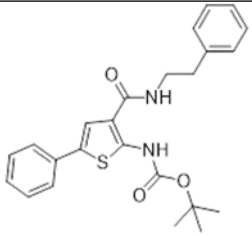	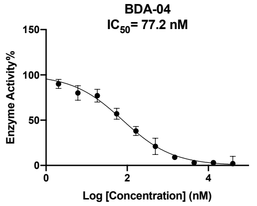	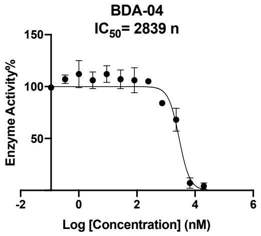
**BDA-05**	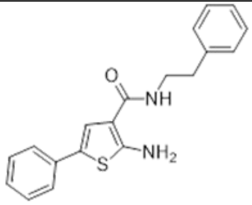	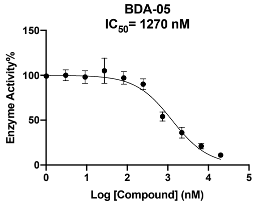	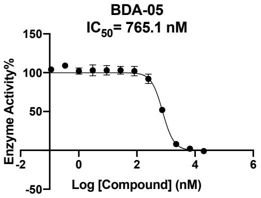

**BDA-06**	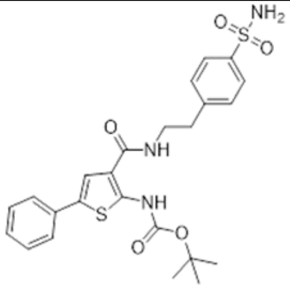	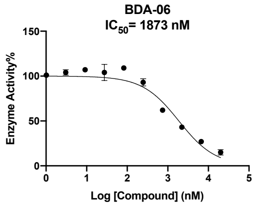	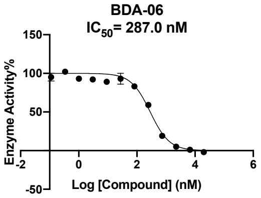
**BDA-07**	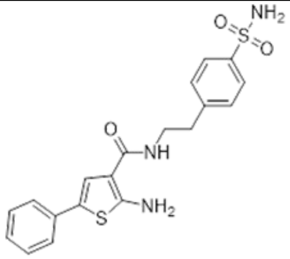	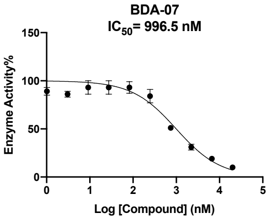	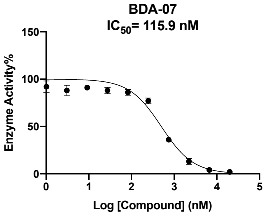
**BDA-08**	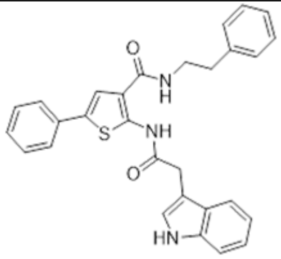	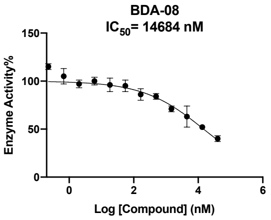	No
**BDA-09**	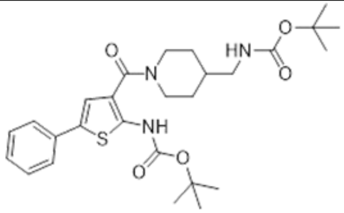	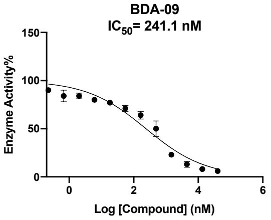	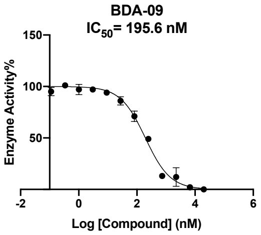
**BDA-10**	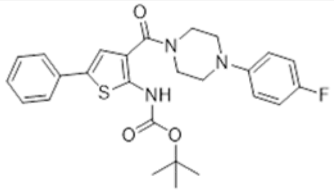	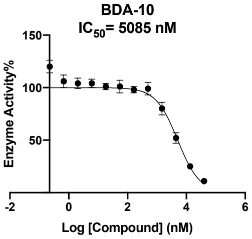	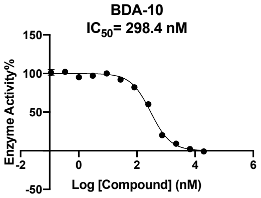
**BDA-11**	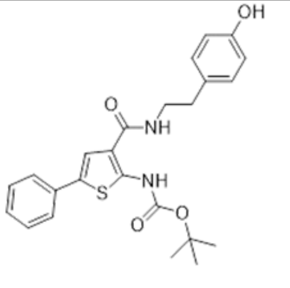	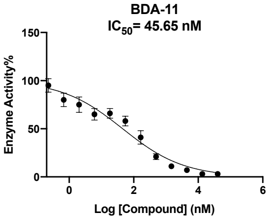	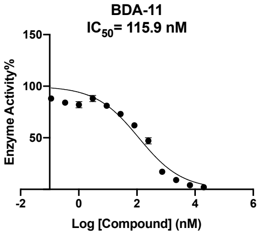

**BDA-12**	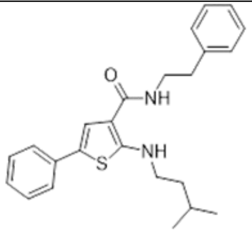	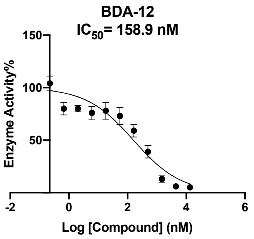	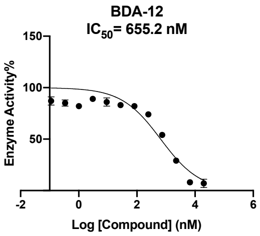
**BDA-13**	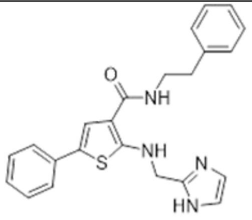	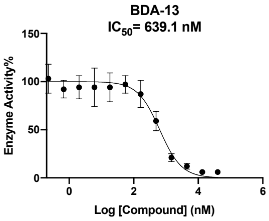	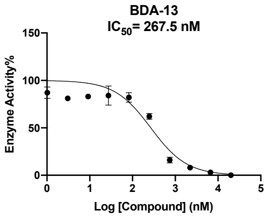
**BDA-14**	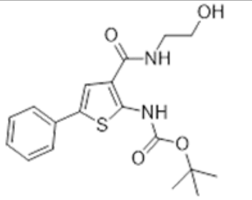	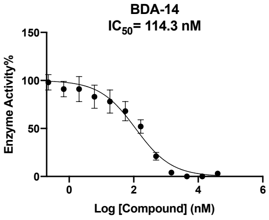	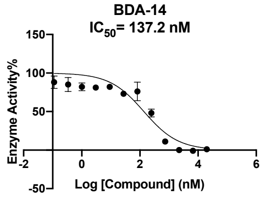
**BDA-15**	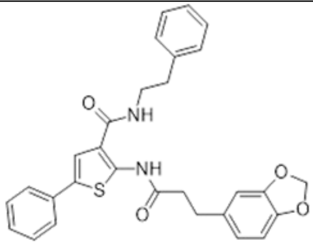	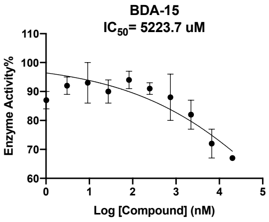	No
**BDA-16**	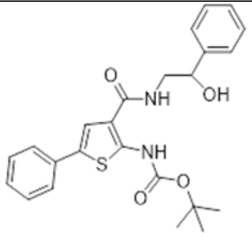	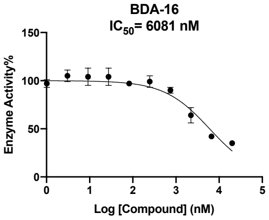	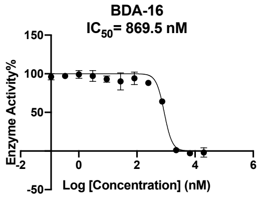
**BDA-17**	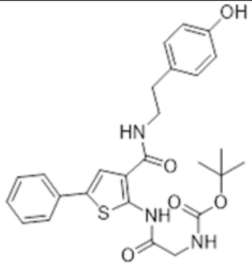	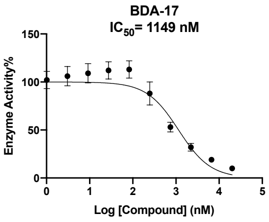	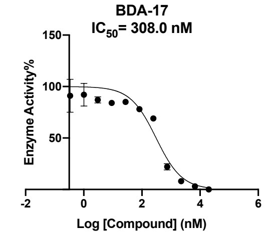

**BDA-18**	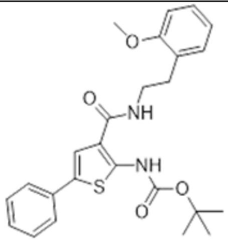	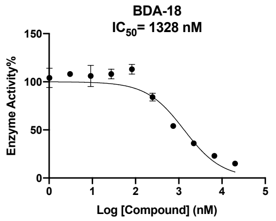	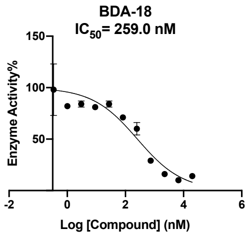
**BDA-19**	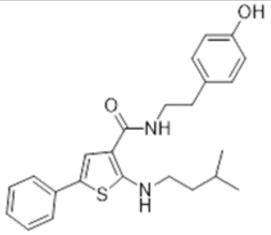	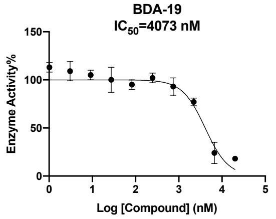	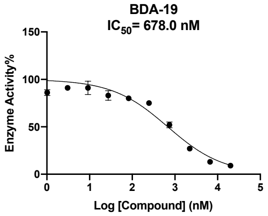
**BDA-20**	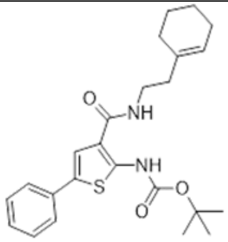	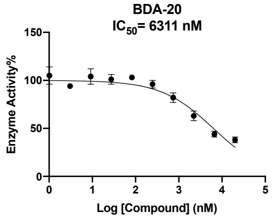	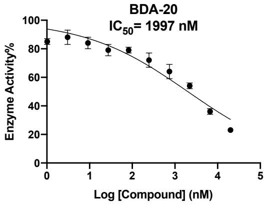
**BDA-21**	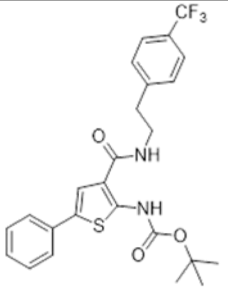	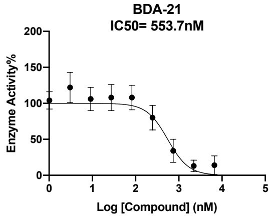	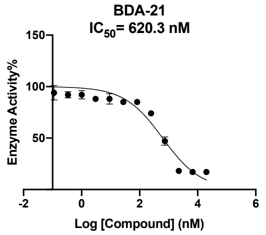
**BDA-22**	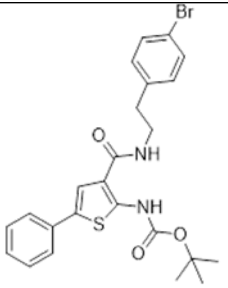	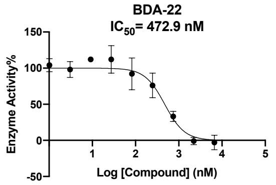	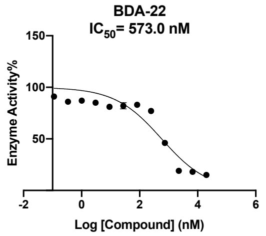
**BDA-23**	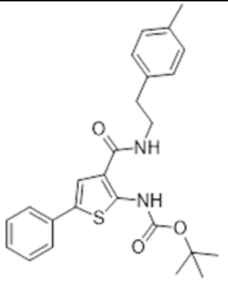	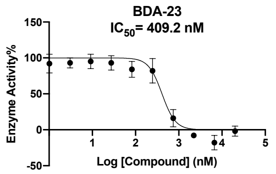	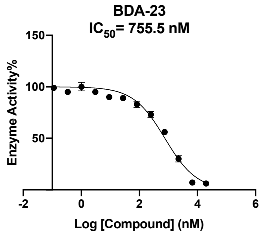

**BDA-24**	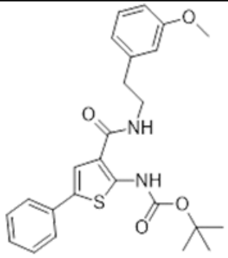	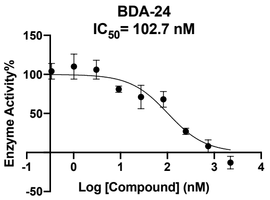	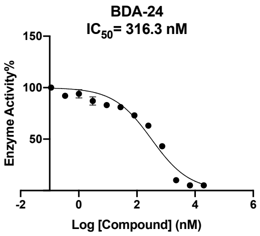
**BDA-25**	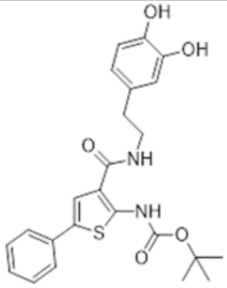	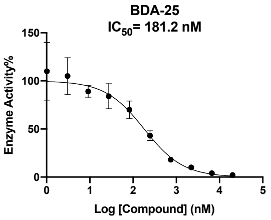	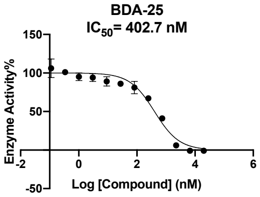
**BDA-26**	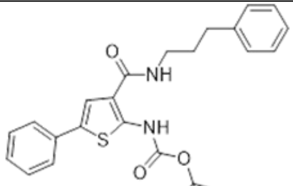	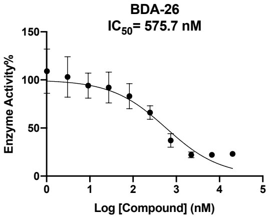	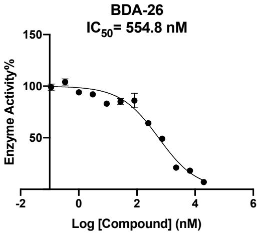
**BDA-27**	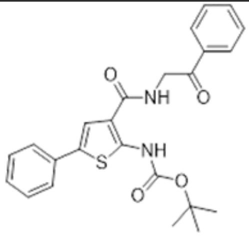	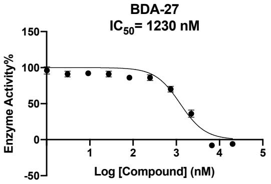	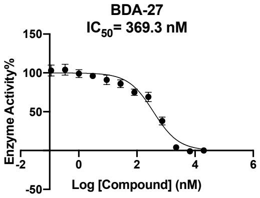
**BDA-28**	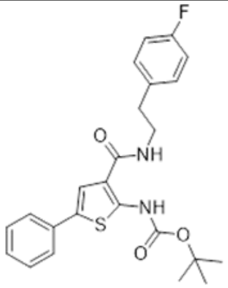	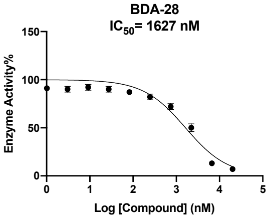	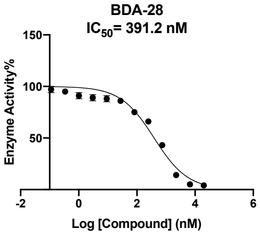

**BDA-29**	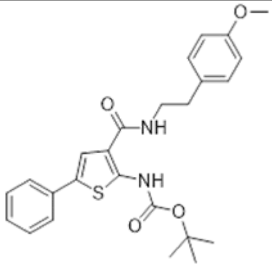	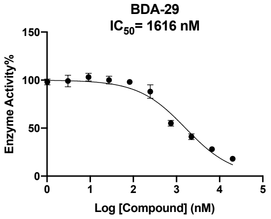	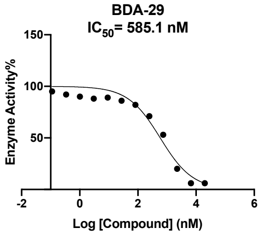
**BDA-30**	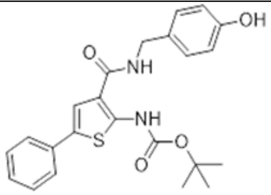	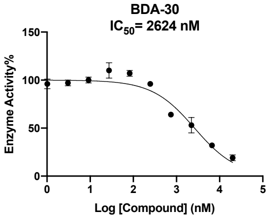	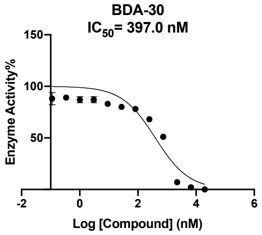
**BDA-31**	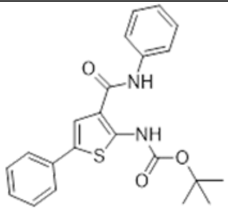	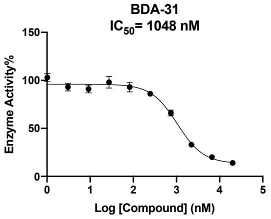	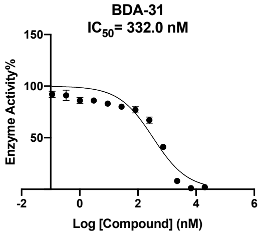
**BDA-32**	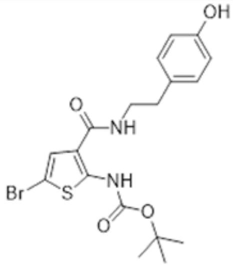	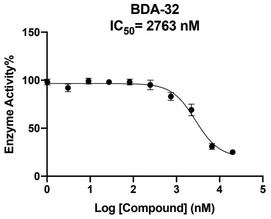	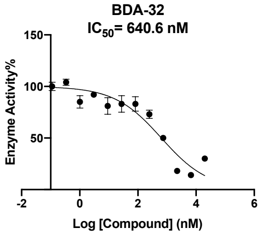
**BDA-33**	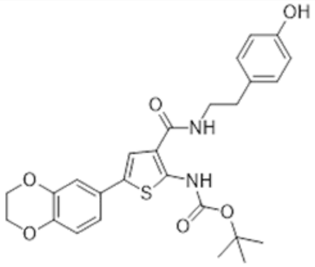	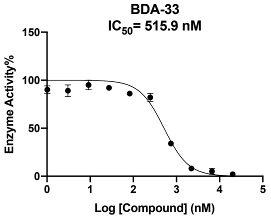	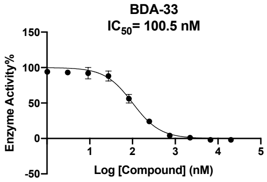
**BDA-34**	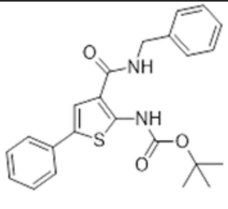	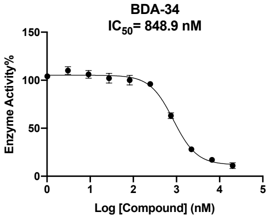	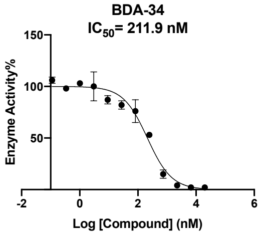

**BDA-35**	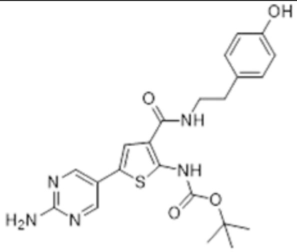	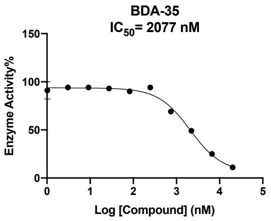	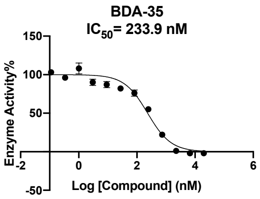
**BDA-36**	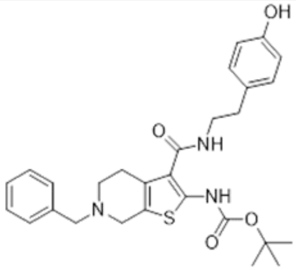	No	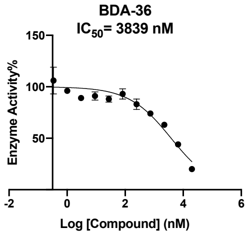
**BDA-37**	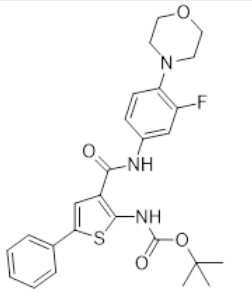	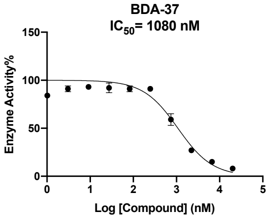	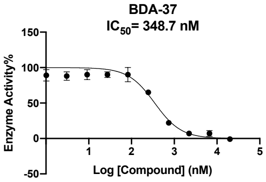
**BDA-38**	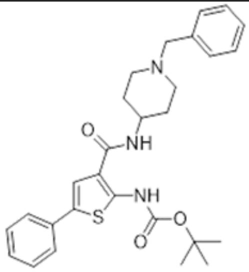	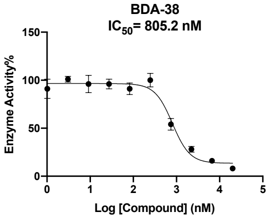	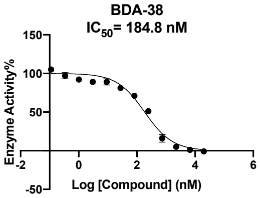
**BDA-39**	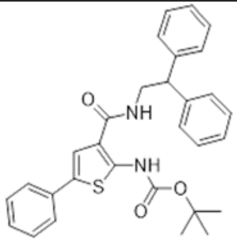	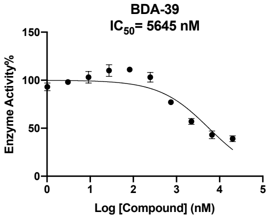	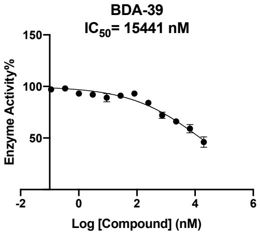

**BDA-40**	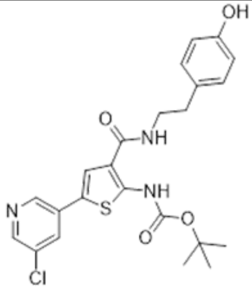	No	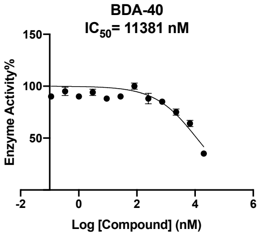
**BDA-41**	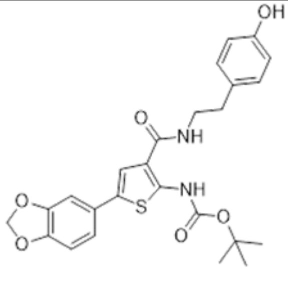	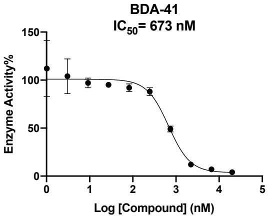	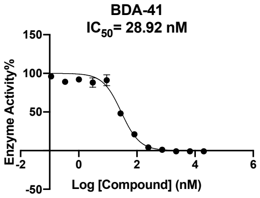
**BDA-42**	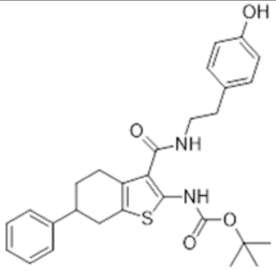	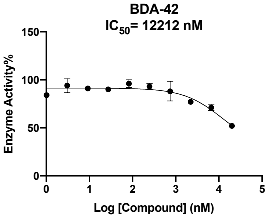	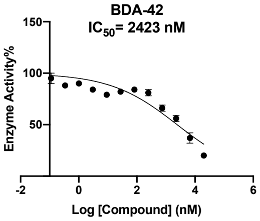
**BDA-43**	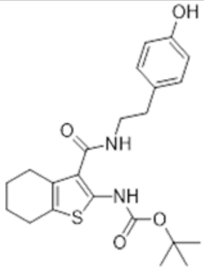	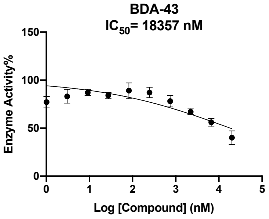	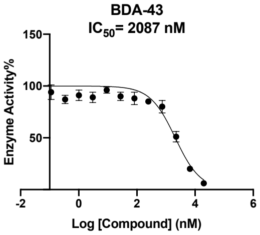
**BDA-44**	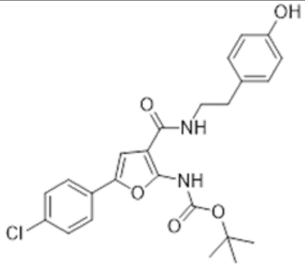	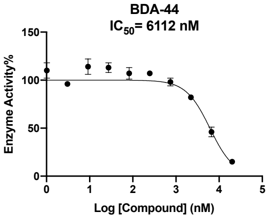	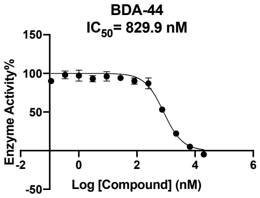

**BDA-45**	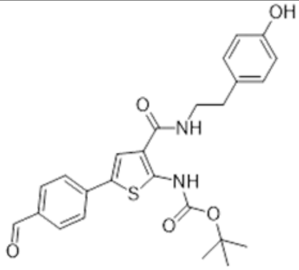	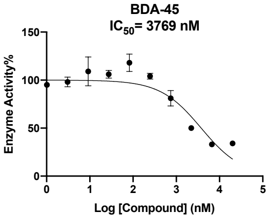	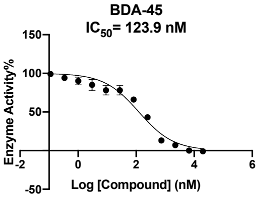
**BDA-46**	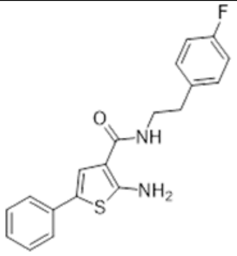	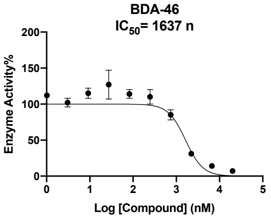	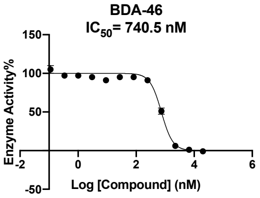
**BDA-47**	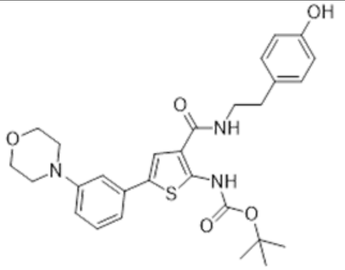	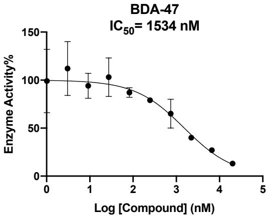	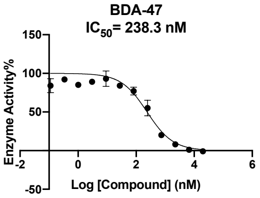
**BDA-48**	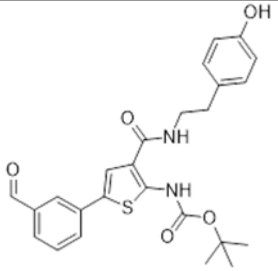	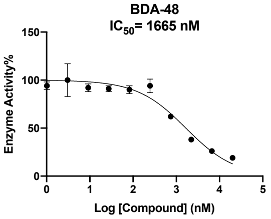	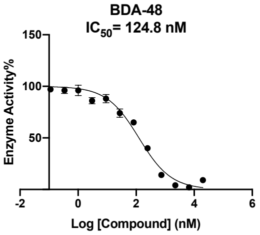
**BDA-49**	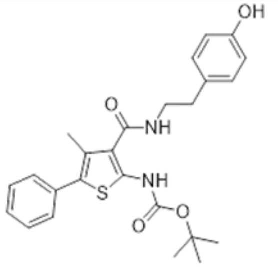	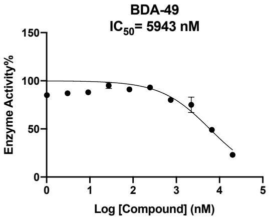	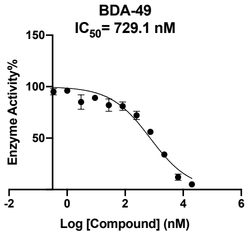

**BDA-50**	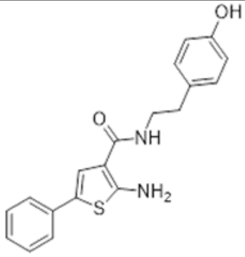	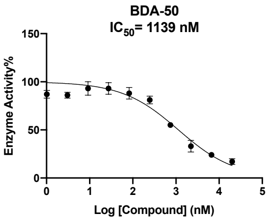	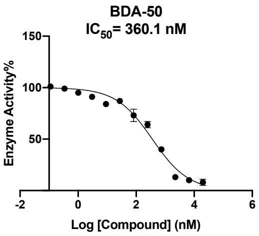
**BDA-51**	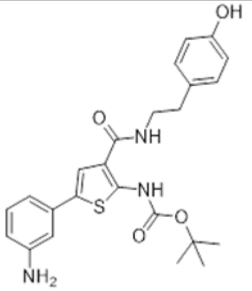	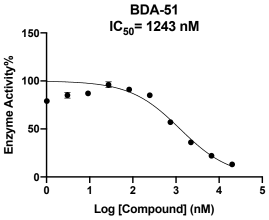	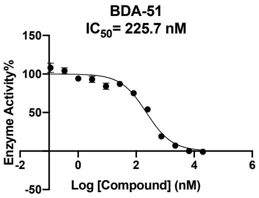
**BDA-52**	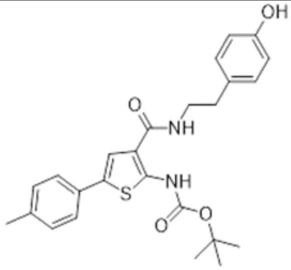	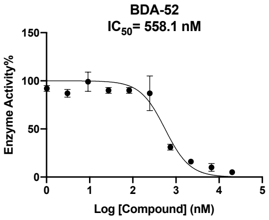	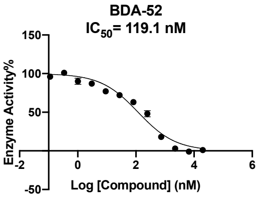
**BDA-53**	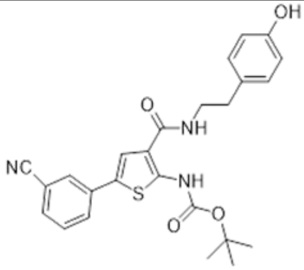	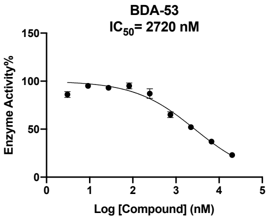	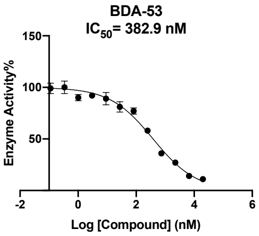
**BDA-54**	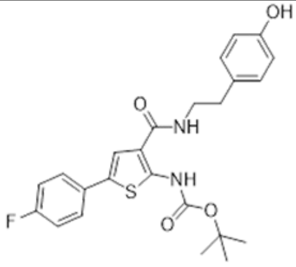	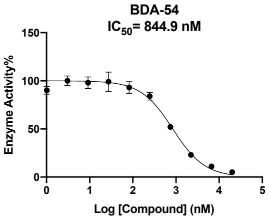	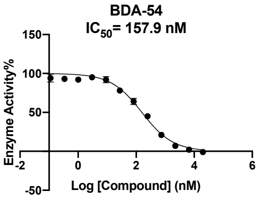

**BDA-55**	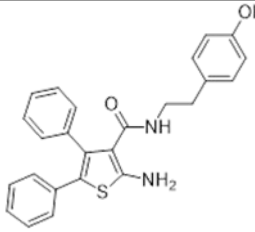	No	No
**BDA-56**	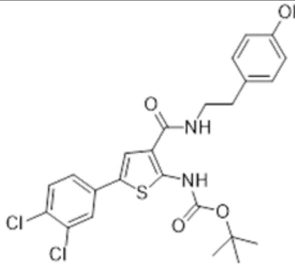	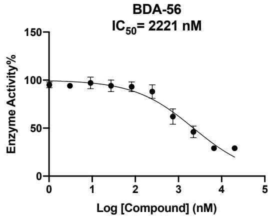	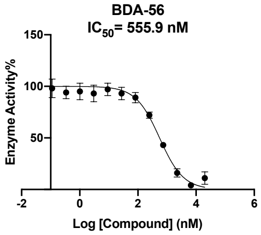
**BDA-57**	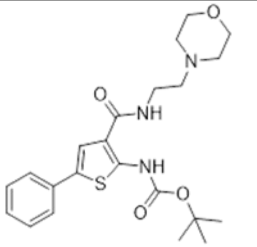	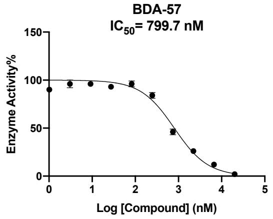	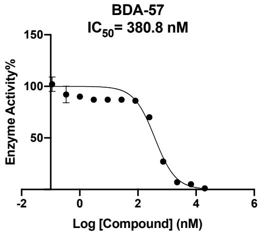
**BDA-58**	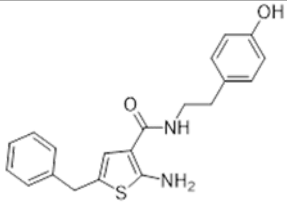	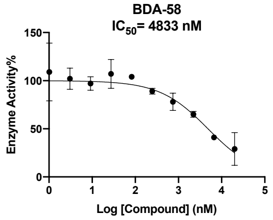	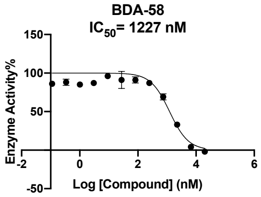
**BDA-59**	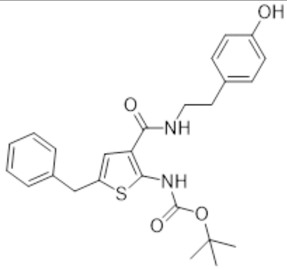	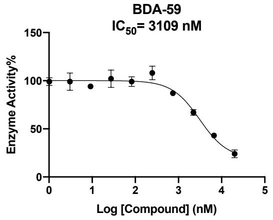	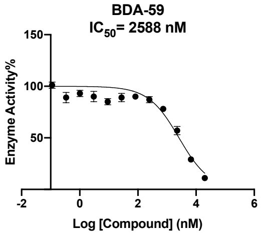
**BDA-60**	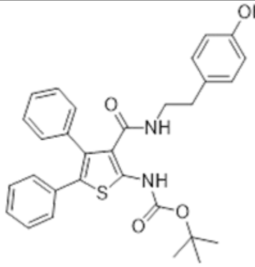	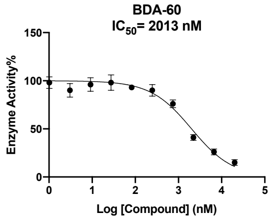	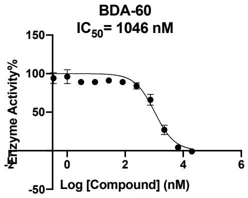

**BDA-61**	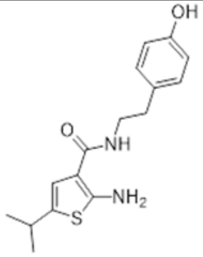	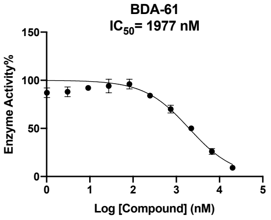	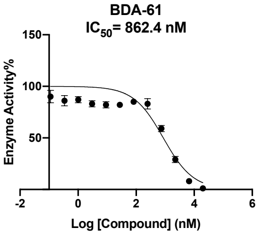
**BDA-62**	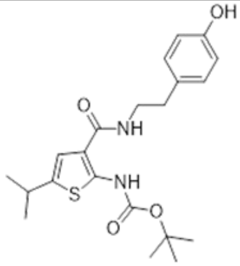	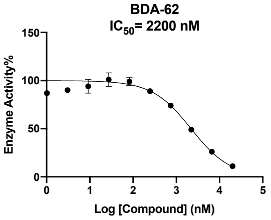	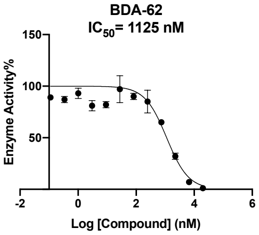
**BDA-63**	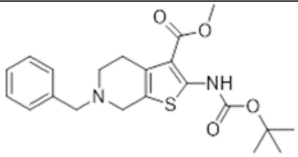	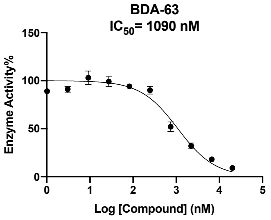	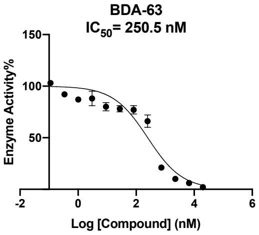
**BDA-64**	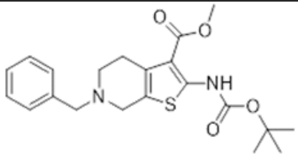	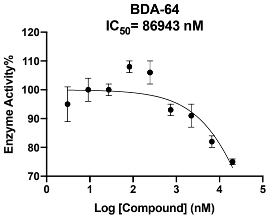	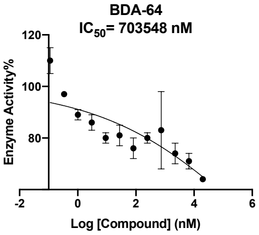
**BDA-65**	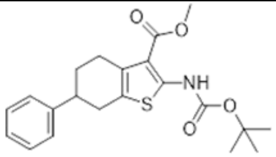	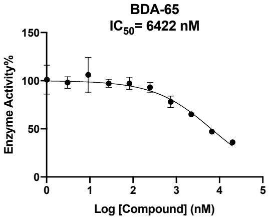	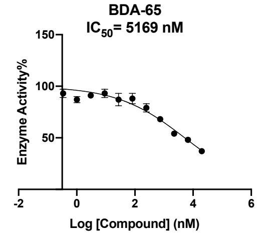
**BDA-66**	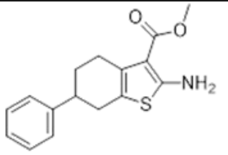	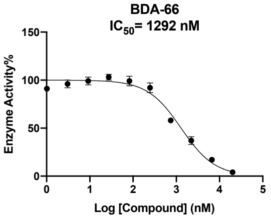	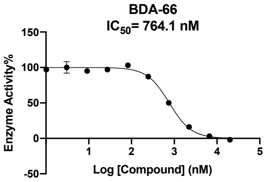

**BDA-67**	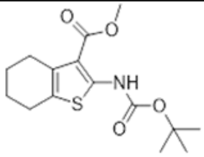	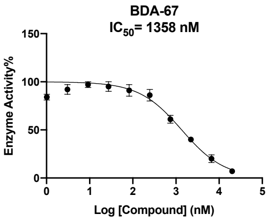	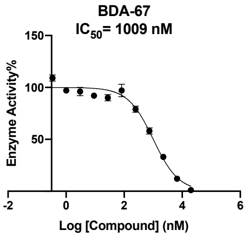
**BDA-68**	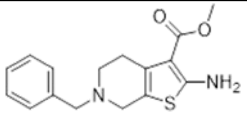	No	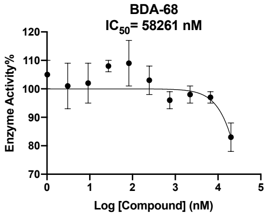
**BDA-69**	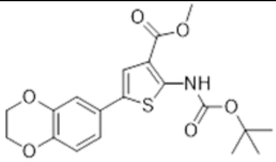	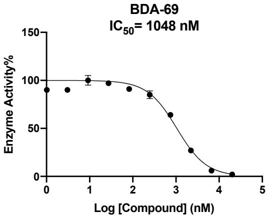	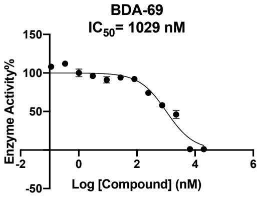
**BDA-70**	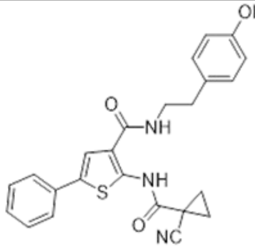	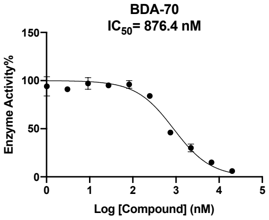	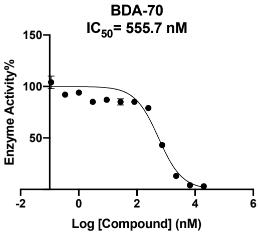

Supporting information Table 1. Chemical structure of BDAs with IC_{50} values against *Pf*ATC and *Hs*ATC.

Potency and Selectivity of selected BDAs				
**Compound code**	**Enzyme**	**IC_{50} values (nM)**	**Organism**	**EC_{50} (µM)**
**BDA-04**	*Pf*ATC	77.2 ± 1.1	*Pf* (3D7)	2.4
	HsATC	2839 ± 1.1	*Hs*	~1000
**BDA-11**	*Pf*ATC	46 ± 1.2	*Pf* (3D7)	3.4
	HsATC	116 ± 1.2	*Hs*	757.1
**BDA-14**	*Pf*ATC	115 ± 1.2	*Pf* (3D7)	42.5
	HsATC	137 ± 1.2	*Hs*	>1000
**BDA-24**	*PfATC*	103 ± 1.2	*Pf* (3D7)	2.0
	HsATC	316 ± 1.1	*Hs*	910.9

Pf Plasmodium falciparum, Hs homo sapiens (normal lymphocytes)

Supporting information Table 2. Shows the potency of selected compounds of the BDA series against isolated enzymes (IC_{50}s vs malarial ATC: *Pf*ATC; human ATC: *Hs*ATC) and EC_{50}s vs cell cultures.

Gene	Turncated *Pf*ATCase (wild type)
Source organism	*Plasmodium falciparum* strain 3D7
DNA source	pASK-IBA3-*Pf*ATC-full plasmid
Forward primer (*BsaI*)	5'-GCGCGCGGTCTCCAATGTTTTATATCAATAGCAAG-3'
Reverse primer (BsaI)	5'-GCGCGCGGTCTCCGCGCTGCTAGTTGATGAAAAAATGAG-3'
Expression vector	pASK-IBA3
Expression host	*Escherichia coli*
Complete amino acid sequence of the construct produced	MFYINSKYKIDLDKIMTKMKNKSVINIDDVDDEELLAILYTSKQFEKIL<br>KNNEDSKYLENKVFCSVFLEPSTRTRCSFDAAILKLGSKVLNITDMNS<br>TSFYKGETVEDAFKILSTYVDGIIYRDPSKKNVDIAVSSSSKPIINAGNG<br>TGEHPTQSLLDFYTIHNYFPFILDRNINKKLNIAFVGDLKNGRTVHSLS<br>KLLSRYNVSFNFVSCKSLNIPKDIVNTITYNLKKNNFYSDDSIKYFDNL<br>EEGLEDVHIIYMTRIQKERFTDVDEYNQYKNAFILSNKTLENTRDDTK<br>ILHPLPRVNEIKVEVDSNPKSVYFTQAENGLYVRMALLYLIFSSTSSAW<br>SHPQFEK

Gene	ATCase domain of CAD
Source organism	Homo sapiens
DNA source	pETM-41-HsATC-full plasmid
HsATCase<br>forward primer	agggcgccATGCTGCACTCATTAGTGG
HsATCase<br>reverse primer	cgaattcgCTAGAAACGGCCCAGCAC
pETM-41<br>forward primer	ccgtttctagCGAATTCGAGCTCCGTCG
pETM-41<br>reverse primer	gcagcatGGCGCCCTGAAAATAAAG
Expression vector	pETM-41
DNA source	*Escherichia coli* BL21(DE3)
Complete amino acid sequence of the construct produced	GPMSPLLHSLVGQHILSVQQFTKDQMSHLFNVAHTLRMMVQKERS<br>LDILKGKVMASMFYEVSTRTSSSFAAAMARLGGAVLSFSEATSSVQKG<br>ESLADSVQTMSCYADVVVLRHPQPGAVELAAKHCRRPVINAGDGVG<br>EHPTQALLDIFTIREELGTVNGMTITMVGDLKHGRTVHSLACLLTQYR<br>VSLRYVAPPSLRMPPTVRAFVASRGTKQEEFESIEEALPDTDVLYMTRI<br>QKERFGSTQEYEACFGQFILTPHIMTRAKKKMVVMHPMPRVNEISVE<br>VDSDPRAAYFRQAENGMYIRMALLATVLGRF

Supporting information Table 3. Macromolecule production information.

	*Pf*ATC + Fragment A/B/C/D	*Pf*ATC + Compound 1/BDA-04/BDA-14
Method	Sitting drop	Sitting drop
Plate type	96 - well	96 - well
Temperature (K)	293	293
Protein concentration (mg ml^{-1})	10	10
Buffer composition of protein solution	20 mM Tris pH 8.0, 300 mM NaCl, 10 mM Na-Malonate, 5% (v/v) glycerol, 3 mM BME	20 mM Tris pH 8.0, 300 mM NaCl, 10 mM Na-Malonate, 5% (v/v) glycerol, 3 mM BME, 1 mM compound 1/BDA-04/BDA-14 (Final DMSO concentration 5% (v/v))
Cyrstallization condition	0.1 M bis-tris propane pH 7.5, 0.2 M Na2SO4, 15% (w/v) PEG 3350<br>10 mM fragment A/B/C/D<br>(Final DMSO concentration 10% (v/v))	0.1 M bis-tris propane pH 7.5, 0.2 M Na2SO4, 15% (w/v) PEG 3350<br>1 mM compound 1/BDA-04/BDA-14<br>(Final DMSO concentration 5% (v/v))
Volume and ratio of drop	2 µL (1:1)	2 µL (1:1)
Volume of reservior	50 µL	50 µL

Supporting information Table 4. Cyrstallization conditions of *Pf*ATCase crystal grown in presence of ligand.

Protein/Ligand	*Pf*ATC+ Fragment A	*Pf*ATC+ Fragment B	*Pf*ATC+ Fragment C	*Pf*ATC+ Fragment D	*Pf*ATC+ Cpd 1	*Pf*ATC+ BDA-04	*Pf*ATC+ BDA-14
**PDB entry**	7ZCZ	7ZEA	7ZGS	7ZHI	7ZST	7ZP2	7ZID
**Data collection**							
Space group	P 1 21 1	P 1 21 1	P 1 21 1	P 1 21 1	I 1 21 1	P 1	I 1 2 1
Cell dimensions							
a, b, c (Å)	a=86.66Å b=103.63Å c=86.86Å	a=87.03 b=104.51Å c=87.30Å	a=86.85Å b=104.37Å c=87.05Å	a=86.71Å b=103.82Å c=87.07Å	a=119.28Å b=89.73Å c=137.05Å	a=87.06Å b=87.36Å c=104.64Å	a=119.26Å b=90.02Å c=136.94Å
α, β, γ (°)	α=90.00° β=117.45° γ=90.00°	α=90.00° β=117.47° γ=90.00°	α=90.00° β=117.53° γ=90.00°	α=90.00° β=117.92° γ=90.00°	α=90.00° β=109.22° γ=90.00°	α=89.98° β=90.04° γ=117.72°	α=90.00° β=108.87° γ=90.00°
Resolution (Å)	42.42 - 2.45	45.28 - 2.45	45.13 - 2.35	43.54 - 2.95	49.50 - 2.50	62.19 - 2.30	45.56 - 2.80
R_{merge}	0.04	0.05	0.04	0.09	0.03	0.06	0.04
I/σ	1.53 (2.45Å)	1.54 (2.45Å)	1.71 (2.34Å)	1.89 (2.95Å)	2.01 (2.51Å)	2.45 (2.29Å)	1.15 (2.29Å)
Completeness(%)	99.1 (85.5)	99.3 (96.3)	98.3 (95.0)	98.3 (95.0)	99.9 (99.8)	96.0 (96.0)	97.7 (95.1)
redundancy	3.4 (3.3)	3.4 (3.3)	3.4 (3.2)	3.3 (3.2)	2.0 (2.0)	1.7 (1.8)	1.9 (1.9)
**Refinement**							
Resolution(Å)	2.45 Å	2.45 Å	2.35 Å	2.95 Å	2.50 Å	2.30 Å	2.80 Å
No.Reflections (unique)	49863	50829	56531	28662	47372	117672	59389
R_{work}/R_{free}	0.177/0.216	0.185/0.223	0.218/0.278	0.192/0.235	0.241/0.284	0.166/0.193	0.187/0.236
No. Atoms							
Protein	16380	16261	16261	16380	15550	32760	15689
Ligand	18	18	23	16	35	56	47
Water	163	163	59	31	44	44	16
B-factors							
Protein	65.84	57.57	65.3	74.77	60.91	58.92	81.91
Ligand	65.84	75.8	73.12	100.56	76.72	65.28	114.0
Water	52.82	45.75	53.09	48.6	43.18	47.15	55.75
R.m.s deviations							
Bond lengths(Å)	0.0121	0.0126	0.0118	0.0094	0.0095	0.0144	0.0103
Bond angles(°)	1.862	1.870	1.874	1.731	1.746	2.037	1.712

Supporting information Table 5. Data collection and model refinement statistics.

References

25. Krug, M.; Weiss, M. S.; Heinemann, U.; Mueller, U., XDSAPP: a graphical user interface for the convenient processing of diffraction data using XDS. *Journal of Applied Crystallography* **2012,** *45* (3), 568-572.

26. Winn, M. D.; Ballard, C. C.; Cowtan, K. D.; Dodson, E. J.; Emsley, P.; Evans, P. R.; Keegan, R. M.; Krissinel, E. B.; Leslie, A. G.; McCoy, A., Overview of the CCP4 suite and current developments. *Acta Crystallographica Section D: Biological Crystallography* **2011,** *67* (4), 235-242.

27. Emsley, P.; Lohkamp, B.; Scott, W. G.; Cowtan, K., Features and development of Coot. *Acta Crystallographica Section D: Biological Crystallography* **2010,** *66* (4), 486-501.

28. Murshudov, G. N.; Vagin, A. A.; Dodson, E. J., Refinement of macromolecular structures by the maximum-likelihood method. *Acta Crystallographica Section D: Biological Crystallography* **1997,** *53* (3), 240-255.

29. Berman, H. M.; Westbrook, J.; Feng, Z.; Gilliland, G.; Bhat, T. N.; Weissig, H.; Shindyalov, I. N.; Bourne, P. E., The protein data bank. *Nucleic acids research* **2000,** *28* (1), 235-242.

30. Chin, W.; Moss, D.; Collins, W. E., The continuous cultivation of Plasmodium fragile by the method of Trager-Jensen. *The American Journal of Tropical Medicine and Hygiene* **1979,** *28* (3), 591-592.

31. Das Gupta, R.; Krause-Ihle, T.; Bergmann, B. r.; Müller, I. B.; Khomutov, A. R.; Müller, S.; Walter, R. D.; Lüersen, K., 3-Aminooxy-1-aminopropane and derivatives have an antiproliferative effect on cultured Plasmodium falciparum by decreasing intracellular polyamine concentrations. *Antimicrobial agents and chemotherapy* **2005,** *49* (7), 2857-2864.

32. Smilkstein, M.; Sriwilaijaroen, N.; Kelly, J. X.; Wilairat, P.; Riscoe, M., Simple and inexpensive fluorescence-based technique for high-throughput antimalarial drug screening. *Antimicrobial agents and chemotherapy* **2004,** *48* (5), 1803-1806.

33. Meissner, K. A.; Kronenberger, T.; Maltarollo, V. G.; Trossini, G. H. G.; Wrenger, C., Targeting the Plasmodium falciparum plasmepsin V by ligand-based virtual screening. *Chemical Biology & Drug Design* **2019,** *93* (3), 300-312.
